# Supplementary material for: Effect of different visual presentations on the public’s comprehension of prognostic information using acute and chronic condition scenarios: two online randomised controlled trials
Source: BMJ Open. 2023 Jun 14;13(6):e067624. doi: 10.1136/bmjopen-2022-067624 (PMC10277048; doi:10.1136/bmjopen-2022-067624)
Supplement: Supplementary data [file bmjopen-2022-067624supp001.pdf]

Additional file 1

(Supplementary methods)

CONSORT checklist **2**

Dynata (demographics in Australia, recruitment strategies) **5**

Interventions (visual presentations)

- **Trial A (Acute otitis media)** **6**
- **Trial B (Lateral epicondylitis)** **9**

The flow of the trials and outcome measurements **11**

Survey **12**

Abukmail E, *et al. BMJ Open* 2023; 13:e067624. doi: 10.1136/bmjopen-2022-067624

CONSORT 2010 checklist of information to include when reporting a randomised trial*

Section/Topic	Item No	Checklist item	Reported on page No
**Title and abstract**			
	1a	Identification as a randomised trial in the title	1
	1b	Structured summary of trial design, methods, results, and conclusions (for specific guidance see CONSORT for abstracts)	2-3
**Introduction**			
Background and objectives	2a	Scientific background and explanation of rationale	4-5
	2b	Specific objectives or hypotheses	5
**Methods**			
Trial design	3a	Description of trial design (such as parallel, factorial) including allocation ratio	5
	3b	Important changes to methods after trial commencement (such as eligibility criteria), with reasons	9
Participants	4a	Eligibility criteria for participants	5
	4b	Settings and locations where the data were collected	5
Interventions	5	The interventions for each group with sufficient details to allow replication, including how and when they were actually administered	6-7
Outcomes	6a	Completely defined pre-specified primary and secondary outcome measures, including how and when they were assessed	7-8
	6b	Any changes to trial outcomes after the trial commenced, with reasons	NA
Sample size	7a	How sample size was determined	8
	7b	When applicable, explanation of any interim analyses and stopping guidelines	NA
Randomisation:			
Sequence generation	8a	Method used to generate the random allocation sequence	6
	8b	Type of randomisation; details of any restriction (such as blocking and block size)	6
Allocation concealment mechanism	9	Mechanism used to implement the random allocation sequence (such as sequentially numbered containers), describing any steps taken to conceal the sequence until interventions were assigned	6
Implementation	10	Who generated the random allocation sequence, who enrolled participants, and who assigned participants to interventions	6

Blinding	11a	If done, who was blinded after assignment to interventions (for example, participants, care providers, those assessing outcomes) and how	10
	11b	If relevant, description of the similarity of interventions	6-7
Statistical methods	12a	Statistical methods used to compare groups for primary and secondary outcomes	9
	12b	Methods for additional analyses, such as subgroup analyses and adjusted analyses	9-10
**Results**			
Participant flow (a diagram is strongly recommended)	13a	For each group, the numbers of participants who were randomly assigned, received intended treatment, and were analysed for the primary outcome	Figure 3
	13b	For each group, losses and exclusions after randomisation, together with reasons	Figure 3
Recruitment	14a	Dates defining the periods of recruitment and follow-up	5
	14b	Why the trial ended or was stopped	NA
Baseline data	15	A table showing baseline demographic and clinical characteristics for each group	Table 1
Numbers analysed	16	For each group, number of participants (denominator) included in each analysis and whether the analysis was by original assigned groups	Tables and figures
Outcomes and estimation	17a	For each primary and secondary outcome, results for each group, and the estimated effect size and its precision (such as 95% confidence interval)	10-14, and tables
	17b	For binary outcomes, presentation of both absolute and relative effect sizes is recommended	NA
Ancillary analyses	18	Results of any other analyses performed, including subgroup analyses and adjusted analyses, distinguishing pre-specified from exploratory	Additional file 2
Harms	19	All important harms or unintended effects in each group (for specific guidance see CONSORT for harms)	Ethics application
**Discussion**			
Limitations	20	Trial limitations, addressing sources of potential bias, imprecision, and, if relevant, multiplicity of analyses	14-15
Generalisability	21	Generalisability (external validity, applicability) of the trial findings	15-16
Interpretation	22	Interpretation consistent with results, balancing benefits and harms, and considering other relevant evidence	14-17
**Other information**			

Registration	23	Registration number and name of trial registry	5
Protocol	24	Where the full trial protocol can be accessed, if available	In the registry
Funding	25	Sources of funding and other support (such as supply of drugs), role of funders	18

*We strongly recommend reading this statement in conjunction with the CONSORT 2010 Explanation and Elaboration for important clarifications on all the items. If relevant, we also recommend reading CONSORT extensions for cluster randomised trials, non-inferiority and equivalence trials, non-pharmacological treatments, herbal interventions, and pragmatic trials. Additional extensions are forthcoming: for those and for up to date references relevant to this checklist, see www.consort-statement.org.

Abukmail E, *et al. BMJ Open* 2023; 13:e067624. doi: 10.1136/bmjopen-2022-067624

Dynata (demographics in Australia, recruitment strategies)

(Screenshots were taken from the information documents Dynata shared with the researchers at the start of the study and gave permission to share)

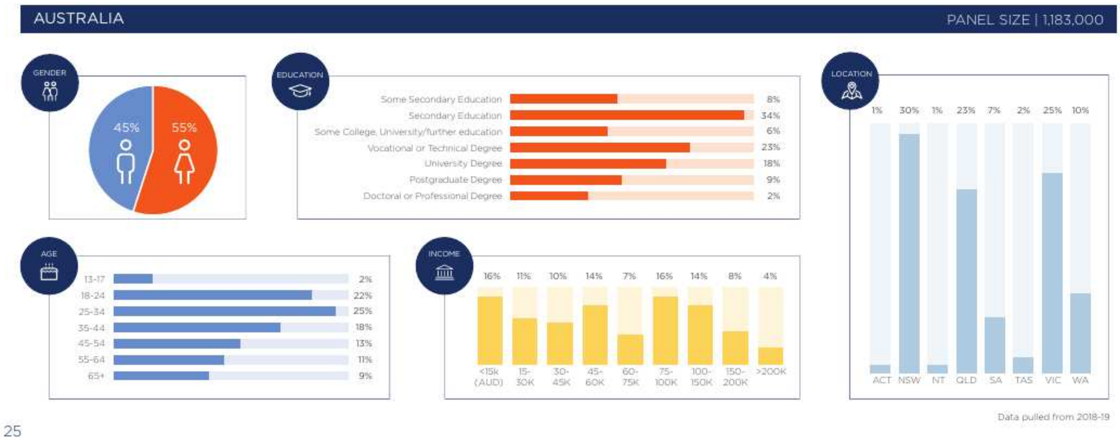

Recruitment Methodologies

SOURCE NAME	RECRUITMENT
Loyalty Panels	By invitation to 1000's of loyalty program members in travel, entertainment, media and retail.
Organic, Open Enrolment & Partnerships	1000's of websites, social media influencers and mobile apps.
Affiliate Network	Broad range of websites, incl. schools, communities. Member logging into communities with valid username and password invited to participate.

Abukmail E, *et al. BMJ Open* 2023; 13:e067624. doi: 10.1136/bmjopen-2022-067624

Interventions (visual presentations)

Trial A (Acute otitis media)

Pictograph

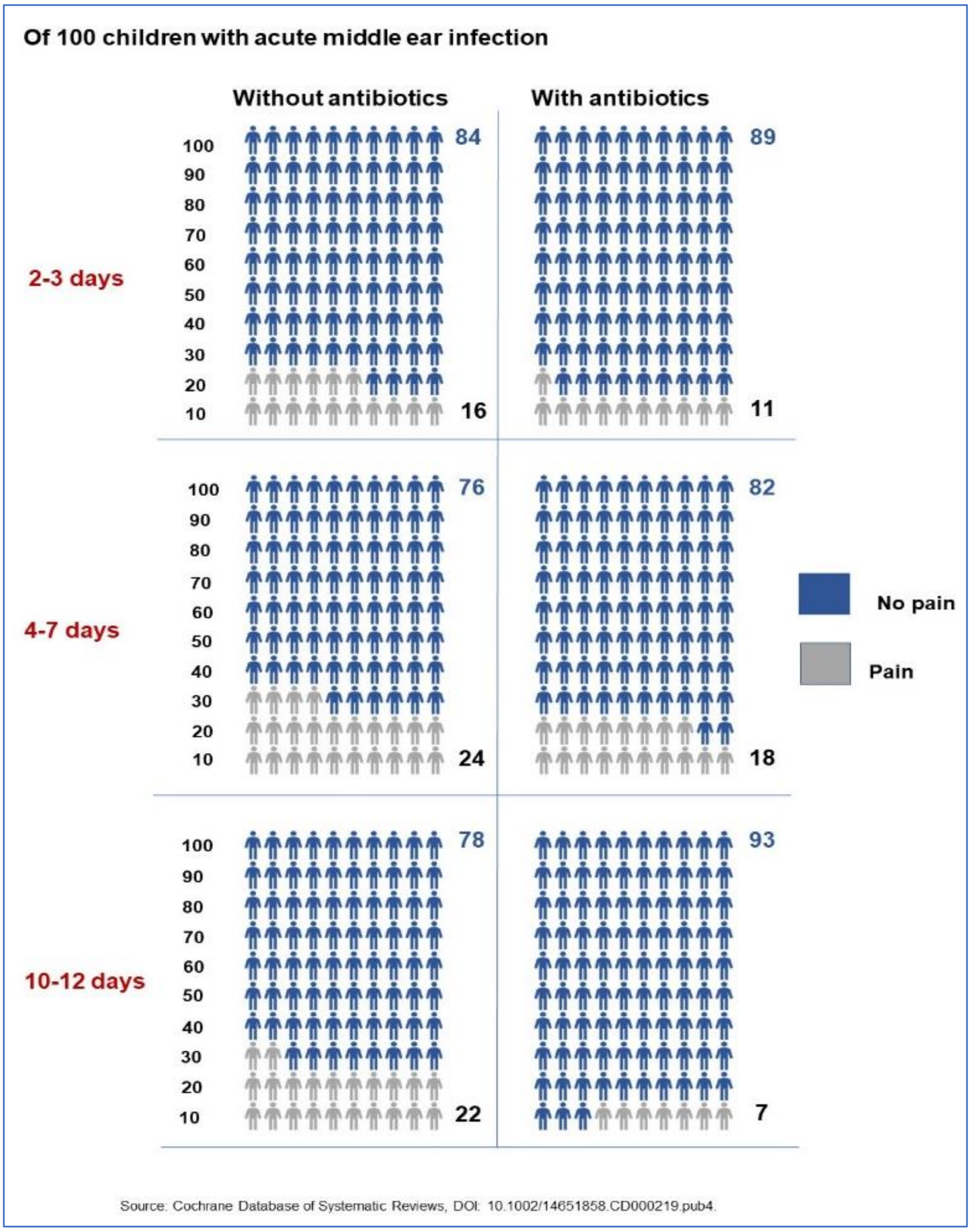

Bar graph

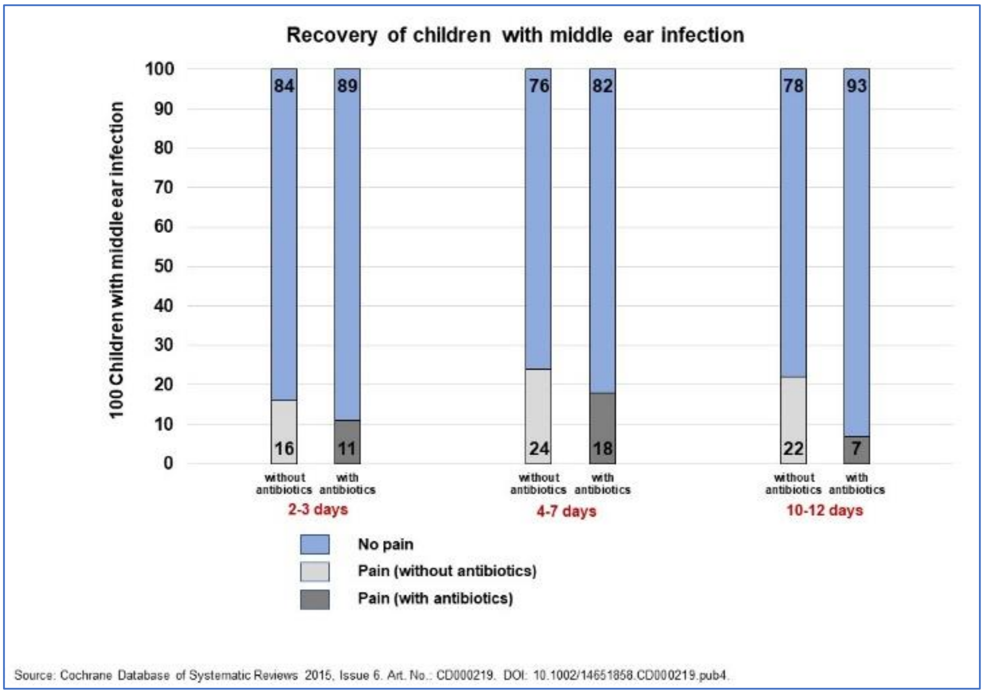

Text-only

Recovery of children with middle ear infection

Of 100 children who have middle ear infection:

Without antibiotic treatment

16 children will still experience pain at **2-3 days** after diagnosis. 24 children will have pain at **4-7 days** and 22 children will have pain at **10-12 days**. The others will have no pain.

With antibiotic treatment

11 children will still experience pain at **2-3 days** after diagnosis, 18 children will have pain at **4-7 days** and 7 children will have pain at **10-12 days**. The others will have no pain.

Source: Cochrane Database of Systematic Reviews 2015, Issue 6. Art. No.: CD000219. DOI: 10.1002/14651858.CD000219.pub4.

Line graph

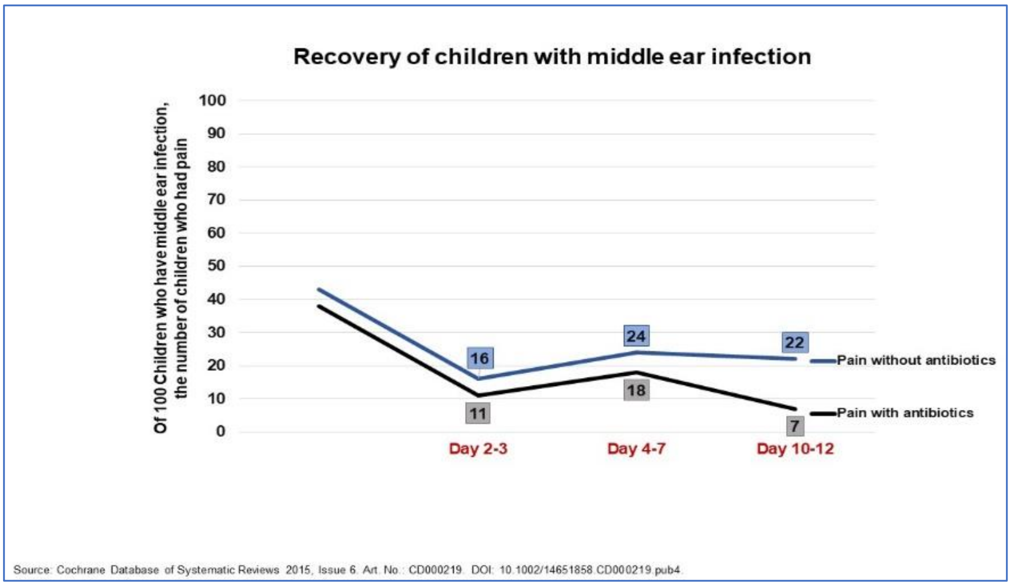
Recovery of children with middle ear infection
Of 100 Children who have middle ear infection, the number of children who had pain
100
90
80
70
60
50
40
30
20
10
0
16
24
22
11
18
7
Day 2-3
Day 4-7
Day 10-12
Pain without antibiotics
Pain with antibiotics
Source: Cochrane Database of Systematic Reviews 2015, Issue 6. Art. No.: CD000219. DOI: 10.1002/14651858.CD000219.pub4.

Trial B (Lateral epicondylitis)

Pictograph

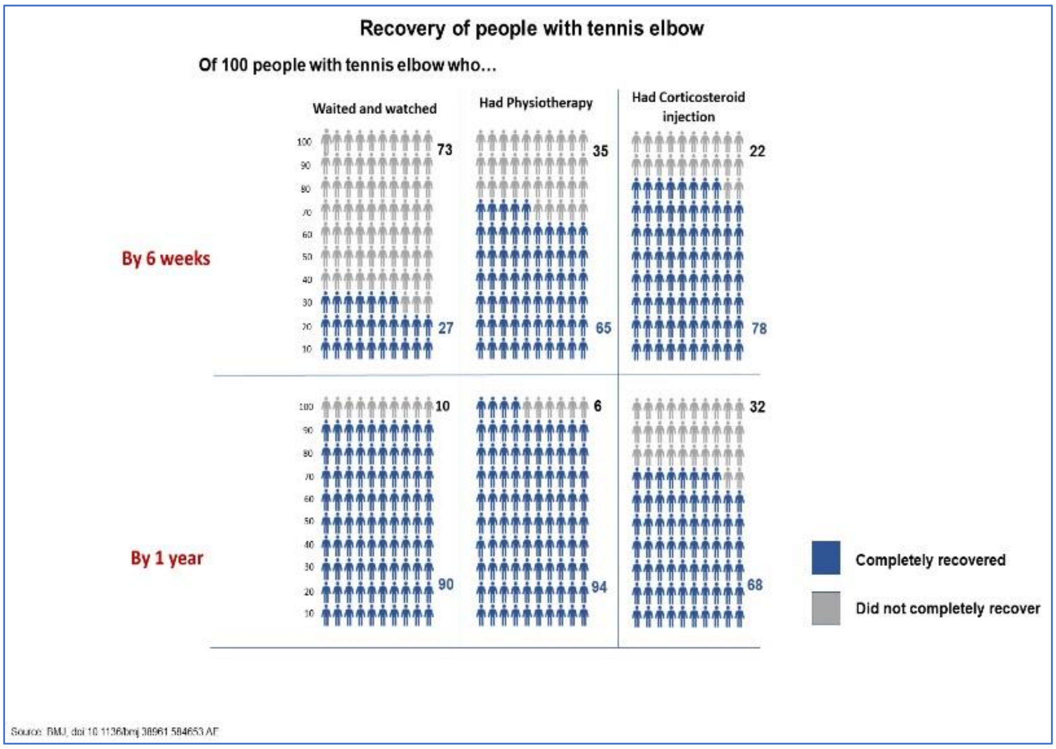

Text-only

Recovery of people with tennis elbow

Of 100 people with tennis elbow who:

Waited and watched (to see if it gets better on its own)

27 people completely recovered **by 6 weeks**. **By 1 year**, 90 people completely recovered; the other 10 did not completely recover.

Had physiotherapy

65 people completely recovered **by 6 weeks**. **By 1 year**, 94 people completely recovered; the other 6 did not completely recover.

Had corticosteroid injection

78 people completely recovered **by 6 weeks**. **By 1 year**, 68 people completely recovered; the other 32 did not completely recover.

Source: BMJ, doi:10.1136/bmj.38961.584653.AE

Bar graph

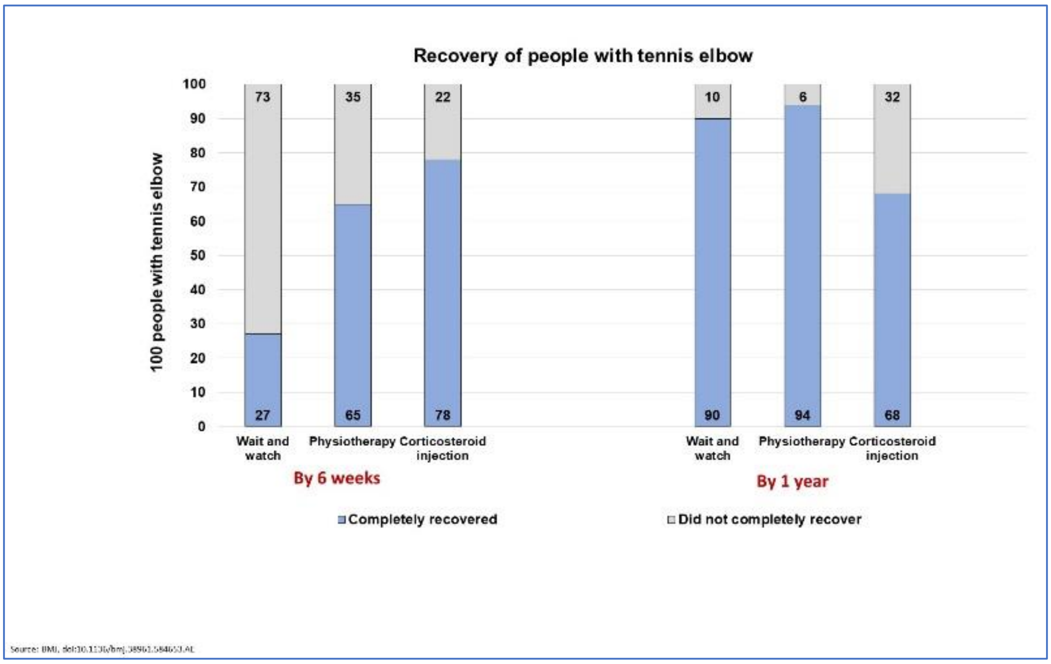

Line graph

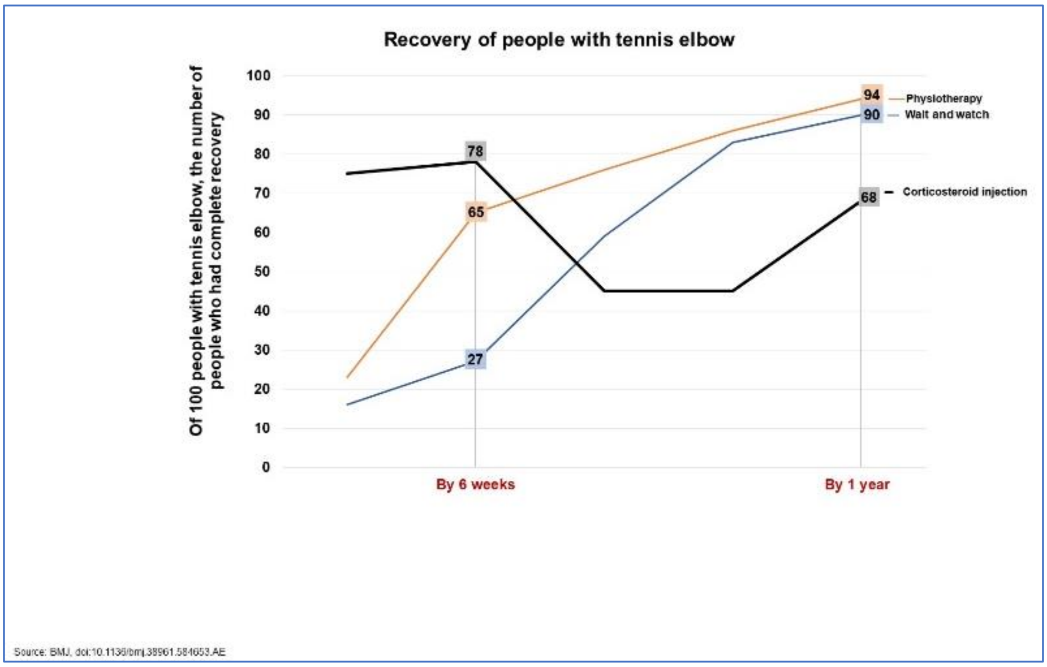

The flow of the trials and outcome measurements

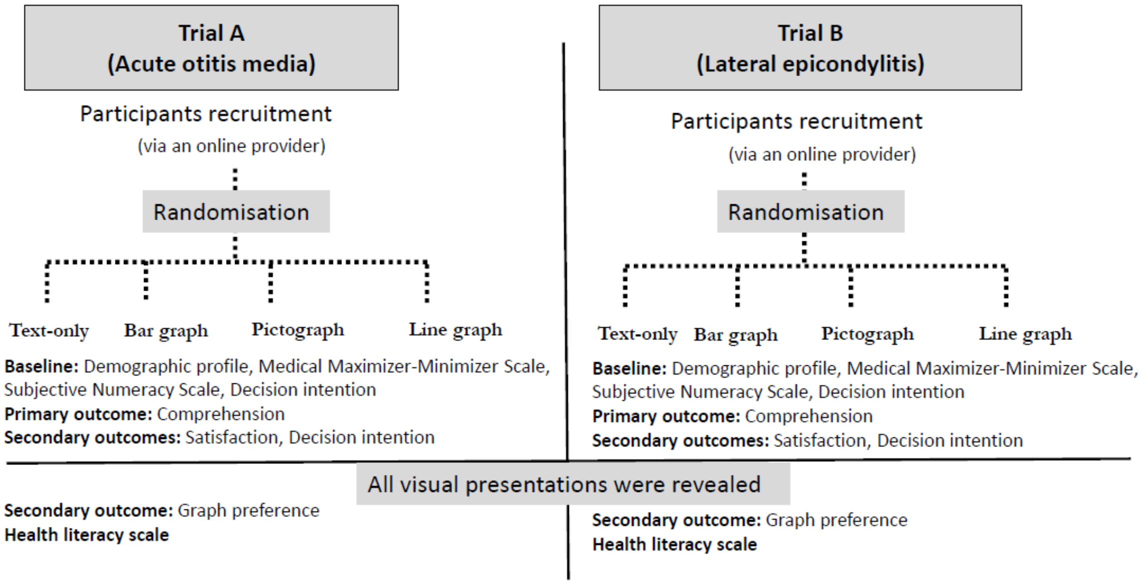

Survey

Questions 1-6 are the same for both trials

1. Which age group applies to you?

[ ] Under 18 [ ] 18 to 24 years [ ] 25 to 34 years
[ ] 35 to 44 years [ ] 45 to 54 years [ ] 55 to 64 years
[ ] 65 years or older [ ] Prefer not to say

2. What is your gender?

[ ] Male [ ] Female
[ ] Other [ ] Prefer not to say

3. What is your level of education? Please indicate the highest level of education you have completed.

[ ] High school - Year 10
[ ] High school – Year 12
[ ] Certificate I-IV, Diploma, or apprenticeship
[ ] Undergraduate degree: Bachelor's degree, or equivalent
[ ] Postgraduate degree: Masters or Doctoral degree, or equivalent

4. Which state or territory do you live in? [PUNCH STATE FROM POSTCODE- OKAY TO MOVE THIS AFTER Q5 IN SCRIPT- DO NOT ASK RESPONDENTS]

[ ] QLD [ ] NSW [ ] ACT [ ] NT [ ] WA [ ] VIC
[ ] TAS [ ] SA

5. What is your postcode? ___________

6. Is English the main language that you speak at home?

[ ] Yes [ ] No

Questions 7 and 8 are specific for each trial

Trial A (Acute otitis media)

7. Acute middle ear infection is an infection behind the eardrum. It is very common, usually in children, and can be very painful. You can take any pain medicine with any of the options below. Imagine that a child you take care of gets a middle ear infection, what decision about using antibiotics might you make?

[ ] The child usually should take antibiotics.
[ ] The child usually should not take antibiotics.
[ ] Unsure

8. Have you, or a person that you are responsible for, previously had an acute middle ear infection?

[ ] Yes
[ ] No

Trial B (lateral epicondylitis)

7. Tennis elbow is a condition in which people have pain and tenderness over the outside of the elbow It usually gets better by itself, regardless of the type of treatment, but symptoms may last up to 2 years[1].

[1] Source: Olaussen M, Holmedal O, Lindbaek M, et al Treating lateral epicondylitis with corticosteroid injections or non -electrotherapeutical physiotherapy: a systematic review BMJ Open 2013;3:e003564. doi: 10.1136/bmjopen-2013-003564

You can take any pain medicine with any of the options below. Imagine that you have tennis elbow, what decision about other treatment options, from the below list, might you make?

[ ] I would probably have a corticosteroid injection.
[ ] I would probably have physiotherapy.
[ ] I would probably wait and watch to see if it gets better on its own.
[ ] Unsure

8. Have you, or a person that you know, previously had tennis elbow (lateral epicondylitis)?

[ ] Yes [ ] No

Questions 9 -11 are the same for both trials (Q9 is MMM scale, Q10 and 11 are Subjective Numeracy Scale)

Comprehension questions Q12-Q17 (These questions are different for trial A and trial B)

Trial A (Acute otitis media) only

Middle ear infection is one of the most frequent diseases in childhood. Children usually experience symptoms of middle ear inflammation like pain, ear discharge, and fever.[2]

The numbers below show what happens over time to children up to 15 years of age with middle ear infection when different ways of managing it are chosen.

The intervention was inserted here

Based on the information in the XXXX (please insert the type of graph e.g., pictograph), please answer the following questions. (Used for all graph interventions)

Based on the information in the text box, please answer the following questions. (Used for all text interventions):

(Q12-Q17- ON SAME PAGE ALONG WITH THE INTERVENTION).

12. At 2-3 days, out of 100 children who did NOT take antibiotics, how many had pain?

Answer: ___________

13. At 10-12 days, out of 100 children who took antibiotics, how many had pain?

Answer: ___________

14. Which group was less likely to experience pain at about 4-7 days?

[ ] Children who took antibiotics
[ ] Children who did not take antibiotics.
[ ] No difference between the groups.
[ ] I do not know.

15. At 4-7 days, how many more children who did NOT take antibiotics will have pain, compared to those who took antibiotics?

Answer: ___________

[2] Source: Cochrane Database of Systematic Reviews 2015, Issue 6. Art. No.: CD000219. DOI: 10.1002/14651858.CD000219.pub4.

16. At 2-3 days, how many more/fewer children who did NOT take antibiotics will be pain-free, compared to children who did take antibiotics?

[ ] 5 fewer [ ] 5 more [ ] 16 more [ ] 11 fewer

17. In the week or so after a middle ear infection starts, the majority of children will NOT be in pain, regardless of whether they do or do not take antibiotics

[ ] True [ ] False

Trial B (Lateral epicondylitis) only

Tennis elbow is a condition in which people have pain and tenderness over the outside of the elbow. This can make some movements of the arm and wrist painful.

The numbers below show what happens over time to people with tennis elbow when different ways of managing it are chosen.

The intervention was inserted here

Based on the information in the XXXX (please insert the type of graph e.g., pictograph), please answer the following questions. (Used for all graph interventions)

Based on the information in the text box, please answer the following questions. (Used for all text interventions):

(Q12-Q17- ON SAME PAGE ALONG WITH THE INTERVENTION)

12. Of 100 people with tennis elbow who chose to 'wait and watch, how many people completely recovered by 6 weeks?

Answer: ___________

13. Of 100 people with tennis elbow who chose to have a corticosteroid injection, how many people completely recovered by 6 weeks?

Answer: ___________

14. How many <u>more</u> people who chose physiotherapy completely recovered by 6 weeks, compared to those who waited and watched?

Answer: ___________

15. Which group was <u>more likely</u> to be completely recovered by 1 year?

[ ] People who waited and watched
[ ] People who had physiotherapy
[ ] People who had a corticosteroid injection
[ ] No difference between the groups
[ ] I do not know

16. **By 1 year, how many more/ fewer people who had physiotherapy completely recovered, compared to people who had a corticosteroid injection?**

[ ] 26 fewer [ ] 26 more [ ] 32 more [ ] 6 fewer

17. **By 1 year, the majority of people who chose to wait and watch will be completely recovered, without having physiotherapy or a corticosteroid injection.**

[ ] True [ ] False

Question 18 is trial specific

Trial A

18. Imagine again that a child you take care of gets a middle ear infection. What decision about using antibiotics might you make?

[ ] The child usually should ***take*** antibiotics.

[ ] The child usually should ***not take*** antibiotics.

[ ] Unsure

Trial B

18. Imagine again that you have tennis elbow. What decision about the management option might you make?

[ ] I would probably have a corticosteroid injection.

[ ] I would probably have physiotherapy.

[ ] I would probably wait and watch to see if it gets better on its own.

[ ] Unsure

Questions 19-23 are the same for both trials

19. **Can you please indicate the most important reason for your decision?**

Questions 20-28 are the same for both trials

Trial A statement: You have just seen A XXXX-(PIPE IN TEXT AS PER GRAPH SHOWN pictograph bar graph, line graph, text) presenting information about the recovery of children with middle ear infection

Trial B statement: You have just seen a XXXX (PIPE IN TEXT AS PER GRAPH SHOWN pictograph bar graph, line graph, text) presenting information about the recovery of people with tennis elbow over time.

The allocated intervention was shown here again

Now we would like to ask you a few questions about ***how the information*** is presented.

20. In this presentation, how ***easy*** was it to understand the information presented? (From 1= Not at all easy to 10= extremely easy)
21. How ***satisfied*** are you with **HOW** the information is presented? (From 1= not at all satisfied to 10=I am totally satisfied)
22. **In the future, you may have to make a decision about managing a health condition. To help you make this decision you may like more information about how your health may change over time because of this condition (i.e. its prognosis). You have just seen A (PIPE IN TEXT AS PER GRAPH SHOWN pictograph bar graph, line graph, text) presenting prognostic information.**

We will now show other ways to you and ask you to rank these ways from the MOST preferred option (1) to the LEAST preferred option (4) for receiving this type of information in future. [SHOW ALL INTERVENTIONS ALONG WITH LABELS]

[ ] Text only
[ ] A bar graph
[ ] A pictograph
[ ] A line graph

23. In a few words, please tell us why you chose the option that you ranked the highest (1)?

24. Do you prefer another way of presenting prognostic information THAT IS NOT IN THE PREVIOUS LIST (e.g., a combination of text and a graph, a table, a combination of a table and a graph)?

Please note that these are just examples and please feel free to suggest any other ways you may prefer.

25. Please rate how much experience you have had with each of the following graphs before today: (Rating from 1= no experience to 5 = a lot of experience) (graphs were shown)

Questions 26 and 27 are the NVS health literacy scale

28. If you had any difficulties with the survey (reading, understanding questions, technical problems) or you have any comments to improve the survey, please note them here

Thank you for your time and for taking part in our survey

Abukmail E, *et al. BMJ Open* 2023; 13:e067624. doi: 10.1136/bmjopen-2022-067624
