# Supplementary material for: Effect of different visual presentations on the public’s comprehension of prognostic information using acute and chronic condition scenarios: two online randomised controlled trials
Source: BMJ Open. 2023 Jun 14;13(6):e067624. doi: 10.1136/bmjopen-2022-067624 (PMC10277048; doi:10.1136/bmjopen-2022-067624)
Supplement: Supplementary data [file bmjopen-2022-067624supp002.pdf]

Additional file 2

(Supplementary results)

Comprehension results 2

Equivalence comparisons of graph types 3

Association of comprehension with HL, numeracy, and education level

Trial A (Acute otitis media) 4

Trial B (Lateral epicondylitis) 4

Change of decision intention by intervention

Trial A (Acute otitis media) 5

Trial B (Lateral epicondylitis) 7

Reasons for decision intention Q19

Trial A (Acute otitis media) 9

Trial B (Lateral epicondylitis) 12

Reasons for first choice preference Q23

Trial A (Acute otitis media) 16

Trial B (Lateral epicondylitis) 18

Exploratory results

Association of comprehension with MMM, time to complete comprehension question, and graph preference

Trial A (Acute otitis media) 20

Trial B (Lateral epicondylitis) 20

Visual presentation preference by previous graph experience

Trial A (Acute otitis media) 21

Trial B (Lateral epicondylitis) 21

Abukmail E, *et al. BMJ Open* 2023; 13:e067624. doi: 10.1136/bmjopen-2022-067624

Comprehension results

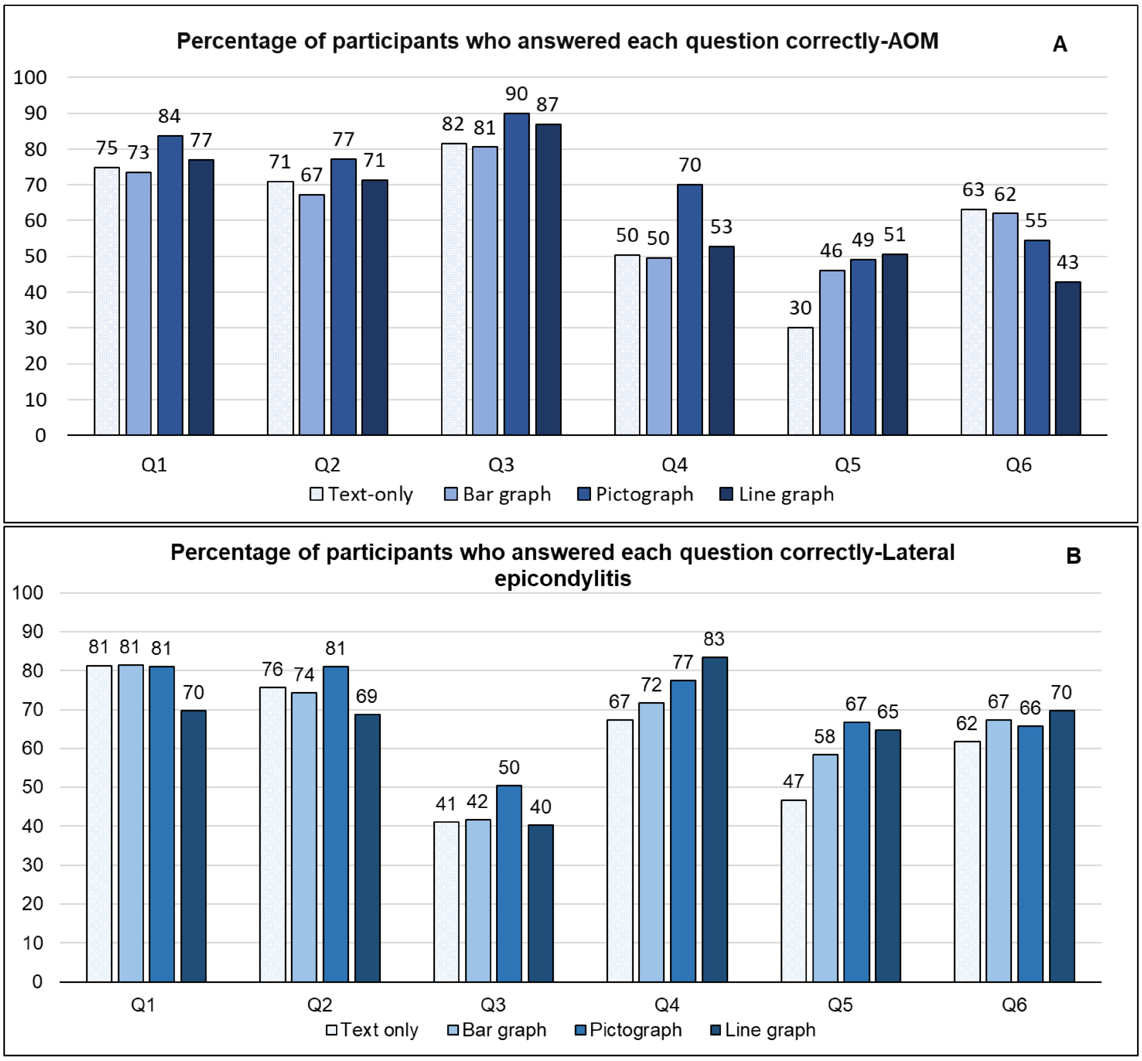

Comprehension of prognostic information. The numbers displayed represent the percentage of participants who answered each question correctly in (A) acute condition-AOM and (B) chronic condition-lateral epicondylitis. Q1-Q6 corresponds to Q12-Q17 in the survey (see additional file 1 for the questions).

Equivalence comparisons of graph types

AOM-Comprehension equivalence comparison between 3 intervention groups

Group 1	Group 2	Difference	95% CI
Pictograph	Bar graph	0.46	0.07 to 0.85
Pictograph	Line graph	0.43	0.03 to 0.84
Line graph	Bar graph	0.03	-0.40 to 0.45

Lateral epicondylitis- Comprehension equivalence comparison between 3 intervention groups

Group 1	Group 2	Difference	95% CI
Pictograph	Bar graph	0.28	-0.14 to 0.70
Pictograph	Line graph	0.26	-0.21 to 0.74
Line graph	Bar graph	0.01	-0.45 to 0.48

Abukmail E, *et al. BMJ Open* 2023; 13:e067624. doi: 10.1136/bmjopen-2022-067624

Association of comprehension with HL, numeracy, and education level

Trial A (Acute otitis media)

Variable	R2	Parameter Estimate	P-value	95% Confidence Limits	
Health literacy (categorised)	24%	High vs med 1.19	<.0001	0.86	1.51
		Low vs med -0.78	0.0007	-1.23	-0.33
Education	0%	0.05	0.47	-0.08	0.17
SNS	4%	0.33	<0.0001	0.19	0.48

Trial B (Lateral epicondylitis)

**Variable**	**R2**	**Parameter Estimate**	**P-value**	**95% Confidence Limits**	
**Health literacy (categorised)**	**29%**	**High vs med 1.28**	**<.0001**	**0.94**	**1.61**
		**Low vs med -1.29**	**<0.0001**	**-1.77**	**-0.81**
**Education**	**2%**	**0.21**	**0.0024**	**0.07**	**0.34**
**SNS**	**3%**	**0.3**	**0.0001**	**0.15**	**0.45**

Abukmail E, *et al. BMJ Open* 2023; 13:e067624. doi: 10.1136/bmjopen-2022-067624

Change of decision intention by intervention[1]

Trial A (Acute otitis media)

Acute condition - Acute otitis media (AOM) - Text only

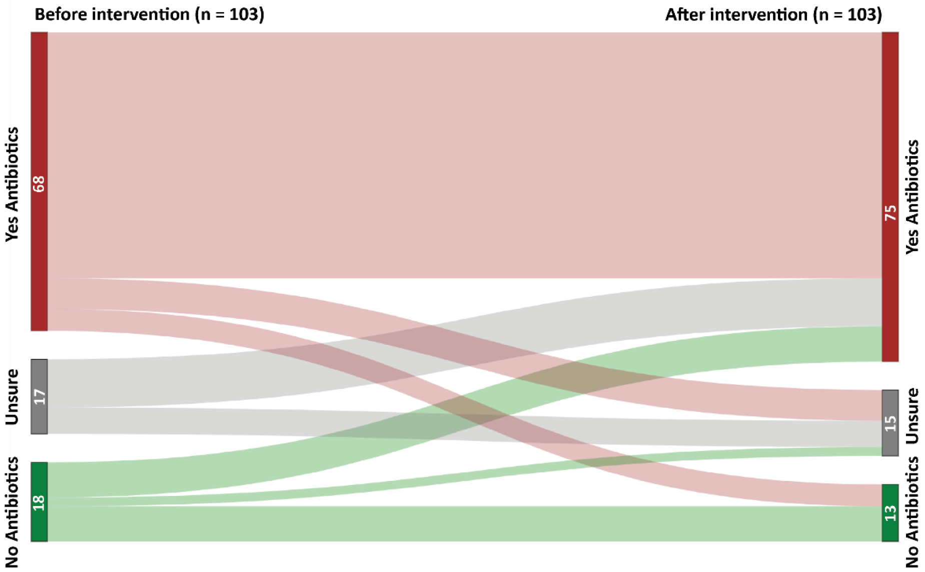

Acute condition - Acute otitis media (AOM) - Pictograph

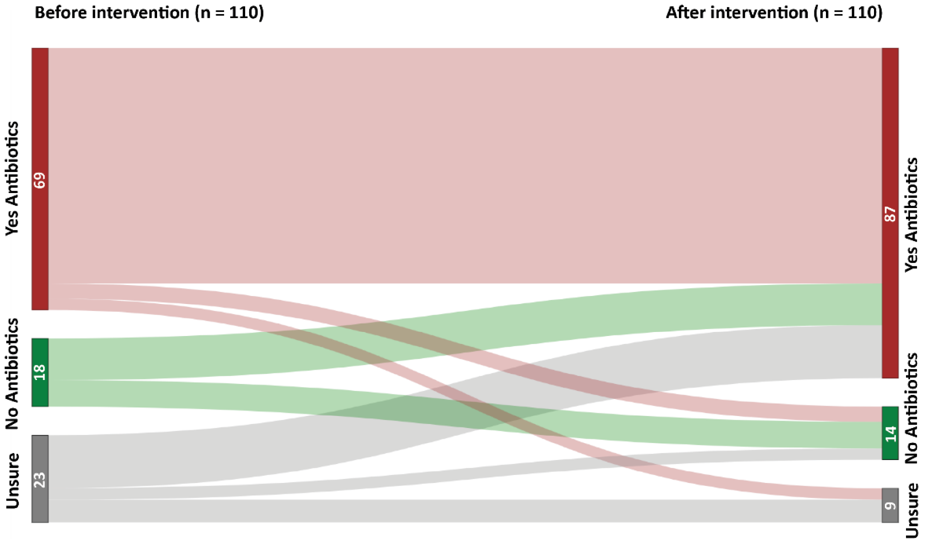

[1] Numbers and percentages of each choice before and after the interventions are presented in table 3 in the main article

Acute condition - Acute otitis media (AOM) - Line graph

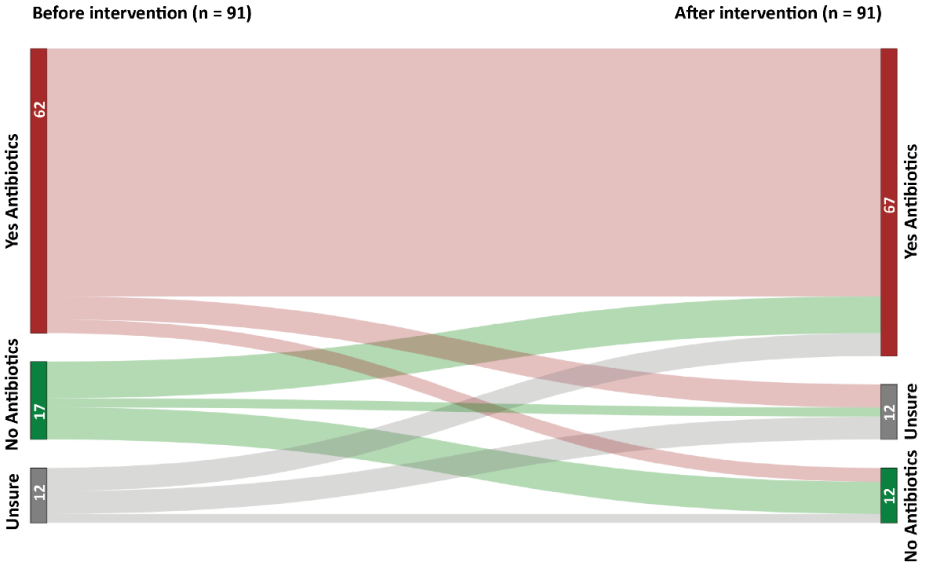

Acute condition - Acute otitis media (AOM) - Bar graph

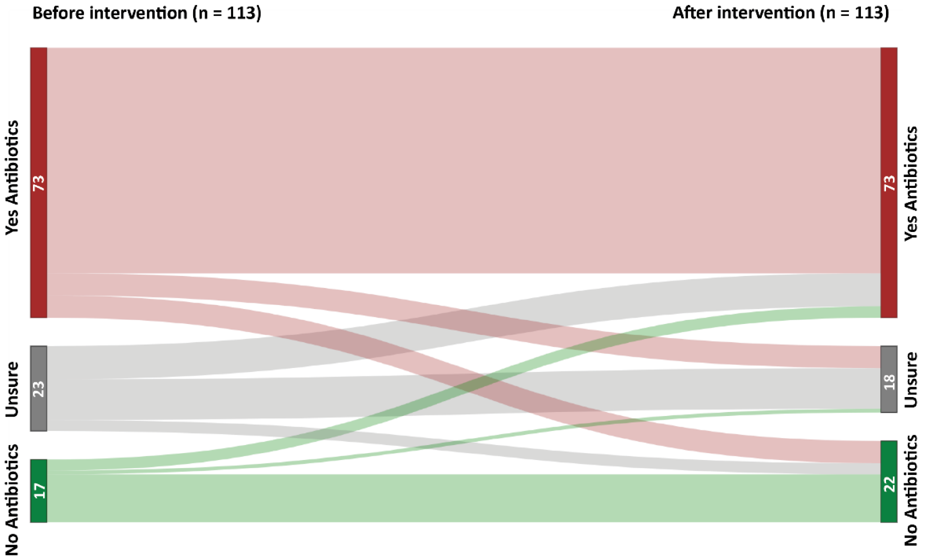

Abukmail E, *et al. BMJ Open* 2023; 13:e067624. doi: 10.1136/bmjopen-2022-067624

Trial B (Lateral epicondylitis)

Chronic condition - Lateral epicondylitis - Text only

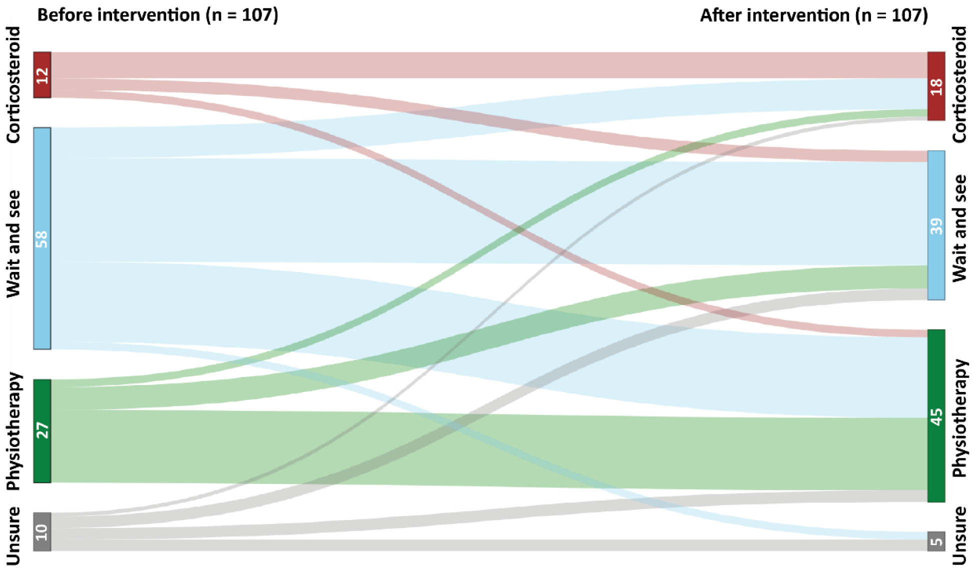

Chronic condition - Lateral epicondylitis - Pictograph

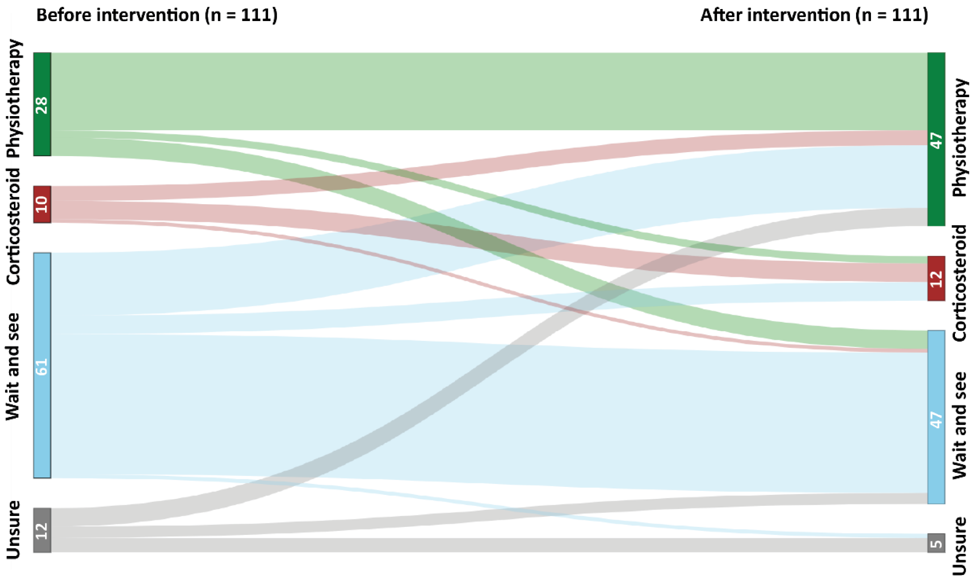

Chronic condition - Lateral epicondylitis - Bar graph

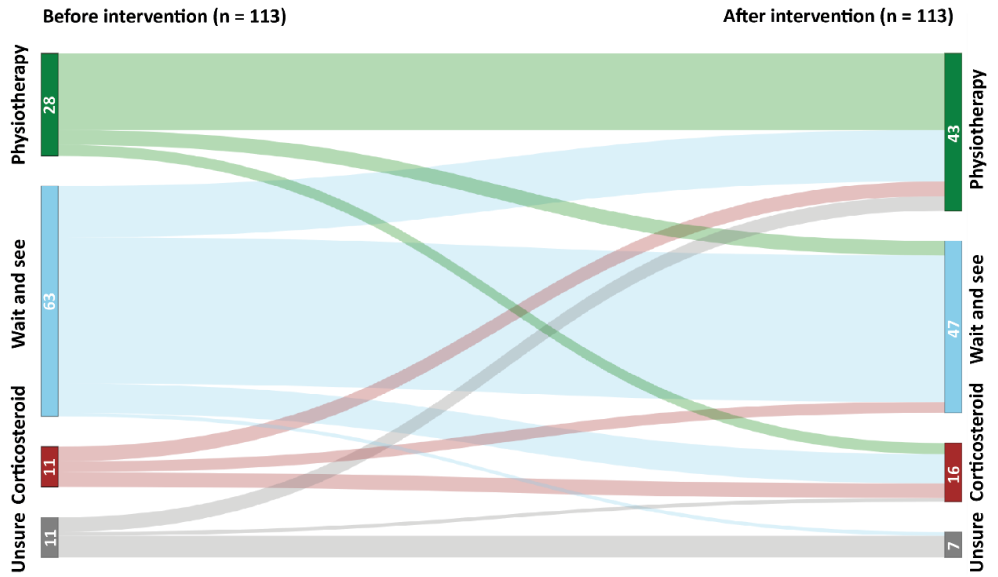

Chronic condition - Lateral epicondylitis - Line graph

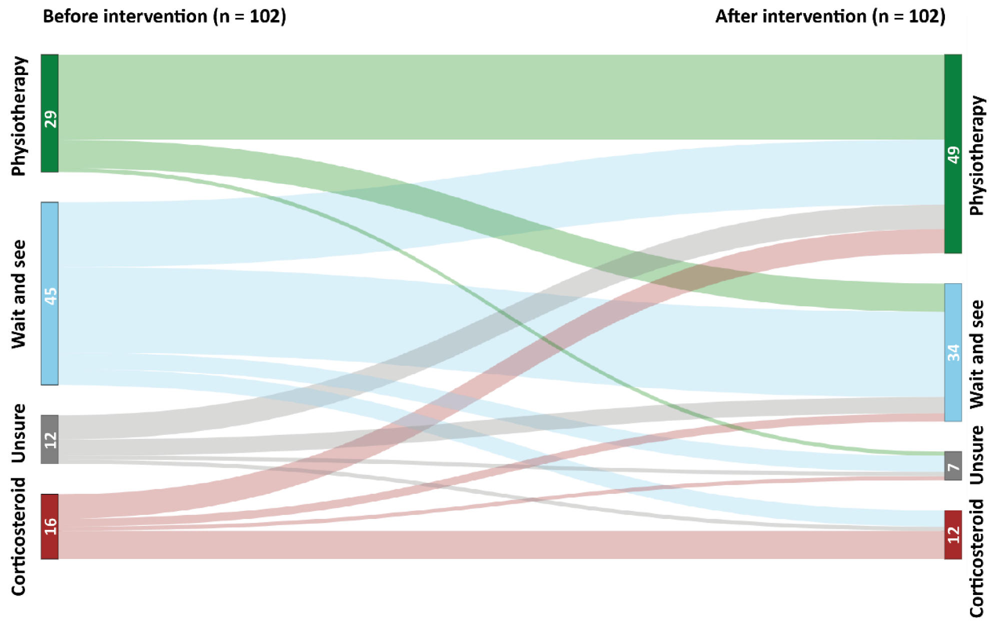

Reasons for decision intention Q19

Trial A (Acute otitis media)

Question 19: Can you please indicate the most important reason for your decision?					
**Reasons of participants who chose to give antibiotics**					
	**Category**	**Description**	**N total**	**Count/ intervention**	**Examples**
1	Based on the presented data	participants who thought data was favouring AB use or referred that their answers were based on the presented data	39	Text-only: 12<br>Bar graph: 9<br>Pictograph: 9<br>Line graph: 9	"the graph indicated that antibiotics has good effects"<br>"data proved fewer children in pain who took antibotics"
2	Antibiotics are effective	Decreased pain<br>Getting better<br>Reduce symptoms	145	Text-only: 33<br>Bar graph: 38<br>Pictograph: 43<br>Line graph: 31	"It helps relieve the pain for the child and fight the infection"<br>"Antibiotics will probably make slow down the infection and also pain."
3	Antibiotics save time	Decrease pain duration, quick recovery from illness, clear the infection faster	45	Text-only: 13<br>Bar graph: 11<br>Pictograph: 11<br>Line graph: 9	"quicker recovery"<br>"Because it helps it get better quickly"<br>"get rid of the pain faster"
4	Antibiotics prevent complications, concerns about complications if antibiotics were not used	Prevent superadded infections, prevent hearing loos, prevent eardrum perforation, prevent recurrence	50	Text-only: 12<br>Bar graph: 12<br>Pictograph: 17<br>Line graph: 9	"to prevent a perforated eardrum"<br>"it is important for your health and to avoid complications"<br>"The child would probably not be able to eliminate the infection themselves and this could possible lead to deafness or even death left untreated."<br>"To ensure that the infection doesnt get worse and permanently affect their hearing"
5	Following medical advice	The GPs decides if I should take antibiotics, or if necessary	14	Text-only: 3<br>Bar graph: 4<br>Pictograph: 4<br>Line graph: 3	"take it only if the doctor says"<br>"I rely on Medical Advice as to how to proceed in situations like this."
6	Based on personal experience or perspectives	Participants who had either negative/positive previous experience with the condition when they used/not antibiotics	21	Text-only: 7<br>Bar graph: 3<br>Pictograph: 4<br>Line graph: 6	"ive been a sufferer of inner ear infections so have my children antis are the only way for us"<br>"have had a child with ear infection, very painful, and always needed antibiotics"
7	Antibiotics are necessary/ needed	Participants who believed antibiotics are needed anyway, e.g., antibiotics work wonder	24	Text-only: 4<br>Bar graph: 5<br>Pictograph: 10<br>Line graph: 5	"you must take antibiotics to stop the infection"<br>"You have to treat an infection."<br>"pain is acute and the ear wont clear itself up"
8	Conditional use of antibiotics	Participants who think they would use antibiotics if pain was severe, or if there is fever, it took longer than expected, if my child is suffering	22	Text-only: 5<br>Bar graph: 5<br>Pictograph: 7<br>Line graph: 5	"Depends on the severity of the case."<br>"if he has fever"

**Reasons of participants who chose not to give antibiotics**					
	**Category**	**Description**	**N total**	**Count/ intervention**	**Example**
1	Based on the presented data	participants who thought that the presented data was not favouring antibiotic use	20	Text-only: 5<br>Bar graph: 8<br>Pictograph: 2<br>Line graph: 5	“previous chart showed only small difference”<br>“The statistics don’t support antibiotic use for this condition”
2	Based on their preference of other alternatives	Participants who preferred to use alternative options (other medicines, home remedies) or to wait and see if it goes on its own	13	Text-only: 0<br>Bar graph: 5<br>Pictograph: 4<br>Line graph: 4	“In many case home remedy will take care of this problem.”<br>“there may be other way to solve this problem”
3	Based on personal preference	Participants who didn’t give a specific reason e.g. I like this option	4	Text-only: 2<br>Pictograph: 1<br>Pictograph: 1<br>Line graph: 0	“i think its helpful”<br>“because I like it”
4	Concerns about antibiotic overuse and resistance	Participants who related their decision to the effect of overuse of antibiotics	11	Text-only: 0<br>Bar graph: 7<br>Pictograph: 4<br>Line graph: 0	“not worth becoming immune to antibiotics over a few percentage points.”<br>“Because of growing immunity to anibiotics it is always best to avoid them if they are not stricty necessary”
5	Concerns about adverse events	Participants who were worried about the side effects of antibiotics	18	Text-only: 1<br>Bar graph: 8<br>Pictograph: 5<br>Line graph: 4	“Because antibiotics has side effects and most children have self antibody with which he/she can immune himself/herself.”<br>“antibiotics can cause allergic reactions in kids”
6	Conditional use of antibiotics	Depends on severity of symptoms, or if prescribed by a GP	12	Text-only: 3<br>Bar graph: 4<br>Pictograph: 4<br>Line graph: 1	“Better not to take antibiotics unless it was really necessary”<br>“wait for doctor decision”
7	Antibiotics have little benefit/ not effective	Participants who thought antibiotics are not of great benefit in this case or in general	11	Text-only: 4<br>Bar graph: 3<br>Pictograph: 1<br>Line graph: 3	“Because generally middle ear infections are viral therefore do not need antibiotics”

**Reasons of participants who were unsure**					
	**Category**	**Description**	**N total**	**Count/ intervention**	**Example**
1	Based on the presented data	participants who can't understand the data presented/ need	8	Text-only: 1<br>Bar graph: 4<br>Pictograph: 2<br>Line graph: 1	"very little difference between taking and not taking drugs"<br>"did not understand"<br>"its hard to figure it"
2	Based on their preference of other alternatives	Participants who preferred to use alternative options (other medicines, home remedies), wait and see or having no treatment	6	Text-only: 1<br>Bar graph: 2<br>Pictograph: 2<br>Line graph: 1	"I think it would depend on how severe the infection is, and I would also look for a natural option opposed to anitbiotics"<br>"theres a fine line between the two,,, i prefer to wait a few days and see what eventuates,, not a big fan of antibiotics"
3	Lack of personal knowledge or experience	Participants who had no previous experience of the condition or knowledge about it	5	Text-only: 2<br>Bar graph: 2<br>Pictograph: 0<br>Line graph: 1	"None of my children have ever had a middle ear infection and they are all grown up now"<br>"I do not have children"
4	Concerns about side effects including resistance	Participants who were unsure because they were worried about side effect of using antibiotics	4	Text-only: 0<br>Bar graph: 0<br>Pictograph: 0<br>Line graph: 3	"medication causes the problem"<br>"It will depend on the child's other medical issues and the parents consent. Some children given antibiotics can have serious issues caused by them"
5	Conditional use, depends on severity, …etc	Participants who think using antibiotics depends on individual cases or on specific symptoms or causes	14	Text-only: 5<br>Bar graph: 4<br>Pictograph: 2<br>Line graph: 3	"depends on the history of the indvidual child"<br>"depends if the cause is bacterial"
6	Need more information	Participants who needed more information to make an informed decision	4	Text-only: 1<br>Bar graph: 1<br>Pictograph: 2<br>Line graph: 0	"i can not tell base on just one study"<br>"would need more details how long they have had it , if its reacurring etc"
7	Follow medical advice	Participants who would follow what the doctors say either to take antibiotics or not	9	Text-only: 3<br>Bar graph: 2<br>Pictograph: 0<br>Line graph: 4	"I prefer to consult a doctor first before answering the question."<br>"would consult a doctor"
8	Others	Participants who were unsure, don't know	11	Text-only: 3<br>Bar graph: 5<br>Pictograph: 1<br>Line graph: 2	"I don't know"<br>"Dont have any"

Trial B (Lateral epicondylitis)

Q19- Can you please indicate the most important reason for your decision?					
**Reasons of participants who chose corticosteroid injection**					
	**Category**	**Description**	**N Total**	**Count/ intervention**	**Examples**
1	Based on my understanding of the presented data	Participants who based their answers to their understanding of the presented data	24	Bar graph: 6<br>Pictograph: 5<br>Line graph: 3<br>Text only: 10	"The data shows that it has the best outcome of the three."<br>"Ease them immediate pain, best chance of recovery in the short term"
2	Based on previous experience	Based on the participants own experience with the treatment chosen or negative experience with the alternative treatments either personally or heard about it (e.g., someone they know)	12	Bar graph: 5<br>Pictograph: 3<br>Line graph: 3<br>Text only: 1	"because i have this condition and so far it works best for me"<br>"had previous cortizone injection"
3	Avoiding complications or side effects of alternative options/ against other options or personal preference	Participants who chose corticosteroid injection to avoid any complications that might happen if they choose the other options. either based on previous experience or expectation or as a personal preference	13	Bar graph: 2<br>Pictograph: 4<br>Line graph: 3<br>Text only: 4	"i dont believe in waiting to see if something get better or worse, i prefer this as i have had in the past"<br>"no physio here"
4	Corticosteroid offer a speedy recovery	Patients who chose steroid because it has better outcome on the short term	13	Bar graph: 4<br>Pictograph: 4<br>Line graph: 1<br>Text only: 4	"fast recovery chances"<br>"Time is not on my side, so I would take the speediect option"
5	This treatment is effective	Participants who thought this option would be effective	24	Bar graph: 7<br>Pictograph: 2<br>Line graph: 4<br>Text only: 11	"More people recover by using this method"<br>"to help cure the tennis elbow"
3 irrelevant responses were not categorized					

Reasons of participants who chose physiotherapy					
	**Category**	**Description**	**N Total**	**Count/intervention**	**Examples**
1	Based on my understanding of the presented data	Participants who based their answers to their understanding of the presented data	96	Bar graph: 20<br>Pictograph: 25<br>Line graph: 23<br>Text only: 28	"As the graphs show Physiotheraphy works"<br>"According to the graph patients getting physiotherapy recovers more quickly."
2	Based on previous experience and personal preference	Based on participants' own experience with the treatment chosen or negative experience with the alternative treatments either personally or heard about it (e.g., someone they know)	33	Bar graph: 10<br>Pictograph: 8<br>Line graph: 9<br>Text only: 6	"had a corticosteroid for my shoulder once willnever get one of those again it hurt all the time"<br>"I had it and it was working great"
3	Avoiding complications of the condition or side effects of alternative options/ against other options	Participants who chose corticosteroid injection to avoid any complications that might happen if they choose the other options either based on previous experience or expectation	13	Bar graph: 2<br>Pictograph: 2<br>Line graph: 3<br>Text only: 6	"drug free"<br>"I am doing something about it rather than waiting and watching and physiotherapy is more natural treatment than injecting myself"
4	A more natural option with less side effects	Participants who thought this option is safer and alleviate the side effects of other options	20	Bar graph: 3<br>Pictograph: 5<br>Line graph: 5<br>Text only: 7	"less invasive and high chance of recovery"<br>"It seems to be the best and least-invasive treatment option"
5	I would follow medical/GP advice	Participants who thought seeking medical advice is the right thing to do	6	Bar graph: 3<br>Pictograph: 0<br>Line graph: 3<br>Text only: 0	"get an expert to help with any pain or swelling"<br>"see a doctor sooner"
6	Physiotherapy is better on the long term	Participants who based their response mainly on the long-term recovery rate	12	Bar graph: 3<br>Pictograph: 2<br>Line graph: 2<br>Text only: 5	"Seems to be the best long term option"<br>"The long-term outcome"
7	Based on both short term and long-term effect	Participants who compared effectiveness both on the short term and long term	12	Bar graph: 1<br>Pictograph: 5<br>Line graph: 4<br>Text only: 2	"Best chance of short and long term recovery."<br>"Physiotherapy had a better long term result compared to the others which had a more of a short term solution"
8	Treatment is effective	Participants who thought this option is effective	94	Bar graph: 15<br>Pictograph: 28<br>Line graph: 27<br>Text only: 24	"I think it would help recover"<br>"Physio includes execises which help recovery and future protection"
9	Physiotherapy provides quicker recovery	Participants who believed physiotherapy will provide faster/ quicker recovery	33	Bar graph: 7<br>Pictograph: 9<br>Line graph: 6<br>Text only: 11	"To treat it quicker"<br>"to recover faster"

Abukmail E, et al. BMJ Open 2023; 13:e067624. doi: 10.1136/bmjopen-2022-067624

Reasons of participants who chose to wait and watch					
	**Category**	**Description**	**N Total**	**Count/intervention**	**Examples**
1	Based on my understanding of the presented data	Participants who based their answers to their understanding of the presented data	45	Text only: 9<br>Bar graph: 14<br>Pictograph: 15<br>Line graph:7	"Because the data shows that time will help heal the condition, and that although physiotherapy helped, doing nothing ultimately has the same outcome."
2	Based on previous experience	Based on the participants own experience with the treatment chosen or negative experience with the alternative treatments either personally or heard about it (e.g., someone they know)	15	Text only: 2<br>Bar graph: 6<br>Pictograph: 3<br>Line graph:4	"previous history and treatment"<br>"never had success with physio and other health issues make corticosteroid injection risky"
3	Avoiding complications of alternative options/ against other options	Participants who chose corticosteroid injection to avoid any complications that might happen if they choose the other options either based on previous experience or expectation	21	Text only: 3<br>Bar graph: 5<br>Pictograph: 8<br>Line graph:5	"I have tennis elbow now, I tend to let it run its course. I don't see the value in Physio vs time, and Cortisone damages the bone"<br>"i dont really like injections and physio is expensive"
4	A more natural option with less side effects	Participants who thought this option is safer and alleviate the side effects of other options	56	Text only: 16<br>Bar graph: 14<br>Pictograph: 14<br>Line graph:12	"safe option"<br>"Don't believe in using drugs if it can be avoided."
5	Long term benefit	Participants who based their response mainly on the long-term recovery rate	16	Text only: 3<br>Bar graph: 8<br>Pictograph: 3<br>Line graph:2	"The long term outcome isn't different enough to make me think that one course of action is much better than another"
6	Treatment is effective	Participants who thought this option is effective	38	Text only: 8<br>Bar graph: 11<br>Pictograph: 11<br>Line graph: 8	"effective"<br>"more chance of recovery"
7	Cost free	Participants who chose this option to avoid costs of treatment either because it is free, their insurance would not cover other treatment expenses or they wouldn't wish to spend money to achieve very minimal better chance recovery with the other options	35	Text only: 6<br>Bar graph: 11<br>Pictograph: 11<br>Line graph:7	"If I can save money not going to the doctor right away, then I'll do it."<br>"This is primarily from a cost perspective as I do not have the optimal health insurance that covers the cost of physiotherapy"

8	Depends on nature/ severity of the disease	Participants who chose this option as first option but are open to chose different options depending on the severity and time of the condition	17	Text only: 6<br>Bar graph: 5<br>Pictograph: 5<br>Line graph:1	"It would depend on the level of pain/discomfort and functional impairment, but I generally feel I could handle it at home with exercise and possibly analgesics if necessary"
9	Avoidance of medical advice	Either don't trust GPs/ medical advice	11	Text only: 2<br>Bar graph: 3<br>Pictograph: 4<br>Line graph:2	"I don't trust the medical profession that much, always been a wait and see person"<br>"i hate doctors"
10	compared both short term and long-term effects	Participants who compared effectiveness both on the short term and long term	4	Text only: 0<br>Bar graph: 2<br>Pictograph: 1<br>Line graph:1	"Short term you might get better results from the injection but in the long term you just need to let it work out"

**Reasons of participants who were unsure**					
	**Category**	**Description**	**N Total**	**Count/intervention**	**Example**
1	No reason, don't know, unsure	Participants who had no reason for their choice	11	Text only: 1<br>Bar graph: 4<br>Pictograph: 3<br>Line graph:3	"Unsure"<br>"no idea"
2	Conditional choice	Depending on severity, work commitments, cost	8	Text only: 2<br>Bar graph: 3<br>Pictograph: 1<br>Line graph:2	"depen on how much it's pain"<br>"it would depend on situation and if I was working and how much it impacted my life"
3	Previous experience/ lack of personal experience	Based on the participants own experience with the treatment chosen or negative experience with the alternative treatments either personally or heard about it (e.g., someone they know)	2	Text only: 0<br>Bar graph: 0<br>Pictograph: 1<br>Line graph:1	"because I'm unsure how I would react to find out that's what I have."<br>" Hard question... I have bilateral epicondylitis in both arms and have tried all of the above so I know nothing works lol Probably watch and wait"
4	Follow medical advice	Participants who thought seeking medical advice is the right thing to do	3	Text only: 2<br>Bar graph: 0<br>Pictograph: 0<br>Line graph:1	"I would see my doctor and be advised by his medical expertise which way to receive treatment"<br>"would probably consult with my doctor for the right treatment"

Reasons for first choice preference Q23

Trial A (Acute otitis media)

Q23. In a few words, please tell us why you chose the option that you ranked the highest (1)?		
	**Participants who chose text-only first**	
	**Explanation**	**Count**
0	No reason, Unsure, I don't know	6
1	It is easy to understand, clear, concise or it is just good	84
2	It is better than other presentations and easier to compare the different options in this presentation	25
3	It is my personal preference, or the presentation that I am more familiar with or appeals to me and my understanding the best	14
4	Less effort and time consuming to understand the information	3

	**Participants who chose bar graph first**	
	**Explanation**	**Count**
0	No reason, Unsure, I don't know	2
1	It is easy to understand, clear, concise or it is just good	71
2	It is better than other presentations and easier to compare the different options in this presentation	40
3	It is my personal preference, or the presentation that I am more familiar with or appeals to me and my understanding the best	9
4	Less effort and time consuming to understand the information	4
5	Visually better presented, I can understand the information at a glance. The colours are easy to distinguish	27
6	Better in showing the progress, prognosis, trend over time	0

	**Participants who chose pictograph first**	
	**Explanation**	**Count**
0	No reason, Unsure, I don't know	0
1	It is easy to understand, clear, concise or it is just good	32
2	It is better than other presentations and easier to compare the different options in this presentation	14
3	It is my personal preference, or the presentation that I am more familiar with or appeals to me and my understanding the best	1
4	Less effort and time consuming to understand the information	3
5	Visually better presented, I can understand the information at a glance. The colours are easy to distinguish	15
6	Better in showing the progress, prognosis, trend over time	0

Abukmail E, *et al. BMJ Open* 2023; 13:e067624. doi: 10.1136/bmjopen-2022-067624

	**Participants who chose line graph first**	
	**Explanation**	**Counts**
0	No reason, Unsure, I don't know	3
1	It is easy to understand, clear, concise or it is just good	54
2	It is better than other presentations and easier to compare the different options in this presentation	27
3	It is my personal preference, or the presentation that I am more familiar with or appeals to me and my understanding the best	1
4	Less effort and time consuming to understand the information	5
5	Visually better presented, I can understand the information at a glance. The colours are easy to distinguish	18
6	Better in showing the progress, prognosis, trend over time	11

Abukmail E, *et al. BMJ Open* 2023; 13:e067624. doi: 10.1136/bmjopen-2022-067624

Trial B (Lateral epicondylitis)

Q23. In a few words, please tell us why you chose the option that you ranked the highest (1)?		
	**Participants who chose text-only first**	
	**Explanation**	**Counts**
0	No reason, Unsure, I don't know	9
1	It is easy to understand, clear, concise or it is just good	67
2	It is better than other presentations and easier to compare the different options in this presentation	54
3	It is my personal preference, or the presentation that I am more familiar with or appeals to me and my understanding the best	16
4	Less effort and time consuming to understand the information	15

	**Participants who chose pictograph first**	
	**Explanation**	**Counts**
0	No reason, Unsure, I don't know	5
1	It is easy to understand, clear, concise or it is just good	25
2	It is better than other presentations and easier to compare the different options in this presentation	24
3	It is my personal preference, or the presentation that I am more familiar with or appeals to me and my understanding the best	3
4	Less effort and time consuming to understand the information	2
5	Visually better presented, I can understand the information at a glance. The colours are easy to distinguish	16
6	Better in showing the progress, prognosis, trend over time	0

	**Participants who chose bar graph first**	
	**Explanation**	**Counts**
0	No reason, Unsure, I don't know	3
1	It is easy to understand, clear, concise or it is just good	55
2	It is better than other presentations and easier to compare the different options in this presentation	68
3	It is my personal preference, or the presentation that I am more familiar with or appeals to me and my understanding the best	6
4	Less effort and time consuming to understand the information	8
5	Visually better presented, I can understand the information at a glance. The colours are easy to distinguish	28
6	Better in showing the progress, prognosis, trend over time	2

	**Participants who chose line graph first**	
	**Explanation**	**Counts**
0	No reason, Unsure, I don't know	1
1	It is easy to understand, clear, concise or it is just good	28
2	It is better than other presentations and easier to compare the different options in this presentation	26
3	It is my personal preference, or the presentation that I am more familiar with or appeals to me and my understanding the best	5
4	Less effort and time consuming to understand the information	4
5	Visually better presented, I can understand the information at a glance. The colours are easy to distinguish	2
6	Better in showing the progress, prognosis, trend over time	17

Exploratory results

Association between comprehension and MMM, time to complete comprehension questions, and graph preference

Trial A (Acute otitis media)

Variable	R2	Parameter Estimate	P-value	95% Confidence Limits	Variable
MMM scale (categorised)	2%	High vs med -0.60	0.01	-1.05	-0.14
		Low vs med -0.18	0.3	-0.52	0.16
Time to complete comprehension questions (mins)	2%	0.02	0.0066	0.01	0.04
Graph preference	3%	Bar vs text: 0.63	0.0008	0.27	1
		Picto vs text: 0.69	0.0045	0.21	1.16
		Line vs text: 0.52	0.011	0.12	0.92

Trial B (Lateral epicondylitis)

Variable	R2	Parameter Estimate	P-value	95% Confidence Limits	Variable
MMM scale (categorised)	3%	High vs med -0.81	0.0009	-1.28	-0.33
		Low vs med -0.07	0.71	-0.43	0.29
Time to complete comprehension questions (mins)	1%	0.03	0.053	0	0.05
Graph preference	4%	Bar vs text: 0.63	0.0011	0.25	1
		Picto vs text: 0.65	0.0077	0.17	1.12
		Line vs text: 0.84	0.0006	0.37	1.32

Abukmail E, *et al. BMJ Open* 2023; 13:e067624. doi: 10.1136/bmjopen-2022-067624

Overall Visual presentation preference by graph experience

Trial A (Acute otitis media)

Preference	N	Previous experience	N	Mean	Std Dev	Minimum	Maximum
Text only	124.0	Bar graph	124.0	2.9	1.2	1	5
		Pictograph	124.0	2.4	1.2	1	5
		Line graph	124.0	3.2	1.3	1	5
Bar graph	137.0	Bar graph	137.0	3.7	1.2	1	5
		Pictograph	137.0	2.5	1.2	1	5
		Line graph	137.0	3.4	1.2	1	5
Pictograph	58.0	Bar graph	58.0	3.7	1.0	1	5
		Pictograph	58.0	3.6	1.1	1	5
		Line graph	58.0	3.8	0.9	2	5
Line graph	98.0	Bar graph	98.0	3.2	1.2	1	5
		Pictograph	98.0	2.6	1.2	1	5
		Line graph	98.0	4.1	1.1	1	5

Trial B (Lateral epicondylitis)

Preference	N	Previous experience	N	Mean	Std Dev	Minimum	Maximum
Text only	144	Bar graph	144	2.92	1.18	1	5
		Pictograph	144	2.22	1.22	1	5
		Line graph	144	2.89	1.2	1	5
Bar graph	154	Bar graph	154	3.96	1.07	1	5
		Pictograph	154	2.48	1.19	1	5
		Line graph	154	3.51	1.12	1	5
Pictograph	68	Bar graph	68	3.71	1.12	1	5
		Pictograph	68	3.49	1.31	1	5
		Line graph	68	3.78	1.09	1	5
Line graph	67	Bar graph	67	3.64	1.05	1	5
		Pictograph	67	2.63	1.23	1	5
		Line graph	67	4.15	1.09	1	5

Abukmail E, *et al. BMJ Open* 2023; 13:e067624. doi: 10.1136/bmjopen-2022-067624
