# Supplementary material for: Effect of different visual presentations on the public’s comprehension of prognostic information using acute and chronic condition scenarios: two online randomised controlled trials
Source: BMJ Open. 2023 Jun 14;13(6):e067624. doi: 10.1136/bmjopen-2022-067624 (PMC10277048; doi:10.1136/bmjopen-2022-067624)
Supplement: Author's manuscript [file bmjopen-2022-067624.draft_revisions.pdf]

BMJ Open is committed to open peer review. As part of this commitment we make the peer review history of every article we publish publicly available.

When an article is published we post the peer reviewers' comments and the authors' responses online. We also post the versions of the paper that were used during peer review. These are the versions that the peer review comments apply to.

The versions of the paper that follow are the versions that were submitted during the peer review process. They are not the versions of record or the final published versions. They should not be cited or distributed as the published version of this manuscript.

BMJ Open is an open access journal and the full, final, typeset and author-corrected version of record of the manuscript is available on our site with no access controls, subscription charges or pay-per-view fees (http://bmjopen.bmj.com).

If you have any questions on BMJ Open's open peer review process please email info.bmjopen@bmj.com

BMJ Open

Effect on comprehension of different visual presentations to communicate prognosis: Two online randomised controlled trials in healthy adults

Journal:	*BMJ Open*
Manuscript ID	bmjopen-2022-067624
Article Type:	Original research
Date Submitted by the Author:	22-Aug-2022

Complete List of Authors:	Abukmail, Eman; Bond University Institute for Evidence-Based Healthcare, Faculty of Health Sciences and Medicine<br>Bakhit, Mina; Bond University, Centre for research in Evidence-Based Practice<br>Jones, Mark; Bond University<br>Del Mar, Chris; Bond University, Faculty of Health Sciences and Medicne<br>Hoffmann, Tammy; Bond University

Keywords:	GENERAL MEDICINE (see Internal Medicine), PRIMARY CARE, INTERNAL MEDICINE

SCHOLARONE™
Manuscripts

I, the Submitting Author has the right to grant and does grant on behalf of all authors of the Work (as defined in the below author licence), an exclusive licence and/or a non-exclusive licence for contributions from authors who are: i) UK Crown employees; ii) where BMJ has agreed a CC-BY licence shall apply, and/or iii) in accordance with the terms applicable for US Federal Government officers or employees acting as part of their official duties; on a worldwide, perpetual, irrevocable, royalty-free basis to BMJ Publishing Group Ltd ("BMJ") its licensees and where the relevant Journal is co-owned by BMJ to the co-owners of the Journal, to publish the Work in this journal and any other BMJ products and to exploit all rights, as set out in our licence.

The Submitting Author accepts and understands that any supply made under these terms is made by BMJ to the Submitting Author unless you are acting as an employee on behalf of your employer or a postgraduate student of an affiliated institution which is paying any applicable article publishing charge ("APC") for Open Access articles. Where the Submitting Author wishes to make the Work available on an Open Access basis (and intends to pay the relevant APC), the terms of reuse of such Open Access shall be governed by a Creative Commons licence – details of these licences and which Creative Commons licence will apply to this Work are set out in our licence referred to above.

Other than as permitted in any relevant BMJ Author's Self Archiving Policies, I confirm this Work has not been accepted for publication elsewhere, is not being considered for publication elsewhere and does not duplicate material already published. I confirm all authors consent to publication of this Work and authorise the granting of this licence.

For peer review only

Effect on comprehension of different visual presentations to communicate prognosis: Two online randomised controlled trials in healthy adults

Eman Abukmail, MD, M.Sc.[1], eabukmai@bond.edu.au

Mina Bakhit, MBBCh, PhD[1], mbakhit@bond.edu.au

Mark Jones, PhD[1], majones@bond.edu.au

Chris Del Mar, MD[1], FRACGP, cdelmar@bond.edu.au

Tammy Hoffmann, PhD[1], thoffman@bond.edu.au

[1]Institute for Evidence-Based Healthcare, Faculty of Health Sciences and Medicine, Bond University, Queensland, Australia.

Corresponding author:

Eman Abukmail, MD, M.Sc.

Institute for Evidence-Based Healthcare

Faculty of Health Sciences and Medicine

Bond University

14 University Dr, Robina

QLD, Australia 4229

Email: eabukmai@bond.edu.au

For peer review only - http://bmjopen.bmj.com/site/about/guidelines.xhtml

Manuscript word count: 3515

Abstract word count (without keywords): 299

Tables and Figures: 3 figures and 2 tables, 2 Additional files [Additional file 1 (Supplementary methods), Additional file 2 (Supplementary results)], copy of the registered protocol

References: 33

Keywords:

Health communication, prognosis communication, natural history communication, clinician-patient communication, communication in primary healthcare, visual presentations, formats

For peer review only

For peer review only - http://bmjopen.bmj.com/site/about/guidelines.xhtml

Abstract

Objectives

To assess the effectiveness of bar graph, pictograph, and line graph compared to text-only (superiority) and each other (equivalence) for communicating prognosis to the public.

Design Two online 4-arm parallel-group randomised controlled trials. Statistical significance was set at $p<0.016$ to allow for 3 primary comparisons.

Participants and setting Two separate Australian representative samples were recruited from a pool of members registered at Dynata online survey company. 481 participants were randomised to one of the four arms in trial A and 545 participants in trial B. 471 (98%) participants were included in the analysis of trial A while 433 (79%) were included in trial B.

Interventions In each trial 4 different visual presentations were tested: bar graph, pictograph, line graph and text-only. Trial A communicated prognostic information about an acute condition (acute otitis media-AOM) and trial B communicated the prognosis of a chronic condition (lateral epicondylitis). Both conditions are typically managed in primary where '**wait and see'** is a legitimate option.

Main outcome: Comprehension of prognostic information (score 0-6).

Secondary outcomes: Decision intention, satisfaction with the presentation, and visual presentation preferences.

Results In both trials, the mean comprehension score was 3.7 for the text-only group, with a pictograph superior to text-only in trial A (mean difference (MD) 0.54, 95% CI 0.13- 0.95, *p* 0.011) but not in trial B (MD 0.49, 95% CI 0.04-0.93, *p* 0.032).

For peer review only - http://bmjopen.bmj.com/site/about/guidelines.xhtml

Pairwise comparisons between the 4 presentation styles showed all were clinically equivalent (95% CIs between -1.0 and 1.0). In both trials, the bar graph was the most preferred presentation, chosen by 32.9% and 35.6% of participants in trial A and trial B respectively.

Conclusions

Any of the four visual presentations tested (text-only, bar graph, pictograph, line graph) may be suitable to use when discussing quantitative prognostic information with patients.

Trial registration (ACTRN12621001305819)

Keywords: prognosis communication, natural history communication, clinician-patient communication, visual presentations, graphs

For peer review only - http://bmjopen.bmj.com/site/about/guidelines.xhtml

For peer review only

Article Summary

Strengths and limitations

- This study is the first to focus on communicating prognostic information about conditions managed in primary care and for which "wait and see" is a legitimate option.
- The study recruited a wide range of participants to ensure inclusivity and diversity of gender, background, age and educational level.
- Baseline comprehension was not tested as it was not possible to ask specific comprehension questions without showing participants the interventions.
- Trial participants did not need prior or current experience with the condition to be eligible, which may have influenced responses to some questions.

For peer review only

Introduction

Providing patients with information on their health can encourage active participation in making decisions.[1] Having a conversation about prognosis can involve communicating quantitative data such as the likelihood of occurrence of an outcome, recurrence, or improvement over time, or the likely duration of the illness. Information about a condition's natural history (that is, the natural course of the condition without treatment) is also encompassed under the umbrella of prognostic information. Understanding prognostic information may help patients to have realistic expectations about the course of their condition and make well-informed decisions about subsequent steps.[2,3]

Communicating quantitative information can be challenging for both clinicians and patients[4] and most research has focused on how to communicate treatment benefits and harms. Several factors may contribute to patient's understanding of the information that is conveyed, including the format used and the framing of it.[5] We conducted a systematic review[6] to evaluate the visual presentations (e.g., a table, graph) that have been used to communicate prognostic information. The review identified only 11 studies and found no significant superior effect on comprehension for any of the visual presentations evaluated in the existing studies. It was unclear due to the lack of existing research if visual presentations would facilitate patients' understanding of the communicated information. Most studies presented prognostic information relevant to decision-making about undertaking screening or treatment for cancer.

The best ways of presenting prognostic information for conditions that are typically managed in primary care have rarely been explored. Some decisions in primary

For peer review only - http://bmjopen.bmj.com/site/about/guidelines.xhtml

care, for both acute and chronic conditions, may be enhanced by having prognostic information. Prognostic information is often presented verbally and whether the addition of visually presented information can facilitate its comprehension has not been studied. This study aimed to investigate, using conditions typically seen in primary care, whether graphs improve the comprehension of prognostic information compared to text-only, and if the type of graph matters. Within this study, we conducted two trials (A and B). In trial A, the scenario involved an acute condition (where a decision is needed within a few days), and trial B involved a chronic condition that requires a less urgent decision about how to manage it. Results from both trials informed the generalisability of our results for conditions managed in primary care.

Methods

This study was prospectively registered at the Australian New Zealand Clinical Trials Registry (ACTRN12621001305819, 27/09/2021). CONSORT and CHERRIES reporting statements were followed when applicable (See additional file 1).

Study design

The study consisted of two online 4-arm parallel-group randomised controlled trials. Both trials had the same design and differed only in the condition used; trial A conveyed information about an acute condition (acute otitis media-AOM) and trial B conveyed information about a chronic condition (lateral epicondylitis).

Participants and recruitment

Participants were recruited between the 1st and 12th of October 2021 through an online research company, Dynata (www.dynata.com). Dynata recruited a national representative sample (for age, gender, and education level) using an algorithm-

For peer review only - http://bmjopen.bmj.com/site/about/guidelines.xhtml

based sampling tool and sent the surveys to existing registered members who had previously consented to complete online surveys. At the survey commencement, participants read study information and a statement about their right to withdraw from it at any time. To ensure validity and unique responses, Dynata used a captcha at the start of each survey as well as an IP-digital stamp for each participant. Participants were compensated with 5.90 AUD by Dynata for survey completion. An invitation to the study was emailed to the participants individually using an automated router. Participants accessed the survey link via their Dynata dashboard. More information about Dynata Australian demographics, sampling, and recruitment is provided in Additional file 1.

Participants were eligible if they lived in Australia, are 18 years old or older, and could read and understand English. Participants did not have to have the condition presented in the scenario. Participants can only participate in either trial A or B.

Randomisation

In each trial, eligible participants were randomly allocated using a computer-generated sequence, generated by Dynata, to 1 of 4 groups (text-only, bar graph, pictograph, line graph).

Trial scenarios and intervention groups

Trial A: the scenario conveyed information about the prognosis of children with AOM. AOM was chosen as it is one of the most common childhood infections[7], with about 50% to 85% of all children experiencing at least one episode.[8] Data came from a Cochrane systematic review.[8]

Trial B: the scenario conveyed information about the recovery of people with lateral epicondylitis who had completely recovered or not at two time points when they

For peer review only - http://bmjopen.bmj.com/site/about/guidelines.xhtml

decided to watch and wait, have physiotherapy, or have a corticosteroid injection. We chose lateral epicondylitis as it is a chronic condition that has a few treatment options, including the option of waiting for the condition to spontaneously improve.[9]

Graphical details of bar graphs and pictographs (e.g., symmetric distribution in pictograph, adding a scale, shape of the icon) were chosen based on best practice principles identified from relevant literature.[10-27] The interventions used are provided in Additional file 1.

Patient and public involvement

We pilot tested the AOM materials in a convenience sample of day-care centre teachers (n= 8) and the lateral epicondylitis materials with university administration staff (n=9). The feedback helped to iteratively refine minor details of the interventions and the clarity of some survey questions.

Before commencing trial recruitment, online piloting of all trial materials was conducted with approximately 10% of the sample size needed for the full trial (trial A, n= 47; trial B, n= 41). Participants were recruited by Dynata and not invited to subsequently participate in the trials. Piloting data were also used to adjust the sample size calculation and were not included in the trial results.

Data collection and outcome measurement

Our primary outcome was comprehension of the information presented, measured using 6 questions based on previous studies.[12,16,23,27] (see Additional file 1 for questions). The comprehension questions were developed to assess different understanding skills, for example, the ability to extract numbers from the information provided (e.g., At 10-12 days, out of 100 children who took antibiotics, how many had pain?), the ability to use the numbers to compare the two options (e.g., Which

For peer review only - http://bmjopen.bmj.com/site/about/guidelines.xhtml

group was less likely to experience pain at about 4-7 days?), and the ability to make use of the information (e.g., In the week or so after a middle ear infection starts, the majority of children will NOT be in pain, regardless of whether they do or do not take antibiotics). Therefore, we were interested to assess overall comprehension differences across groups rather than differences in comprehension of individual questions. Secondary outcomes were decision intention, satisfaction with the presented format (before revealing all formats), and format preference (after revealing all formats). All outcomes were measured once (immediately post-intervention), except for decision intention which was measured pre-and post-intervention (See Additional file 2 for the results of individual questions).

Participants completed a 3-part, 28-question structured survey developed for the trial, based on surveys from relevant research. **Part 1**: five demographic questions, a baseline decision intention question, a question on previous experience with the condition, the medical maximizer-minimizer scale (MMS)[28], and a validated subjective numeracy scale (SNS)[29,30]. **Part 2:** each group was presented with information according to their allocated group (e.g., text-only, bar graph, pictograph, or line graph). This was followed by six comprehension questions, two questions about decision intention, and two about satisfaction with the intervention. **Part 3:** All four intervention presentations (text-only, bar graph, pictograph, and line graph) were revealed to participants and visual presentation preference was measured using three questions. Participants rated their previous graphic experience on a 1-5 scale. Health literacy level was measured using the Newest Vital Sign (NVS) scale.[31]

The multiple-choice options for the comprehension and decision intentions questions were randomised to minimise order bias. Participants were able to see the randomised intervention while answering the questions in Part 2, but once they

For peer review only - http://bmjopen.bmj.com/site/about/guidelines.xhtml

moved to the next page, they were unable to go back to change their responses. This was to ensure that responses to comprehension questions were not influenced by the other interventions that were revealed in Part 3.

Sample size

In the online piloting, the standard deviation (SD) for the primary outcome was, at most, 1.66 units. Assuming 90% power, 0.016% level of significance (adjusted to account for 3 primary comparisons), the difference between groups of 1 unit, and up to 20% missing data, 97 participants were calculated as required in each group (a total of 388 participants) in each trial.

Data analysis

The primary analysis tested whether comprehension was different between each graphic presentation and the text-only presentation (3 comparisons). Linear regression was used and mean differences with 95% confidence intervals (CI) and p-values calculated. Adjusted analysis was performed to account for any potential important baseline imbalances using linear regression, with adjustment for age group, education level, and health literacy level. Statistical significance was set at p<0.016 to allow for 3 primary comparisons.

To assess the equivalence of the three graphic presentations, 95% confidence intervals for the mean difference between groups were estimated for each of the three pairwise comparisons using linear regression. Equivalence was to be concluded if the 95% confidence intervals were between -1.0 and 1.0. Univariable linear regression models were constructed to estimate the association between comprehension as the dependent variable and the independent variables (including

For peer review only - http://bmjopen.bmj.com/site/about/guidelines.xhtml

health literacy, numeracy, and education level). The measure of association reported is the R^2 value. SAS® OnDemand for Academics 2014 was used to analyse the data. For the four open-ended questions (e.g., the most important reason for participant's decision to choose one of the management options), two authors (EA, MB) independently categorised the responses. Another author (TH) was consulted when there was disagreement. Each response could be categorised into one or more categories (to a maximum of three).

Post-protocol analysis and modifications from protocol

It was not feasible to analyse the data blinded to group allocation because we needed to identify the control group (text-only) so that the appropriate comparisons could be made.

We estimated the association between comprehension as the dependent variable and time to finish the comprehension questions and previous experience with graphs as independent variables. (Results are provided in the Additional file 2)

Results

Figure 1 shows the CONSORT flow diagram of participants in both trials. Table 1 presents the baseline demographic results. There were no baseline differences among the groups in either trial, except for health literacy which was slightly higher in the pictograph group in trial A.

Please insert figure 1 here

For peer review only - http://bmjopen.bmj.com/site/about/guidelines.xhtml

Table 1: Participants' characteristics

	Trial A				Trial B			
	**Text only (n= 103)**	**Bar graph (n= 113)**	**Pictograph (n= 110)**	**Line graph (n= 91)**	**Text only (n= 107)**	**Bar graph (n= 113)**	**Pictograph (n= 111)**	**Line graph (n= 102)**
**Age (years) n (%)**								
18-24	5 (4.9)	7 (6.2)	7 (6.4)	4 (4.4)	5 (4.7)	6 (5.3)	5 (4.5)	10 (9.8)
25-34	15 (14.6)	17 (15.0)	18 (16.4)	8 (8.8)	17 (15.9)	22 (19.5)	12 (10.8)	15 (14.7)
35-44	14 (13.6)	19 (16.8)	14 (12.7)	18 (19.8)	21 (19.6)	17 (15.0)	25 (22.5)	16 (15.7)
45-54	25 (24.3)	23 (20.4)	30 (27.3)	8 (8.8)	20 (18.7)	23 (20.4)	19 (17.1)	14 (13.7)
55-64	26 (25.2)	24 (21.2)	23 (20.9)	28 (30.8)	24 (22.4)	24 (21.2)	28 (25.2)	21 (20.6)
65+	18 (17.5)	23 (20.4)	18 (16.4)	25 (27.5)	20 (18.7)	21 (18.6)	21 (18.9)	26 (25.5)
Prefer not to say	0 (0.0)	0 (0.0)	0 (0.0)	0 (0.0)	0 (0.0)	0 (0.0)	1 (0.9)	0 (0.0)
**Gender n (%)**								
Male	48 (46.6)	53 (46.9)	49 (44.6)	47 (51.7)	52(48.6)	44 (38.9)	46 (41.4)	57 (55.9)
Female	55 (53.4)	60 (53.1)	60 (54.6)	44 (48.4)	55(51.4)	69 (61.1)	64 (57.7)	45 (44.1)
Others	0 (0.0)	0 (0.0)	1 (0.9)	0 (0.0)	0 (0.0)	0 (0.0)	1 (0.9)	0 (0.0)
**State n (%)**								
QLD	26 (25.2)	31 (27.4)	18 (16.4)	24 (26.4)	23 (21.5)	20 (17.7)	24 (21.6)	22 (21.6)
NSW	31 (30.1)	34 (30.1)	38 (34.6)	33 (36.3)	32 (29.9)	39 (34.5)	41 (36.9)	33 (32.4)
ACT	0 (0.0)	2 (1.8)	3 (2.7)	1 (1.1)	2 (1.9)	3 (2.7)	1 (0.9)	2 (2.0)
NT	1 (1.0)	0 (0.0)	0 (0.0)	0 (0.0)	1 (0.9)	0 (0.0)	1 (0.9)	1 (1.0)
WA	12 (11.7)	7 (6.2)	17 (15.5)	4 (4.4)	8 (7.5)	17 (15.0)	11 (9.9)	10 (9.8)
VIC	22 (21.4)	31 (27.4)	23 (20.9)	23 (25.3)	28 (26.2)	28 (24.8)	21 (18.9)	22 (21.6)
TAS	2 (1.9)	2 (1.8)	1 (1.0)	0 (0.0)	1 (0.9)	2 (1.8)	2 (1.8)	3 (2.9)
SA	9 (8.7)	6 (5.3)	10 (10.0)	6 (6.6)	12 (11.2)	4 (3.5)	10 (9.0)	9 (8.8)
**Educational level n (%)**								
High school Y10	13 (12.6)	19 (16.8)	12 (10.9)	10 (11.0)	12(11.2)	9(8.0)	11(9.9)	12(11.8)
High school Y12	11 (10.7)	20 (17.7)	25 (22.7)	16 (17.6)	14(13.1)	17(15.0)	19(17.1)	13(12.8)
Certificate I-IV, Diploma, or apprenticeship	31 (30.1)	32 (28.3)	32 (29.1)	25 (27.5)	39(36.5)	34(30.1)	35(31.5)	33(32.4)
Undergraduate degree[1]	32 (31.1)	29 (25.7)	28 (25.5)	28 (30.8)	29(27.1)	37(32.7)	33(29.7)	27(26.5)
Postgraduate degree[2]	16 (15.5)	13 (11.5)	13 (11.8)	12 (13.2)	13(12.2)	16(14.2)	13(11.7)	17(16.7)
**SNS Mean[3] (SD)**	4.2 (0.9)	4.2 (1.1)	4.2 (1.0)	4.2 (1.0)	4.1 (1.0)	4.2 (1.1)	4.2 (1.1)	4.2 (1.0)
SNS ability	4.3 (1.2)	4.3 (1.4)	4.3 (1.2)	4.3 (1.2)	4.1 (1.3)	4.1 (1.4)	4.1 (1.5)	4.3 (1.3)
SNS preferences	4.2 (0.8)	4.1 (1.1)	4.2 (0.9)	4.1 (1.0)	4.1 (0.9)	4.2 (1.0)	4.2 (1.1)	4.1 (1.0)
**Health literacy n (%)**								
4-6 adequate	60 (58.3)	77 (68.1)	86 (78.2)	59 (64.8)	68 (63.55)	76 (67.26)	80 (72.07)	67 (65.69)
2-3 possibly limited	38 (36.9)	25 (22.1)	19 (17.3)	24 (26.4)	24 (22.43)	23 (20.35)	16 (14.41)	26 (25.49)
0-1 highly limited	5 (4.9)	11 (9.7)	5 (4.6)	8 (8.8)	15 (14.02)	14 (12.39)	15 (13.51)	9 (8.82)
**MMM scale[4] n (%)**								
<4 Minimizer	54 (52.4)	65 (57.5)	61 (55.5)	52 (57.1)	63 (58.9)	65 (57.5)	62 (55.9)	47 (46.1)
=4 Neutral	31 (30.1)	32 (28.3)	31 (28.2)	21 (23.1)	31 (29.0)	27(23.9)	28 (25.2)	34 (33.3)
>4 Maximizer	18 (17.5)	16 (14.2)	18 (16.4)	18 (19.8)	13 (12.2)	21(18.6)	21 (18.9)	21 (20.6)
**Experienced with the condition n (%)**	47 (45.6)	48 (42.5)	50 (45.5)	46 (50.6)	32 (29.9)	46 (40.7)	39 (35.1)	26 (25.5)
**Time (mins) to finish comprehension Qs Median (IQR)**	3.5 (2.6-5.3)	3.5 (2.5-5.1)	3.5 (2.4-5.4)	3.3 (2.3-4.8)	3.4 (2.2-4.6)	3.2 (2.1-4.2)	3.2 (2.6-4.6)	3.1 (2.2-4.6)
**Speaking English at home n (%)**	98 (95.15)	105 (92.92)	101 (91.82)	86 (94.51)	100 (93.5)	101 (89.4)	103 (92.8)	94 (92.2)

[1] Bachelor's degree, or equivalent

[2] Masters or Doctoral degree, or equivalent

[3] SNS: is a self-report measure of numeracy and scored as the average rating of 8 items (4 items assessed ability and 4 items assessed preferences) marked (1-6)

[4] Medical Maximizer-Minimizer Scale (MMM): medical maximizers (seek health care even for minor problems), medical minimizers: (avoid medical intervention unless it is necessary)

[5]Percentages were reported to one decimal

For peer review only - http://bmjopen.bmj.com/site/about/guidelines.xhtml

Trial A

Comprehension

Figure 2A shows the mean comprehension score for each group. For the percentage of participants, in each group, who correctly answered the comprehension questions, see additional file 2. The total mean score across all questions was 3.71 (SD 1.63), 3.79 (SD 1.54), 3.81 (SD 1.53), and 4.25 (SD 1.40) for text-only, bar graph, line graph, and pictograph groups respectively. Only pictograph group participants had statistically significantly superior comprehension to the text-only group participants, with a mean difference (MD) of 0.54 (95% CI 0.13 to 0.95, *p*-value 0.011).

There was no significant difference between the bar graph group and the text-only group or between the line graph group and the text-only group (Table 2). After adjustment for age group, education level, and health literacy: pictograph was not statistically different compared to text-only (MD 0.40, 95% CI 0.04 to 0.76, *p*-value 0.031). Although comprehension was higher in participants in the pictograph group than those in the other two graph groups, the 95% CIs are within the prespecified equivalence threshold, suggesting the differences among the three graph groups are not clinically meaningful. (See Additional file 2).

Health literacy was strongly correlated with comprehension (r^2 = 0.24; *p*-value 0.0001) with higher health literacy associated with better comprehension. No association was found between education level and comprehension (r^2= 0; *p*-value 0.47). The association between numeracy and comprehension was weak although statistically significant (r^2=.04; *p*-value 0.0001) (See Additional file 2).

Please insert figure 2 here

For peer review only - http://bmjopen.bmj.com/site/about/guidelines.xhtml

Table 2: Outcomes: Superiority comparisons of mean comprehension scores**,** Decision intention, Satisfaction with the presentation, Visual presentation preference in all intervention groups, in Trial A and Trial B.

	Trial A				Trial B			
	**Text only (n= 103)**	**Bar graph (n= 113)**	**Pictograph (n= 110)**	**Line graph (n= 91)**	**Text only (n= 107)**	**Bar graph (n= 113)**	**Pictograph (n= 111)**	**Line graph (n= 102)**
**Superiority comparison (not adjusted)**								
Mean (SD)	3.71 (1.63)	3.79 (1.54)	4.25 (1.40)	3.81 (1.53)	3.74 (1.60)	3.95 (1.59)	4.23 (1.59)	3.96 (1.89)
MD	Ref	0.08	0.54	0.1	Ref	0.21	0.49	0.22
95% CI	Ref	-0.33 to 0.49	0.13 to 0.95	-0.33 to 0.54	Ref	-0.23 to 0.65	0.04 to 0.93	-0.23 to 0.68
P-value	Ref	0.70	0.011	0.63	Ref	0.35	0.032	0.34
**Superiority comparison (adjusted for age group, level of education and categorised health literacy)**								
MD[1]	Ref	0.19	0.4	0.06	Ref	0.1	0.38	0.1
95% CI	Ref	-0.16 to 0.55	0.04 to 0.76	-0.32 to 0.44	Ref	-0.27 to 0.47	0.01 to 0.74	-0.27 to 0.48
P-value	Ref	0.29	0.031	0.76	Ref	0.59	0.046	0.59
**Decision intention n (%) before the intervention**								
Take antibiotics	68 (66.0)	73 (64.6)	69 (62.7)	62 (68.1)				
Not to take antibiotics	18 (17.5)	17 (15.0)	18 (16.4)	17 (18.7)				
Unsure	17 (16.5)	23 (20.4)	23 (20.9)	12 (13.2)	10 (9.4)	11 (9.7)	12 (10.8)	12 (11.8)
To wait and watch					58 (54.2)	63 (55.8)	61 (55.0)	45 (44.1)
To have physiotherapy					27 (25.2)	28 (24.8)	28 (25.2)	29 (28.4)
To have corticosteroid					12 (11.2)	11 (9.7)	10 (9.0)	16 (15.7)
**Decision intention n (%) after the intervention**								
Take antibiotics	75 (72.8)	73 (64.6)	87 (79.1)	67 (73.6)				
Not to take antibiotics	13 (12.6)	22 (19.5)	14 (12.7)	12 (13.2)				
Unsure	15 (14.6)	18 (15.9)	9 (8.2)	12 (13.2)	5 (4.7)	7 (6.2)	5 (4.5)	7 (6.9)
To wait and watch					39 (36.4)	47 (41.6)	47 (42.3)	34 (33.3)
To have physiotherapy					45 (42.1)	43 (38.1)	47 (42.3)	49 (48.0)
To have corticosteroid					18 (16.8)	16 (14.2)	12 (10.8)	12(11.8)
**Satisfaction mean (SD)**								
How easy (1-10)[2]	7.2 (2.2)	7.3 (2.5)	7.7 (2.0)	7.6 (2.2)	7.8 (1.8)	7.8 (2.2)	8.3 (1.8)	7.5 (2.6)
How satisfied (1-10)[3]	7.0 (2.2)	7.4 (2.4)	7.7 (2.0)	7.6 (2.2)	7.8 (1.9)	8.0 (2.0)	8.3 (1.9)	7.7 (2.4)
**Visual presentation preference- most preferred (1st) N (%)**								
Text only	40 (38.8)	38 (33.6)	25 (22.7)	21 (23.1)	43 (40.2)	40 (35.4)	27 (24.3)	34 (33.3)
Bar graph	31 (30.1)	43 (38.1)	34 (30.9)	29 (31.9)	40 (37.4)	56 (49.6)	28 (25.2)	30 (29.4)
Pictograph	9 (8.7)	8 (7.1)	34 (30.9)	7 (7.7)	12 (11.2)	8 (7.1)	45 (40.5)	3 (2.9)
Line graph	23 (22.3)	24 (21.2)	17 (15.5)	34 (37.4)	12 (11.2)	9 (8.0)	11 (9.9)	35 (34.3)
**Visual presentation preference- least preferred (4th) N (%)**								
Text only	29 (28.2)	25 (22.1)	47 (42.7)	29 (31.9)	20 (18.7)	30 (26.6)	41 (36.9)	32 (31.4)
Bar graph	10 (9.7)	12 (10.6)	12 (10.9)	15 (16.5)	6 (5.6)	8 (7.1)	9 (8.1)	7 (6.9)
Pictograph	43 (41.8)	56 (49.6)	25 (22.7)	34 (37.4)	41 (38.3)	34 (30.1)	11 (9.9)	45 (44.1)
Line graph	21 (20.4)	20 (17.7)	26 (23.6)	13 (14.3)	40 (37.4)	41 (36.3)	50 (45.1)	18 (17.7)
**Previous graph experience[4] mean (SD)**								
Bar graph (1-5)	3.3 (1.2)	3.3 (1.4)	3.4 (1.2)	3.3 (1.2)	3.6 (1.2)	3.6 (1.2)	3.6 (1.1)	3.3 (1.2)
Pictograph (1-5)	2.7 (1.2)	2.6 (1.3)	2.9 (1.3)	2.4 (1.1)	2.5 (1.4)	2.5 (1.3)	2.9 (1.3)	2.4 (1.2)
Line graph (1-5)	3.6 (1.1)	3.3 (1.3)	3.5 (1.1)	3.7 (1.1)	3.5 (1.2)	3.3 (1.2)	3.4 (1.3)	3.6 (1.3)

[1]MD is the mean difference from text only group
[2]1=not at all easy to 10= extremely easy
[3]1= not at all satisfied to 10= I am totally satisfied
[4] 1= No experience to 5= a lot of experience

For peer review only

For peer review only - http://bmjopen.bmj.com/site/about/guidelines.xhtml

Decision intention

At baseline, about two-thirds of participants (66.0% in text-only, 64.6% in bar graph, 62.7% in pictograph, 68.1% in line graph) chose the option that a child with AOM should usually take antibiotics. This increased after viewing the intervention in three of the groups (to 72.8%, 79.1%, 73.6% in text-only, pictograph, and line graph respectively) while in the bar graph group, the percentage remained similar (64.6%). Regardless of group, more people chose to give the child antibiotics after receiving the prognostic information (n=272,65.2% before; n=302,72.4% after). At least 50% in each group did not alter their choice, regardless of which intervention they received. (Figure 3A). The most common reason that participants gave for choosing antibiotics was their belief that antibiotics are effective. Other reasons are reported in the Additional file 2.

Satisfaction

Mean scores for ease of use and satisfaction were similar for all formats, with pictograph scores slightly higher (Table 2).

Visual presentation preference

Participants ranked their allocated intervention as the highest, except for participants allocated to the pictograph group who ranked pictograph and bar graph equally best (30.9% of participants). Overall and regardless of the randomised group, the bar graph was the most preferred option; chosen by 137 (32.9%), followed by text-only (n=124, 29.7%). The pictograph was the option least likely to be ranked as the first choice (chosen by 58,13.9%) (Table 2). For the least preferred options and reasons for preferences, see Additional file 2.

For peer review only - http://bmjopen.bmj.com/site/about/guidelines.xhtml

Trial B

Comprehension

Comprehension scores were higher in the pictograph group compared to the text-only group (MD 0.49, 95% CI 0.04 to 0.93, *p*-value 0.032) however the difference was not statistically significant. The differences between the bar graph and text-only group and between the line graph and text-only group were not statistically significant (Table 2).

Figure 2B shows the mean comprehension score for each group. For the percentage of participants, in each group, who answered the comprehension questions correctly, see additional file 2.

Health literacy was found to strongly correlate with comprehension with higher health literacy associated with better comprehension (r^2=.29; *p*-value <.0001). Comprehension was weakly associated with numeracy (r^2=.03; *p*-value 0.0001) and education level (r^2=0.02; *p*-value 0.0024)

Decision intention

Overall, before receiving any prognostic information, 52.4% (n=227) of participants intended to wait and see when asked about decision intentions. After receiving the information, 30.0% of those changed their choice to physiotherapy, while 11.0% changed to corticosteroid (Figure 3B). A similar pattern was observed across all four interventions (Table 2). Of the participants who chose physiotherapy (the most common choice after receiving the information, regardless of its type), the most common reasons for doing so were their understanding of the presented information followed by their belief that this was the most effective option. For other reasons see Additional file 2.

Please insert figure 3 here

For peer review only - http://bmjopen.bmj.com/site/about/guidelines.xhtml

Satisfaction

Mean scores for ease of use and satisfaction were similar for all formats, with pictograph scores slightly higher (Table 2).

Visual presentation preference

Overall, the bar graph was the most preferred option for receiving prognostic information, chosen by 35.6% of participants, followed by 33.3% who ranked text-only as their most preferred format. The bar graph was also the highest to be chosen as a second preference (35% of participants). By intervention group, 40.2% of the text-only group, 49.6% of bar graph group, 40.5% of pictograph group, and 34.31% of line graph group ranked their allocated intervention as their most preferred option to receive prognostic information in the future.

Table 2 shows the number and proportion of participants, in each group, who chose each format as their most preferred or least preferred option (other ranking choices are provided in Additional file 2).

Discussion

In our two parallel online randomised controlled trials, there were no clinically meaningful differences in comprehension between the groups that viewed the bar graph, pictograph, or line graph. Only participants who were randomised to the pictograph presentation had statistically significant better comprehension of quantitative prognostic information compared to participants who were randomised to text-only (MD 0.54, 95% CI 0.13- 0.95, p 0.011), but this only occurred in trial A (AOM scenario). After all visual presentations were revealed to all participants, the bar graph was the most preferred option for receiving prognostic information in the

For peer review only - http://bmjopen.bmj.com/site/about/guidelines.xhtml

future; chosen by about one-third of participants in both trials. The type of visual presentation viewed did not appear to influence change in decision intention.

There were potential limitations to these two trials; participants were members of an online survey provider and did not need to have experience with the conditions studied, and only spent about 15-20 minutes engaged in completing the survey. We were not able to assess participants' baseline knowledge as the comprehension questions asked for the exact numbers which we expected to be inapplicable for participants without showing them some information. The impact of prior experience, or lack of, is unclear as previous experience may have influenced decision intention, whereas no experience or personal relevance may have reduced engagement. As the questions about decision intention were hypothetical, motivation to properly read the information presented and consider the decision options may have been low. Additionally, to make a well-informed choice, patients need information about both the benefits and harms of each available treatment option. We did not present harms data as part of the information, and this may have influenced responses to questions about decision intention. Although we chose two conditions that are typically managed in primary health care settings, our findings should be interpreted with caution and might not apply to the communication of other health conditions and might be different if the information was presented in face-to-face communication.

Our trials have several strengths, including testing the visual presentations in two conditions with different trajectories and conducting the trials in parallel. Measures were used to prevent cross contamination between the trials (e.g., participants of trial A were not invited to trial B, and participants of the piloting phase were not invited to participate in any of the trials).

For peer review only - http://bmjopen.bmj.com/site/about/guidelines.xhtml

403 The results from our systematic review found no clear evidence to support the superiority of any particular visual presentation to communicate prognosis, with existing research mostly investigating the communication of long-term prognosis for various types of cancer.[6] In our current trials, using non-cancer conditions and shorter prognostic duration, the difference between the various graph types on comprehension or decision intention was not significant. We found some minor inconsistencies between participants' preferred graph type and their ratings of satisfaction and ease of use of the various graphs. It appears that no graph type is clearly superior at facilitating comprehension, strongly preferred by participants, and viewed as the easiest to understand. Prior to the widespread use of a graph to convey prognostic information, piloting it with the target audience (ensuring a range of health literacy levels) may help to ensure appropriateness.

415 Several reviews and primary studies of methods of communicating treatment or screening benefits and harms have recommended adding visual presentations to textual information when communicating quantitative data.[13,22,32,33] For example, a test of three different visual presentations (text-only, fact box, visual aid) for communicating mortality with and without screening for prostate and breast cancer found that visual aids (i.e., a pictograph) generally increased comprehension by 18%.[24] However, in our trials, we found that the pictograph was superior to text-only, but only in trial A (AOM scenario). Reasons for this are unclear and might be explained by more complicated prognostic information, and more treatment options, were presented in trial B (e.g., the prognostic course was more variable at the different time periods).

426 Visual presentations are often incorporated into patient decision aids, which are tools that can be used to support shared decision-making and contain quantitative

For peer review only - http://bmjopen.bmj.com/site/about/guidelines.xhtml

information about the benefits and harms of options for managing a condition. The International Patient Decision Aids Standards (IPDAS) collaboration recently reviewed the evidence about using and displaying numbers when designing patient decision aids and recommended adding a visual presentation (e.g., pictographs, bar charts) to communicate quantitative information, without specific recommendation about which type to use.[32]

Line graphs have traditionally been avoided when communicating with patients as it has been assumed they are too difficult to understand[18], however, our study did not find significantly poor comprehension in participants in the line graph group. These findings suggest line graphs are an appropriate graph that might be used to communicate prognostic information. As showing the trajectory of the condition over time is often very important, a line graph may be best suited to use when there are outcome data for more than two time points, as the equivalent information would need multiple pictographs (one at each time point) and the trajectory less clear.

Communicating quantitative information can be more challenging when multiple management options are available or multiple important outcomes. Further studies are needed to investigate how to best visually, and verbally, communicate more complex prognostic information.

Conclusion

Although the pictograph was statistically superior to the text-only presentation in one of the two trials conducted, we did not find a clinically important difference between the types of graphs tested. Our findings suggest that, if the information is clearly displayed, any of the tested visual presentations can be used to communicate quantitative prognostic information.

For peer review only - http://bmjopen.bmj.com/site/about/guidelines.xhtml

Ethics approval and consent to participate

This study received ethical approval from Bond University Human Research Ethics Committee (EA00088). All methods in this study were performed in accordance with the Declaration of Helsinki. Informed consent was obtained from all Participants to receive online surveys by Dynata survey company. Additionally, before starting the survey, participants were provided with a formal invitation to participate in the study which included information about the study, and their right to withdraw at any time point with no negative consequences

Consent for publication

Not applicable

Availability of data and materials

The datasets (raw data) and statistical codes generated during the current study are available in the Open Science Framework repository (OSF), https://osf.io/p2t9w/?view_only=36b1a232ef444d5fb5241735046e34dd

All data analysed during this study are included in this published article and its supplementary information files. (Additional files 1, 2).

A copy of the registered protocol is provided as a supplementary.

Authors' contributions

EA, TH, and CDM conceived the idea of this study. EA developed the interventions with support from TH, CDM and MB. MB helped in the refinement of pictographs intervention. EA, TH and CDM developed the survey. TH was reviewing and giving feedback on all the steps. Data collection was managed by Dynata under the direction of EA, TH. Data were analysed by EA, MJ, and MB. EA drafted the main

For peer review only - http://bmjopen.bmj.com/site/about/guidelines.xhtml
For peer review only

manuscript and developed all tables and figures with support and feedback from all authors. All authors reviewed the final version of the manuscript and approved it for submission.

Competing interests

None declared

Funding

This work was supported by the Centre for Research Excellence in Minimizing Antibiotic Resistance in the Community (CRE-MARC), funded by the Australian National Health and Medical Research Council grant number (1153299).

Acknowledgements

We thank our colleagues at the Institute of Evidence-Based Healthcare (IEBH)-Bond University, for giving their feedback on the interventions and testing the survey during the pre-piloting phase. A special thank you to the childcare teachers who pilot tested Trial A surveys and to Bond University members who pilot tested Trial B surveys.

This work is dedicated to the memory of Professor Chris Del Mar, one of my PhD supervisors, who contributed fully to this work (passed away in March 2022).

For peer review only - http://bmjopen.bmj.com/site/about/guidelines.xhtml

References

1. Charles C, Gafni A, Whelan T. Shared decision-making in the medical encounter: what does it mean? (or it takes at least two to tango). *Soc Sci Med*. Mar 1997;44(5):681-92. doi:10.1016/s0277-9536(96)00221-3

2. Feemster LC, Curtis JR. "We Understand the Prognosis, but We Live with Our Heads in the Clouds": Understanding Patient and Family Outcome Expectations and Their Influence on Shared Decision Making. *Am J Respir Crit Care Med*. Feb 1 2016;193(3):239-41. doi:10.1164/rccm.201511-2125ED

3. Hoffmann TC, Del Mar C. Patients' expectations of the benefits and harms of treatments, screening, and tests: a systematic review. *JAMA Intern Med*. Feb 2015;175(2):274-86. doi:10.1001/jamainternmed.2014.6016

4. Paladino J, Lakin JR, Sanders JJ. Communication Strategies for Sharing Prognostic Information With Patients: Beyond Survival Statistics. *JAMA*. Aug 15 2019;doi:10.1001/jama.2019.11533

5. Feldman-Stewart D, Brundage MD, Zotov V. Further insight into the perception of quantitative information: judgments of gist in treatment decisions. *Med Decis Making*. Jan-Feb 2007;27(1):34-43. doi:10.1177/0272989X06297101

6. Abukmail E, Bakhit M, Del Mar C, Hoffmann T. Effect of different visual presentations on the comprehension of prognostic information: a systematic review. *BMC Med Inform Decis Mak*. Aug 25 2021;21(1):249. doi:10.1186/s12911-021-01612-9

7. Rovers MM, Zielhuis GA. Otitis media meta-analysis. *Pediatrics*. Aug 2004;114(2):508-9; author reply 508-9. doi:10.1542/peds.114.2.508-a

8. Venekamp RP, Sanders SL, Glasziou PP, Del Mar CB, Rovers MM. Antibiotics for acute otitis media in children. *Cochrane Database Syst Rev*. Jun 23 2015;(6):CD000219. doi:10.1002/14651858.CD000219.pub4

9. Bisset L, Beller E, Jull G, Brooks P, Darnell R, Vicenzino B. Mobilisation with movement and exercise, corticosteroid injection, or wait and see for tennis elbow: randomised trial. *BMJ*. Nov 4 2006;333(7575):939. doi:10.1136/bmj.38961.584653.AE

10. Lipkus IM, Hollands JG. The visual communication of risk. *J Natl Cancer Inst Monogr*. 1999;(25):149-63. doi:10.1093/oxfordjournals.jncimonographs.a024191

11. Fortin JM, Hirota LK, Bond BE, O'Connor AM, Col NF. Identifying patient preferences for communicating risk estimates: a descriptive pilot study. *BMC Med Inform Decis Mak*. 2001;1:2. doi:10.1186/1472-6947-1-2

12. Armstrong K, Schwartz JS, Fitzgerald G, Putt M, Ubel PA. Effect of framing as gain versus loss on understanding and hypothetical treatment choices: survival and mortality curves. *Med Decis Making*. Jan-Feb 2002;22(1):76-83. doi:10.1177/0272989X0202200108

13. Ancker JS, Senathirajah Y, Kukafka R, Starren JB. Design features of graphs in health risk communication: a systematic review. *J Am Med Inform Assoc*. Nov-Dec 2006;13(6):608-18. doi:10.1197/jamia.M2115

14. Feldman-Stewart D, Brennenstuhl S, Brundage MD. A purpose-based evaluation of information for patients: an approach to measuring effectiveness. *Patient Educ Couns*. Mar 2007;65(3):311-9. doi:10.1016/j.pec.2006.08.012

15. Zikmund-Fisher BJ, Fagerlin A, Ubel PA. Mortality versus survival graphs: improving temporal consistency in perceptions of treatment effectiveness. *Patient Educ Couns*. Apr 2007;66(1):100-7. doi:10.1016/j.pec.2006.10.013

16. Zikmund-Fisher BJ, Fagerlin A, Ubel PA. Improving understanding of adjuvant therapy options by using simpler risk graphics. *Cancer*. Dec 15 2008;113(12):3382-90. doi:10.1002/cncr.23959

For peer review only - http://bmjopen.bmj.com/site/about/guidelines.xhtml

17. Zikmund-Fisher BJ, Witteman HO, Dickson M, et al. Blocks, ovals, or people? Icon type affects risk perceptions and recall of pictographs. *Med Decis Making*. May 2014;34(4):443-53. doi:10.1177/0272989X13511706

18. Carling CL, Kristoffersen DT, Flottorp S, et al. The effect of alternative graphical displays used to present the benefits of antibiotics for sore throat on decisions about whether to seek treatment: a randomized trial. *PLoS Med*. Aug 2009;6(8):e1000140. doi:10.1371/journal.pmed.1000140

19. Zikmund-Fisher BJ, Fagerlin A, Ubel PA. A demonstration of ''less can be more'' in risk graphics. *Med Decis Making*. Nov-Dec 2010;30(6):661-71. doi:10.1177/0272989X10364244

20. Brown SM, Culver JO, Osann KE, et al. Health literacy, numeracy, and interpretation of graphical breast cancer risk estimates. *Patient Educ Couns*. Apr 2011;83(1):92-8. doi:10.1016/j.pec.2010.04.027

21. Trevena LJ, Zikmund-Fisher BJ, Edwards A, et al. Presenting quantitative information about decision outcomes: a risk communication primer for patient decision aid developers. *BMC Med Inform Decis Mak*. 2013;13 Suppl 2:S7. doi:10.1186/1472-6947-13-S2-S7

22. Zipkin DA, Umscheid CA, Keating NL, et al. Evidence-based risk communication: a systematic review. *Ann Intern Med*. Aug 19 2014;161(4):270-80. doi:10.7326/M14-0295

23. Hamstra DA, Johnson SB, Daignault S, et al. The impact of numeracy on verbatim knowledge of the longitudinal risk for prostate cancer recurrence following radiation therapy. *Med Decis Making*. Jan 2015;35(1):27-36. doi:10.1177/0272989X14551639

24. Petrova D, Garcia-Retamero R, Cokely ET. Understanding the Harms and Benefits of Cancer Screening: A Model of Factors That Shape Informed Decision Making. *Med Decis Making*. Oct 2015;35(7):847-58. doi:10.1177/0272989X15587676

25. Kasper J, van de Roemer A, Pottgen J, et al. A new graphical format to communicate treatment effects to patients-A web-based randomized controlled trial. *Health Expect*. Aug 2017;20(4):797-804. doi:10.1111/hex.12522

26. McDowell M, Gigerenzer G, Wegwarth O, Rebitschek FG. Effect of Tabular and Icon Fact Box Formats on Comprehension of Benefits and Harms of Prostate Cancer Screening: A Randomized Trial. *Med Decis Making*. Jan 2019;39(1):41-56. doi:10.1177/0272989X18818166

27. Brick C, McDowell M, Freeman ALJ. Risk communication in tables versus text: a registered report randomized trial on 'fact boxes'. *R Soc Open Sci*. Mar 2020;7(3):190876. doi:10.1098/rsos.190876

28. Scherer LD, Caverly TJ, Burke J, et al. Development of the Medical Maximizer-Minimizer Scale. *Health Psychol*. Nov 2016;35(11):1276-1287. doi:10.1037/hea0000417

29. Fagerlin A, Zikmund-Fisher BJ, Ubel PA, Jankovic A, Derry HA, Smith DM. Measuring numeracy without a math test: development of the Subjective Numeracy Scale. *Med Decis Making*. Sep-Oct 2007;27(5):672-80. doi:10.1177/0272989X07304449

30. Zikmund-Fisher BJ, Smith DM, Ubel PA, Fagerlin A. Validation of the Subjective Numeracy Scale: effects of low numeracy on comprehension of risk communications and utility elicitations. *Med Decis Making*. Sep-Oct 2007;27(5):663-71. doi:10.1177/0272989X07303824

31. Weiss BD, Mays MZ, Martz W, et al. Quick assessment of literacy in primary care: the newest vital sign. *Ann Fam Med*. Nov-Dec 2005;3(6):514-22. doi:10.1370/afm.405

32. Trevena LJ, Bonner C, Okan Y, et al. Current Challenges When Using Numbers in Patient Decision Aids: Advanced Concepts. *Med Decis Making*. Mar 4 2021:272989X21996342. doi:10.1177/0272989X21996342

33. Garcia-Retamero R, Galesic M. Who profits from visual aids: overcoming challenges in people's understanding of risks [corrected]. *Soc Sci Med*. Apr 2010;70(7):1019-25. doi:10.1016/j.socscimed.2009.11.031

For peer review only

For peer review only - http://bmjopen.bmj.com/site/about/guidelines.xhtml

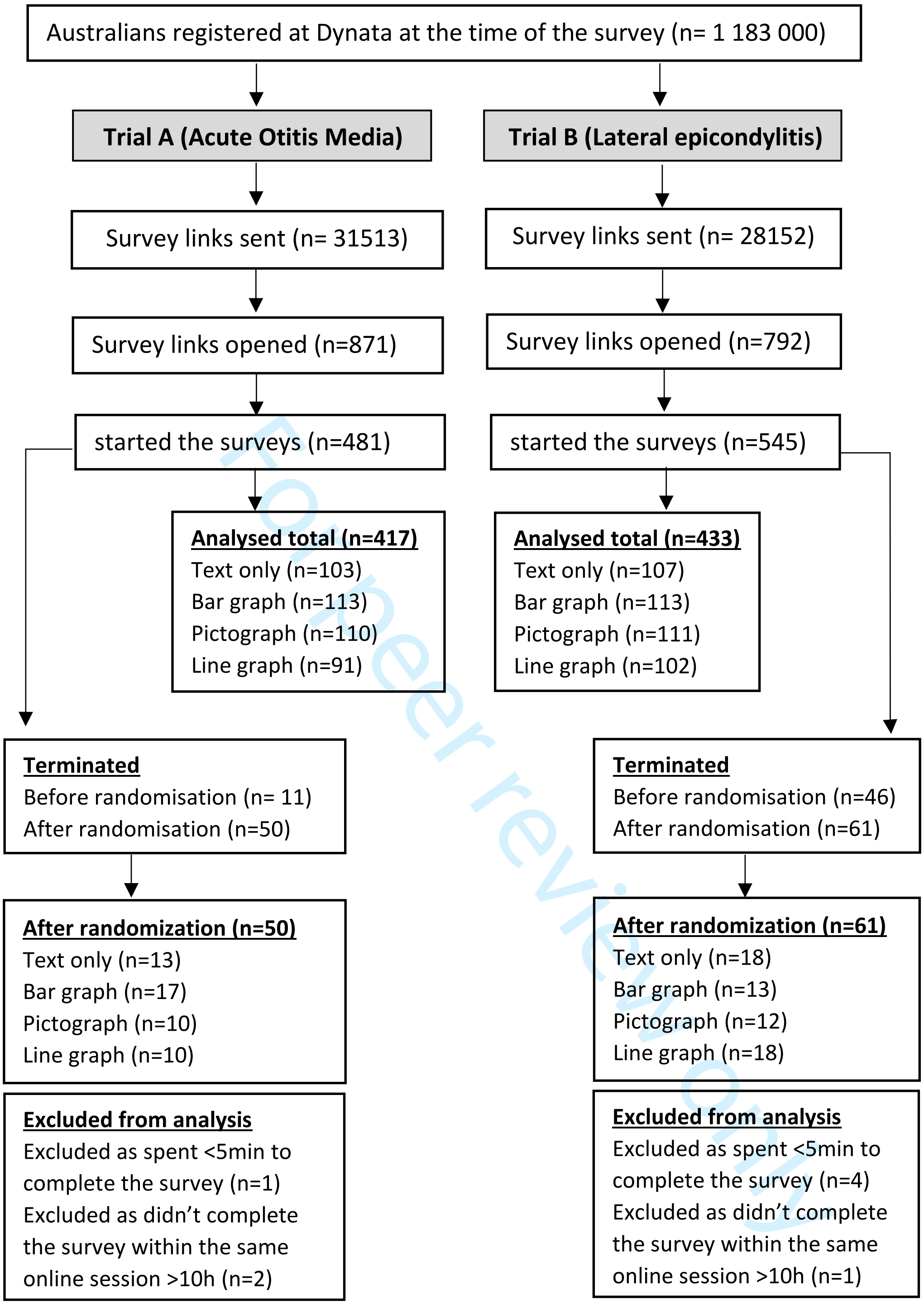

Figure 1: CONSORT flow diagrams of participants in Trial A and Trial B.

For peer review only

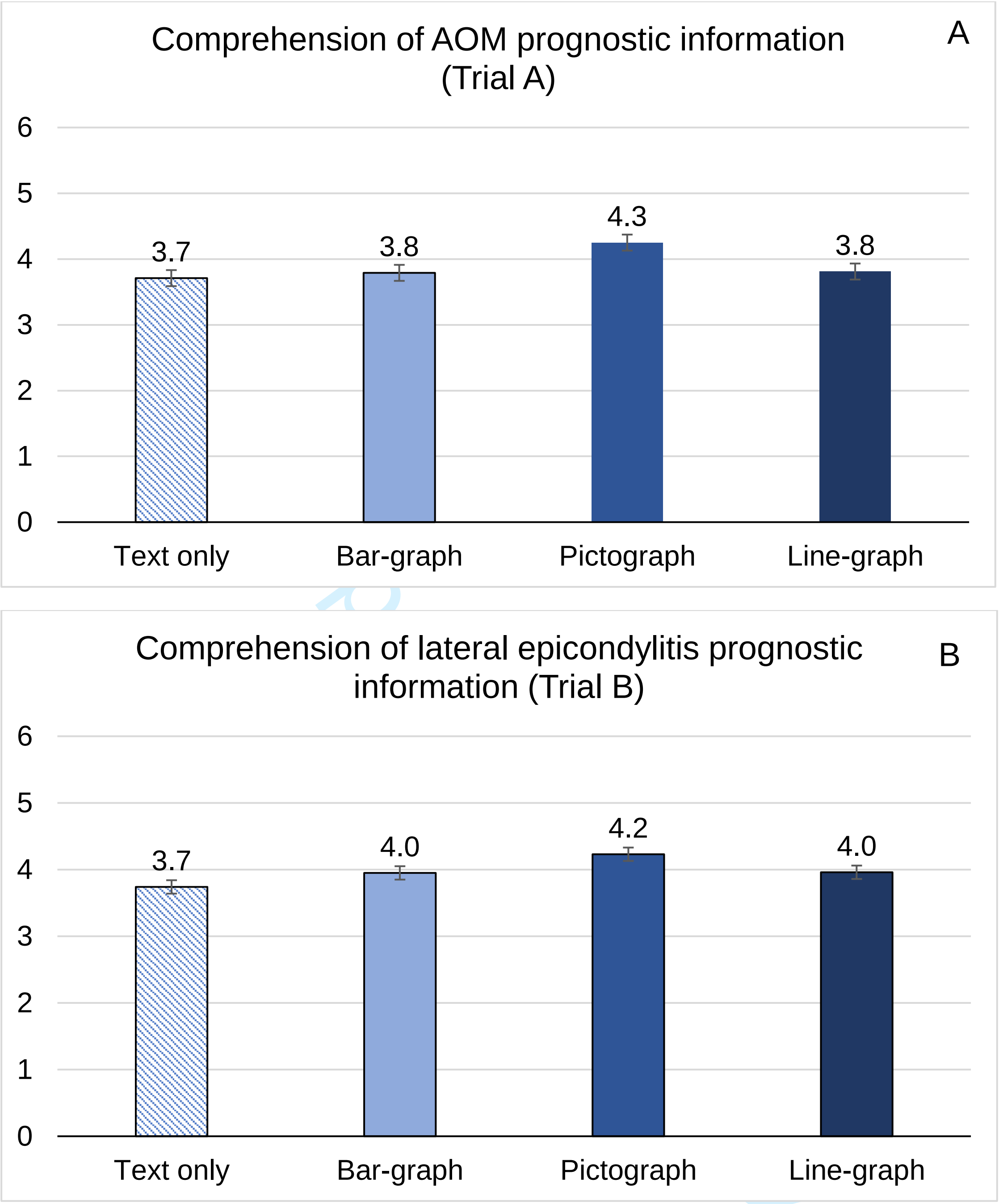

Figure 2: Mean comprehension score of prognostic information per group in trial A (AOM) and trial B (lateral epicondylitis). Comprehension questions are provided in additional file 1. Trial A and B comprehension questions are different.

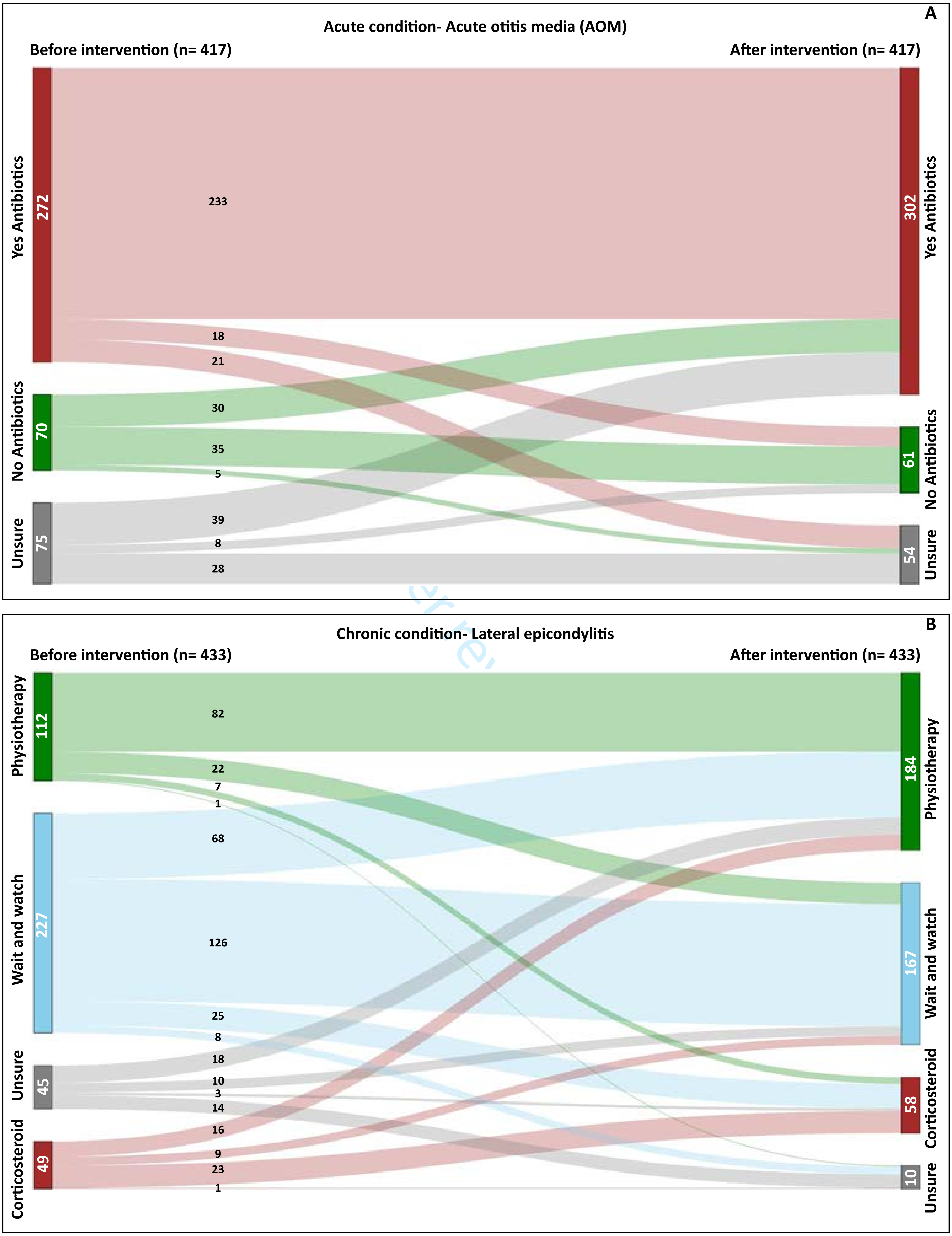

Figure 3: Overall change in treatment decision intentions before and after viewing the interventions.
The figure shows the number of participants who chose each management option in (A) acute condition-AOM and (B) chronic condition-lateral epicondylitis.

Additional file 1

(Supplementary methods)

CONSORT checklist **2**

Dynata (demographics in Australia, recruitment strategies) **5**

Interventions (visual presentations)

Trial A (Acute otitis media) **6**

Trial B (Lateral epicondylitis) **9**

The flow of the trials and outcome measurements **11**

Survey **12**

For peer review only

For peer review only - http://bmjopen.bmj.com/site/about/guidelines.xhtml

CONSORT 2010 checklist of information to include when reporting a randomised trial*

Section/Topic	Item No	Checklist item	Reported on page No
**Title and abstract**			
	1a	Identification as a randomised trial in the title	1
	1b	Structured summary of trial design, methods, results, and conclusions (for specific guidance see CONSORT for abstracts)	2-3
**Introduction**			
Background and objectives	2a	Scientific background and explanation of rationale	4-5
	2b	Specific objectives or hypotheses	5
**Methods**			
Trial design	3a	Description of trial design (such as parallel, factorial) including allocation ratio	5
	3b	Important changes to methods after trial commencement (such as eligibility criteria), with reasons	9
Participants	4a	Eligibility criteria for participants	5
	4b	Settings and locations where the data were collected	5
Interventions	5	The interventions for each group with sufficient details to allow replication, including how and when they were actually administered	6-7
Outcomes	6a	Completely defined pre-specified primary and secondary outcome measures, including how and when they were assessed	7-8
	6b	Any changes to trial outcomes after the trial commenced, with reasons	NA
Sample size	7a	How sample size was determined	8
	7b	When applicable, explanation of any interim analyses and stopping guidelines	NA
Randomisation:			
Sequence generation	8a	Method used to generate the random allocation sequence	6
	8b	Type of randomisation; details of any restriction (such as blocking and block size)	6
Allocation concealment mechanism	9	Mechanism used to implement the random allocation sequence (such as sequentially numbered containers), describing any steps taken to conceal the sequence until interventions were assigned	6
Implementation	10	Who generated the random allocation sequence, who enrolled participants, and who assigned participants to interventions	6

For peer review only - http://bmjopen.bmj.com/site/about/guidelines.xhtml

Blinding	11a	If done, who was blinded after assignment to interventions (for example, participants, care providers, those assessing outcomes) and how	10
	11b	If relevant, description of the similarity of interventions	6-7
Statistical methods	12a	Statistical methods used to compare groups for primary and secondary outcomes	9
	12b	Methods for additional analyses, such as subgroup analyses and adjusted analyses	9-10
**Results**			
Participant flow (a diagram is strongly recommended)	13a	For each group, the numbers of participants who were randomly assigned, received intended treatment, and were analysed for the primary outcome	Figure 3
	13b	For each group, losses and exclusions after randomisation, together with reasons	Figure 3
Recruitment	14a	Dates defining the periods of recruitment and follow-up	5
	14b	Why the trial ended or was stopped	NA
Baseline data	15	A table showing baseline demographic and clinical characteristics for each group	Table 1
Numbers analysed	16	For each group, number of participants (denominator) included in each analysis and whether the analysis was by original assigned groups	Tables and figures
Outcomes and estimation	17a	For each primary and secondary outcome, results for each group, and the estimated effect size and its precision (such as 95% confidence interval)	10-14, and tables
	17b	For binary outcomes, presentation of both absolute and relative effect sizes is recommended	NA
Ancillary analyses	18	Results of any other analyses performed, including subgroup analyses and adjusted analyses, distinguishing pre-specified from exploratory	Additional file 2
Harms	19	All important harms or unintended effects in each group (for specific guidance see CONSORT for harms)	Ethics application
**Discussion**			
Limitations	20	Trial limitations, addressing sources of potential bias, imprecision, and, if relevant, multiplicity of analyses	14-15
Generalisability	21	Generalisability (external validity, applicability) of the trial findings	15-16
Interpretation	22	Interpretation consistent with results, balancing benefits and harms, and considering other relevant evidence	14-17
**Other information**			

For peer review only - http://bmjopen.bmj.com/site/about/guidelines.xhtml

Registration	23	Registration number and name of trial registry	5
Protocol	24	Where the full trial protocol can be accessed, if available	In the registry
Funding	25	Sources of funding and other support (such as supply of drugs), role of funders	18

*We strongly recommend reading this statement in conjunction with the CONSORT 2010 Explanation and Elaboration for important clarifications on all the items. If relevant, we also recommend reading CONSORT extensions for cluster randomised trials, non-inferiority and equivalence trials, non-pharmacological treatments, herbal interventions, and pragmatic trials. Additional extensions are forthcoming: for those and for up to date references relevant to this checklist, see www.consort-statement.org.

For peer review only

Dynata (demographics in Australia, recruitment strategies)

(Screenshots were taken from the information documents Dynata shared with the researchers at the start of the study and gave permission to share)

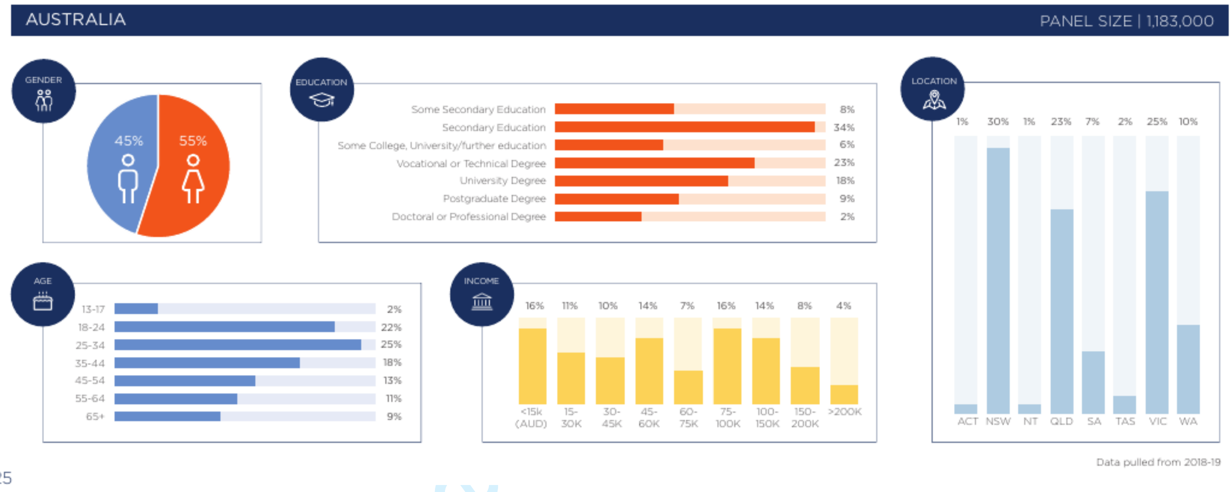

Recruitment Methodologies

SOURCE NAME	RECRUITMENT
Loyalty Panels	By invitation to 1000's of loyalty program members in travel, entertainment, media and retail.
Organic, Open Enrolment & Partnerships	1000's of websites, social media influencers and mobile apps.
Affiliate Network	Broad range of websites, incl. schools, communities. Member logging into communities with valid username and password invited to participate.

5

Interventions (visual presentations)

Trial A (Acute otitis media)

Pictograph

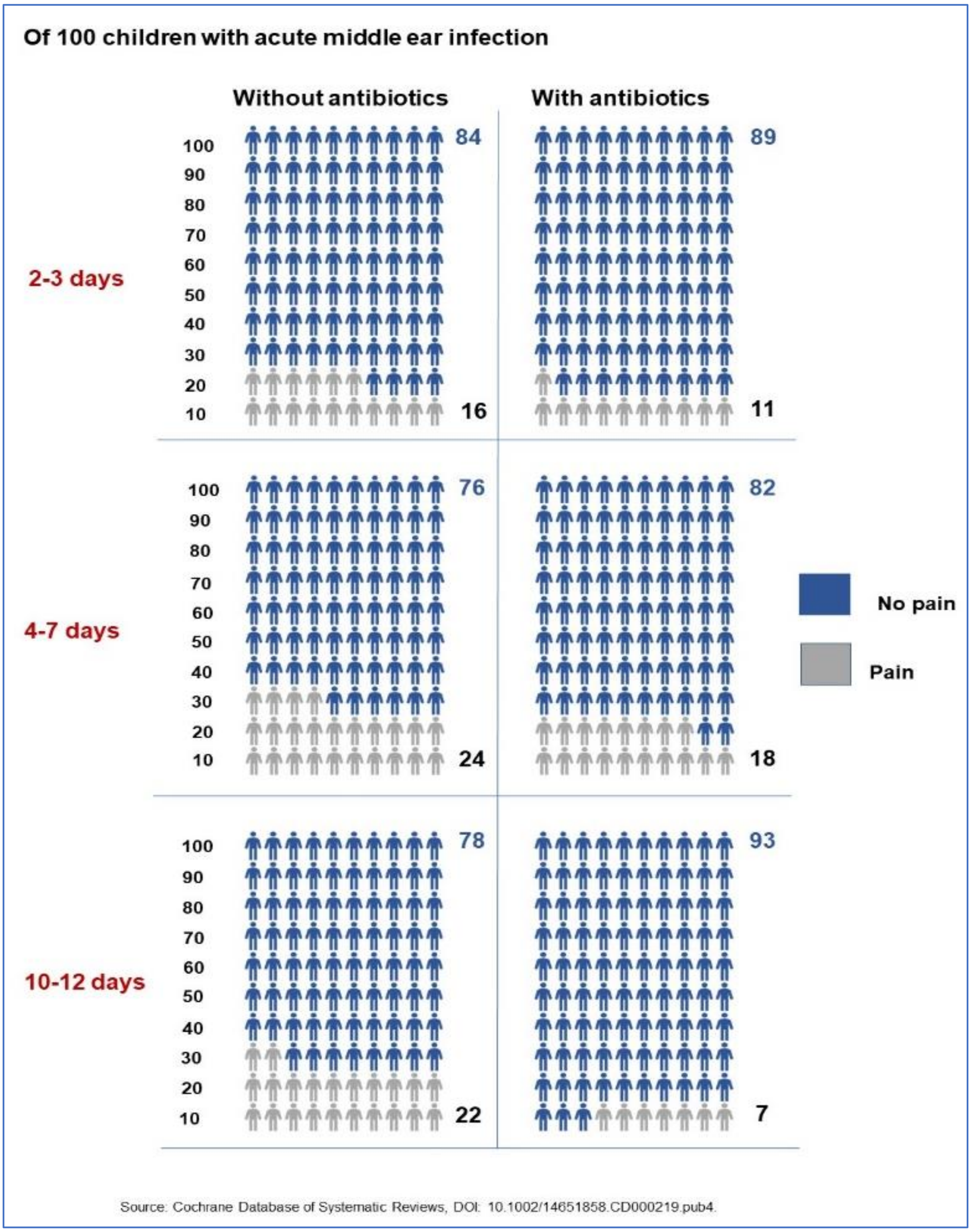

Bar graph

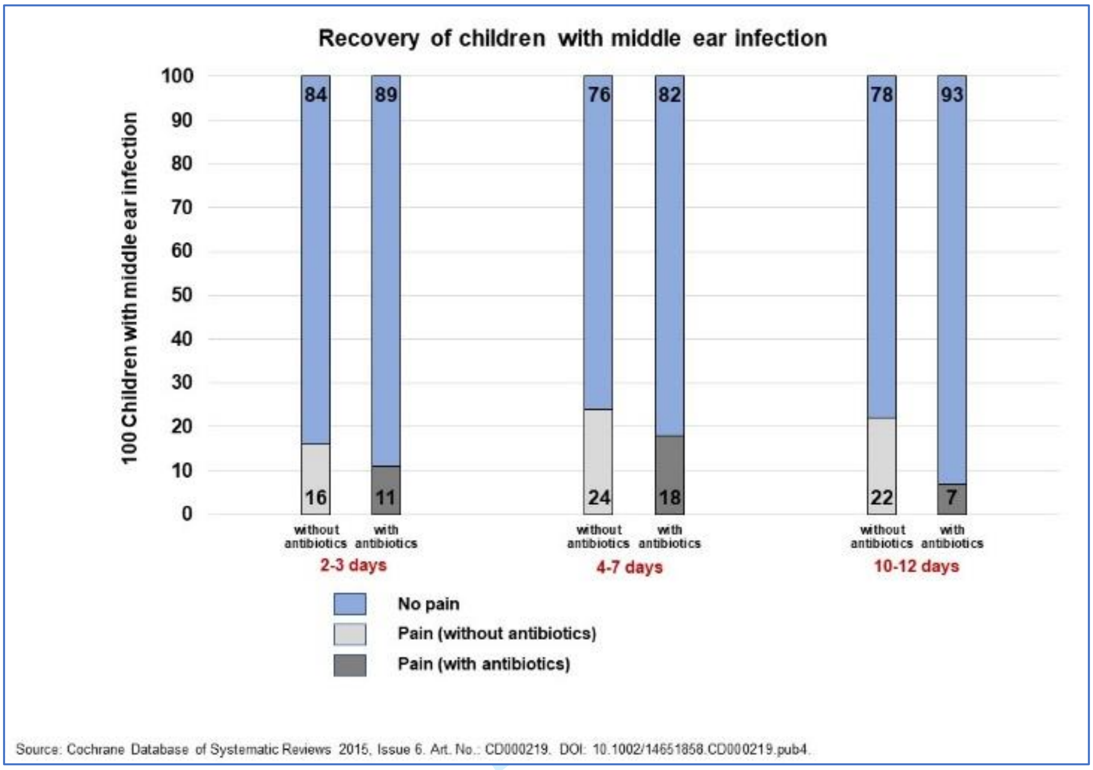

Text-only

Recovery of children with middle ear infection

Of 100 children who have middle ear infection:

Without antibiotic treatment

16 children will still experience pain at **2-3 days** after diagnosis. 24 children will have pain at **4-7 days** and 22 children will have pain at **10-12 days**. The others will have no pain.

With antibiotic treatment

11 children will still experience pain at **2-3 days** after diagnosis, 18 children will have pain at **4-7 days** and 7 children will have pain at **10-12 days**. The others will have no pain.

Source: Cochrane Database of Systematic Reviews 2015, Issue 6. Art. No.: CD000219. DOI: 10.1002/14651858.CD000219.pub4.

For peer review only - http://bmjopen.bmj.com/site/about/guidelines.xhtml

Line graph

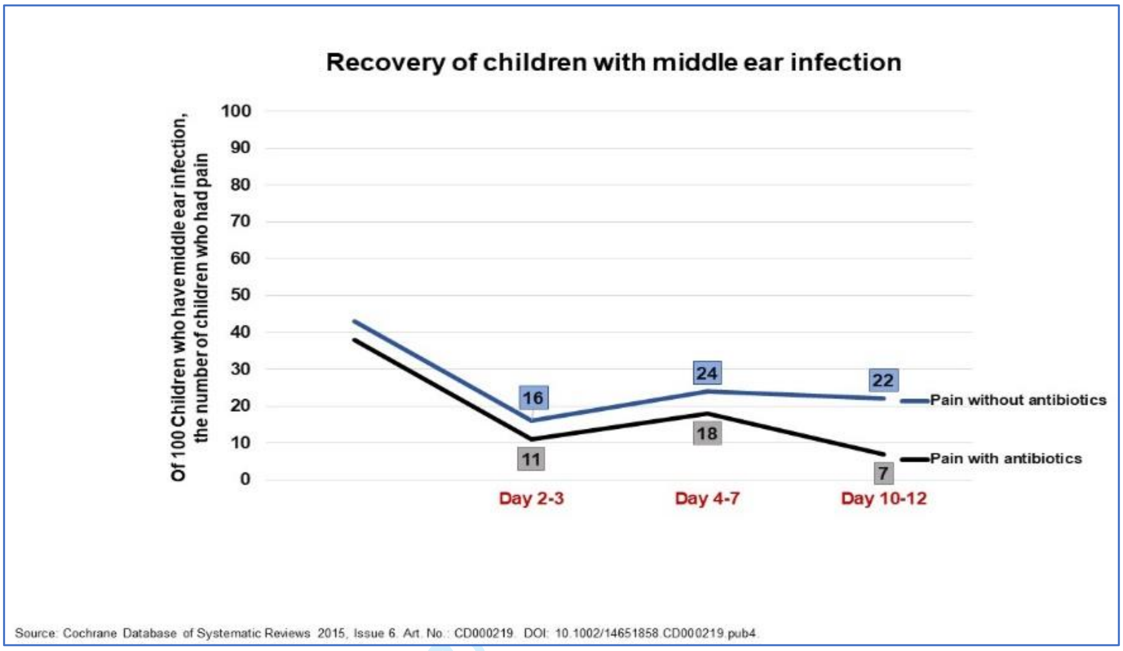

For peer review only - http://bmjopen.bmj.com/site/about/guidelines.xhtml

Trial B (Lateral epicondylitis)

Pictograph

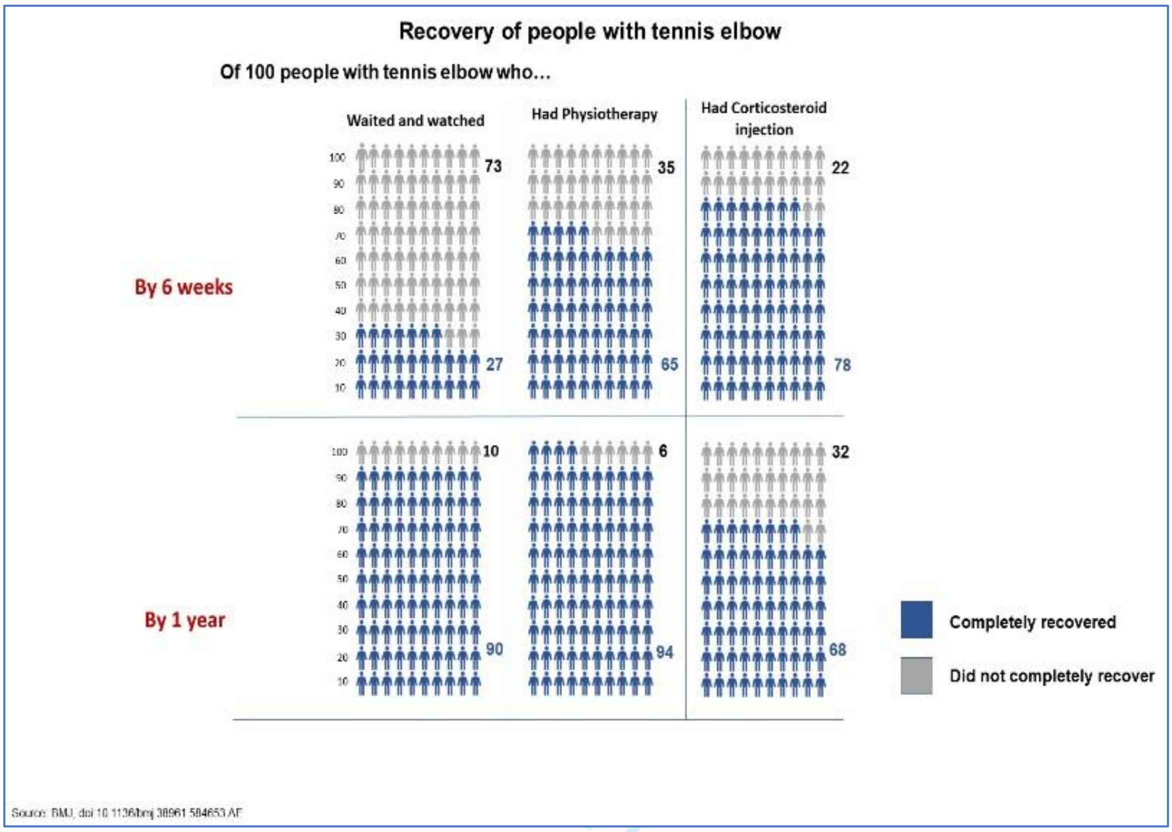

Text-only

Recovery of people with tennis elbow

Of 100 people with tennis elbow who:

Waited and watched (to see if it gets better on its own)

27 people completely recovered **by 6 weeks**. **By 1 year**, 90 people completely recovered; the other 10 did not completely recover.

Had physiotherapy

65 people completely recovered **by 6 weeks**. **By 1 year**, 94 people completely recovered; the other 6 did not completely recover.

Had corticosteroid injection

78 people completely recovered **by 6 weeks**. **By 1 year**, 68 people completely recovered; the other 32 did not completely recover.

Source: BMJ, doi:10.1136/bmj.38961.584653.AE

Bar graph

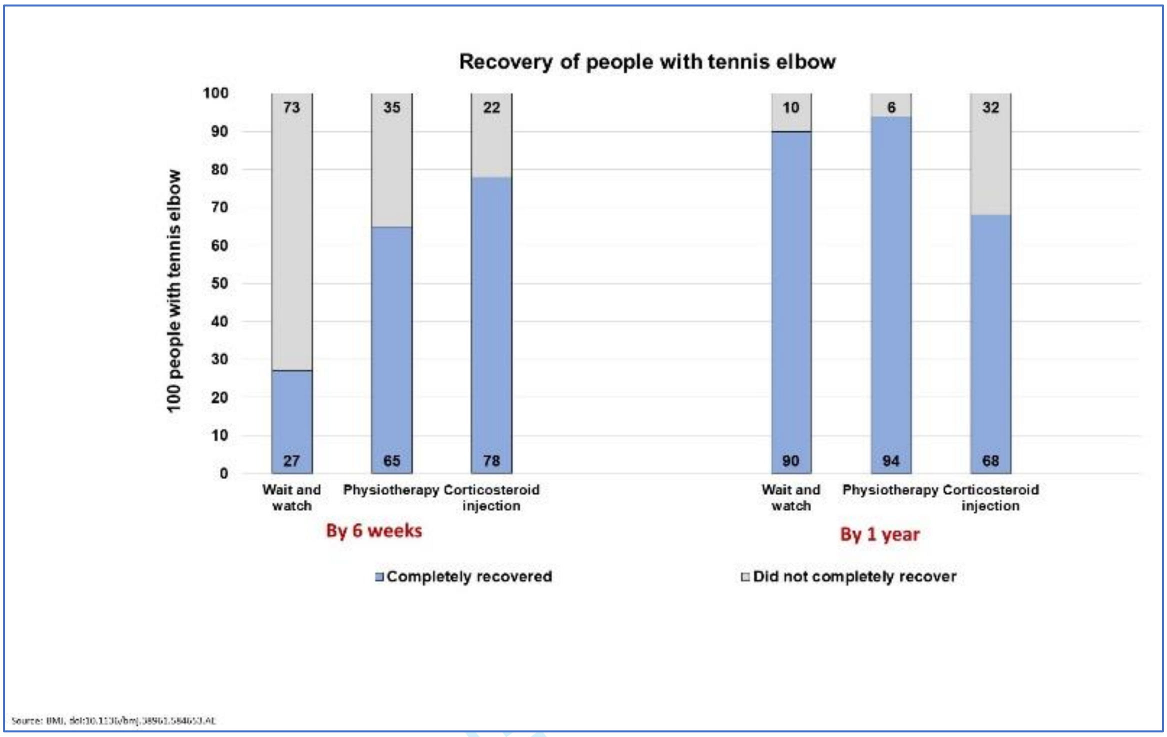

Line graph

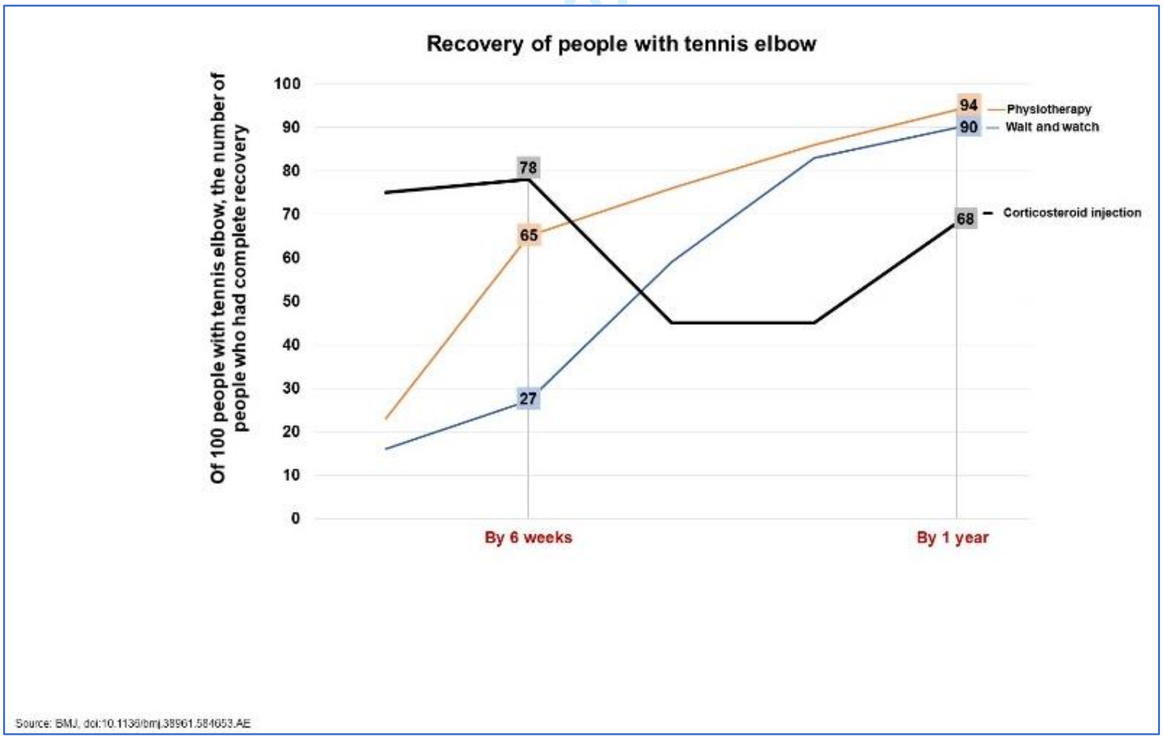

For peer review only - http://bmjopen.bmj.com/site/about/guidelines.xhtml

The flow of the trials and outcome measurements

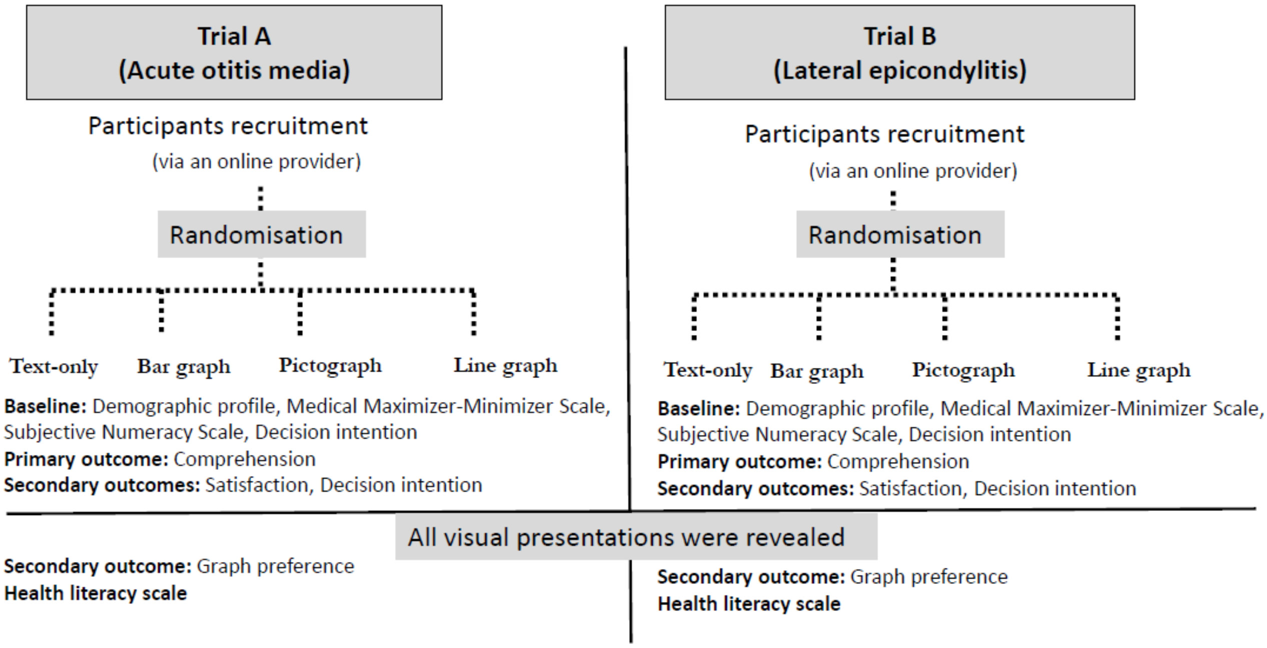

For peer review only - http://bmjopen.bmj.com/site/about/guidelines.xhtml

Survey

Questions 1-6 are the same for both trials

1. Which age group applies to you?

[ ] Under 18 [ ] 18 to 24 years [ ] 25 to 34 years
[ ] 35 to 44 years [ ] 45 to 54 years [ ] 55 to 64 years
[ ] 65 years or older [ ] Prefer not to say

2. What is your gender?

[ ] Male [ ] Female
[ ] Other [ ] Prefer not to say

3. What is your level of education? Please indicate the highest level of education you have completed.

[ ] High school - Year 10
[ ] High school – Year 12
[ ] Certificate I-IV, Diploma, or apprenticeship
[ ] Undergraduate degree: Bachelor's degree, or equivalent
[ ] Postgraduate degree: Masters or Doctoral degree, or equivalent

4. Which state or territory do you live in? [PUNCH STATE FROM POSTCODE- OKAY TO MOVE THIS AFTER Q5 IN SCRIPT- DO NOT ASK RESPONDENTS]

[ ] QLD [ ] NSW [ ] ACT [ ] NT [ ] WA [ ] VIC
[ ] TAS [ ] SA

5. What is your postcode? ___________

6. Is English the main language that you speak at home?

[ ] Yes [ ] No

Questions 7 and 8 are specific for each trial

Trial A (Acute otitis media)

7. Acute middle ear infection is an infection behind the eardrum. It is very common, usually in children, and can be very painful. You can take any pain medicine with any of the options below. Imagine that a child you take care of gets a middle ear infection, what decision about using antibiotics might you make?

[ ] The child usually should take antibiotics.
[ ] The child usually should not take antibiotics.
[ ] Unsure

8. Have you, or a person that you are responsible for, previously had an acute middle ear infection?

[ ] Yes
[ ] No

Trial B (lateral epicondylitis)

7. Tennis elbow is a condition in which people have pain and tenderness over the outside of the elbow It usually gets better by itself, regardless of the type of treatment, but symptoms may last up to 2 years[1].

---

[1] Source: Olaussen M, Holmedal O, Lindbaek M, et al Treating lateral epicondylitis with corticosteroid injections or non -electrotherapeutical physiotherapy: a systematic review BMJ Open 2013;3:e003564. doi: 10.1136/bmjopen-2013-003564

For peer review only - http://bmjopen.bmj.com/site/about/guidelines.xhtml

<u>You can take any pain medicine with any of the options below</u>**. Imagine that you have tennis elbow, what decision about other treatment options, from the below list, might you make?**

[ ] I would probably have a corticosteroid injection.
[ ] I would probably have physiotherapy.
[ ] I would probably wait and watch to see if it gets better on its own.
[ ] Unsure

8. Have you, or a person that you know, previously had tennis elbow (lateral epicondylitis)?

[ ] Yes [ ] No

Questions 9 -11 are the same for both trials (Q9 is MMM scale, Q10 and 11 are Subjective Numeracy Scale)

Comprehension questions Q12-Q17 (These questions are different for trial A and trial B)

Trial A (Acute otitis media) only

Middle ear infection is one of the most frequent diseases in childhood. Children usually experience symptoms of middle ear inflammation like pain, ear discharge, and fever.[2]

The numbers below show what happens over time to children up to 15 years of age with middle ear infection when different ways of managing it are chosen.

The intervention was inserted here

Based on the information in the XXXX (please insert the type of graph e.g., pictograph), please answer the following questions. (Used for all graph interventions)

Based on the information in the text box, please answer the following questions. (Used for all text interventions):

(Q12-Q17- ON SAME PAGE ALONG WITH THE INTERVENTION).

12. At 2-3 days, out of 100 children who did NOT take antibiotics, how many had pain?

Answer: ___________

13. At 10-12 days, out of 100 children who took antibiotics, how many had pain?

Answer: ___________

14. Which group was less likely to experience pain at about 4-7 days?

[ ] Children who took antibiotics
[ ] Children who did not take antibiotics.
[ ] No difference between the groups.
[ ] I do not know.

15. At 4-7 days, how many more children who did NOT take antibiotics will have pain, compared to those who took antibiotics?

Answer: ___________

[2] Source: Cochrane Database of Systematic Reviews 2015, Issue 6. Art. No.: CD000219. DOI: 10.1002/14651858.CD000219.pub4.

For peer review only - http://bmjopen.bmj.com/site/about/guidelines.xhtml

16. At 2-3 days, how many more/fewer children who did NOT take antibiotics will be pain-free, compared to children who did take antibiotics?

[ ] 5 fewer [ ] 5 more [ ] 16 more [ ] 11 fewer

17. In the week or so after a middle ear infection starts, the majority of children will NOT be in pain, regardless of whether they do or do not take antibiotics

[ ] True [ ] False

Trial B (Lateral epicondylitis) only

Tennis elbow is a condition in which people have pain and tenderness over the outside of the elbow. This can make some movements of the arm and wrist painful.

The numbers below show what happens over time to people with tennis elbow when different ways of managing it are chosen.

The intervention was inserted here

Based on the information in the XXXX (please insert the type of graph e.g., pictograph), please answer the following questions. (Used for all graph interventions)

Based on the information in the text box, please answer the following questions. (Used for all text interventions):

(Q12-Q17- ON SAME PAGE ALONG WITH THE INTERVENTION)

12. Of 100 people with tennis elbow who chose to 'wait and watch, how many people completely recovered by 6 weeks?

Answer: ___________

13. Of 100 people with tennis elbow who chose to have a corticosteroid injection, how many people completely recovered by 6 weeks?

Answer: ___________

14. How many <u>more</u> people who chose physiotherapy completely recovered by 6 weeks, compared to those who waited and watched?

Answer: ___________

15. Which group was <u>more likely</u> to be completely recovered by 1 year?

[ ] People who waited and watched
[ ] People who had physiotherapy
[ ] People who had a corticosteroid injection
[ ] No difference between the groups
[ ] I do not know

16. **By 1 year, how many more/ fewer people who had physiotherapy completely recovered, compared to people who had a corticosteroid injection?**

[ ] 26 fewer [ ] 26 more [ ] 32 more [ ] 6 fewer

17. **By 1 year, the majority of people who chose to wait and watch will be completely recovered, without having physiotherapy or a corticosteroid injection.**

[ ] True [ ] False

For peer review only - http://bmjopen.bmj.com/site/about/guidelines.xhtml

Question 18 is trial specific

Trial A

18. Imagine again that a child you take care of gets a middle ear infection. What decision about using antibiotics might you make?

[ ] The child usually should ***take*** antibiotics.

[ ] The child usually should ***not take*** antibiotics.

[ ] Unsure

Trial B

18. Imagine again that you have tennis elbow. What decision about the management option might you make?

[ ] I would probably have a corticosteroid injection.

[ ] I would probably have physiotherapy.

[ ] I would probably wait and watch to see if it gets better on its own.

[ ] Unsure

Questions 19-23 are the same for both trials

19. **Can you please indicate the most important reason for your decision?**

Questions 20-28 are the same for both trials

Trial A statement: You have just seen A XXXX-(PIPE IN TEXT AS PER GRAPH SHOWN pictograph bar graph, line graph, text) presenting information about the recovery of children with middle ear infection

Trial B statement: You have just seen a XXXX (PIPE IN TEXT AS PER GRAPH SHOWN pictograph bar graph, line graph, text) presenting information about the recovery of people with tennis elbow over time.

The allocated intervention was shown here again

Now we would like to ask you a few questions about ***how the information*** is presented.

20. In this presentation, how ***easy*** was it to understand the information presented? (From 1= Not at all easy to 10= extremely easy)
21. How ***satisfied*** are you with **HOW** the information is presented? (From 1= not at all satisfied to 10=I am totally satisfied)
22. **In the future, you may have to make a decision about managing a health condition. To help you make this decision you may like more information about how your health may change over time because of this condition (i.e. its prognosis). You have just seen A (PIPE IN TEXT AS PER GRAPH SHOWN pictograph bar graph, line graph, text) presenting prognostic information.**

For peer review only - http://bmjopen.bmj.com/site/about/guidelines.xhtml

We will now show other ways to you and ask you to rank these ways from the MOST preferred option (1) to the LEAST preferred option (4) for receiving this type of information in future. [SHOW ALL INTERVENTIONS ALONG WITH LABELS]

[ ] Text only
[ ] A bar graph
[ ] A pictograph
[ ] A line graph

23. In a few words, please tell us why you chose the option that you ranked the highest (1)?

24. Do you prefer another way of presenting prognostic information THAT IS NOT IN THE PREVIOUS LIST (e.g., a combination of text and a graph, a table, a combination of a table and a graph)?

Please note that these are just examples and please feel free to suggest any other ways you may prefer.

25. Please rate how much experience you have had with each of the following graphs before today: (Rating from 1= no experience to 5 = a lot of experience) (graphs were shown)

Questions 26 and 27 are the NVS health literacy scale

28. If you had any difficulties with the survey (reading, understanding questions, technical problems) or you have any comments to improve the survey, please note them here

Thank you for your time and for taking part in our survey

For peer review only - http://bmjopen.bmj.com/site/about/guidelines.xhtml

For peer review only

Additional file 2

(Supplementary results)

Comprehension results **2**

Equivalence comparisons of graph types **3**

Association of comprehension with HL, numeracy, and education level

Trial A (Acute otitis media) 4

Trial B (Lateral epicondylitis) 4

Change of decision intention by intervention

Trial A (Acute otitis media) 5

Trial B (Lateral epicondylitis) 7

Reasons for decision intention Q19

Trial A (Acute otitis media) 9

Trial B (Lateral epicondylitis) 12

Reasons for first choice preference Q23

Trial A (Acute otitis media) 16

Trial B (Lateral epicondylitis) 18

Exploratory results

Association of comprehension with MMM, time to complete comprehension question, and graph preference

Trial A (Acute otitis media) 20

Trial B (Lateral epicondylitis) 20

Visual presentation preference by previous graph experience

Trial A (Acute otitis media) 21

Trial B (Lateral epicondylitis) 21

For peer review only

For peer review only - http://bmjopen.bmj.com/site/about/guidelines.xhtml

Comprehension results

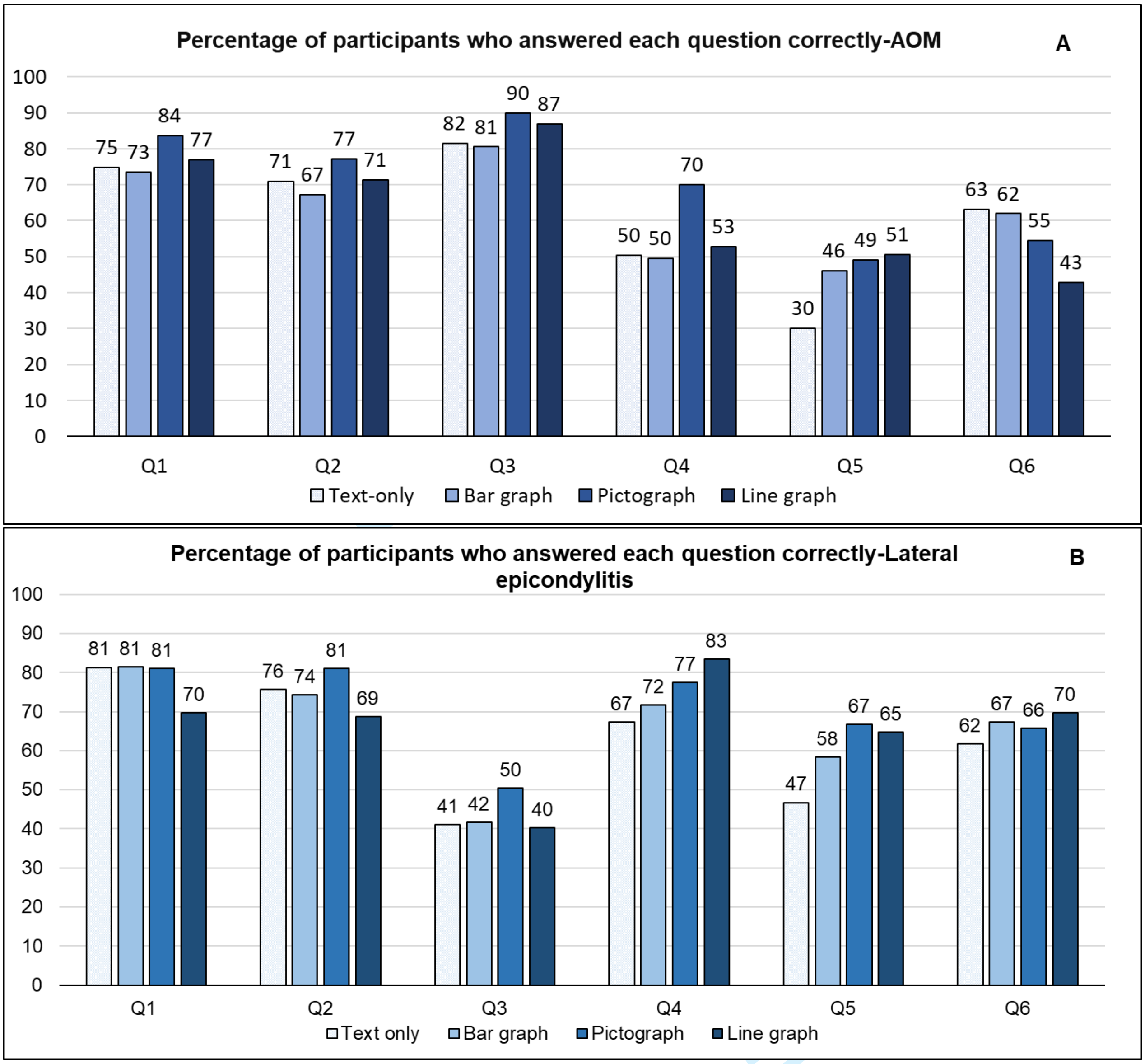

Comprehension of prognostic information. The numbers displayed represent the percentage of participants who answered each question correctly in (A) acute condition-AOM and (B) chronic condition-lateral epicondylitis. Q1-Q6 corresponds to Q12-Q17 in the survey (see additional file 1 for the questions).

Equivalence comparisons of graph types

AOM-Comprehension equivalence comparison between 3 intervention groups

Group 1	Group 2	Difference	95% CI
Pictograph	Bar graph	0.46	0.07 to 0.85
Pictograph	Line graph	0.43	0.03 to 0.84
Line graph	Bar graph	0.03	-0.40 to 0.45

Lateral epicondylitis- Comprehension equivalence comparison between 3 intervention groups

Group 1	Group 2	Difference	95% CI
Pictograph	Bar graph	0.28	-0.14 to 0.70
Pictograph	Line graph	0.26	-0.21 to 0.74
Line graph	Bar graph	0.01	-0.45 to 0.48

For peer review only

For peer review only - http://bmjopen.bmj.com/site/about/guidelines.xhtml

Association of comprehension with HL, numeracy, and education level

Trial A (Acute otitis media)

Variable	R2	Parameter Estimate	P-value	95% Confidence Limits	
Health literacy (categorised)	24%	High vs med 1.19	<.0001	0.86	1.51
		Low vs med -0.78	0.0007	-1.23	-0.33
Education	0%	0.05	0.47	-0.08	0.17
SNS	4%	0.33	<0.0001	0.19	0.48

Trial B (Lateral epicondylitis)

**Variable**	**R2**	**Parameter Estimate**	**P-value**	**95% Confidence Limits**	
**Health literacy (categorised)**	**29%**	**High vs med 1.28**	**<.0001**	**0.94**	**1.61**
		**Low vs med -1.29**	**<0.0001**	**-1.77**	**-0.81**
**Education**	**2%**	**0.21**	**0.0024**	**0.07**	**0.34**
**SNS**	**3%**	**0.3**	**0.0001**	**0.15**	**0.45**

For peer review only

Change of decision intention by intervention[1]

<u>Trial A (Acute otitis media)</u>

Acute condition - Acute otitis media (AOM) - Text only

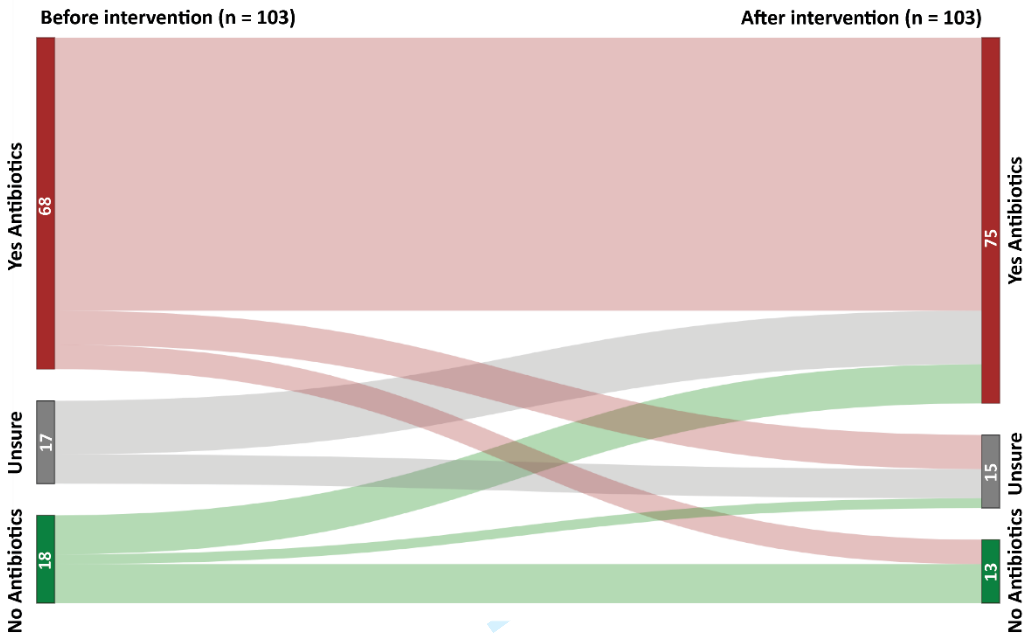

Acute condition - Acute otitis media (AOM) - Pictograph

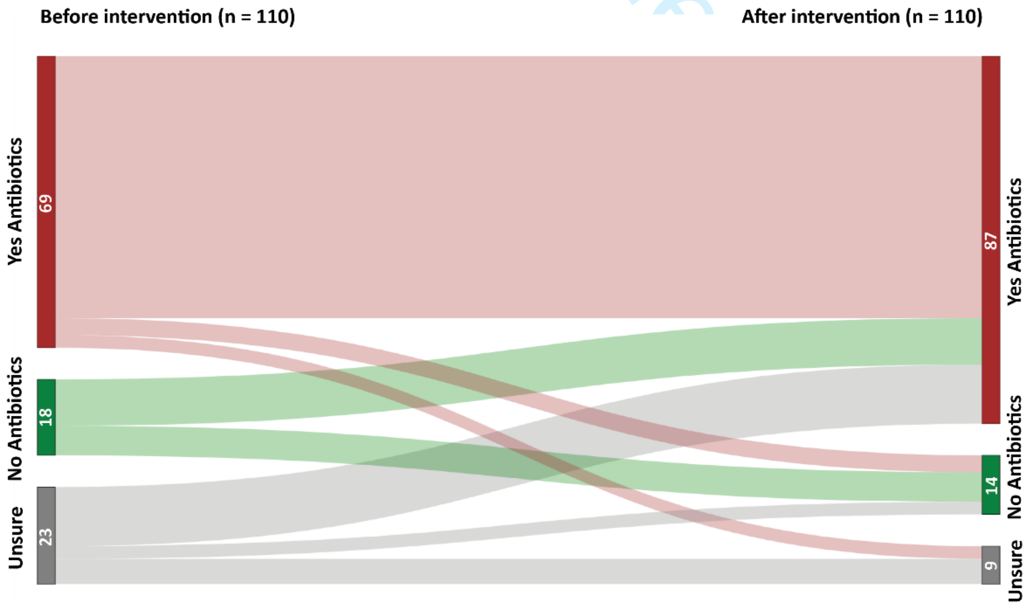

[1] Numbers and percentages of each choice before and after the interventions are presented in table 3 in the main article

For peer review only - http://bmjopen.bmj.com/site/about/guidelines.xhtml

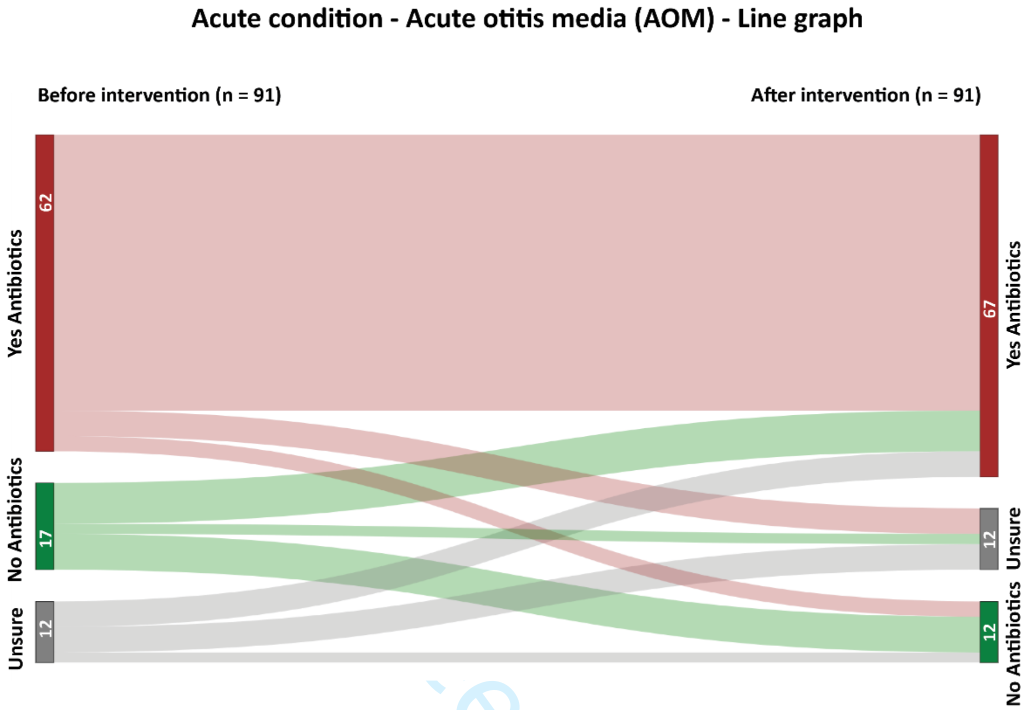

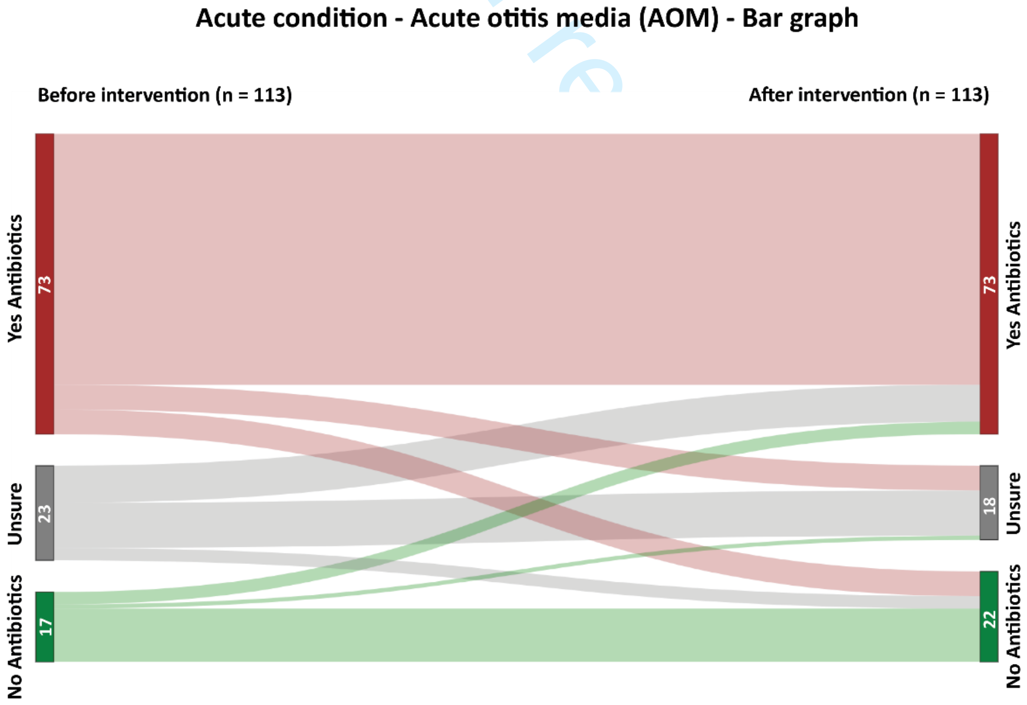

For peer review only - http://bmjopen.bmj.com/site/about/guidelines.xhtml

Trial B (Lateral epicondylitis)

Chronic condition - Lateral epicondylitis - Text only

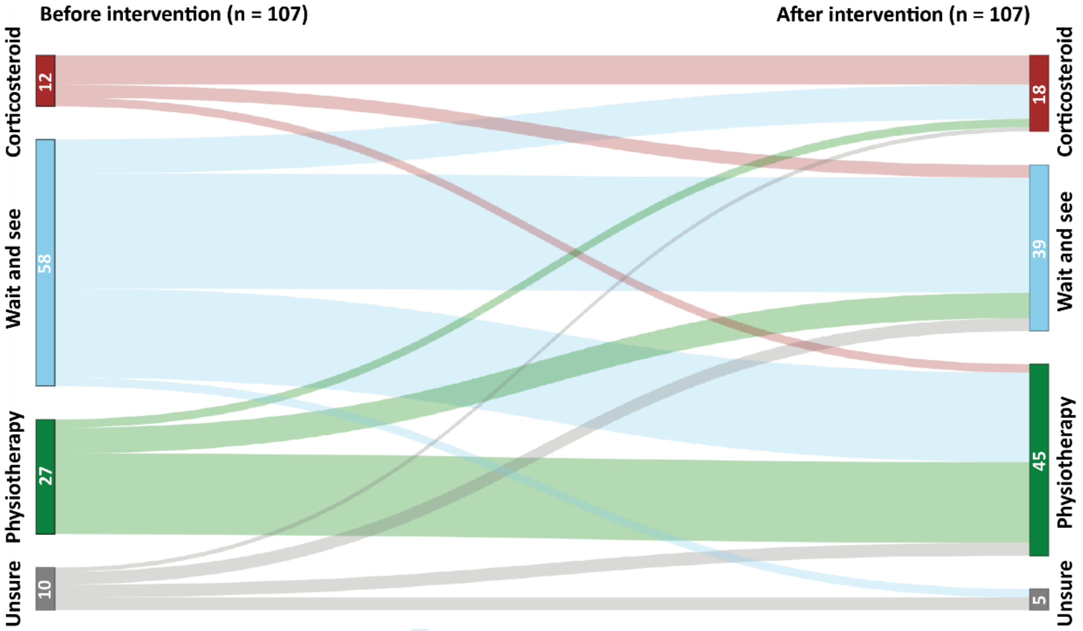

Chronic condition - Lateral epicondylitis - Pictograph

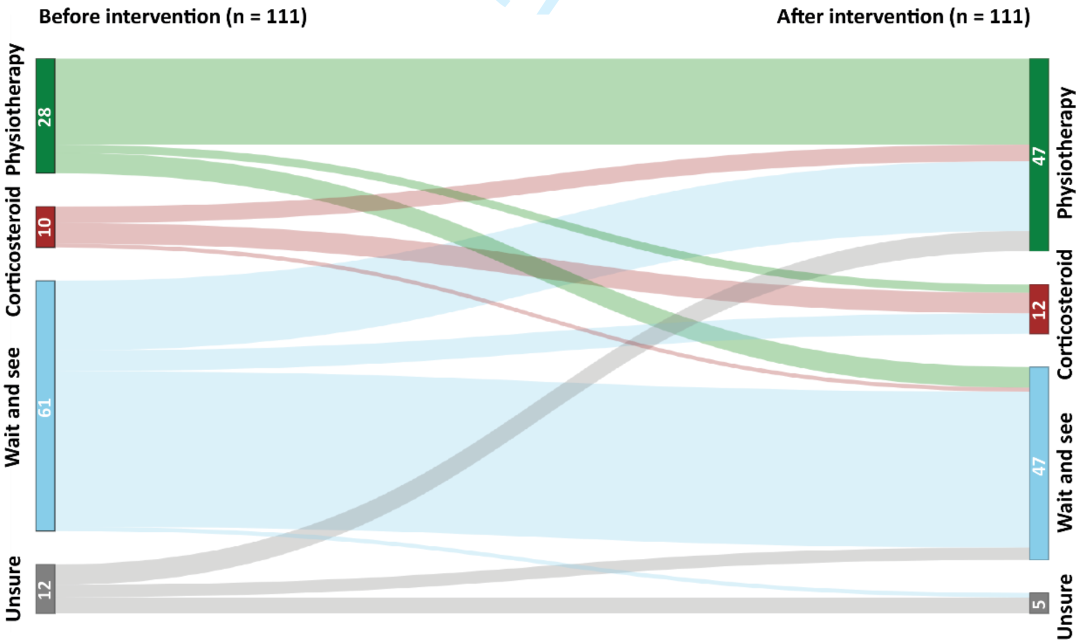

Chronic condition - Lateral epicondylitis - Bar graph

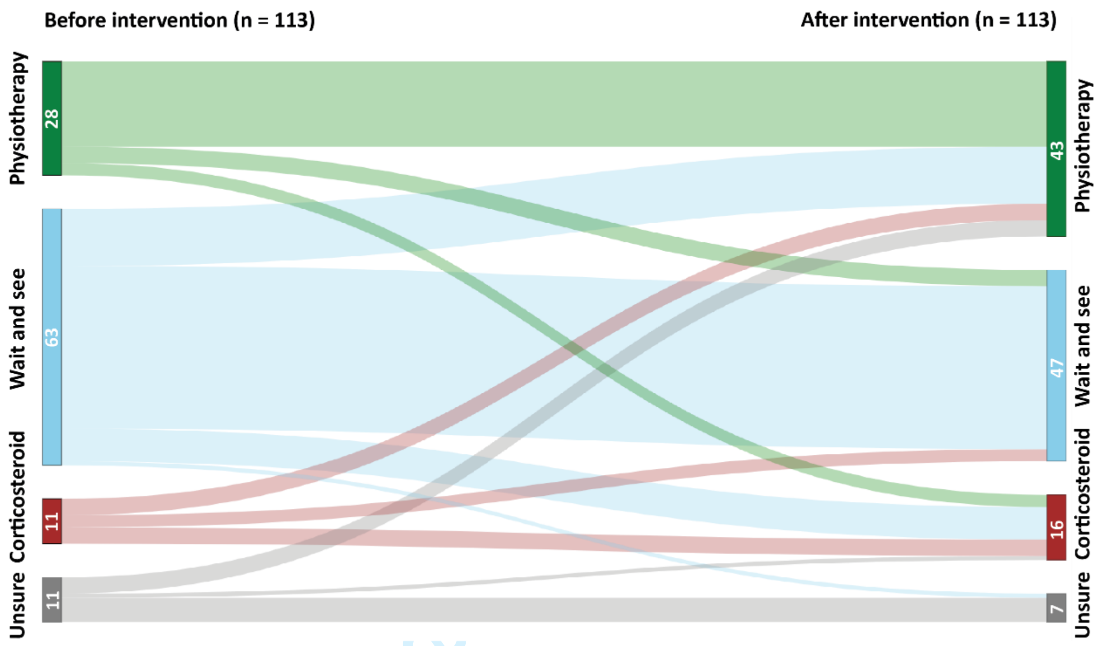

Chronic condition - Lateral epicondylitis - Line graph

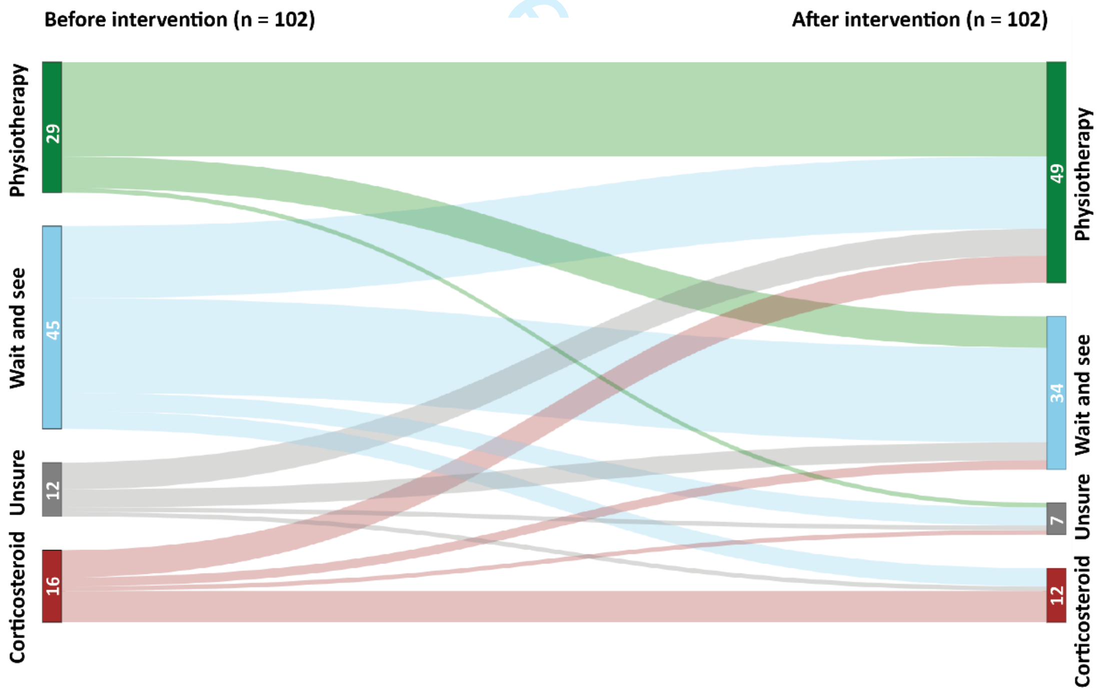

Reasons for decision intention Q19

Trial A (Acute otitis media)

Question 19: Can you please indicate the most important reason for your decision?					
**Reasons of participants who chose to give antibiotics**					
	**Category**	**Description**	**N total**	**Count/ intervention**	**Examples**
1	Based on the presented data	participants who thought data was favouring AB use or referred that their answers were based on the presented data	39	Text-only: 12<br>Bar graph: 9<br>Pictograph: 9<br>Line graph: 9	"the graph indicated that antibiotics has good effects"<br>"data proved fewer children in pain who took antibotics"
2	Antibiotics are effective	Decreased pain<br>Getting better<br>Reduce symptoms	145	Text-only: 33<br>Bar graph: 38<br>Pictograph: 43<br>Line graph: 31	"It helps relieve the pain for the child and fight the infection"<br>"Antibiotics will probably make slow down the infection and also pain."
3	Antibiotics save time	Decrease pain duration, quick recovery from illness, clear the infection faster	45	Text-only: 13<br>Bar graph: 11<br>Pictograph: 11<br>Line graph: 9	"quicker recovery"<br>"Because it helps it get better quickly"<br>"get rid of the pain faster"
4	Antibiotics prevent complications, concerns about complications if antibiotics were not used	Prevent superadded infections, prevent hearing loos, prevent eardrum perforation, prevent recurrence	50	Text-only: 12<br>Bar graph: 12<br>Pictograph: 17<br>Line graph: 9	"to prevent a perforated eardrum"<br>"it is important for your health and to avoid complications"<br>"The child would probably not be able to eliminate the infection themselves and this could possible lead to deafness or even death left untreated."<br>"To ensure that the infection doesnt get worse and permanently affect their hearing"
5	Following medical advice	The GPs decides if I should take antibiotics, or if necessary	14	Text-only: 3<br>Bar graph: 4<br>Pictograph: 4<br>Line graph: 3	"take it only if the doctor says"<br>"I rely on Medical Advice as to how to proceed in situations like this."
6	Based on personal experience or perspectives	Participants who had either negative/positive previous experience with the condition when they used/not antibiotics	21	Text-only: 7<br>Bar graph: 3<br>Pictograph: 4<br>Line graph: 6	"ive been a sufferer of inner ear infections so have my children antis are the only way for us"<br>"have had a child with ear infection, very painful, and always needed antibiotics"
7	Antibiotics are necessary/ needed	Participants who believed antibiotics are needed anyway, e.g., antibiotics work wonder	24	Text-only: 4<br>Bar graph: 5<br>Pictograph: 10<br>Line graph: 5	"you must take antibiotics to stop the infection"<br>"You have to treat an infection."<br>"pain is acute and the ear wont clear itself up"
8	Conditional use of antibiotics	Participants who think they would use antibiotics if pain was severe, or if there is fever, it took longer than expected, if my child is suffering	22	Text-only: 5<br>Bar graph: 5<br>Pictograph: 7<br>Line graph: 5	"Depends on the severity of the case."<br>"if he has fever"

For peer review only - http://bmjopen.bmj.com/site/about/guidelines.xhtml

Reasons of participants who chose not to give antibiotics					
	**Category**	**Description**	**N total**	**Count/ intervention**	**Example**
1	Based on the presented data	participants who thought that the presented data was not favouring antibiotic use	20	Text-only: 5<br>Bar graph: 8<br>Pictograph: 2<br>Line graph: 5	"previous chart showed only small difference"<br>"The statistics don't support antibiotic use for this condition"
2	Based on their preference of other alternatives	Participants who preferred to use alternative options (other medicines, home remedies) or to wait and see if it goes on its own	13	Text-only: 0<br>Bar graph: 5<br>Pictograph: 4<br>Line graph: 4	"In many case home remedy will take care of this problem."<br>"there may be other way to solve this problem"
3	Based on personal preference	Participants who didn't give a specific reason e.g. I like this option	4	Text-only: 2<br>Pictograph: 1<br>Pictograph: 1<br>Line graph: 0	"i think its helpful"<br>"because I like it"
4	Concerns about antibiotic overuse and resistance	Participants who related their decision to the effect of overuse of antibiotics	11	Text-only: 0<br>Bar graph: 7<br>Pictograph: 4<br>Line graph: 0	"not worth becoming immune to antibiotics over a few percentage points."<br>"Because of growing immunity to anibiotics it is always best to avoid them if they are not stricty necessary"
5	Concerns about adverse events	Participants who were worried about the side effects of antibiotics	18	Text-only: 1<br>Bar graph: 8<br>Pictograph: 5<br>Line graph: 4	"Because antibiotics has side effects and most children have self antibody with which he/she can immune himself/herself."<br>"antibiotics can cause allergic reactions in kids"
6	Conditional use of antibiotics	Depends on severity of symptoms, or if prescribed by a GP	12	Text-only: 3<br>Bar graph: 4<br>Pictograph: 4<br>Line graph: 1	"Better not to take antibiotics unless it was really necessary"<br>"wait for doctor decision"
7	Antibiotics have little benefit/ not effective	Participants who thought antibiotics are not of great benefit in this case or in general	11	Text-only: 4<br>Bar graph: 3<br>Pictograph: 1<br>Line graph: 3	"Because generally middle ear infections are viral therefore do not need antibiotics"

For peer review only

For peer review only - http://bmjopen.bmj.com/site/about/guidelines.xhtml

Reasons of participants who were unsure					
	**Category**	**Description**	**N total**	**Count/ intervention**	**Example**
1	Based on the presented data	participants who can't understand the data presented/ need	8	Text-only: 1<br>Bar graph: 4<br>Pictograph: 2<br>Line graph: 1	"very little difference between taking and not taking drugs"<br>"did not understand"<br>"its hard to figure it"
2	Based on their preference of other alternatives	Participants who preferred to use alternative options (other medicines, home remedies), wait and see or having no treatment	6	Text-only: 1<br>Bar graph: 2<br>Pictograph: 2<br>Line graph: 1	"I think it would depend on how severe the infection is, and I would also look for a natural option opposed to anitbiotics"<br>"theres a fine line between the two,,, i prefer to wait a few days and see what eventuates,, not a big fan of antibiotics"
3	Lack of personal knowledge or experience	Participants who had no previous experience of the condition or knowledge about it	5	Text-only: 2<br>Bar graph: 2<br>Pictograph: 0<br>Line graph: 1	"None of my children have ever had a middle ear infection and they are all grown up now"<br>"I do not have children"
4	Concerns about side effects including resistance	Participants who were unsure because they were worried about side effect of using antibiotics	4	Text-only: 0<br>Bar graph: 0<br>Pictograph: 0<br>Line graph: 3	"medication causes the problem"<br>"It will depend on the child's other medical issues and the parents consent. Some children given antibiotics can have serious issues caused by them"
5	Conditional use, depends on severity, …etc	Participants who think using antibiotics depends on individual cases or on specific symptoms or causes	14	Text-only: 5<br>Bar graph: 4<br>Pictograph: 2<br>Line graph: 3	"depends on the history of the indvidual child"<br>"depends if the cause is bacterial"
6	Need more information	Participants who needed more information to make an informed decision	4	Text-only: 1<br>Bar graph: 1<br>Pictograph: 2<br>Line graph: 0	"i can not tell base on just one study"<br>"would need more details how long they have had it , if its reacurring etc"
7	Follow medical advice	Participants who would follow what the doctors say either to take antibiotics or not	9	Text-only: 3<br>Bar graph: 2<br>Pictograph: 0<br>Line graph: 4	"I prefer to consult a doctor first before answering the question."<br>"would consult a doctor"
8	Others	Participants who were unsure, don't know	11	Text-only: 3<br>Bar graph: 5<br>Pictograph: 1<br>Line graph: 2	"I don't know"<br>"Dont have any"

For peer review only - http://bmjopen.bmj.com/site/about/guidelines.xhtml

Trial B (Lateral epicondylitis)

**Q19- Can you please indicate the most important reason for your decision?**					
**Reasons of participants who chose corticosteroid injection**					
	**Category**	**Description**	**N Total**	**Count/ intervention**	**Examples**
1	Based on my understanding of the presented data	Participants who based their answers to their understanding of the presented data	24	Bar graph: 6<br>Pictograph: 5<br>Line graph: 3<br>Text only: 10	"The data shows that it has the best outcome of the three."<br>"Ease them immediate pain, best chance of recovery in the short term"
2	Based on previous experience	Based on the participants own experience with the treatment chosen or negative experience with the alternative treatments either personally or heard about it (e.g., someone they know)	12	Bar graph: 5<br>Pictograph: 3<br>Line graph: 3<br>Text only: 1	"because i have this condition and so far it works best for me"<br>"had previous cortizone injection"
3	Avoiding complications or side effects of alternative options/ against other options or personal preference	Participants who chose corticosteroid injection to avoid any complications that might happen if they choose the other options. either based on previous experience or expectation or as a personal preference	13	Bar graph: 2<br>Pictograph: 4<br>Line graph: 3<br>Text only: 4	"i dont believe in waiting to see if something get better or worse, i prefer this as i have had in the past"<br>"no physio here"
4	Corticosteroid offer a speedy recovery	Patients who chose steroid because it has better outcome on the short term	13	Bar graph: 4<br>Pictograph: 4<br>Line graph: 1<br>Text only: 4	"fast recovery chances"<br>"Time is not on my side, so I would take the speediect option"
5	This treatment is effective	Participants who thought this option would be effective	24	Bar graph: 7<br>Pictograph: 2<br>Line graph: 4<br>Text only: 11	"More people recover by using this method"<br>"to help cure the tennis elbow"
3 irrelevant responses were not categorized					

For peer review only - http://bmjopen.bmj.com/site/about/guidelines.xhtml

Reasons of participants who chose physiotherapy					
	**Category**	**Description**	**N Total**	**Count/intervention**	**Examples**
1	Based on my understanding of the presented data	Participants who based their answers to their understanding of the presented data	96	Bar graph: 20<br>Pictograph: 25<br>Line graph: 23<br>Text only: 28	"As the graphs show Physiotheraphy works"<br>"According to the graph patients getting physiotherapy recovers more quickly."
2	Based on previous experience and personal preference	Based on participants' own experience with the treatment chosen or negative experience with the alternative treatments either personally or heard about it (e.g., someone they know)	33	Bar graph: 10<br>Pictograph: 8<br>Line graph: 9<br>Text only: 6	"had a corticosteroid for my shoulder once willnever get one of those again it hurt all the time"<br>"I had it and it was working great"
3	Avoiding complications of the condition or side effects of alternative options/ against other options	Participants who chose corticosteroid injection to avoid any complications that might happen if they choose the other options either based on previous experience or expectation	13	Bar graph: 2<br>Pictograph: 2<br>Line graph: 3<br>Text only: 6	"drug free"<br>"I am doing something about it rather than waiting and watching and physiotherapy is more natural treatment than injecting myself"
4	A more natural option with less side effects	Participants who thought this option is safer and alleviate the side effects of other options	20	Bar graph: 3<br>Pictograph: 5<br>Line graph: 5<br>Text only: 7	"less invasive and high chance of recovery"<br>"It seems to be the best and least-invasive treatment option"
5	I would follow medical/GP advice	Participants who thought seeking medical advice is the right thing to do	6	Bar graph: 3<br>Pictograph: 0<br>Line graph: 3<br>Text only: 0	"get an expert to help with any pain or swelling"<br>"see a doctor sooner"
6	Physiotherapy is better on the long term	Participants who based their response mainly on the long-term recovery rate	12	Bar graph: 3<br>Pictograph: 2<br>Line graph: 2<br>Text only: 5	"Seems to be the best long term option"<br>"The long-term outcome"
7	Based on both short term and long-term effect	Participants who compared effectiveness both on the short term and long term	12	Bar graph: 1<br>Pictograph: 5<br>Line graph: 4<br>Text only: 2	"Best chance of short and long term recovery."<br>"Physiotherapy had a better long term result compared to the others which had a more of a short term solution"
8	Treatment is effective	Participants who thought this option is effective	94	Bar graph: 15<br>Pictograph: 28<br>Line graph: 27<br>Text only: 24	"I think it would help recover"<br>"Physio includes execises which help recovery and future protection"
9	Physiotherapy provides quicker recovery	Participants who believed physiotherapy will provide faster/ quicker recovery	33	Bar graph: 7<br>Pictograph: 9<br>Line graph: 6<br>Text only: 11	"To treat it quicker"<br>"to recover faster"

For peer review only

Reasons of participants who chose to wait and watch					
	**Category**	**Description**	**N Total**	**Count/intervention**	**Examples**
1	Based on my understanding of the presented data	Participants who based their answers to their understanding of the presented data	45	Text only: 9<br>Bar graph: 14<br>Pictograph: 15<br>Line graph:7	"Because the data shows that time will help heal the condition, and that although physiotherapy helped, doing nothing ultimately has the same outcome."
2	Based on previous experience	Based on the participants own experience with the treatment chosen or negative experience with the alternative treatments either personally or heard about it (e.g., someone they know)	15	Text only: 2<br>Bar graph: 6<br>Pictograph: 3<br>Line graph:4	"previous history and treatment"<br>"never had success with physio and other health issues make corticosteroid injection risky"
3	Avoiding complications of alternative options/ against other options	Participants who chose corticosteroid injection to avoid any complications that might happen if they choose the other options either based on previous experience or expectation	21	Text only: 3<br>Bar graph: 5<br>Pictograph: 8<br>Line graph:5	"I have tennis elbow now, I tend to let it run its course. I don't see the value in Physio vs time, and Cortisone damages the bone"<br>"i dont really like injections and physio is expensive"
4	A more natural option with less side effects	Participants who thought this option is safer and alleviate the side effects of other options	56	Text only: 16<br>Bar graph: 14<br>Pictograph: 14<br>Line graph:12	"safe option"<br>"Don't believe in using drugs if it can be avoided."
5	Long term benefit	Participants who based their response mainly on the long-term recovery rate	16	Text only: 3<br>Bar graph: 8<br>Pictograph: 3<br>Line graph:2	"The long term outcome isn't different enough to make me think that one course of action is much better than another"
6	Treatment is effective	Participants who thought this option is effective	38	Text only: 8<br>Bar graph: 11<br>Pictograph: 11<br>Line graph: 8	"effective"<br>"more chance of recovery"
7	Cost free	Participants who chose this option to avoid costs of treatment either because it is free, their insurance would not cover other treatment expenses or they wouldn't wish to spend money to achieve very minimal better chance recovery with the other options	35	Text only: 6<br>Bar graph: 11<br>Pictograph: 11<br>Line graph:7	"If I can save money not going to the doctor right away, then I'll do it."<br>"This is primarily from a cost perspective as I do not have the optimal health insurance that covers the cost of physiotherapy"

For peer review only - http://bmjopen.bmj.com/site/about/guidelines.xhtml

8	Depends on nature/ severity of the disease	Participants who chose this option as first option but are open to chose different options depending on the severity and time of the condition	17	Text only: 6<br>Bar graph: 5<br>Pictograph: 5<br>Line graph:1	"It would depend on the level of pain/discomfort and functional impairment, but I generally feel I could handle it at home with exercise and possibly analgesics if necessary"
9	Avoidance of medical advice	Either don't trust GPs/ medical advice	11	Text only: 2<br>Bar graph: 3<br>Pictograph: 4<br>Line graph:2	"I don't trust the medical profession that much, always been a wait and see person"<br>"i hate doctors"
10	compared both short term and long-term effects	Participants who compared effectiveness both on the short term and long term	4	Text only: 0<br>Bar graph: 2<br>Pictograph: 1<br>Line graph:1	"Short term you might get better results from the injection but in the long term you just need to let it work out"

**Reasons of participants who were unsure**					
	**Category**	**Description**	**N Total**	**Count/intervention**	**Example**
1	No reason, don't know, unsure	Participants who had no reason for their choice	11	Text only: 1<br>Bar graph: 4<br>Pictograph: 3<br>Line graph:3	"Unsure"<br>"no idea"
2	Conditional choice	Depending on severity, work commitments, cost	8	Text only: 2<br>Bar graph: 3<br>Pictograph: 1<br>Line graph:2	"depen on how much it's pain"<br>"it would depend on situation and if I was working and how much it impacted my life"
3	Previous experience/ lack of personal experience	Based on the participants own experience with the treatment chosen or negative experience with the alternative treatments either personally or heard about it (e.g., someone they know)	2	Text only: 0<br>Bar graph: 0<br>Pictograph: 1<br>Line graph:1	"because I'm unsure how I would react to find out that's what I have."<br>" Hard question... I have bilateral epicondylitis in both arms and have tried all of the above so I know nothing works lol Probably watch and wait"
4	Follow medical advice	Participants who thought seeking medical advice is the right thing to do	3	Text only: 2<br>Bar graph: 0<br>Pictograph: 0<br>Line graph:1	"I would see my doctor and be advised by his medical expertise which way to receive treatment"<br>"would probably consult with my doctor for the right treatment"

For peer review only - http://bmjopen.bmj.com/site/about/guidelines.xhtml

Reasons for first choice preference Q23

Trial A (Acute otitis media)

Q23. In a few words, please tell us why you chose the option that you ranked the highest (1)?		
	**Participants who chose text-only first**	
	**Explanation**	**Count**
0	No reason, Unsure, I don't know	6
1	It is easy to understand, clear, concise or it is just good	84
2	It is better than other presentations and easier to compare the different options in this presentation	25
3	It is my personal preference, or the presentation that I am more familiar with or appeals to me and my understanding the best	14
4	Less effort and time consuming to understand the information	3

	**Participants who chose bar graph first**	
	**Explanation**	**Count**
0	No reason, Unsure, I don't know	2
1	It is easy to understand, clear, concise or it is just good	71
2	It is better than other presentations and easier to compare the different options in this presentation	40
3	It is my personal preference, or the presentation that I am more familiar with or appeals to me and my understanding the best	9
4	Less effort and time consuming to understand the information	4
5	Visually better presented, I can understand the information at a glance. The colours are easy to distinguish	27
6	Better in showing the progress, prognosis, trend over time	0

	**Participants who chose pictograph first**	
	**Explanation**	**Count**
0	No reason, Unsure, I don't know	0
1	It is easy to understand, clear, concise or it is just good	32
2	It is better than other presentations and easier to compare the different options in this presentation	14
3	It is my personal preference, or the presentation that I am more familiar with or appeals to me and my understanding the best	1
4	Less effort and time consuming to understand the information	3
5	Visually better presented, I can understand the information at a glance. The colours are easy to distinguish	15
6	Better in showing the progress, prognosis, trend over time	0

For peer review only - http://bmjopen.bmj.com/site/about/guidelines.xhtml

	Participants who chose line graph first	
	**Explanation**	**Counts**
0	No reason, Unsure, I don't know	3
1	It is easy to understand, clear, concise or it is just good	54
2	It is better than other presentations and easier to compare the different options in this presentation	27
3	It is my personal preference, or the presentation that I am more familiar with or appeals to me and my understanding the best	1
4	Less effort and time consuming to understand the information	5
5	Visually better presented, I can understand the information at a glance. The colours are easy to distinguish	18
6	Better in showing the progress, prognosis, trend over time	11

For peer review only

Trial B (Lateral epicondylitis)

Q23. In a few words, please tell us why you chose the option that you ranked the highest (1)?		
	**Participants who chose text-only first**	
	**Explanation**	**Counts**
0	No reason, Unsure, I don't know	9
1	It is easy to understand, clear, concise or it is just good	67
2	It is better than other presentations and easier to compare the different options in this presentation	54
3	It is my personal preference, or the presentation that I am more familiar with or appeals to me and my understanding the best	16
4	Less effort and time consuming to understand the information	15

	**Participants who chose pictograph first**	
	**Explanation**	**Counts**
0	No reason, Unsure, I don't know	5
1	It is easy to understand, clear, concise or it is just good	25
2	It is better than other presentations and easier to compare the different options in this presentation	24
3	It is my personal preference, or the presentation that I am more familiar with or appeals to me and my understanding the best	3
4	Less effort and time consuming to understand the information	2
5	Visually better presented, I can understand the information at a glance. The colours are easy to distinguish	16
6	Better in showing the progress, prognosis, trend over time	0

	**Participants who chose bar graph first**	
	**Explanation**	**Counts**
0	No reason, Unsure, I don't know	3
1	It is easy to understand, clear, concise or it is just good	55
2	It is better than other presentations and easier to compare the different options in this presentation	68
3	It is my personal preference, or the presentation that I am more familiar with or appeals to me and my understanding the best	6
4	Less effort and time consuming to understand the information	8
5	Visually better presented, I can understand the information at a glance. The colours are easy to distinguish	28
6	Better in showing the progress, prognosis, trend over time	2

For peer review only - http://bmjopen.bmj.com/site/about/guidelines.xhtml

	Participants who chose line graph first	
	**Explanation**	**Counts**
0	No reason, Unsure, I don't know	1
1	It is easy to understand, clear, concise or it is just good	28
2	It is better than other presentations and easier to compare the different options in this presentation	26
3	It is my personal preference, or the presentation that I am more familiar with or appeals to me and my understanding the best	5
4	Less effort and time consuming to understand the information	4
5	Visually better presented, I can understand the information at a glance. The colours are easy to distinguish	2
6	Better in showing the progress, prognosis, trend over time	17

For peer review only

Exploratory results

Association between comprehension and MMM, time to complete comprehension questions, and graph preference

Trial A (Acute otitis media)

Variable	R2	Parameter Estimate	P-value	95% Confidence Limits	Variable
MMM scale (categorised)	2%	High vs med -0.60	0.01	-1.05	-0.14
		Low vs med -0.18	0.3	-0.52	0.16
Time to complete comprehension questions (mins)	2%	0.02	0.0066	0.01	0.04
Graph preference	3%	Bar vs text: 0.63	0.0008	0.27	1
		Picto vs text: 0.69	0.0045	0.21	1.16
		Line vs text: 0.52	0.011	0.12	0.92

Trial B (Lateral epicondylitis)

Variable	R2	Parameter Estimate	P-value	95% Confidence Limits	Variable
MMM scale (categorised)	3%	High vs med -0.81	0.0009	-1.28	-0.33
		Low vs med -0.07	0.71	-0.43	0.29
Time to complete comprehension questions (mins)	1%	0.03	0.053	0	0.05
Graph preference	4%	Bar vs text: 0.63	0.0011	0.25	1
		Picto vs text: 0.65	0.0077	0.17	1.12
		Line vs text: 0.84	0.0006	0.37	1.32

For peer review only - http://bmjopen.bmj.com/site/about/guidelines.xhtml

Overall Visual presentation preference by graph experience

Trial A (Acute otitis media)

Preference	N	Previous experience	N	Mean	Std Dev	Minimum	Maximum
Text only	124.0	Bar graph	124.0	2.9	1.2	1	5
		Pictograph	124.0	2.4	1.2	1	5
		Line graph	124.0	3.2	1.3	1	5
Bar graph	137.0	Bar graph	137.0	3.7	1.2	1	5
		Pictograph	137.0	2.5	1.2	1	5
		Line graph	137.0	3.4	1.2	1	5
Pictograph	58.0	Bar graph	58.0	3.7	1.0	1	5
		Pictograph	58.0	3.6	1.1	1	5
		Line graph	58.0	3.8	0.9	2	5
Line graph	98.0	Bar graph	98.0	3.2	1.2	1	5
		Pictograph	98.0	2.6	1.2	1	5
		Line graph	98.0	4.1	1.1	1	5

Trial B (Lateral epicondylitis)

Preference	N	Previous experience	N	Mean	Std Dev	Minimum	Maximum
Text only	144	Bar graph	144	2.92	1.18	1	5
		Pictograph	144	2.22	1.22	1	5
		Line graph	144	2.89	1.2	1	5
Bar graph	154	Bar graph	154	3.96	1.07	1	5
		Pictograph	154	2.48	1.19	1	5
		Line graph	154	3.51	1.12	1	5
Pictograph	68	Bar graph	68	3.71	1.12	1	5
		Pictograph	68	3.49	1.31	1	5
		Line graph	68	3.78	1.09	1	5
Line graph	67	Bar graph	67	3.64	1.05	1	5
		Pictograph	67	2.63	1.23	1	5
		Line graph	67	4.15	1.09	1	5

For peer review only

For peer review only - http://bmjopen.bmj.com/site/about/guidelines.xhtml

Items to include when reporting a randomized trial in a journal or conference abstract

Item	Description	Reported on line number
Title	Identification of the study as randomized	1-2
Authors *	Contact details for the corresponding author	4-20
Trial design	Description of the trial design (e.g. parallel, cluster, non-inferiority)	40, 42-43
Methods		
Participants	Eligibility criteria for participants and the settings where the data were collected	44-48
Interventions	Interventions intended for each group	49-53
Objective	Specific objective or hypothesis	38-41
Outcome	Clearly defined primary outcome for this report	54
Randomization	How participants were allocated to interventions	In the main article
Blinding (masking)	Whether or not participants, care givers, and those assessing the outcomes were blinded to group assignment	In the main article
Results		
Numbers randomized	Number of participants randomized to each group	45-48
Recruitment	Trial status	completed
Numbers analysed	Number of participants analysed in each group	45-48
Outcome	For the primary outcome, a result for each group and the estimated effect size and its precision	57-63
Harms	Important adverse events or side effects	No harms
Conclusions	General interpretation of the results	64-67
Trial registration	Registration number and name of trial register	68
Funding	Source of funding	92-95

**this item is specific to conference abstracts*

iew only

CONSORT 2010 checklist of information to include when reporting a randomised trial*

Section/Topic	Item No	Checklist item	Reported on page No
**Title and abstract**			
	1a	Identification as a randomised trial in the title	1
	1b	Structured summary of trial design, methods, results, and conclusions (for specific guidance see CONSORT for abstracts)	2-3
**Introduction**			
Background and objectives	2a	Scientific background and explanation of rationale	4-5
	2b	Specific objectives or hypotheses	5
**Methods**			
Trial design	3a	Description of trial design (such as parallel, factorial) including allocation ratio	5
	3b	Important changes to methods after trial commencement (such as eligibility criteria), with reasons	9
Participants	4a	Eligibility criteria for participants	5
	4b	Settings and locations where the data were collected	5
Interventions	5	The interventions for each group with sufficient details to allow replication, including how and when they were actually administered	6-7
Outcomes	6a	Completely defined pre-specified primary and secondary outcome measures, including how and when they were assessed	7-8
	6b	Any changes to trial outcomes after the trial commenced, with reasons	NA
Sample size	7a	How sample size was determined	8
	7b	When applicable, explanation of any interim analyses and stopping guidelines	NA
Randomisation:			
Sequence generation	8a	Method used to generate the random allocation sequence	6
	8b	Type of randomisation; details of any restriction (such as blocking and block size)	6
Allocation concealment mechanism	9	Mechanism used to implement the random allocation sequence (such as sequentially numbered containers), describing any steps taken to conceal the sequence until interventions were assigned	6

For peer review only

Implementation	10	Who generated the random allocation sequence, who enrolled participants, and who assigned participants to interventions	6
Blinding	11a	If done, who was blinded after assignment to interventions (for example, participants, care providers, those assessing outcomes) and how	10
	11b	If relevant, description of the similarity of interventions	6-7
Statistical methods	12a	Statistical methods used to compare groups for primary and secondary outcomes	9
	12b	Methods for additional analyses, such as subgroup analyses and adjusted analyses	9-10
**Results**			
Participant flow (a diagram is strongly recommended)	13a	For each group, the numbers of participants who were randomly assigned, received intended treatment, and were analysed for the primary outcome	Figure 3
	13b	For each group, losses and exclusions after randomisation, together with reasons	Figure 3
Recruitment	14a	Dates defining the periods of recruitment and follow-up	5
	14b	Why the trial ended or was stopped	NA
Baseline data	15	A table showing baseline demographic and clinical characteristics for each group	Table 1
Numbers analysed	16	For each group, number of participants (denominator) included in each analysis and whether the analysis was by original assigned groups	Tables and figures
Outcomes and estimation	17a	For each primary and secondary outcome, results for each group, and the estimated effect size and its precision (such as 95% confidence interval)	10-14, and tables
	17b	For binary outcomes, presentation of both absolute and relative effect sizes is recommended	NA
Ancillary analyses	18	Results of any other analyses performed, including subgroup analyses and adjusted analyses, distinguishing pre-specified from exploratory	Additional file 2
Harms	19	All important harms or unintended effects in each group (for specific guidance see CONSORT for harms)	Ethics application
**Discussion**			
Limitations	20	Trial limitations, addressing sources of potential bias, imprecision, and, if relevant, multiplicity of analyses	14-15
Generalisability	21	Generalisability (external validity, applicability) of the trial findings	15-16

For peer review only

Interpretation	22	Interpretation consistent with results, balancing benefits and harms, and considering other relevant evidence	14-17
**Other information**			
Registration	23	Registration number and name of trial registry	5
Protocol	24	Where the full trial protocol can be accessed, if available	In the registry
Funding	25	Sources of funding and other support (such as supply of drugs), role of funders	18

*We strongly recommend reading this statement in conjunction with the CONSORT 2010 Explanation and Elaboration for important clarifications on all the items. If relevant, we also recommend reading CONSORT extensions for cluster randomised trials, non-inferiority and equivalence trials, non-pharmacological treatments, herbal interventions, and pragmatic trials. Additional extensions are forthcoming: for those and for up to date references relevant to this checklist, see www.consort-statement.org.

For peer review only

BMJ Open

Effect of different visual presentations on the public's comprehension of prognostic information using acute and chronic condition scenarios: Two online randomised controlled trials

Journal:	*BMJ Open*
Manuscript ID	bmjopen-2022-067624.R1
Article Type:	Original research
Date Submitted by the Author:	07-Mar-2023
Complete List of Authors:	Abukmail, Eman; Bond University Institute for Evidence-Based Healthcare, Faculty of Health Sciences and Medicine<br>Bakhit, Mina; Bond University, Centre for research in Evidence-Based Practice<br>Jones, Mark; Bond University<br>Del Mar, Chris; Bond University, Faculty of Health Sciences and Medicne<br>Hoffmann, Tammy; Bond University
**Primary Subject Heading**:	General practice / Family practice
Secondary Subject Heading:	Patient-centred medicine, Communication, Evidence based practice
Keywords:	GENERAL MEDICINE (see Internal Medicine), PRIMARY CARE, INTERNAL MEDICINE, Prognosis

SCHOLARONE™
Manuscripts

I, the Submitting Author has the right to grant and does grant on behalf of all authors of the Work (as defined in the below author licence), an exclusive licence and/or a non-exclusive licence for contributions from authors who are: i) UK Crown employees; ii) where BMJ has agreed a CC-BY licence shall apply, and/or iii) in accordance with the terms applicable for US Federal Government officers or employees acting as part of their official duties; on a worldwide, perpetual, irrevocable, royalty-free basis to BMJ Publishing Group Ltd ("BMJ") its licensees and where the relevant Journal is co-owned by BMJ to the co-owners of the Journal, to publish the Work in this journal and any other BMJ products and to exploit all rights, as set out in our licence.

The Submitting Author accepts and understands that any supply made under these terms is made by BMJ to the Submitting Author unless you are acting as an employee on behalf of your employer or a postgraduate student of an affiliated institution which is paying any applicable article publishing charge ("APC") for Open Access articles. Where the Submitting Author wishes to make the Work available on an Open Access basis (and intends to pay the relevant APC), the terms of reuse of such Open Access shall be governed by a Creative Commons licence – details of these licences and which Creative Commons licence will apply to this Work are set out in our licence referred to above.

Other than as permitted in any relevant BMJ Author's Self Archiving Policies, I confirm this Work has not been accepted for publication elsewhere, is not being considered for publication elsewhere and does not duplicate material already published. I confirm all authors consent to publication of this Work and authorise the granting of this licence.

For peer review only

Effect of different visual presentations on the public's comprehension of prognostic information using acute and chronic condition scenarios: Two online randomised controlled trials

Eman Abukmail, MD, M.Sc.[1], eabukmai@bond.edu.au

Mina Bakhit, MBBCh, PhD[1], mbakhit@bond.edu.au

Mark Jones, PhD[1], majones@bond.edu.au

Chris Del Mar, MD[1], FRACGP, cdelmar@bond.edu.au

Tammy Hoffmann, PhD[1], thoffman@bond.edu.au

[1]Institute for Evidence-Based Healthcare, Faculty of Health Sciences and Medicine, Bond University, Queensland, Australia.

Corresponding author:

Eman Abukmail, MD, M.Sc.

Institute for Evidence-Based Healthcare
Faculty of Health Sciences and Medicine
Bond University
14 University Dr, Robina
QLD, Australia 4229
Email: eabukmai@bond.edu.au

For peer review only - http://bmjopen.bmj.com/site/about/guidelines.xhtml

Manuscript word count: 3455 (Introduction to conclusions, without tables and figure legends)

Abstract word count (without keywords): 300

Tables and Figures: 3 figures and 2 tables, 2 Additional files [Additional file 1 (Supplementary methods), Additional file 2 (Supplementary results)], copy of the registered protocol

References: 35

Keywords:

Health communication, prognosis communication, natural history communication, clinician-patient communication, communication in primary healthcare, visual presentations, formats

For peer review only

Abstract

Objectives

To assess the effectiveness of bar graph, pictograph, and line graph compared to text-only, and to each other for communicating prognosis to the public.

Design Two online 4-arm parallel-group randomised controlled trials. Statistical significance was set at $p<0.016$ to allow for 3-primary comparisons.

Participants and setting Two Australian samples were recruited from members registered at Dynata online survey company. In trial A: 470 participants were randomised to one of the four arms, 417 were included in the analysis. In trial B: 499 were randomised and 433 were analysed.

Interventions In each trial 4 visual presentations were tested: bar graph, pictograph, line graph and text-only. Trial A communicated prognostic information about an acute condition (acute otitis media-AOM) and trial B about a chronic condition (lateral epicondylitis). Both conditions are typically managed in primary care where 'wait and see' is a legitimate option.

Main outcome: Comprehension of information (scored 0-6).

Secondary outcomes: Decision intention, presentation satisfaction and preferences.

Results In both trials, the mean comprehension score was 3.7 for the text-only group. None of the visual presentations were superior to text-only. In trial A, the adjusted mean difference (MD) compared to text-only was: 0.19 (95% CI -0.16 to 0.55) for bar graph, 0.4 (0.04 to 0.76) for pictograph, and 0.06 (-0.32 to 0.44) for line graph. In trial B, the adjusted MD was: 0.1 (-0.27 to 0.47) for bar graph), 0.38 (0.01

For peer review only - http://bmjopen.bmj.com/site/about/guidelines.xhtml

to 0.74) for pictograph, and 0.1 (-0.27 to 0.48) for line graph. Pairwise comparisons between the three graphs showed all were clinically equivalent (95% CIs between -1.0 and 1.0). In both trials, bar graph was the most preferred presentation (chosen by 32.9% of trial A participants and 35.6% in trial B).

Conclusions

Any of the four visual presentations tested may be suitable to use when discussing quantitative prognostic information.

Trial registration (ACTRN12621001305819)

Keywords: prognosis communication, natural history communication, clinician-patient communication, visual presentations, graphs

For peer review only - http://bmjopen.bmj.com/site/about/guidelines.xhtml

Article Summary

Strengths and limitations

- This study is the first to focus on communicating prognostic information about conditions managed in primary care and for which "wait and see" is a legitimate option.
- The study recruited a wide range of participants to ensure inclusivity and diversity of gender, background, age and educational level.
- Baseline comprehension was not tested as it was not possible to ask specific comprehension questions without showing participants the interventions.
- Trial participants did not need prior or current experience with the condition to be eligible, which may have influenced responses to some questions.

For peer review only

Introduction

Providing patients with information on their health can encourage active participation in making decisions.[1] Having a conversation about prognosis can involve communicating quantitative data such as the likelihood of occurrence of an outcome, recurrence, or improvement over time, or the likely duration of the illness. Information about a condition's natural history (that is, the natural course of the condition without treatment) is also encompassed under the umbrella of prognostic information. Understanding prognostic information may help patients to have realistic expectations about the course of their condition and make well-informed decisions about subsequent steps.[2 3]

Communicating quantitative information can be challenging for both clinicians and patients[4] and most research has focused on how to communicate treatment benefits and harms. Several factors may contribute to patient's understanding of the information that is conveyed, including the format used and the framing of it.[5] We conducted a systematic review[6] to evaluate the visual presentations (e.g., a table, graph) that have been used to communicate prognostic information. The review identified only 11 studies and found no significant superior effect on comprehension for any of the visual presentations evaluated in the existing studies. It was unclear due to the lack of existing research if visual presentations would facilitate patients' understanding of the communicated information. Most studies presented prognostic information relevant to decision-making about undertaking screening or treatment for cancer.

Some decisions in primary care, for both acute and chronic conditions, may be enhanced by having prognostic information. Discussing prognostic information using

For peer review only

visual presentations may improve patients' comprehension and facilitate decision-making that accommodates patients' values and preferences. The best ways of visually presenting prognostic information for conditions that are typically managed in primary care have rarely been explored. This study aimed to investigate, using conditions typically seen in primary care, whether graphs improve the comprehension of prognostic information compared to text-only, and if the type of graph matters. Within this study, we conducted two trials (A and B). In trial A, the scenario involved an acute condition (where a decision is needed within a few days), and trial B involved a chronic condition that requires a less urgent decision about how to manage it. Results from both trials informed the generalisability of our results for conditions managed in primary care.

Methods

This study was prospectively registered at the Australian New Zealand Clinical Trials Registry (ACTRN12621001305819, 27/09/2021). CONSORT and CHERRIES reporting statements were followed when applicable (See additional file 1).

Study design

The study consisted of two online 4-arm parallel-group randomised controlled trials. Both trials had the same design and differed only in the condition used; trial A conveyed information about an acute condition (acute otitis media-AOM) and trial B conveyed information about a chronic condition (lateral epicondylitis).

Participants and recruitment

Participants were recruited between the 1st and 12th of October 2021 through an online research company, Dynata (www.dynata.com). Dynata recruited a national representative sample (for age, gender, and education level) using an algorithm-

For peer review only - http://bmjopen.bmj.com/site/about/guidelines.xhtml

based sampling tool and sent the surveys to existing registered members who had previously consented to complete online surveys. At the survey commencement, participants read study information and a statement about their right to withdraw from it at any time. To ensure validity and unique responses, Dynata used a captcha at the start of each survey as well as an IP-digital stamp for each participant.

Participants were compensated with 5.90 AUD by Dynata for survey completion. An invitation to the study was emailed to the participants individually using an automated router. Participants accessed the survey link via their Dynata dashboard. More information about Dynata Australian demographics, sampling, and recruitment is provided in Additional file 1.

Participants were eligible if they lived in Australia, are 18 years old or older, and could read and understand English. Participants did not have to have the condition presented in the scenario. Participants can only participate in either trial A or B.

Randomisation

In each trial, eligible participants were randomly allocated using a computer-generated sequence, generated by Dynata, to 1 of 4 groups (text-only, bar graph, pictograph, line graph).

Trial scenarios and intervention groups

Trial A: the scenario conveyed information about the prognosis of children with AOM. AOM was chosen as it is one of the most common childhood infections[7], with about 50% to 85% of all children experiencing at least one episode.[8] Data came from a Cochrane systematic review.[8]

Trial B: the scenario conveyed information about the recovery of people with lateral epicondylitis who had completely recovered or not at two time points when they

For peer review only - http://bmjopen.bmj.com/site/about/guidelines.xhtml

decided to watch and wait, have physiotherapy, or have a corticosteroid injection. We chose lateral epicondylitis as it is a chronic condition that has a few treatment options, including the option of waiting for the condition to spontaneously improve.[9]

Graphical details of bar graphs and pictographs (e.g., symmetric distribution in pictograph, adding a scale, shape of the icon) were chosen based on best practice principles identified from relevant literature.[10-27] The interventions used are provided in Additional file 1.

Patient and public involvement

We pilot tested the AOM materials in a convenience sample of day-care centre teachers (n= 8) and the lateral epicondylitis materials with university administration staff (n=9). The feedback helped to iteratively refine minor details of the interventions and the clarity of some survey questions.

Before commencing trial recruitment, online piloting of all trial materials was conducted with approximately 10% of the sample size needed for the full trial (trial A, n= 47; trial B, n= 41). Participants were recruited by Dynata and not invited to subsequently participate in the trials. Piloting data were also used to adjust the sample size calculation and test for any technical problems. These data were not included in the trial results.

Data collection and outcome measurement

Our primary outcome was comprehension of the information presented, measured using 6 questions based on previous studies.[12 16 23 27] (see Additional file 1 for questions). The comprehension questions were developed to assess different understanding skills, for example, the ability to extract numbers from the information provided (e.g., At 10-12 days, out of 100 children who took antibiotics, how many

For peer review only - http://bmjopen.bmj.com/site/about/guidelines.xhtml

had pain?), the ability to use the numbers to compare the two options (e.g., Which group was less likely to experience pain at about 4-7 days?), and the ability to make use of the information (e.g., In the week or so after a middle ear infection starts, the majority of children will NOT be in pain, regardless of whether they do or do not take antibiotics). Therefore, we were interested to assess overall comprehension differences across groups rather than differences in comprehension of individual questions. Secondary outcomes were decision intention, satisfaction with the presented format (before revealing all formats), and format preference (after revealing all formats) and measured with a combination of ranking questions, Likert scales, and open-ended questions (Additional file 1). All outcomes were measured once (immediately post-intervention), except for decision intention which was measured pre-and post-intervention (See Additional file 2 for the results of individual questions).

Participants completed a 3-part, 28-question structured survey developed for the trial, based on surveys from relevant research. **Part 1**: five demographic questions, a baseline decision intention question, a question on previous experience with the condition, the medical maximizer-minimizer scale (MMMS assesses patients' preferences for aggressive versus more passive approaches to health care)[28], and a validated subjective numeracy scale (SNS)[29 30]. **Part 2:** each group was presented with information according to their allocated group (e.g., text-only, bar graph, pictograph, or line graph). This was followed by six comprehension questions, two questions about decision intention, and two about satisfaction with the intervention. **Part 3:** All four intervention presentations (text-only, bar graph, pictograph, and line graph) were revealed to participants and visual presentation preference was measured using three questions. Participants rated their previous graphic experience

For peer review only - http://bmjopen.bmj.com/site/about/guidelines.xhtml

on a 1-5 scale. Health literacy level was measured using the Newest Vital Sign (NVS) scale.[31]

The multiple-choice options for the comprehension and decision intention questions were randomised to minimise order bias. Participants were able to see the randomised intervention while answering the questions in Part 2, but once they moved to the next page, they were unable to go back to change their responses. This was to ensure that responses to comprehension questions were not influenced by the other interventions that were revealed in Part 3.

Sample size

In the online piloting, the standard deviation (SD) for the primary outcome was, at most, 1.66 units. Assuming 90% power, 0.016% level of significance (adjusted to account for 3 primary comparisons), the difference between groups of 1 unit, and up to 20% missing data, 97 participants were calculated as required in each group (a total of 388 participants) in each trial.

Data analysis

The primary analysis tested whether comprehension was different between each graphic presentation and the text-only presentation (3 comparisons). Linear regression was used and mean differences with 95% confidence intervals (CI) and p-values calculated. Adjusted analysis was performed to account for any potential important baseline imbalances using linear regression, with adjustment for age group, education level, and health literacy level. Statistical significance was set at p<0.016 to allow for 3 primary comparisons.

To assess the equivalence of the three graphic presentations, 95% confidence intervals for the mean difference between groups were estimated for each of the

For peer review only

For peer review only - http://bmjopen.bmj.com/site/about/guidelines.xhtml

three pairwise comparisons using linear regression. Equivalence was to be concluded if the 95% confidence intervals were between -1.0 and 1.0. We were guided by a previous study that used 10% as a clinically important difference for a similar comprehension outcome measure.[32] Univariable linear regression models were constructed to estimate the association between comprehension as the dependent variable and the independent variables (including health literacy, numeracy, and education level). The measure of association reported is the R^2 value. SAS® OnDemand for Academics 2014 was used to analyse the data.

For the four open-ended questions (e.g., the most important reason for participant's decision to choose one of the management options), two authors (EA, MB) independently categorised the responses. Another author (TH) was consulted when there was disagreement. Each response could be categorised into one or more categories (to a maximum of three).

Post-protocol analysis and modifications from protocol

It was not feasible to analyse the data blinded to group allocation because we needed to identify the control group (text-only) so that the appropriate comparisons could be made.

We estimated the association between comprehension as the dependent variable and time to finish the comprehension questions and previous experience with graphs as independent variables. (Results are provided in the Additional file 2)

Results

Figure 1 shows the CONSORT flow diagram of participants in both trials. Table 1 presents the baseline demographic results. There were no baseline differences

For peer review only - http://bmjopen.bmj.com/site/about/guidelines.xhtml

among the groups in either trial, except for health literacy which was slightly higher in the pictograph group in trial A.

Please insert figure 1 here

Figure 1: CONSORT flow diagrams of participants in Trial A and Trial B.

Table 1: Participants' characteristics

	Trial A				Trial B			
	**Text only (n= 103)**	**Bar graph (n= 113)**	**Pictograph (n= 110)**	**Line graph (n= 91)**	**Text only (n= 107)**	**Bar graph (n= 113)**	**Pictograph (n= 111)**	**Line graph (n= 102)**
**Age (years) n (%)**								
18-24	5 (4.9)	7 (6.2)	7 (6.4)	4 (4.4)	5 (4.7)	6 (5.3)	5 (4.5)	10 (9.8)
25-34	15 (14.6)	17 (15.0)	18 (16.4)	8 (8.8)	17 (15.9)	22 (19.5)	12 (10.8)	15 (14.7)
35-44	14 (13.6)	19 (16.8)	14 (12.7)	18 (19.8)	21 (19.6)	17 (15.0)	25 (22.5)	16 (15.7)
45-54	25 (24.3)	23 (20.4)	30 (27.3)	8 (8.8)	20 (18.7)	23 (20.4)	19 (17.1)	14 (13.7)
55-64	26 (25.2)	24 (21.2)	23 (20.9)	28 (30.8)	24 (22.4)	24 (21.2)	28 (25.2)	21 (20.6)
65+	18 (17.5)	23 (20.4)	18 (16.4)	25 (27.5)	20 (18.7)	21 (18.6)	21 (18.9)	26 (25.5)
Prefer not to say	0 (0.0)	0 (0.0)	0 (0.0)	0 (0.0)	0 (0.0)	0 (0.0)	1 (0.9)	0 (0.0)
**Gender n (%)**								
Male	48 (46.6)	53 (46.9)	49 (44.6)	47 (51.7)	52(48.6)	44 (38.9)	46 (41.4)	57 (55.9)
Female	55 (53.4)	60 (53.1)	60 (54.6)	44 (48.4)	55(51.4)	69 (61.1)	64 (57.7)	45 (44.1)
Others	0 (0.0)	0 (0.0)	1 (0.9)	0 (0.0)	0 (0.0)	0 (0.0)	1 (0.9)	0 (0.0)
**State n (%)**								
QLD	26 (25.2)	31 (27.4)	18 (16.4)	24 (26.4)	23 (21.5)	20 (17.7)	24 (21.6)	22 (21.6)
NSW	31 (30.1)	34 (30.1)	38 (34.6)	33 (36.3)	32 (29.9)	39 (34.5)	41 (36.9)	33 (32.4)
ACT	0 (0.0)	2 (1.8)	3 (2.7)	1 (1.1)	2 (1.9)	3 (2.7)	1 (0.9)	2 (2.0)
NT	1 (1.0)	0 (0.0)	0 (0.0)	0 (0.0)	1 (0.9)	0 (0.0)	1 (0.9)	1 (1.0)
WA	12 (11.7)	7 (6.2)	17 (15.5)	4 (4.4)	8 (7.5)	17 (15.0)	11 (9.9)	10 (9.8)
VIC	22 (21.4)	31 (27.4)	23 (20.9)	23 (25.3)	28 (26.2)	28 (24.8)	21 (18.9)	22 (21.6)
TAS	2 (1.9)	2 (1.8)	1 (1.0)	0 (0.0)	1 (0.9)	2 (1.8)	2 (1.8)	3 (2.9)
SA	9 (8.7)	6 (5.3)	10 (10.0)	6 (6.6)	12 (11.2)	4 (3.5)	10 (9.0)	9 (8.8)
**Educational level n (%)**								
High school Y10	13 (12.6)	19 (16.8)	12 (10.9)	10 (11.0)	12(11.2)	9(8.0)	11(9.9)	12(11.8)
High school Y12	11 (10.7)	20 (17.7)	25 (22.7)	16 (17.6)	14(13.1)	17(15.0)	19(17.1)	13(12.8)
Certificate I-IV, Diploma, or apprenticeship	31 (30.1)	32 (28.3)	32 (29.1)	25 (27.5)	39(36.5)	34(30.1)	35(31.5)	33(32.4)
Undergraduate degree[1]	32 (31.1)	29 (25.7)	28 (25.5)	28 (30.8)	29(27.1)	37(32.7)	33(29.7)	27(26.5)
Postgraduate degree[2]	16 (15.5)	13 (11.5)	13 (11.8)	12 (13.2)	13(12.2)	16(14.2)	13(11.7)	17(16.7)
**SNS Mean[3] (SD)**	4.2 (0.9)	4.2 (1.1)	4.2 (1.0)	4.2 (1.0)	4.1 (1.0)	4.2 (1.1)	4.2 (1.1)	4.2 (1.0)
SNS ability	4.3 (1.2)	4.3 (1.4)	4.3 (1.2)	4.3 (1.2)	4.1 (1.3)	4.1 (1.4)	4.1 (1.5)	4.3 (1.3)
SNS preferences	4.2 (0.8)	4.1 (1.1)	4.2 (0.9)	4.1 (1.0)	4.1 (0.9)	4.2 (1.0)	4.2 (1.1)	4.1 (1.0)
**Health literacy n (%)**								
4-6 adequate	60 (58.3)	77 (68.1)	86 (78.2)	59 (64.8)	68 (63.55)	76 (67.26)	80 (72.07)	67 (65.69)
2-3 possibly limited	38 (36.9)	25 (22.1)	19 (17.3)	24 (26.4)	24 (22.43)	23 (20.35)	16 (14.41)	26 (25.49)
0-1 highly limited	5 (4.9)	11 (9.7)	5 (4.6)	8 (8.8)	15 (14.02)	14 (12.39)	15 (13.51)	9 (8.82)

[1] Bachelor's degree, or equivalent
[2] Masters or Doctoral degree, or equivalent
[3] SNS: is a self-report measure of numeracy and scored as the average rating of 8 items (4 items assessed ability and 4 items assessed preferences) marked (1-6)

For peer review only
For peer review only - http://bmjopen.bmj.com/site/about/guidelines.xhtml

**MMM scale[4] n (%)**								
<4 Minimizer	54 (52.4)	65 (57.5)	61 (55.5)	52 (57.1)	63 (58.9)	65 (57.5)	62 (55.9)	47 (46.1)
=4 Neutral	31 (30.1)	32 (28.3)	31 (28.2)	21 (23.1)	31 (29.0)	27(23.9)	28 (25.2)	34 (33.3)
>4 Maximizer	18 (17.5)	16 (14.2)	18 (16.4)	18 (19.8)	13 (12.2)	21(18.6)	21 (18.9)	21 (20.6)
**Experienced with the condition n (%)**	47 (45.6)	48 (42.5)	50 (45.5)	46 (50.6)	32 (29.9)	46 (40.7)	39 (35.1)	26 (25.5)
**Time (mins) to finish comprehension Qs Median (IQR)**	3.5 (2.6-5.3)	3.5 (2.5-5.1)	3.5 (2.4-5.4)	3.3 (2.3-4.8)	3.4 (2.2-4.6)	3.2 (2.1-4.2)	3.2 (2.6-4.6)	3.1 (2.2-4.6)
**Speaking English at home n (%)**	98 (95.15)	105 (92.92)	101 (91.82)	86 (94.51)	100 (93.5)	101 (89.4)	103 (92.8)	94 (92.2)

Trial A

Comprehension

Figure 2A shows the mean comprehension score for each group. For the percentage of participants, in each group, who correctly answered the comprehension questions, see additional file 2. The total mean score across all questions was 3.71 (SD 1.63), 3.79 (SD 1.54), 3.81 (SD 1.53), and 4.25 (SD 1.40) for text-only, bar graph, line graph, and pictograph groups respectively. Only pictograph group participants had statistically significantly superior comprehension to the text-only group participants, with a mean difference (MD) of 0.54 (95% CI 0.13 to 0.95, *p*-value 0.011). There was no significant difference between the bar graph group and the text-only group or between the line graph group and the text-only group (Table 2). After adjustment for age group, education level, and health literacy: pictograph was not statistically different compared to text-only (MD 0.40, 95% CI 0.04 to 0.76, *p*-value 0.031). Although comprehension was higher in participants in the pictograph group than those in the other two graph groups, the 95% CIs are within the prespecified

[4] Medical Maximizer-Minimizer Scale (MMMS): assesses patients' preferences for aggressive versus more passive approaches to health care, medical maximizers (seek health care even for minor problems), medical minimizers: (avoid medical intervention unless it is necessary)
[5]Percentages were reported to one decimal

For peer review only - http://bmjopen.bmj.com/site/about/guidelines.xhtml

For peer review only

equivalence threshold, suggesting the differences among the three graph groups are not clinically meaningful. (See Additional file 2).

Results reporting the analysis of the association between comprehension and health literacy, education level, and numeracy are provided in the supplementary files (Additional file 2).

	Trial A				Trial B			
	**Text only (n= 103)**	**Bar graph (n= 113)**	**Pictograph (n= 110)**	**Line graph (n= 91)**	**Text only (n= 107)**	**Bar graph (n= 113)**	**Pictograph (n= 111)**	**Line graph (n= 102)**
**Superiority comparison (not adjusted)**								
Mean (SD)	3.71 (1.63)	3.79 (1.54)	4.25 (1.40)	3.81 (1.53)	3.74 (1.60)	3.95 (1.59)	4.23 (1.59)	3.96 (1.89)
MD	Ref	0.08	0.54	0.1	Ref	0.21	0.49	0.22
95% CI	Ref	-0.33 to 0.49	0.13 to 0.95	-0.33 to 0.54	Ref	-0.23 to 0.65	0.04 to 0.93	-0.23 to 0.68
P-value	Ref	0.70	0.011	0.63	Ref	0.35	0.032	0.34
**Superiority comparison (adjusted for age group, level of education and categorised health literacy)**								
MD[1]	Ref	0.19	0.4	0.06	Ref	0.1	0.38	0.1
95% CI	Ref	-0.16 to 0.55	0.04 to 0.76	-0.32 to 0.44	Ref	-0.27 to 0.47	0.01 to 0.74	-0.27 to 0.48
P-value	Ref	0.29	0.031	0.76	Ref	0.59	0.046	0.59
**Decision intention n (%) before the intervention**								
Take antibiotics	68 (66.0)	73 (64.6)	69 (62.7)	62 (68.1)				
Not to take antibiotics	18 (17.5)	17 (15.0)	18 (16.4)	17 (18.7)				
Unsure	17 (16.5)	23 (20.4)	23 (20.9)	12 (13.2)	10 (9.4)	11 (9.7)	12 (10.8)	12 (11.8)
To wait and watch					58 (54.2)	63 (55.8)	61 (55.0)	45 (44.1)
To have physiotherapy					27 (25.2)	28 (24.8)	28 (25.2)	29 (28.4)
To have corticosteroid					12 (11.2)	11 (9.7)	10 (9.0)	16 (15.7)
**Decision intention n (%) after the intervention**								
Take antibiotics	75 (72.8)	73 (64.6)	87 (79.1)	67 (73.6)				
Not to take antibiotics	13 (12.6)	22 (19.5)	14 (12.7)	12 (13.2)				
Unsure	15 (14.6)	18 (15.9)	9 (8.2)	12 (13.2)	5 (4.7)	7 (6.2)	5 (4.5)	7 (6.9)
To wait and watch					39 (36.4)	47 (41.6)	47 (42.3)	34 (33.3)
To have physiotherapy					45 (42.1)	43 (38.1)	47 (42.3)	49 (48.0)
To have corticosteroid					18 (16.8)	16 (14.2)	12 (10.8)	12(11.8)
**Satisfaction mean (SD)**								
How easy (1-10)[2]	7.2 (2.2)	7.3 (2.5)	7.7 (2.0)	7.6 (2.2)	7.8 (1.8)	7.8 (2.2)	8.3 (1.8)	7.5 (2.6)
How satisfied (1-10)[3]	7.0 (2.2)	7.4 (2.4)	7.7 (2.0)	7.6 (2.2)	7.8 (1.9)	8.0 (2.0)	8.3 (1.9)	7.7 (2.4)
**Visual presentation preference- most preferred (1st) N (%)**								
Text only	40 (38.8)	38 (33.6)	25 (22.7)	21 (23.1)	43 (40.2)	40 (35.4)	27 (24.3)	34 (33.3)

Please insert figure 2 here

Figure 2: Mean comprehension score of prognostic information per group in trial A (AOM) and trial B (lateral epicondylitis). Comprehension questions are provided in additional file 1. Trial A and B comprehension questions are different.

For peer review only - http://bmjopen.bmj.com/site/about/guidelines.xhtml

Bar graph	31 (30.1)	43 (38.1)	34 (30.9)	29 (31.9)	40 (37.4)	56 (49.6)	28 (25.2)	30 (29.4)
Pictograph	9 (8.7)	8 (7.1)	34 (30.9)	7 (7.7)	12 (11.2)	8 (7.1)	45 (40.5)	3 (2.9)
Line graph	23 (22.3)	24 (21.2)	17 (15.5)	34 (37.4)	12 (11.2)	9 (8.0)	11 (9.9)	35 (34.3)
**Visual presentation preference- least preferred (4th) N (%)**								
Text only	29 (28.2)	25 (22.1)	47 (42.7)	29 (31.9)	20 (18.7)	30 (26.6)	41 (36.9)	32 (31.4)
Bar graph	10 (9.7)	12 (10.6)	12 (10.9)	15 (16.5)	6 (5.6)	8 (7.1)	9 (8.1)	7 (6.9)
Pictograph	43 (41.8)	56 (49.6)	25 (22.7)	34 (37.4)	41 (38.3)	34 (30.1)	11 (9.9)	45 (44.1)
Line graph	21 (20.4)	20 (17.7)	26 (23.6)	13 (14.3)	40 (37.4)	41 (36.3)	50 (45.1)	18 (17.7)
**Previous graph experience[4] mean (SD)**								
Bar graph (1-5)	3.3 (1.2)	3.3 (1.4)	3.4 (1.2)	3.3 (1.2)	3.6 (1.2)	3.6 (1.2)	3.6 (1.1)	3.3 (1.2)
Pictograph (1-5)	2.7 (1.2)	2.6 (1.3)	2.9 (1.3)	2.4 (1.1)	2.5 (1.4)	2.5 (1.3)	2.9 (1.3)	2.4 (1.2)
Line graph (1-5)	3.6 (1.1)	3.3 (1.3)	3.5 (1.1)	3.7 (1.1)	3.5 (1.2)	3.3 (1.2)	3.4 (1.3)	3.6 (1.3)

Table 2: Superiority comparisons of mean comprehension scores**,** Decision intention, Satisfaction with the presentation, Visual presentation preference in all intervention groups, in Trial A and Trial B.

[1]MD is the mean difference from text only group
[2]1=not at all easy to 10= extremely easy
[3]1= not at all satisfied to 10= I am totally satisfied
[4] 1= No experience to 5= a lot of experience

Decision intention

At baseline, about two-thirds of participants (66.0% in text-only, 64.6% in bar graph, 62.7% in pictograph, 68.1% in line graph) chose the option that a child with AOM should usually take antibiotics. This increased after viewing the intervention in three of the groups to (72.8%, 79.1%, and 73.6%) in text-only, pictograph, and line graph respectively) while in the bar graph group, the percentage remained similar (64.6%). Regardless of the allocated group, more people chose to give the child antibiotics after receiving the prognostic information (n=272,65.2% before; n=302,72.4% after). At least half of each group did not alter their choice, regardless of which intervention they received. (Figure 3A). The most common reason that participants gave for choosing antibiotics was their belief that antibiotics are effective. Other reasons are reported in the supplementary files (Additional file 2).

For peer review only

Satisfaction

Mean scores for ease of use and satisfaction were similar for all formats, with pictograph scores slightly higher (Table 2).

Visual presentation preference

Participants ranked their allocated intervention as the highest, except for participants allocated to the pictograph group who ranked pictograph and bar graph equally best (30.9% of participants). Overall and regardless of the randomised group, the bar graph was the most preferred option; chosen by 137 (32.9%), followed by text-only (n=124, 29.7%). The pictograph was the option least likely to be ranked as the first choice (chosen by 58,13.9%) (Table 2). For the least preferred options and reasons for preferences, see Additional file 2.

Trial B

Comprehension

The difference in comprehension scores between each of the three interventions (pictograph, bar graph, line graph) and text-only was not statistically significant either before or after adjustment for age group, education level, and health literacy (Table 2).

Figure 2B shows the mean comprehension score for each group. For the percentage of participants, in each group, who answered the comprehension questions correctly, see Additional file 2.

Results reporting the analysis of the association between comprehension and health literacy, education level, and numeracy are provided in the supplementary files (Additional file 2).

For peer review only

Decision intention

Overall, before receiving any prognostic information, 52.4% (n=227) of participants intended to wait and see when asked about decision intentions. After receiving the information, 30.0% of those changed their choice to physiotherapy, while 11.0% changed to corticosteroid (Figure 3B). A similar pattern was observed across all four interventions (Table 2). Of the participants who chose physiotherapy (the most common choice after receiving the information, regardless of its type), the most common reasons for doing so were their understanding of the presented information, followed by their belief that this was the most effective option. For other reasons see Additional file 2.

Please insert figure 3 here

Figure 3: Overall change in treatment decision intentions before and after viewing the interventions. The figure shows the number of participants who chose each management option in (A) acute condition-AOM and (B) chronic condition-lateral epicondylitis.

Satisfaction

Mean scores for ease of use and satisfaction were similar for all formats, with pictograph scores slightly higher (Table 2).

Visual presentation preference

Overall, the bar graph was the most preferred option for receiving prognostic information, chosen by 35.6% of participants, followed by 33.3% who ranked text-

For peer review only - http://bmjopen.bmj.com/site/about/guidelines.xhtml

only as their most preferred format. The bar graph was also the highest to be chosen as a second preference (35% of participants). By intervention group, 40.2% of the text-only group, 49.6% of bar graph group, 40.5% of pictograph group, and 34.31% of line graph group ranked their allocated intervention as their most preferred option to receive prognostic information in the future.

Table 2 shows the number and proportion of participants, in each group, who chose each format as their most preferred or least preferred option (other ranking choices are provided in Additional file 2).

Discussion

In our two parallel online randomised controlled trials, none of the interventions (pictograph, bar graph, and line graph) were statistically significantly superior to text-only. There were no clinically meaningful differences in comprehension between the groups that viewed the bar graph, pictograph, or line graph. After all visual presentations were revealed to all participants, the bar graph was the most preferred option for receiving prognostic information in the future; chosen by about one-third of participants in both trials. The type of visual presentation viewed did not appear to influence change in decision intention.

The results from our systematic review found no clear evidence to support the superiority of any particular visual presentation to communicate prognosis, with existing research mostly investigating the communication of long-term prognosis for various types of cancer.[6] In our current trials, using non-cancer conditions and shorter prognostic duration, the difference between the various graph types on comprehension or decision intention was not significant. We found some minor

For peer review only

inconsistencies between participants' preferred graph type and their ratings of satisfaction and ease of use of the various graphs. It appears that no graph type is clearly superior at facilitating comprehension, strongly preferred by participants, and viewed as the easiest to understand. Prior to the widespread use of a graph to convey prognostic information, piloting it with the target audience (ensuring a range of health literacy levels) may help to ensure appropriateness.

Several reviews and primary studies of methods of communicating treatment or screening benefits and harms have recommended adding visual presentations to textual information when communicating quantitative data.[13 22 33 34] For example, a test of three different visual presentations (text-only, fact box, visual aid) for communicating mortality with and without screening for prostate and breast cancer found that visual aids (i.e., a pictograph) generally increased comprehension by 18%.[24] However, in our trials, we found that the pictograph was superior to text-only, but only in trial A (AOM scenario). Reasons for this are unclear and might be explained by more complicated prognostic information, and more treatment options, were presented in trial B (e.g., the prognostic course was more variable at the different time periods).

Visual presentations are often incorporated into patient decision aids, which are tools that can be used to support shared decision-making and contain quantitative information about the benefits and harms of options for managing a condition. The International Patient Decision Aids Standards (IPDAS) collaboration recently reviewed the evidence about using and displaying numbers when designing patient decision aids and recommended adding a visual presentation (e.g., pictographs, bar charts) to communicate quantitative information, without specific recommendation about which type to use.[33]

For peer review only - http://bmjopen.bmj.com/site/about/guidelines.xhtml

Line graphs have traditionally been avoided when communicating with patients as it has been assumed they are too difficult to understand[18], however, our study did not find significantly poor comprehension in participants in the line graph group. These findings suggest line graphs are an appropriate graph that might be used to communicate prognostic information. As showing the trajectory of the condition over time is often very important, a line graph may be best suited to use when there are outcome data for more than two time points, as the equivalent information would need multiple pictographs (one at each time point) and the trajectory less clear.

Communicating quantitative information can be more challenging when multiple management options are available or multiple important outcomes. Further studies are needed to investigate how to best visually, and verbally, communicate more complex prognostic information.

There were potential limitations to these two trials; participants were members of an online survey provider and did not need to have experience with the conditions studied, and only spent about 15-20 minutes engaged in completing the survey. We were not able to assess participants' baseline knowledge as the comprehension questions asked for the exact numbers which we expected to be inapplicable for participants without showing them some information. The impact of prior experience, or lack of, is unclear as previous experience may have influenced decision intention, whereas no experience or personal relevance may have reduced engagement. As the questions about decision intention were hypothetical, motivation to properly read the information presented and consider the decision options may have been low. Additionally, to make a well-informed choice, patients need information about both the benefits and harms of each available treatment option. We did not present harms data as part of the information, and this may have influenced responses to questions

For peer review only - http://bmjopen.bmj.com/site/about/guidelines.xhtml

452 about decision intention. Although we chose two conditions that are typically managed in primary care settings, our findings should be interpreted with caution and might not apply to the communication of other health conditions and might only apply to a setting where participants are to interpret prognostic information by themselves, rather than when the information is presented as part of face-to-face communication.

458 Our trials have several strengths, including testing the visual presentations in two conditions with different trajectories and conducting the trials in parallel. Measures were used to prevent cross contamination between the trials (e.g., participants of trial A were not invited to trial B, and participants of the piloting phase were not invited to participate in any of the trials).

463 **Conclusion**

464 Our findings suggest that, if the information is clearly displayed, any of the tested visual presentations can be used to communicate quantitative prognostic information.

467

468

469 **Ethics approval and consent to participate**

470 This study received ethical approval from Bond University Human Research Ethics Committee (EA00088). All methods in this study were performed in accordance with the Declaration of Helsinki. Informed consent was obtained from all Participants to receive online surveys by Dynata survey company. Additionally, before starting the survey, participants were provided with a formal invitation to participate in the study which included information about the study, and their right to withdraw at any time point with no negative consequences

For peer review only - http://bmjopen.bmj.com/site/about/guidelines.xhtml

Consent for publication

Not applicable

Availability of data and materials

The datasets (raw data) and statistical codes generated during the current study are available in the Open Science Framework repository (OSF).[35]

All data analysed during this study are included in this published article and its supplementary information files. (Additional files 1, 2).

A copy of the registered protocol is provided as a supplementary.

Authors' contributions

EA, TH, and CDM conceived the idea of this study. EA developed the interventions with support from TH, CDM and MB. MB helped in the refinement of pictographs intervention. EA, TH and CDM developed the survey. TH was reviewing and giving feedback on all the steps. Data collection was managed by Dynata under the direction of EA, TH. Data were analysed by EA, MJ, and MB. EA drafted the main manuscript and developed all tables and figures with support and feedback from all authors. All authors reviewed the final version of the manuscript and approved it for submission.

Competing interests

None declared

Funding

For peer review only - http://bmjopen.bmj.com/site/about/guidelines.xhtml

This work was supported by the Centre for Research Excellence in Minimizing Antibiotic Resistance in the Community (CRE-MARC), funded by the Australian National Health and Medical Research Council grant number (1153299).

Acknowledgements

We thank our colleagues at the Institute of Evidence-Based Healthcare (IEBH)-Bond University, for giving their feedback on the interventions and testing the survey during the pre-piloting phase. A special thank you to the childcare teachers who pilot-tested Trial A surveys and to Bond University members who pilot-tested Trial B surveys.

This work is dedicated to the memory of Professor Chris Del Mar, one of my PhD supervisors, who contributed fully to this work (passed away in March 2022).

For peer review only

References

1. Charles C, Gafni A, Whelan T. Shared decision-making in the medical encounter: what does it mean? (or it takes at least two to tango). *Soc Sci Med* 1997;44(5):681-92. doi: 10.1016/s0277-9536(96)00221-3 [published Online First: 1997/03/01]
2. Feemster LC, Curtis JR. "We Understand the Prognosis, but We Live with Our Heads in the Clouds": Understanding Patient and Family Outcome Expectations and Their Influence on Shared Decision Making. *Am J Respir Crit Care Med* 2016;193(3):239-41. doi: 10.1164/rccm.201511-2125ED [published Online First: 2016/02/02]

For peer review only - http://bmjopen.bmj.com/site/about/guidelines.xhtml

3. Hoffmann TC, Del Mar C. Patients' expectations of the benefits and harms of treatments, screening, and tests: a systematic review. *JAMA Intern Med* 2015;175(2):274-86. doi: 10.1001/jamainternmed.2014.6016
4. Paladino J, Lakin JR, Sanders JJ. Communication Strategies for Sharing Prognostic Information With Patients: Beyond Survival Statistics. *JAMA* 2019 doi: 10.1001/jama.2019.11533 [published Online First: 2019/08/16]
5. Feldman-Stewart D, Brundage MD, Zotov V. Further insight into the perception of quantitative information: judgments of gist in treatment decisions. *Med Decis Making* 2007;27(1):34-43. doi: 10.1177/0272989X06297101 [published Online First: 2007/01/24]
6. Abukmail E, Bakhit M, Del Mar C, et al. Effect of different visual presentations on the comprehension of prognostic information: a systematic review. *BMC Med Inform Decis Mak* 2021;21(1):249. doi: 10.1186/s12911-021-01612-9 [published Online First: 2021/08/27]
7. Rovers MM, Zielhuis GA. Otitis media meta-analysis. *Pediatrics* 2004;114(2):508-9; author reply 08-9. doi: 10.1542/peds.114.2.508-a [published Online First: 2004/08/03]
8. Venekamp RP, Sanders SL, Glasziou PP, et al. Antibiotics for acute otitis media in children. *Cochrane Database Syst Rev* 2015(6):CD000219. doi: 10.1002/14651858.CD000219.pub4 [published Online First: 2015/06/24]
9. Bisset L, Beller E, Jull G, et al. Mobilisation with movement and exercise, corticosteroid injection, or wait and see for tennis elbow: randomised trial. *BMJ* 2006;333(7575):939. doi: 10.1136/bmj.38961.584653.AE [published Online First: 2006/10/03]
10. Lipkus IM, Hollands JG. The visual communication of risk. *J Natl Cancer Inst Monogr* 1999(25):149-63. doi: 10.1093/oxfordjournals.jncimonographs.a024191 [published Online First: 2000/06/16]
11. Fortin JM, Hirota LK, Bond BE, et al. Identifying patient preferences for communicating risk estimates: a descriptive pilot study. *BMC Med Inform Decis Mak* 2001;1:2. doi: 10.1186/1472-6947-1-2 [published Online First: 2001/09/08]
12. Armstrong K, Schwartz JS, Fitzgerald G, et al. Effect of framing as gain versus loss on understanding and hypothetical treatment choices: survival and mortality curves. *Med Decis Making* 2002;22(1):76-83. doi: 10.1177/0272989X0202200108 [published Online First: 2002/02/09]
13. Ancker JS, Senathirajah Y, Kukafka R, et al. Design features of graphs in health risk communication: a systematic review. *J Am Med Inform Assoc* 2006;13(6):608-18. doi: 10.1197/jamia.M2115 [published Online First: 2006/08/25]
14. Feldman-Stewart D, Brennenstuhl S, Brundage MD. A purpose-based evaluation of information for patients: an approach to measuring effectiveness. *Patient Educ Couns* 2007;65(3):311-9. doi: 10.1016/j.pec.2006.08.012 [published Online First: 2006/10/04]
15. Zikmund-Fisher BJ, Fagerlin A, Ubel PA. Mortality versus survival graphs: improving temporal consistency in perceptions of treatment effectiveness. *Patient Educ Couns* 2007;66(1):100-7. doi: 10.1016/j.pec.2006.10.013 [published Online First: 2006/12/02]
16. Zikmund-Fisher BJ, Fagerlin A, Ubel PA. Improving understanding of adjuvant therapy options by using simpler risk graphics. *Cancer* 2008;113(12):3382-90. doi: 10.1002/cncr.23959 [published Online First: 2008/11/18]
17. Zikmund-Fisher BJ, Witteman HO, Dickson M, et al. Blocks, ovals, or people? Icon type affects risk perceptions and recall of pictographs. *Med Decis Making* 2014;34(4):443-53. doi: 10.1177/0272989X13511706 [published Online First: 2013/11/20]
18. Carling CL, Kristoffersen DT, Flottorp S, et al. The effect of alternative graphical displays used to present the benefits of antibiotics for sore throat on decisions about whether to seek treatment: a randomized trial. *PLoS Med* 2009;6(8):e1000140. doi: 10.1371/journal.pmed.1000140 [published Online First: 2009/08/27]

For peer review only

For peer review only - http://bmjopen.bmj.com/site/about/guidelines.xhtml

19. Zikmund-Fisher BJ, Fagerlin A, Ubel PA. A demonstration of ''less can be more'' in risk graphics. *Med Decis Making* 2010;30(6):661-71. doi: 10.1177/0272989X10364244 [published Online First: 2010/04/09]

20. Brown SM, Culver JO, Osann KE, et al. Health literacy, numeracy, and interpretation of graphical breast cancer risk estimates. *Patient Educ Couns* 2011;83(1):92-8. doi: 10.1016/j.pec.2010.04.027 [published Online First: 2010/06/18]

21. Trevena LJ, Zikmund-Fisher BJ, Edwards A, et al. Presenting quantitative information about decision outcomes: a risk communication primer for patient decision aid developers. *BMC Med Inform Decis Mak* 2013;13 Suppl 2:S7. doi: 10.1186/1472-6947-13-S2-S7 [published Online First: 2013/01/01]

22. Zipkin DA, Umscheid CA, Keating NL, et al. Evidence-based risk communication: a systematic review. *Ann Intern Med* 2014;161(4):270-80. doi: 10.7326/M14-0295 [published Online First: 2014/08/19]

23. Hamstra DA, Johnson SB, Daignault S, et al. The impact of numeracy on verbatim knowledge of the longitudinal risk for prostate cancer recurrence following radiation therapy. *Med Decis Making* 2015;35(1):27-36. doi: 10.1177/0272989X14551639 [published Online First: 2014/10/04]

24. Petrova D, Garcia-Retamero R, Cokely ET. Understanding the Harms and Benefits of Cancer Screening: A Model of Factors That Shape Informed Decision Making. *Med Decis Making* 2015;35(7):847-58. doi: 10.1177/0272989X15587676 [published Online First: 2015/06/06]

25. Kasper J, van de Roemer A, Pottgen J, et al. A new graphical format to communicate treatment effects to patients-A web-based randomized controlled trial. *Health Expect* 2017;20(4):797-804. doi: 10.1111/hex.12522 [published Online First: 2016/12/17]

26. McDowell M, Gigerenzer G, Wegwarth O, et al. Effect of Tabular and Icon Fact Box Formats on Comprehension of Benefits and Harms of Prostate Cancer Screening: A Randomized Trial. *Med Decis Making* 2019;39(1):41-56. doi: 10.1177/0272989X18818166 [published Online First: 2019/02/26]

27. Brick C, McDowell M, Freeman ALJ. Risk communication in tables versus text: a registered report randomized trial on 'fact boxes'. *R Soc Open Sci* 2020;7(3):190876. doi: 10.1098/rsos.190876 [published Online First: 2020/04/10]

28. Scherer LD, Caverly TJ, Burke J, et al. Development of the Medical Maximizer-Minimizer Scale. *Health Psychol* 2016;35(11):1276-87. doi: 10.1037/hea0000417 [published Online First: 2016/10/18]

29. Fagerlin A, Zikmund-Fisher BJ, Ubel PA, et al. Measuring numeracy without a math test: development of the Subjective Numeracy Scale. *Med Decis Making* 2007;27(5):672-80. doi: 10.1177/0272989X07304449 [published Online First: 2007/07/21]

30. Zikmund-Fisher BJ, Smith DM, Ubel PA, et al. Validation of the Subjective Numeracy Scale: effects of low numeracy on comprehension of risk communications and utility elicitations. *Med Decis Making* 2007;27(5):663-71. doi: 10.1177/0272989X07303824 [published Online First: 2007/07/27]

31. Weiss BD, Mays MZ, Martz W, et al. Quick assessment of literacy in primary care: the newest vital sign. *Ann Fam Med* 2005;3(6):514-22. doi: 10.1370/afm.405 [published Online First: 2005/12/13]

32. Woloshin S, Schwartz LM. Communicating data about the benefits and harms of treatment: a randomized trial. *Ann Intern Med* 2011;155(2):87-96. doi: 10.7326/0003-4819-155-2-201107190-00004 [published Online First: 2011/07/20]

33. Trevena LJ, Bonner C, Okan Y, et al. Current Challenges When Using Numbers in Patient Decision Aids: Advanced Concepts. *Med Decis Making* 2021:272989X21996342. doi: 10.1177/0272989X21996342 [published Online First: 2021/03/05]

For peer review only

For peer review only - http://bmjopen.bmj.com/site/about/guidelines.xhtml

620 34. Garcia-Retamero R, Galesic M. Who profits from visual aids: overcoming challenges in people's understanding of risks [corrected]. *Soc Sci Med* 2010;70(7):1019-25. doi: 10.1016/j.socscimed.2009.11.031 [published Online First: 20100128]

623 35. [dataset] Abukmail E, Hoffmann, T., Bakhit, M., Jones, M., & Del Mar, C. Data from: Effect of different visual presentations on the public's comprehension of prognostic information using acute and chronic condition scenarios: Two online randomised controlled trials. , 2021, October. https://osf.io/p2t9w/?view_only=36b1a232ef444d5fb5241735046e34dd

For peer review only

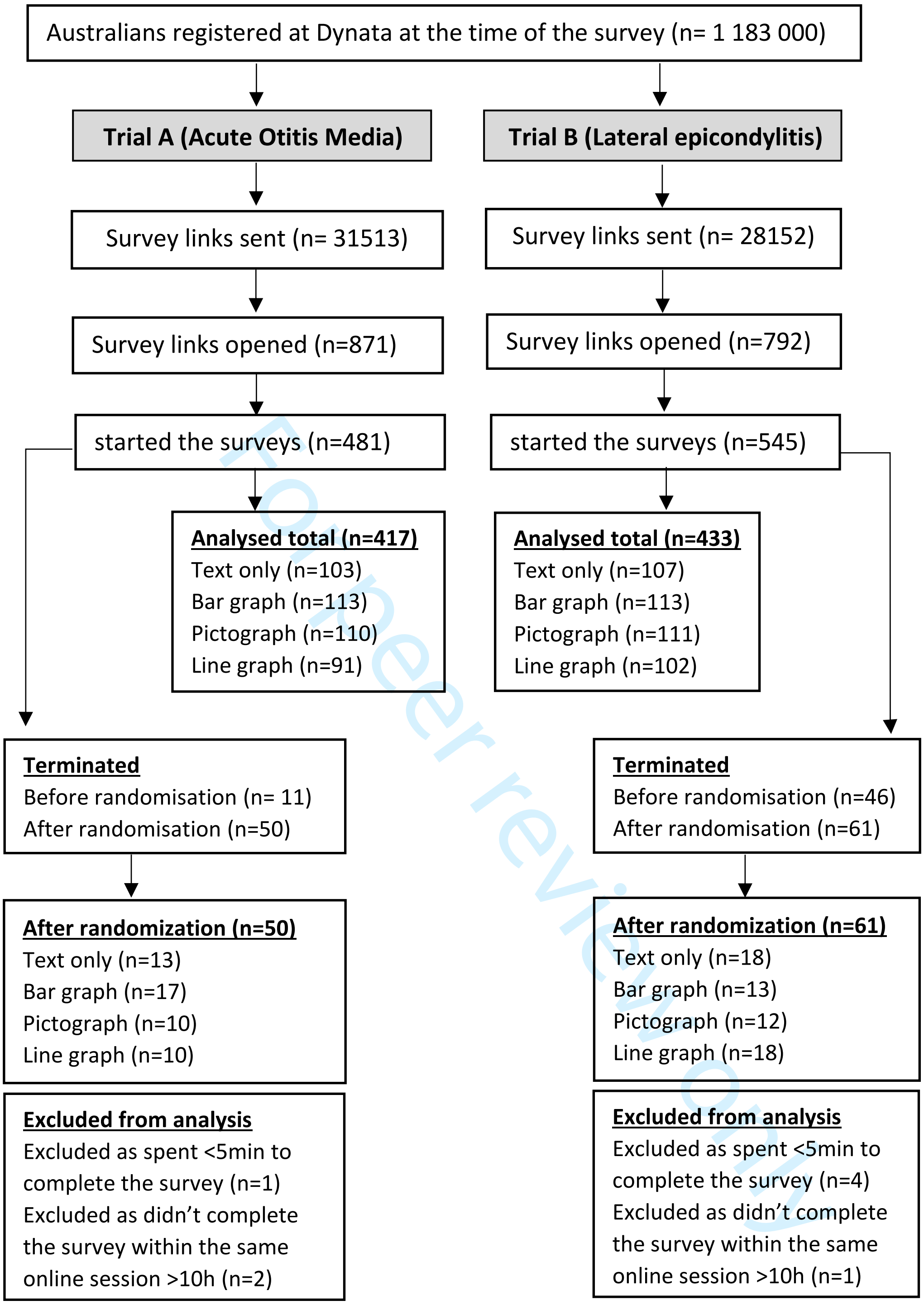

Figure 1: CONSORT flow diagrams of participants in Trial A and Trial B.

For peer review only

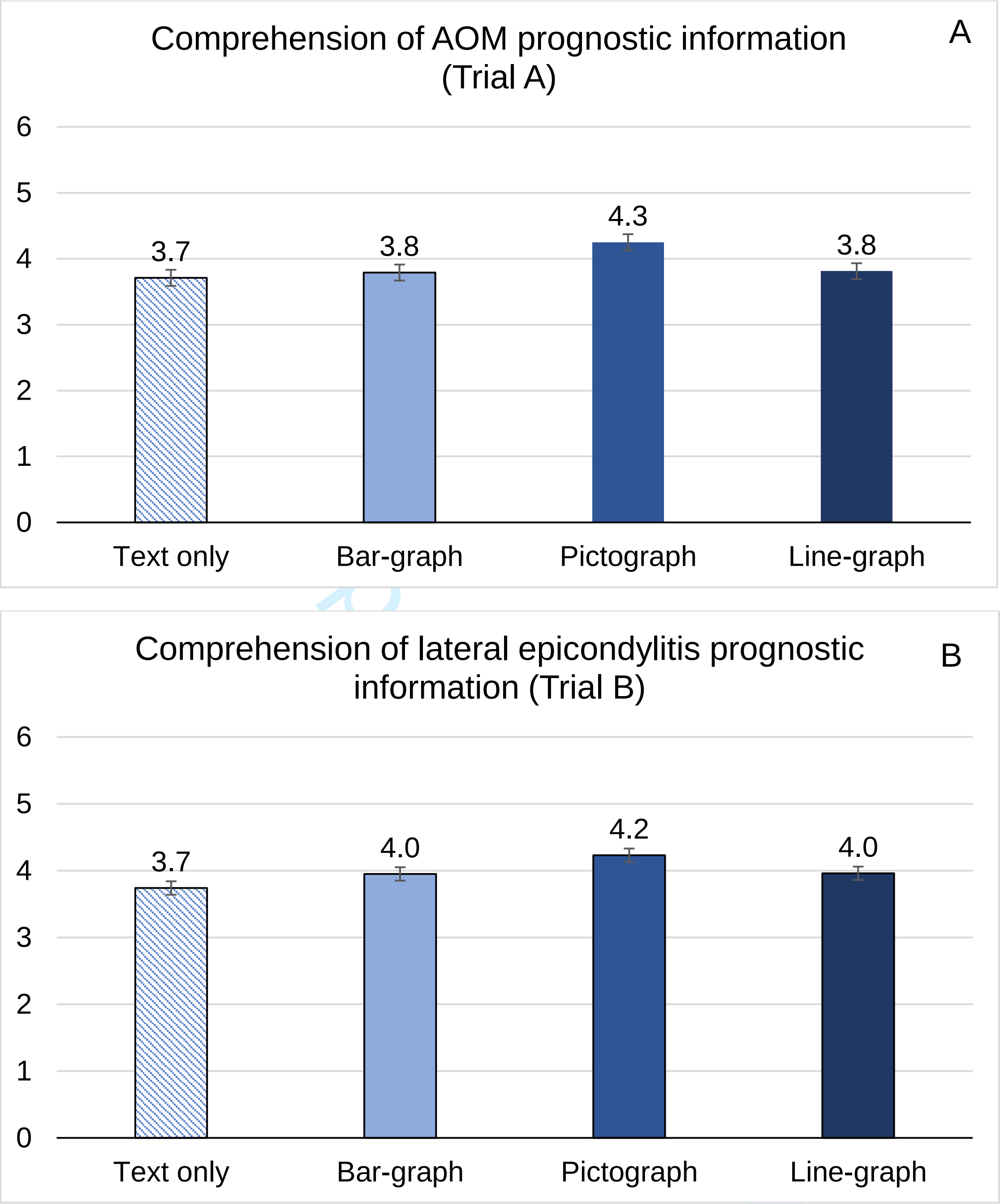

Figure 2: Mean comprehension score of prognostic information per group in trial A (AOM) and trial B (lateral epicondylitis). Comprehension questions are provided in additional file 1. Trial A and B comprehension questions are different.

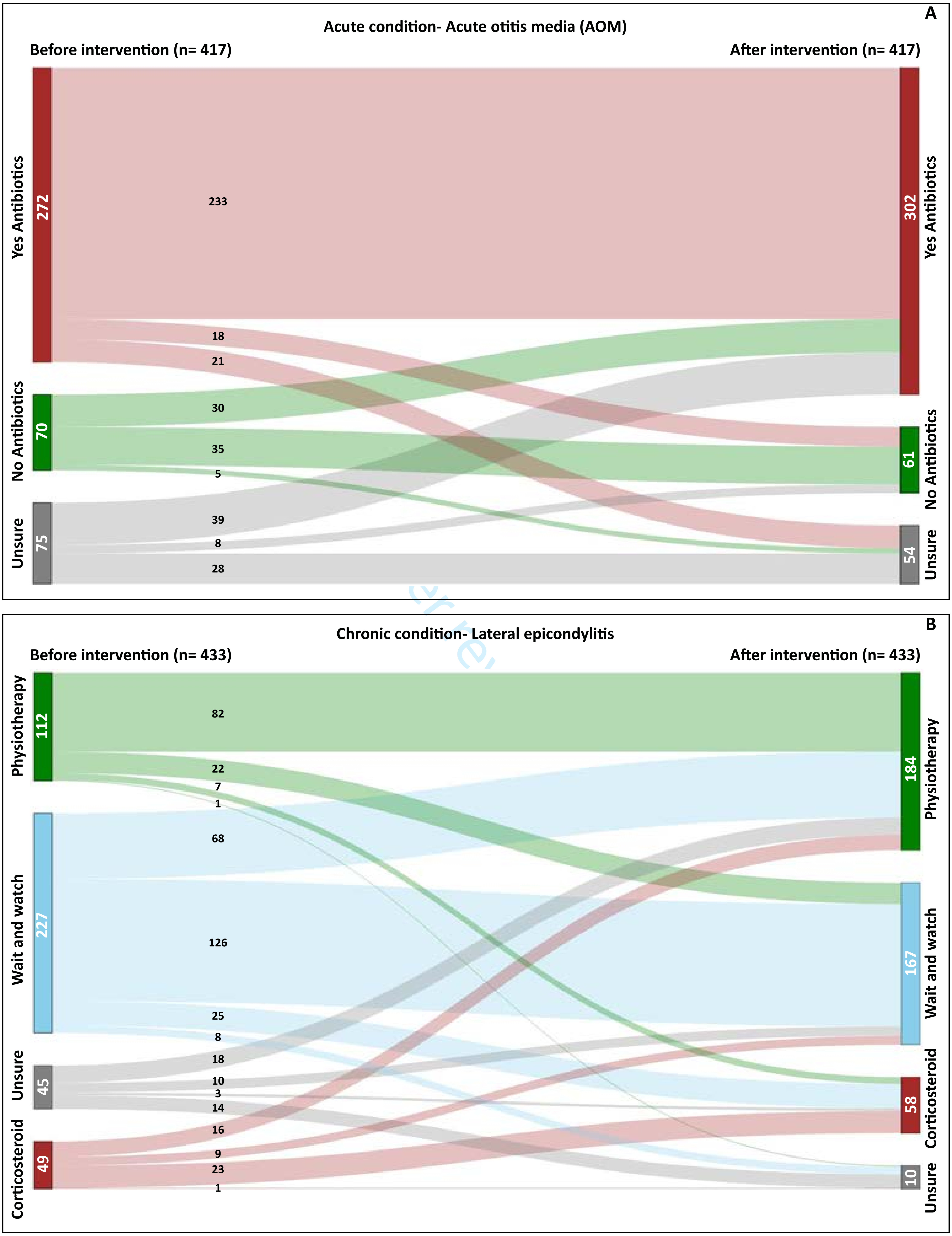

Figure 3: Overall change in treatment decision intentions before and after viewing the interventions.
The figure shows the number of participants who chose each management option in (A) acute condition-AOM and (B) chronic condition-lateral epicondylitis.

Additional file 1

(Supplementary methods)

CONSORT checklist 2

Dynata (demographics in Australia, recruitment strategies) 5

Interventions (visual presentations)

Trial A (Acute otitis media) 6

Trial B (Lateral epicondylitis)........ 9

The flow of the trials and outcome measurements 11

Survey 12

For peer review only

For peer review only - http://bmjopen.bmj.com/site/about/guidelines.xhtml

CONSORT 2010 checklist of information to include when reporting a randomised trial*

Section/Topic	Item No	Checklist item	Reported on page No
**Title and abstract**			
	1a	Identification as a randomised trial in the title	1
	1b	Structured summary of trial design, methods, results, and conclusions (for specific guidance see CONSORT for abstracts)	2-3
**Introduction**			
Background and objectives	2a	Scientific background and explanation of rationale	4-5
	2b	Specific objectives or hypotheses	5
**Methods**			
Trial design	3a	Description of trial design (such as parallel, factorial) including allocation ratio	5
	3b	Important changes to methods after trial commencement (such as eligibility criteria), with reasons	9
Participants	4a	Eligibility criteria for participants	5
	4b	Settings and locations where the data were collected	5
Interventions	5	The interventions for each group with sufficient details to allow replication, including how and when they were actually administered	6-7
Outcomes	6a	Completely defined pre-specified primary and secondary outcome measures, including how and when they were assessed	7-8
	6b	Any changes to trial outcomes after the trial commenced, with reasons	NA
Sample size	7a	How sample size was determined	8
	7b	When applicable, explanation of any interim analyses and stopping guidelines	NA
Randomisation:			
Sequence generation	8a	Method used to generate the random allocation sequence	6
	8b	Type of randomisation; details of any restriction (such as blocking and block size)	6
Allocation concealment mechanism	9	Mechanism used to implement the random allocation sequence (such as sequentially numbered containers), describing any steps taken to conceal the sequence until interventions were assigned	6
Implementation	10	Who generated the random allocation sequence, who enrolled participants, and who assigned participants to interventions	6

For peer review only - http://bmjopen.bmj.com/site/about/guidelines.xhtml

Blinding	11a	If done, who was blinded after assignment to interventions (for example, participants, care providers, those assessing outcomes) and how	10
	11b	If relevant, description of the similarity of interventions	6-7
Statistical methods	12a	Statistical methods used to compare groups for primary and secondary outcomes	9
	12b	Methods for additional analyses, such as subgroup analyses and adjusted analyses	9-10
**Results**			
Participant flow (a diagram is strongly recommended)	13a	For each group, the numbers of participants who were randomly assigned, received intended treatment, and were analysed for the primary outcome	Figure 3
	13b	For each group, losses and exclusions after randomisation, together with reasons	Figure 3
Recruitment	14a	Dates defining the periods of recruitment and follow-up	5
	14b	Why the trial ended or was stopped	NA
Baseline data	15	A table showing baseline demographic and clinical characteristics for each group	Table 1
Numbers analysed	16	For each group, number of participants (denominator) included in each analysis and whether the analysis was by original assigned groups	Tables and figures
Outcomes and estimation	17a	For each primary and secondary outcome, results for each group, and the estimated effect size and its precision (such as 95% confidence interval)	10-14, and tables
	17b	For binary outcomes, presentation of both absolute and relative effect sizes is recommended	NA
Ancillary analyses	18	Results of any other analyses performed, including subgroup analyses and adjusted analyses, distinguishing pre-specified from exploratory	Additional file 2
Harms	19	All important harms or unintended effects in each group (for specific guidance see CONSORT for harms)	Ethics application
**Discussion**			
Limitations	20	Trial limitations, addressing sources of potential bias, imprecision, and, if relevant, multiplicity of analyses	14-15
Generalisability	21	Generalisability (external validity, applicability) of the trial findings	15-16
Interpretation	22	Interpretation consistent with results, balancing benefits and harms, and considering other relevant evidence	14-17
**Other information**			

For peer review only

Registration	23	Registration number and name of trial registry	5
Protocol	24	Where the full trial protocol can be accessed, if available	In the registry
Funding	25	Sources of funding and other support (such as supply of drugs), role of funders	18

*We strongly recommend reading this statement in conjunction with the CONSORT 2010 Explanation and Elaboration for important clarifications on all the items. If relevant, we also recommend reading CONSORT extensions for cluster randomised trials, non-inferiority and equivalence trials, non-pharmacological treatments, herbal interventions, and pragmatic trials. Additional extensions are forthcoming: for those and for up to date references relevant to this checklist, see www.consort-statement.org.

For peer review only

Dynata (demographics in Australia, recruitment strategies)

(Screenshots were taken from the information documents Dynata shared with the researchers at the start of the study and gave permission to share)

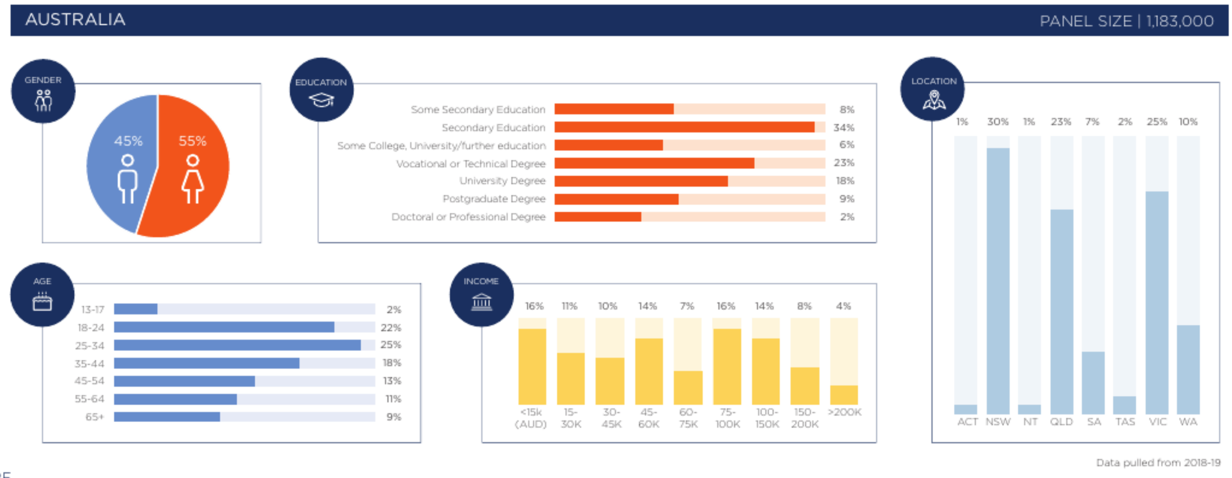

25

Recruitment Methodologies

SOURCE NAME	RECRUITMENT
Loyalty Panels	By invitation to 1000's of loyalty program members in travel, entertainment, media and retail.
Organic, Open Enrolment & Partnerships	1000's of websites, social media influencers and mobile apps.
Affiliate Network	Broad range of websites, incl. schools, communities. Member logging into communities with valid username and password invited to participate.

5

Interventions (visual presentations)

Trial A (Acute otitis media)

Pictograph

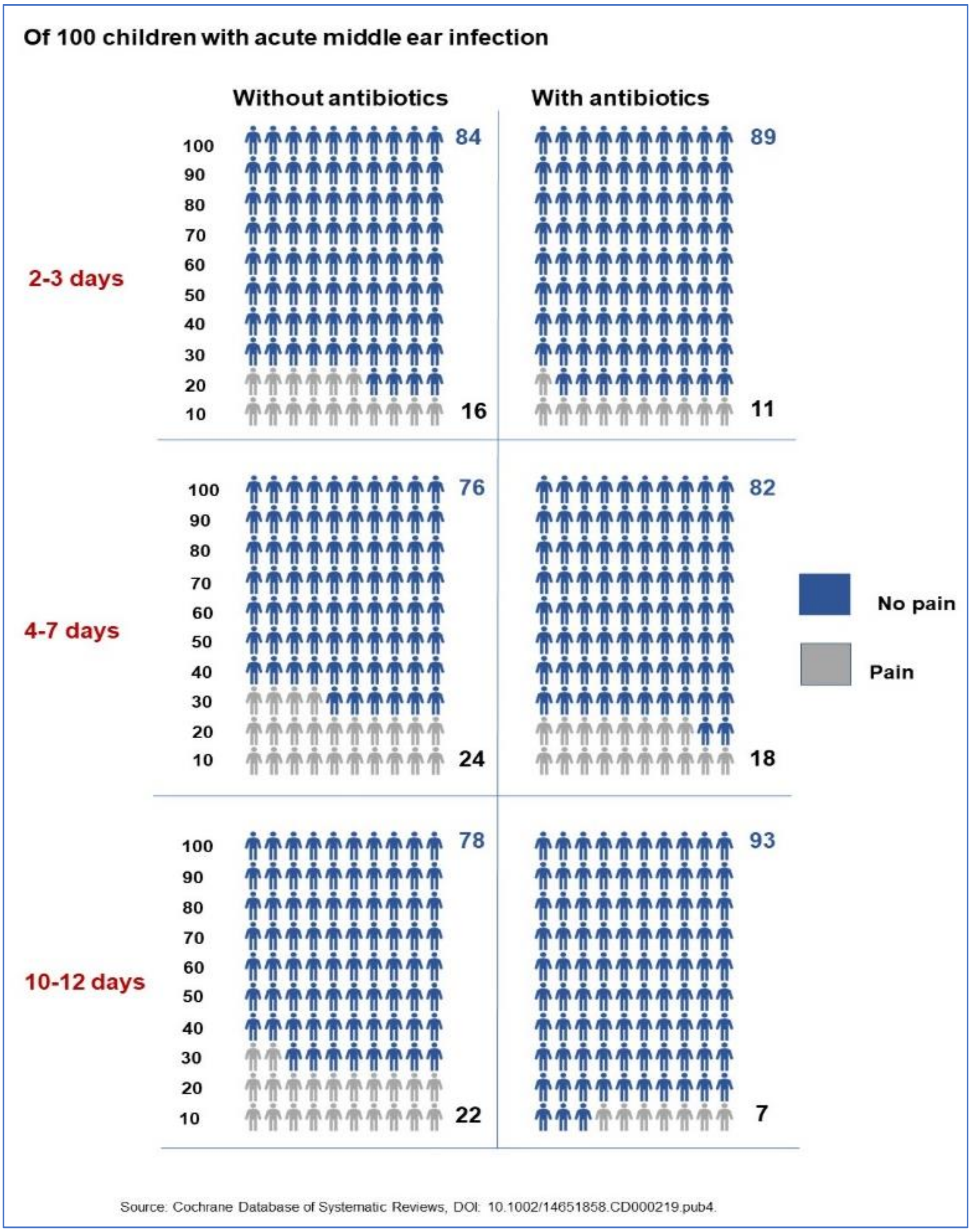

Bar graph

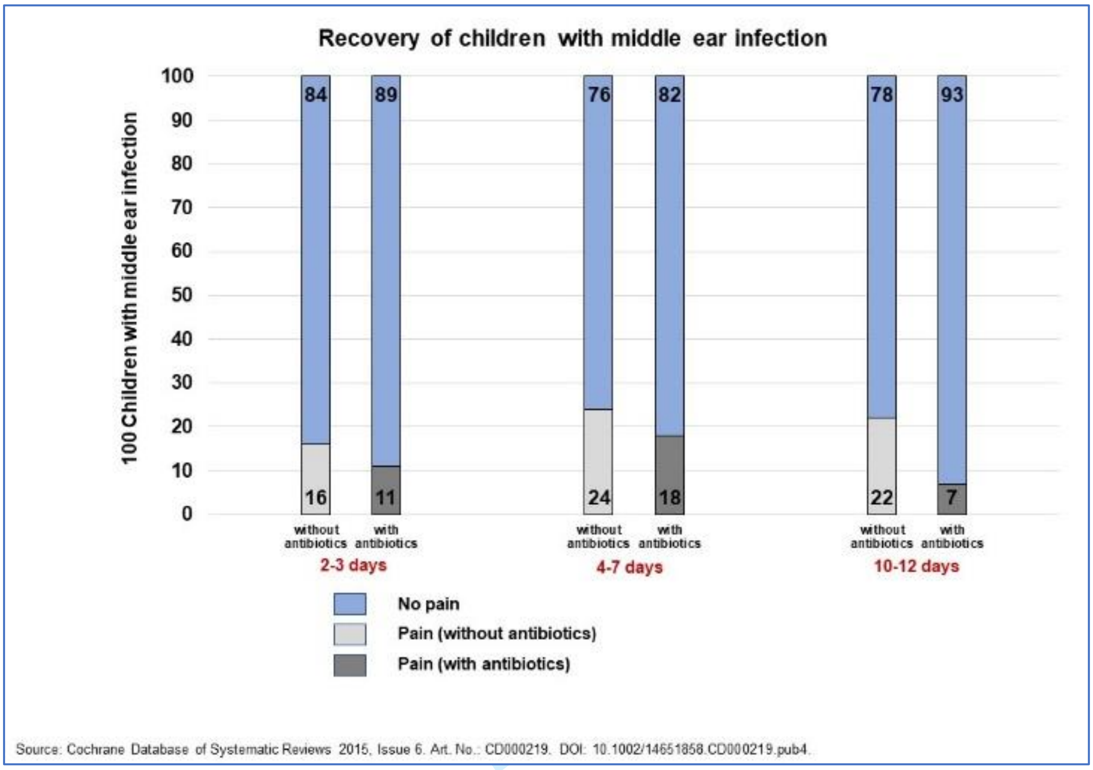

Text-only

Recovery of children with middle ear infection

Of 100 children who have middle ear infection:

Without antibiotic treatment

16 children will still experience pain at **2-3 days** after diagnosis. 24 children will have pain at **4-7 days** and 22 children will have pain at **10-12 days**. The others will have no pain.

With antibiotic treatment

11 children will still experience pain at **2-3 days** after diagnosis, 18 children will have pain at **4-7 days** and 7 children will have pain at **10-12 days**. The others will have no pain.

Source: Cochrane Database of Systematic Reviews 2015, Issue 6. Art. No.: CD000219. DOI: 10.1002/14651858.CD000219.pub4.

For peer review only - http://bmjopen.bmj.com/site/about/guidelines.xhtml

Line graph

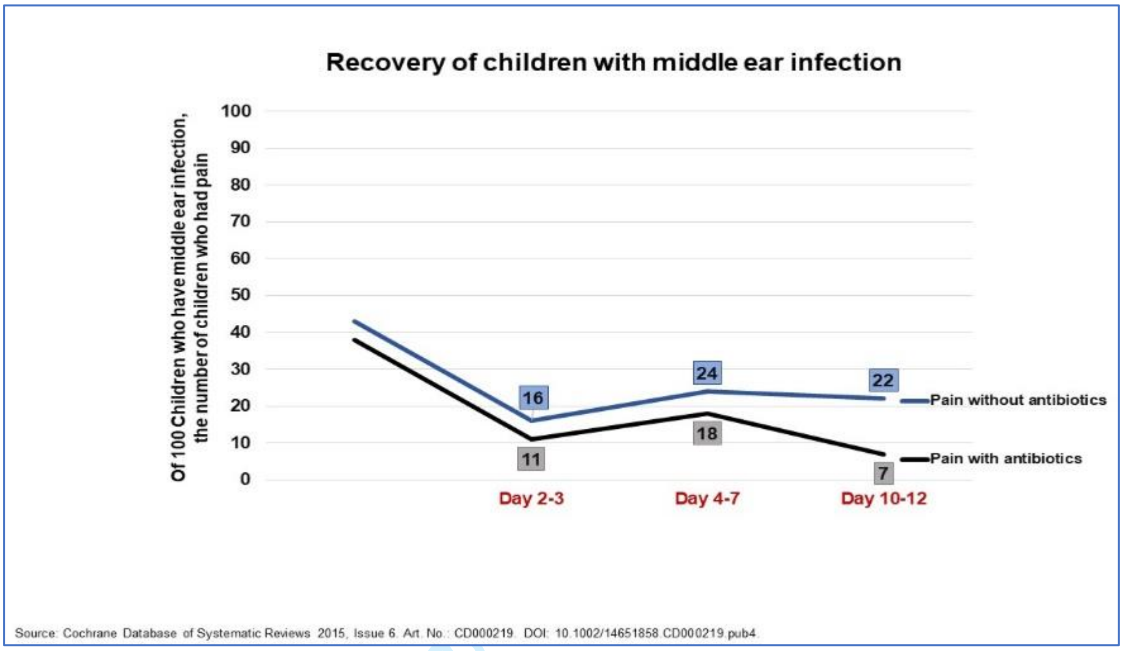

For peer review only - http://bmjopen.bmj.com/site/about/guidelines.xhtml

Trial B (Lateral epicondylitis)

Pictograph

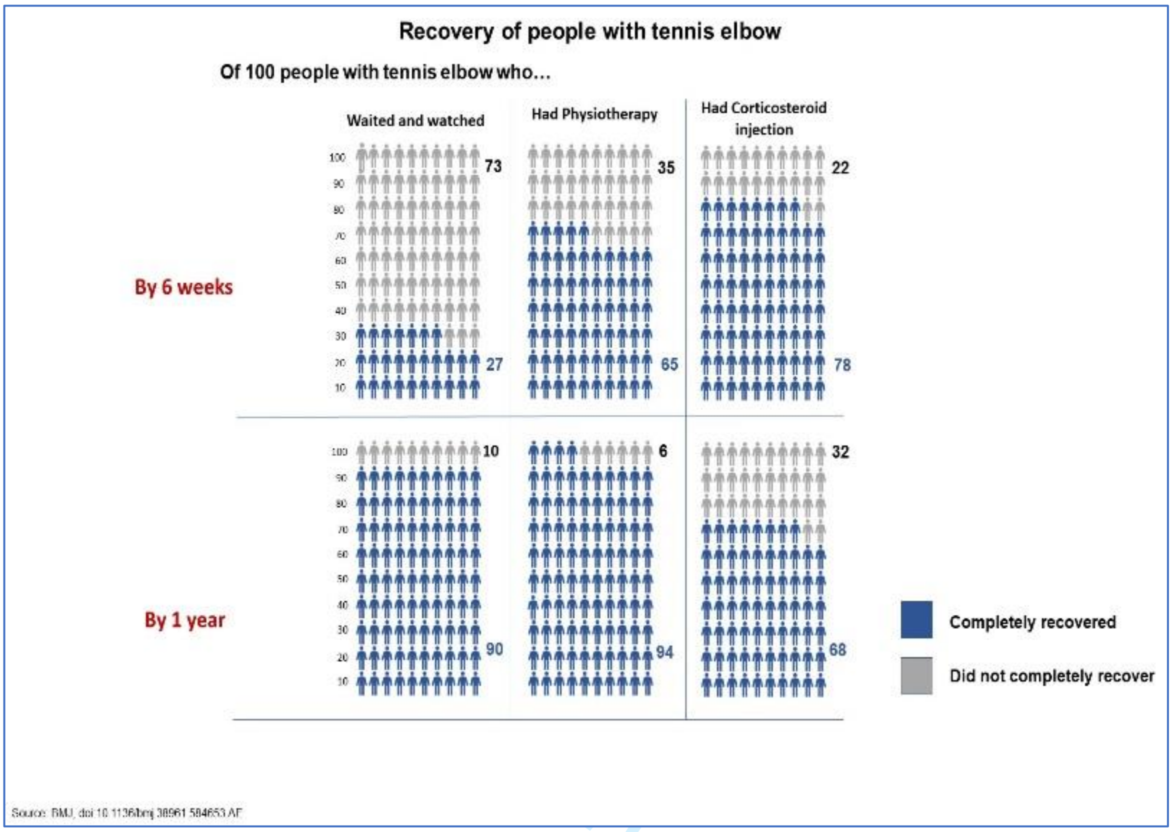

Text-only

Recovery of people with tennis elbow

Of 100 people with tennis elbow who:

Waited and watched (to see if it gets better on its own)

27 people completely recovered **by 6 weeks**. **By 1 year**, 90 people completely recovered; the other 10 did not completely recover.

Had physiotherapy

65 people completely recovered **by 6 weeks**. **By 1 year**, 94 people completely recovered; the other 6 did not completely recover.

Had corticosteroid injection

78 people completely recovered **by 6 weeks**. **By 1 year**, 68 people completely recovered; the other 32 did not completely recover.

Source: BMJ, doi:10.1136/bmj.38961.584653.AE

For peer review only - http://bmjopen.bmj.com/site/about/guidelines.xhtml

Bar graph

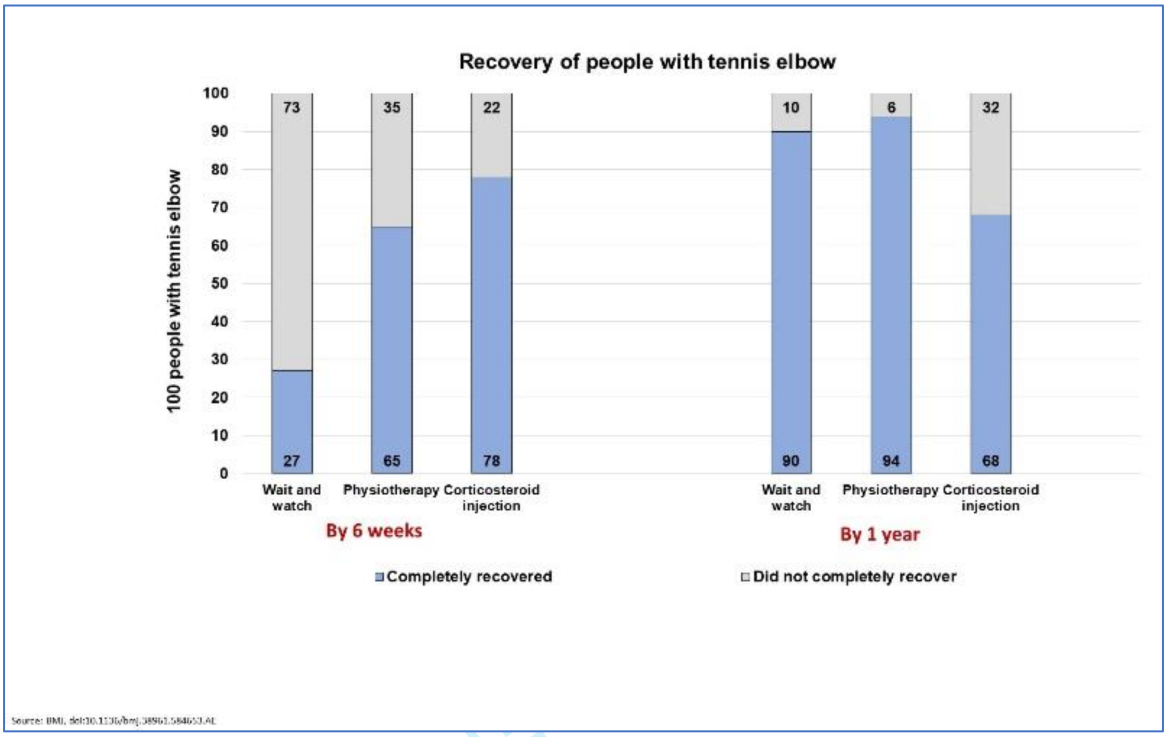

Line graph

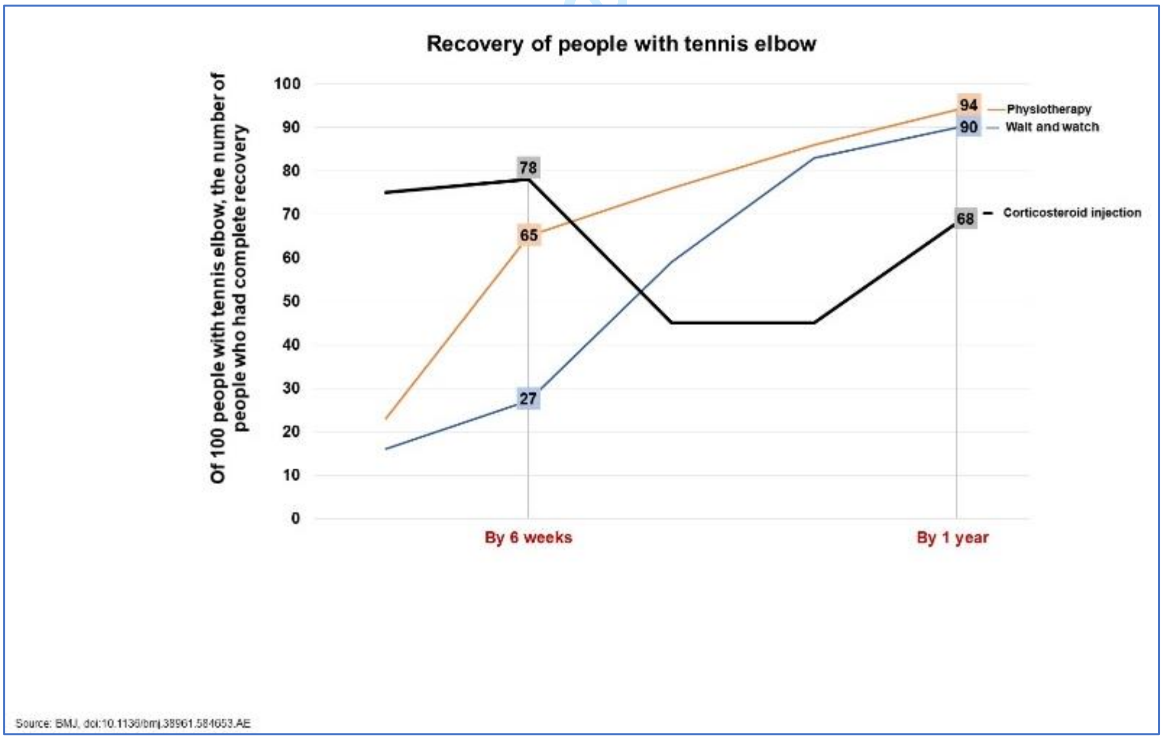

The flow of the trials and outcome measurements

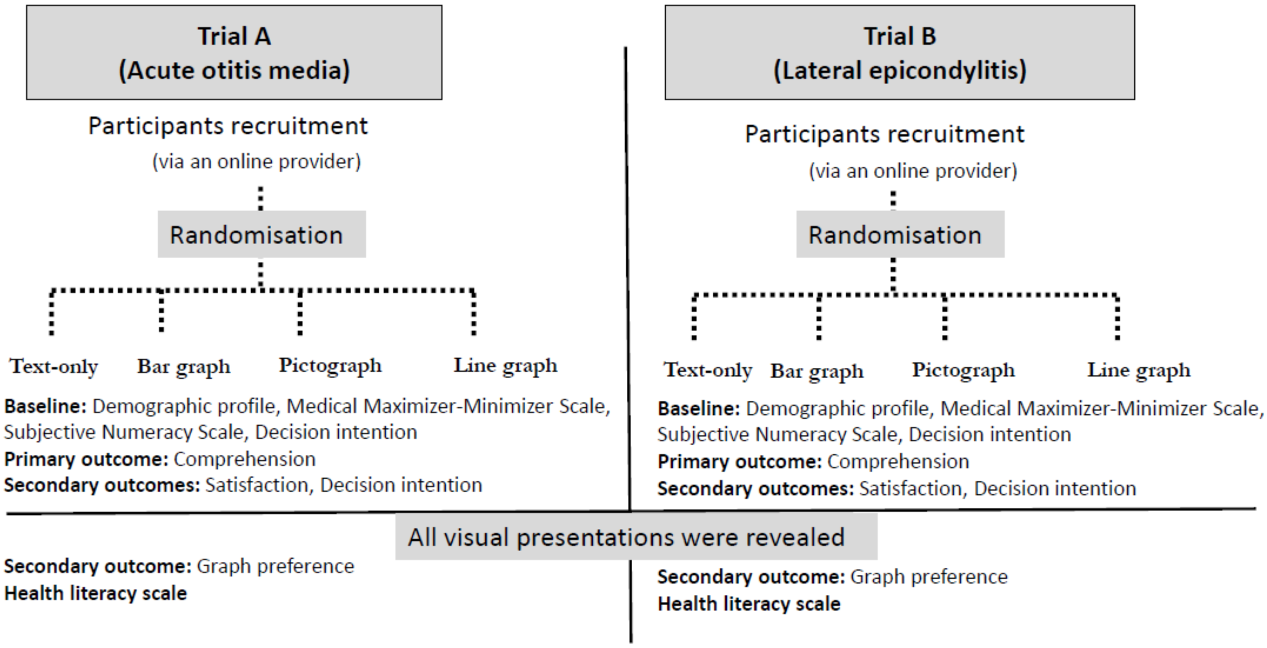

view only

Survey

Questions 1-6 are the same for both trials

1. Which age group applies to you?

[ ] Under 18 [ ] 18 to 24 years [ ] 25 to 34 years
[ ] 35 to 44 years [ ] 45 to 54 years [ ] 55 to 64 years
[ ] 65 years or older [ ] Prefer not to say

2. What is your gender?

[ ] Male [ ] Female
[ ] Other [ ] Prefer not to say

3. What is your level of education? Please indicate the highest level of education you have completed.

[ ] High school - Year 10
[ ] High school – Year 12
[ ] Certificate I-IV, Diploma, or apprenticeship
[ ] Undergraduate degree: Bachelor's degree, or equivalent
[ ] Postgraduate degree: Masters or Doctoral degree, or equivalent

4. Which state or territory do you live in? [PUNCH STATE FROM POSTCODE- OKAY TO MOVE THIS AFTER Q5 IN SCRIPT- DO NOT ASK RESPONDENTS]

[ ] QLD [ ] NSW [ ] ACT [ ] NT [ ] WA [ ] VIC
[ ] TAS [ ] SA

5. What is your postcode? ___________

6. Is English the main language that you speak at home?

[ ] Yes [ ] No

Questions 7 and 8 are specific for each trial

Trial A (Acute otitis media)

7. Acute middle ear infection is an infection behind the eardrum. It is very common, usually in children, and can be very painful. You can take any pain medicine with any of the options below. Imagine that a child you take care of gets a middle ear infection, what decision about using antibiotics might you make?

[ ] The child usually should take antibiotics.
[ ] The child usually should not take antibiotics.
[ ] Unsure

8. Have you, or a person that you are responsible for, previously had an acute middle ear infection?

[ ] Yes
[ ] No

Trial B (lateral epicondylitis)

7. Tennis elbow is a condition in which people have pain and tenderness over the outside of the elbow It usually gets better by itself, regardless of the type of treatment, but symptoms may last up to 2 years[1].

---

[1] Source: Olaussen M, Holmedal O, Lindbaek M, et al Treating lateral epicondylitis with corticosteroid injections or non -electrotherapeutical physiotherapy: a systematic review BMJ Open 2013;3:e003564. doi: 10.1136/bmjopen-2013-003564

For peer review only - http://bmjopen.bmj.com/site/about/guidelines.xhtml

<u>You can take any pain medicine with any of the options below</u>. Imagine that you have tennis elbow, what decision about other treatment options, from the below list, might you make?

[ ] I would probably have a corticosteroid injection.
[ ] I would probably have physiotherapy.
[ ] I would probably wait and watch to see if it gets better on its own.
[ ] Unsure

8. Have you, or a person that you know, previously had tennis elbow (lateral epicondylitis)?

[ ] Yes [ ] No

Questions 9 -11 are the same for both trials (Q9 is MMM scale, Q10 and 11 are Subjective Numeracy Scale)

Comprehension questions Q12-Q17 (These questions are different for trial A and trial B)

Trial A (Acute otitis media) only

Middle ear infection is one of the most frequent diseases in childhood. Children usually experience symptoms of middle ear inflammation like pain, ear discharge, and fever.[2]

The numbers below show what happens over time to children up to 15 years of age with middle ear infection when different ways of managing it are chosen.

The intervention was inserted here

Based on the information in the XXXX (please insert the type of graph e.g., pictograph), please answer the following questions. (Used for all graph interventions)

Based on the information in the text box, please answer the following questions. (Used for all text interventions):

(Q12-Q17- ON SAME PAGE ALONG WITH THE INTERVENTION).

12. At 2-3 days, out of 100 children who did NOT take antibiotics, how many had pain?

Answer: ___________

13. At 10-12 days, out of 100 children who took antibiotics, how many had pain?

Answer: ___________

14. Which group was less likely to experience pain at about 4-7 days?

[ ] Children who took antibiotics
[ ] Children who did not take antibiotics.
[ ] No difference between the groups.
[ ] I do not know.

15. At 4-7 days, how many more children who did NOT take antibiotics will have pain, compared to those who took antibiotics?

Answer: ___________

[2] Source: Cochrane Database of Systematic Reviews 2015, Issue 6. Art. No.: CD000219. DOI: 10.1002/14651858.CD000219.pub4.

For peer review only - http://bmjopen.bmj.com/site/about/guidelines.xhtml

16. At 2-3 days, how many more/fewer children who did NOT take antibiotics will be pain-free, compared to children who did take antibiotics?

[ ] 5 fewer [ ] 5 more [ ] 16 more [ ] 11 fewer

17. In the week or so after a middle ear infection starts, the majority of children will NOT be in pain, regardless of whether they do or do not take antibiotics

[ ] True [ ] False

Trial B (Lateral epicondylitis) only

Tennis elbow is a condition in which people have pain and tenderness over the outside of the elbow. This can make some movements of the arm and wrist painful.

The numbers below show what happens over time to people with tennis elbow when different ways of managing it are chosen.

The intervention was inserted here

Based on the information in the XXXX (please insert the type of graph e.g., pictograph), please answer the following questions. (Used for all graph interventions)

Based on the information in the text box, please answer the following questions. (Used for all text interventions):

(Q12-Q17- ON SAME PAGE ALONG WITH THE INTERVENTION)

12. Of 100 people with tennis elbow who chose to 'wait and watch, how many people completely recovered by 6 weeks?

Answer: ___________

13. Of 100 people with tennis elbow who chose to have a corticosteroid injection, how many people completely recovered by 6 weeks?

Answer: ___________

14. How many <u>more</u> people who chose physiotherapy completely recovered by 6 weeks, compared to those who waited and watched?

Answer: ___________

15. Which group was <u>more likely</u> to be completely recovered by 1 year?

[ ] People who waited and watched
[ ] People who had physiotherapy
[ ] People who had a corticosteroid injection
[ ] No difference between the groups
[ ] I do not know

16. **By 1 year, how many more/ fewer people who had physiotherapy completely recovered, compared to people who had a corticosteroid injection?**

[ ] 26 fewer [ ] 26 more [ ] 32 more [ ] 6 fewer

17. **By 1 year, the majority of people who chose to wait and watch will be completely recovered, without having physiotherapy or a corticosteroid injection.**

[ ] True [ ] False

For peer review only - http://bmjopen.bmj.com/site/about/guidelines.xhtml

Question 18 is trial specific

Trial A

18. Imagine again that a child you take care of gets a middle ear infection. What decision about using antibiotics might you make?

[ ] The child usually should ***take*** antibiotics.

[ ] The child usually should ***not take*** antibiotics.

[ ] Unsure

Trial B

18. Imagine again that you have tennis elbow. What decision about the management option might you make?

[ ] I would probably have a corticosteroid injection.

[ ] I would probably have physiotherapy.

[ ] I would probably wait and watch to see if it gets better on its own.

[ ] Unsure

Questions 19-23 are the same for both trials

19. **Can you please indicate the most important reason for your decision?**

Questions 20-28 are the same for both trials

Trial A statement: You have just seen A XXXX-(PIPE IN TEXT AS PER GRAPH SHOWN pictograph bar graph, line graph, text) presenting information about the recovery of children with middle ear infection

Trial B statement: You have just seen a XXXX (PIPE IN TEXT AS PER GRAPH SHOWN pictograph bar graph, line graph, text) presenting information about the recovery of people with tennis elbow over time.

The allocated intervention was shown here again

Now we would like to ask you a few questions about ***how the information*** is presented.

20. In this presentation, how ***easy*** was it to understand the information presented? (From 1= Not at all easy to 10= extremely easy)
21. How ***satisfied*** are you with **HOW** the information is presented? (From 1= not at all satisfied to 10=I am totally satisfied)
22. **In the future, you may have to make a decision about managing a health condition. To help you make this decision you may like more information about how your health may change over time because of this condition (i.e. its prognosis). You have just seen A (PIPE IN TEXT AS PER GRAPH SHOWN pictograph bar graph, line graph, text) presenting prognostic information.**

For peer review only - http://bmjopen.bmj.com/site/about/guidelines.xhtml

We will now show other ways to you and ask you to rank these ways from the MOST preferred option (1) to the LEAST preferred option (4) for receiving this type of information in future. [SHOW ALL INTERVENTIONS ALONG WITH LABELS]

[ ] Text only
[ ] A bar graph
[ ] A pictograph
[ ] A line graph

23. In a few words, please tell us why you chose the option that you ranked the highest (1)?

24. Do you prefer another way of presenting prognostic information THAT IS NOT IN THE PREVIOUS LIST (e.g., a combination of text and a graph, a table, a combination of a table and a graph)?

Please note that these are just examples and please feel free to suggest any other ways you may prefer.

25. Please rate how much experience you have had with each of the following graphs before today: (Rating from 1= no experience to 5 = a lot of experience) (graphs were shown)

Questions 26 and 27 are the NVS health literacy scale

28. If you had any difficulties with the survey (reading, understanding questions, technical problems) or you have any comments to improve the survey, please note them here

Thank you for your time and for taking part in our survey

For peer review only

Additional file 2

(Supplementary results)

Comprehension results **2**

Equivalence comparisons of graph types **3**

Association of comprehension with HL, numeracy, and education level

Trial A (Acute otitis media) 4

Trial B (Lateral epicondylitis) 4

Change of decision intention by intervention

Trial A (Acute otitis media) 5

Trial B (Lateral epicondylitis) 7

Reasons for decision intention Q19

Trial A (Acute otitis media) 9

Trial B (Lateral epicondylitis) 12

Reasons for first choice preference Q23

Trial A (Acute otitis media) 16

Trial B (Lateral epicondylitis) 18

Exploratory results

Association of comprehension with MMM, time to complete comprehension question, and graph preference

Trial A (Acute otitis media) 20

Trial B (Lateral epicondylitis) 20

Visual presentation preference by previous graph experience

Trial A (Acute otitis media) 21

Trial B (Lateral epicondylitis) 21

For peer review only

For peer review only - http://bmjopen.bmj.com/site/about/guidelines.xhtml

Comprehension results

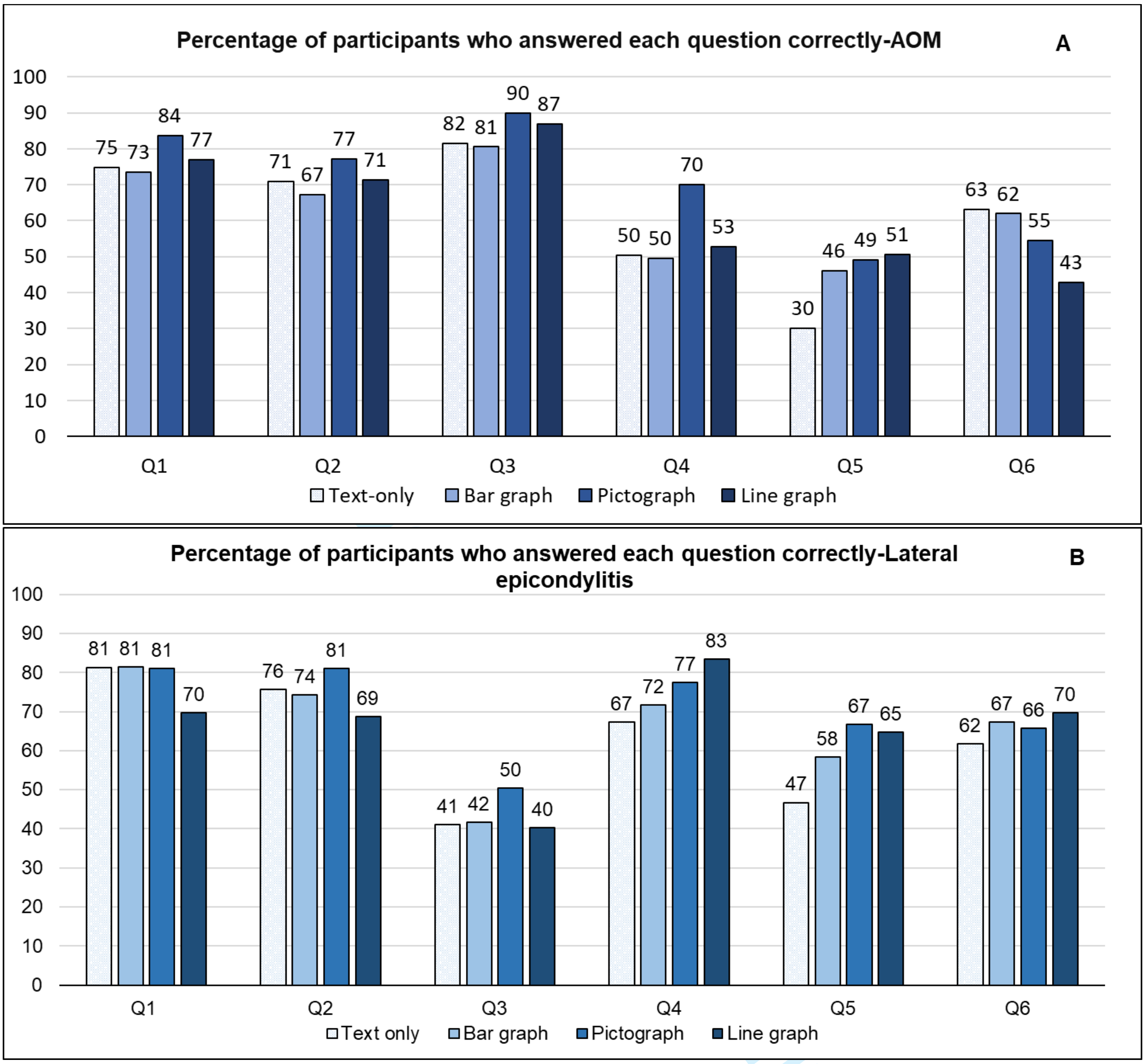

Comprehension of prognostic information. The numbers displayed represent the percentage of participants who answered each question correctly in (A) acute condition-AOM and (B) chronic condition-lateral epicondylitis. Q1-Q6 corresponds to Q12-Q17 in the survey (see additional file 1 for the questions).

For peer review only - http://bmjopen.bmj.com/site/about/guidelines.xhtml

Equivalence comparisons of graph types

AOM-Comprehension equivalence comparison between 3 intervention groups

Group 1	Group 2	Difference	95% CI
Pictograph	Bar graph	0.46	0.07 to 0.85
Pictograph	Line graph	0.43	0.03 to 0.84
Line graph	Bar graph	0.03	-0.40 to 0.45

Lateral epicondylitis- Comprehension equivalence comparison between 3 intervention groups

Group 1	Group 2	Difference	95% CI
Pictograph	Bar graph	0.28	-0.14 to 0.70
Pictograph	Line graph	0.26	-0.21 to 0.74
Line graph	Bar graph	0.01	-0.45 to 0.48

For peer review only

For peer review only - http://bmjopen.bmj.com/site/about/guidelines.xhtml

Association of comprehension with HL, numeracy, and education level

Trial A (Acute otitis media)

Variable	R2	Parameter Estimate	P-value	95% Confidence Limits	
Health literacy (categorised)	24%	High vs med 1.19	<.0001	0.86	1.51
		Low vs med -0.78	0.0007	-1.23	-0.33
Education	0%	0.05	0.47	-0.08	0.17
SNS	4%	0.33	<0.0001	0.19	0.48

Trial B (Lateral epicondylitis)

**Variable**	**R2**	**Parameter Estimate**	**P-value**	**95% Confidence Limits**	
**Health literacy (categorised)**	**29%**	**High vs med 1.28**	**<.0001**	**0.94**	**1.61**
		**Low vs med -1.29**	**<0.0001**	**-1.77**	**-0.81**
**Education**	**2%**	**0.21**	**0.0024**	**0.07**	**0.34**
**SNS**	**3%**	**0.3**	**0.0001**	**0.15**	**0.45**

For peer review only

Change of decision intention by intervention[1]

Trial A (Acute otitis media)

Acute condition - Acute otitis media (AOM) - Text only

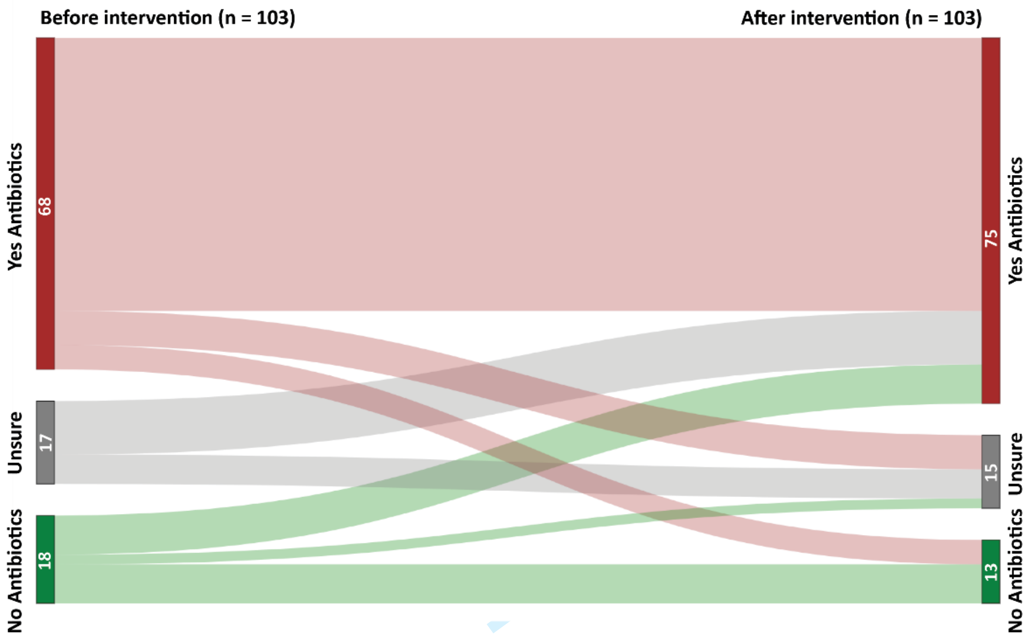

Acute condition - Acute otitis media (AOM) - Pictograph

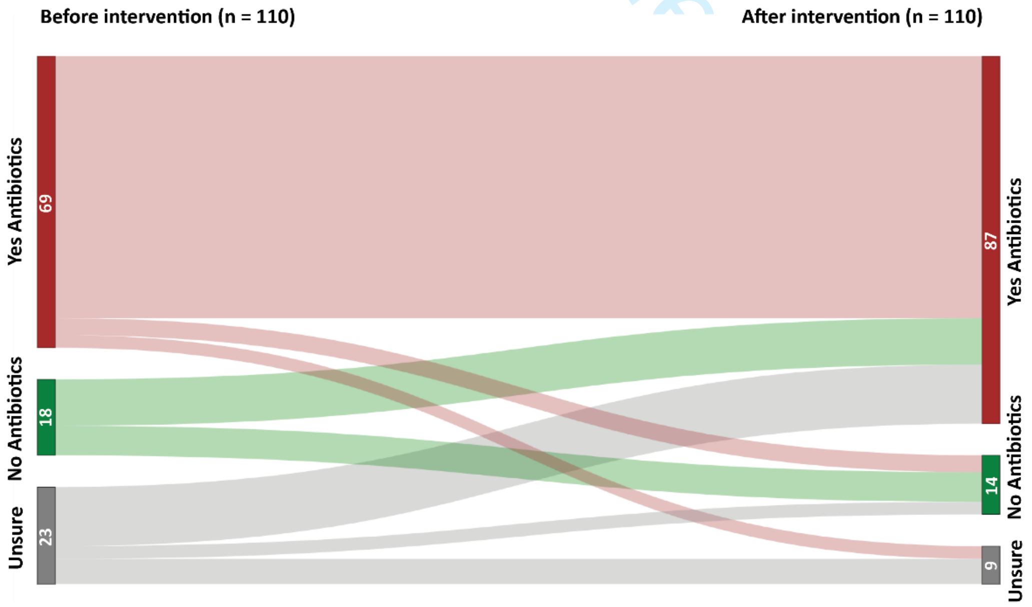

[1] Numbers and percentages of each choice before and after the interventions are presented in table 3 in the main article

For peer review only - http://bmjopen.bmj.com/site/about/guidelines.xhtml

Acute condition - Acute otitis media (AOM) - Line graph

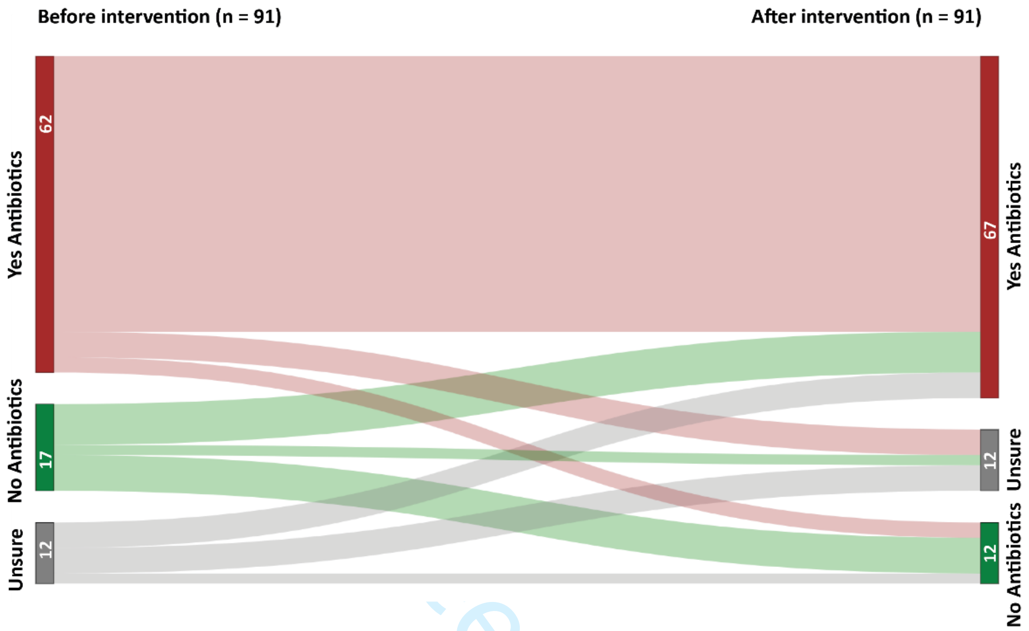

Acute condition - Acute otitis media (AOM) - Bar graph

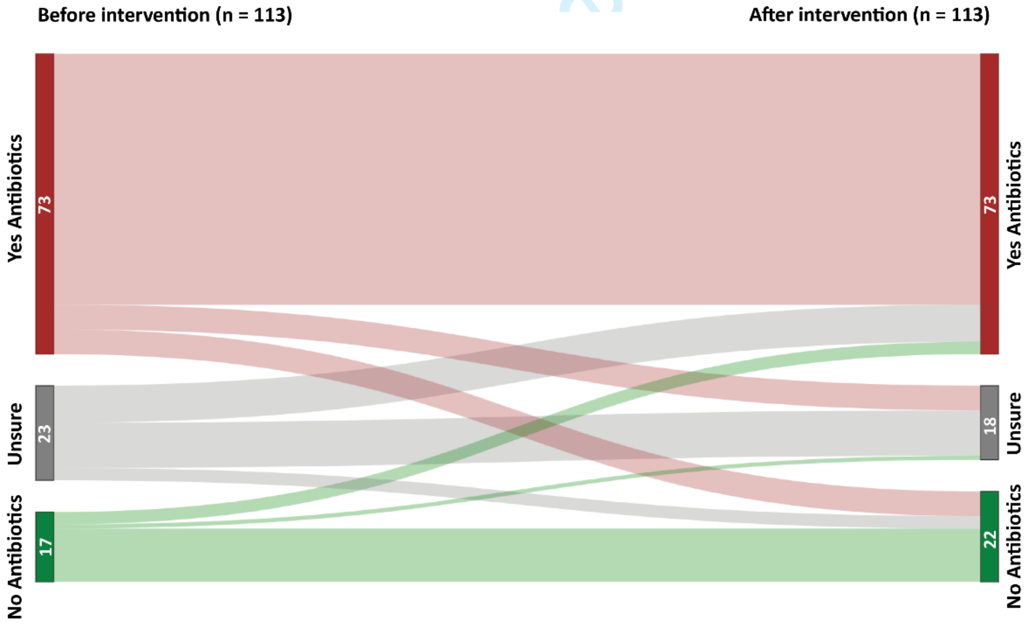

Trial B (Lateral epicondylitis)

Chronic condition - Lateral epicondylitis - Text only

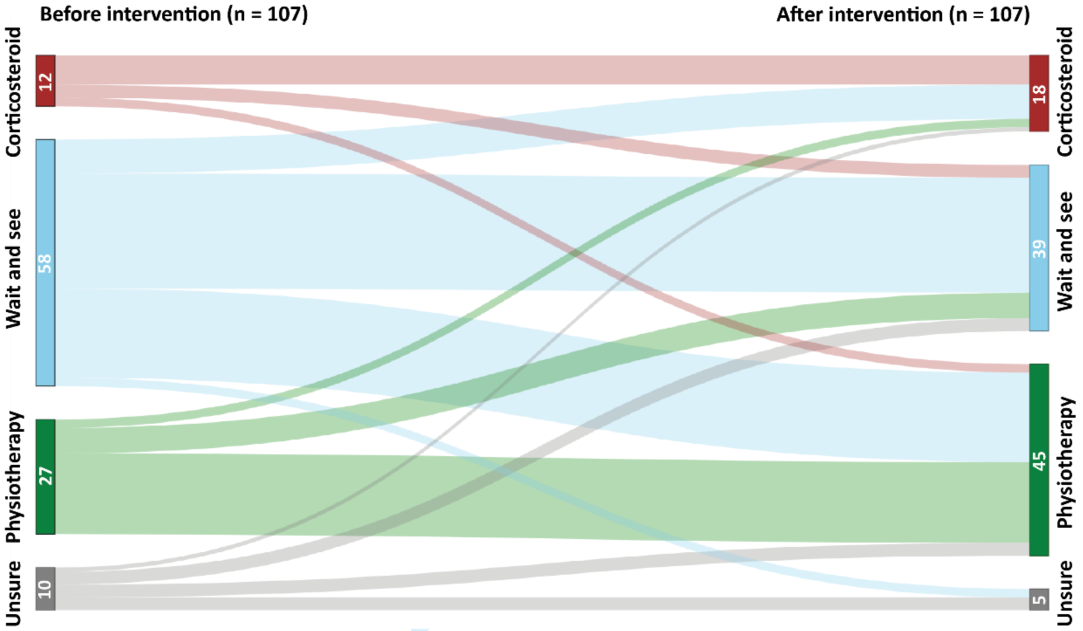

Chronic condition - Lateral epicondylitis - Pictograph

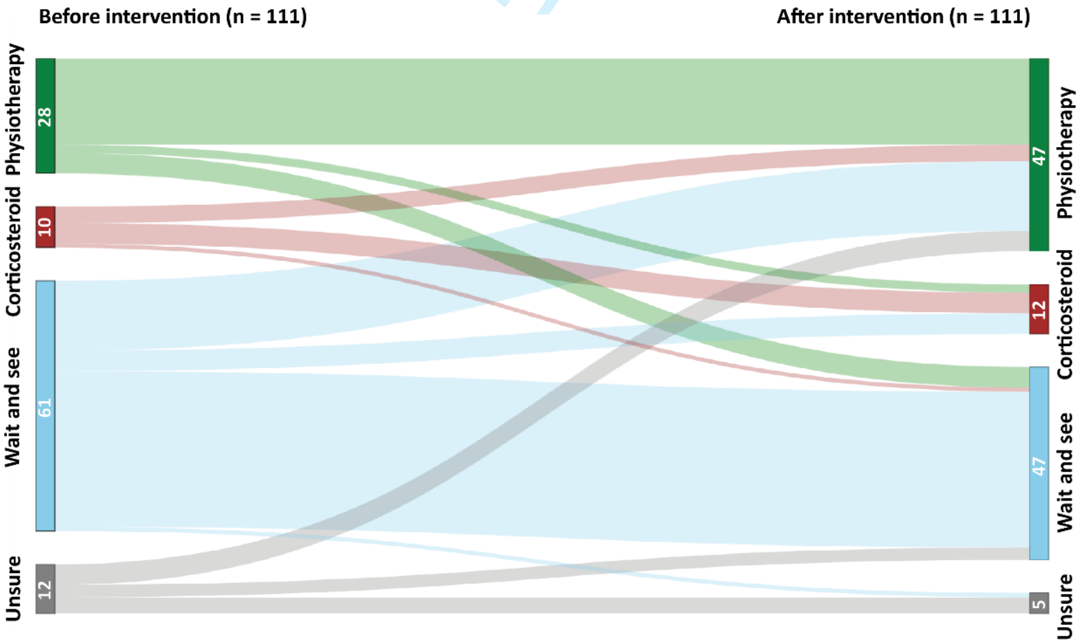

For peer review only - http://bmjopen.bmj.com/site/about/guidelines.xhtml

Chronic condition - Lateral epicondylitis - Bar graph

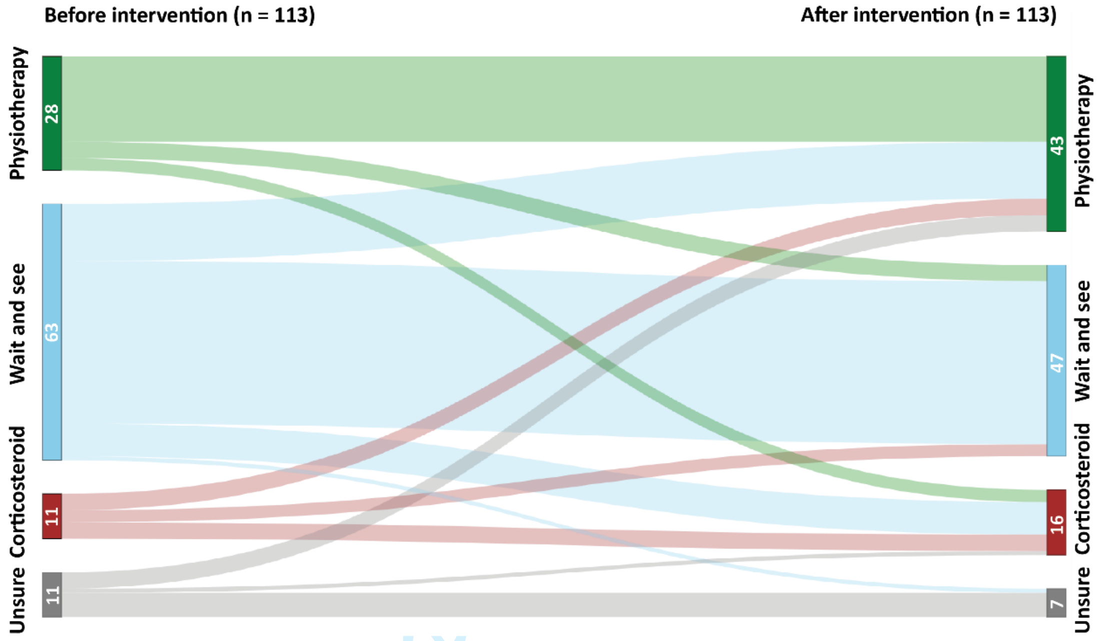

Chronic condition - Lateral epicondylitis - Line graph

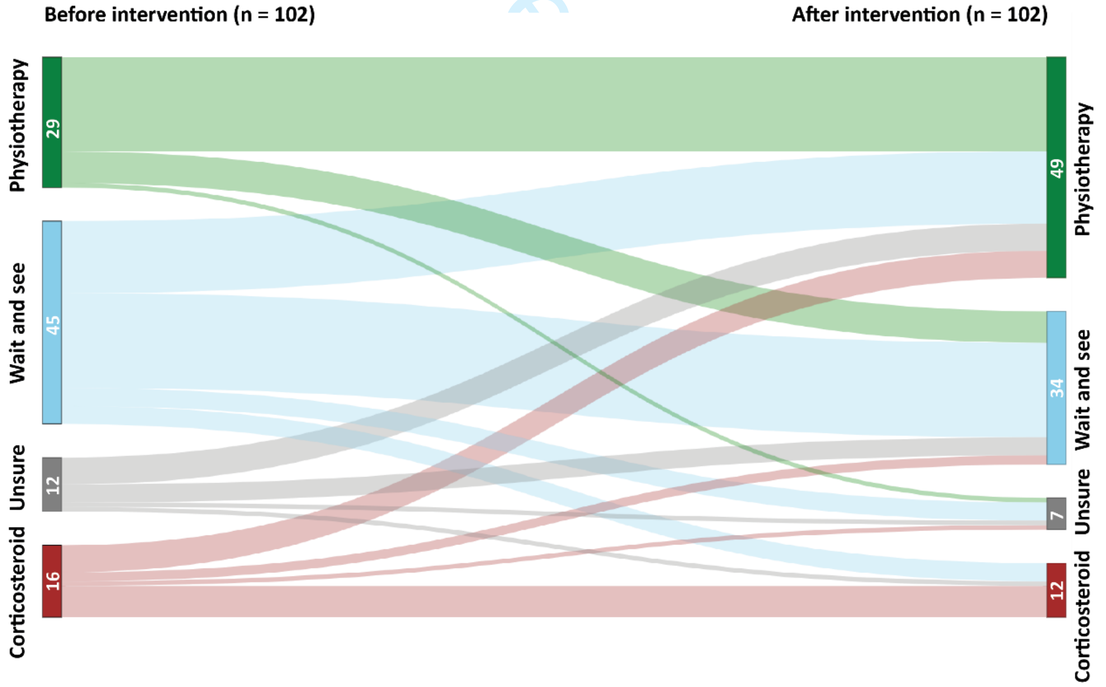

For peer review only - http://bmjopen.bmj.com/site/about/guidelines.xhtml

Reasons for decision intention Q19

Trial A (Acute otitis media)

**Question 19: Can you please indicate the most important reason for your decision?**					
**Reasons of participants who chose to give antibiotics**					
	**Category**	**Description**	**N total**	**Count/ intervention**	**Examples**
1	Based on the presented data	participants who thought data was favouring AB use or referred that their answers were based on the presented data	39	Text-only: 12<br>Bar graph: 9<br>Pictograph: 9<br>Line graph: 9	"the graph indicated that antibiotics has good effects"<br>"data proved fewer children in pain who took antibotics"
2	Antibiotics are effective	Decreased pain<br>Getting better<br>Reduce symptoms	145	Text-only: 33<br>Bar graph: 38<br>Pictograph: 43<br>Line graph: 31	"It helps relieve the pain for the child and fight the infection"<br>"Antibiotics will probably make slow down the infection and also pain."
3	Antibiotics save time	Decrease pain duration, quick recovery from illness, clear the infection faster	45	Text-only: 13<br>Bar graph: 11<br>Pictograph: 11<br>Line graph: 9	"quicker recovery"<br>"Because it helps it get better quickly"<br>"get rid of the pain faster"
4	Antibiotics prevent complications, concerns about complications if antibiotics were not used	Prevent superadded infections, prevent hearing loos, prevent eardrum perforation, prevent recurrence	50	Text-only: 12<br>Bar graph: 12<br>Pictograph: 17<br>Line graph: 9	"to prevent a perforated eardrum"<br>"it is important for your health and to avoid complications"<br>"The child would probably not be able to eliminate the infection themselves and this could possible lead to deafness or even death left untreated."<br>"To ensure that the infection doesnt get worse and permanently affect their hearing"
5	Following medical advice	The GPs decides if I should take antibiotics, or if necessary	14	Text-only: 3<br>Bar graph: 4<br>Pictograph: 4<br>Line graph: 3	"take it only if the doctor says"<br>"I rely on Medical Advice as to how to proceed in situations like this."
6	Based on personal experience or perspectives	Participants who had either negative/positive previous experience with the condition when they used/not antibiotics	21	Text-only: 7<br>Bar graph: 3<br>Pictograph: 4<br>Line graph: 6	"ive been a sufferer of inner ear infections so have my children antis are the only way for us"<br>"have had a child with ear infection, very painful, and always needed antibiotics"
7	Antibiotics are necessary/ needed	Participants who believed antibiotics are needed anyway, e.g., antibiotics work wonder	24	Text-only: 4<br>Bar graph: 5<br>Pictograph: 10<br>Line graph: 5	"you must take antibiotics to stop the infection"<br>"You have to treat an infection."<br>"pain is acute and the ear wont clear itself up"
8	Conditional use of antibiotics	Participants who think they would use antibiotics if pain was severe, or if there is fever, it took longer than expected, if my child is suffering	22	Text-only: 5<br>Bar graph: 5<br>Pictograph: 7<br>Line graph: 5	"Depends on the severity of the case."<br>"if he has fever"

For peer review only - http://bmjopen.bmj.com/site/about/guidelines.xhtml

Reasons of participants who chose not to give antibiotics					
	**Category**	**Description**	**N total**	**Count/ intervention**	**Example**
1	Based on the presented data	participants who thought that the presented data was not favouring antibiotic use	20	Text-only: 5<br>Bar graph: 8<br>Pictograph: 2<br>Line graph: 5	"previous chart showed only small difference"<br>"The statistics don't support antibiotic use for this condition"
2	Based on their preference of other alternatives	Participants who preferred to use alternative options (other medicines, home remedies) or to wait and see if it goes on its own	13	Text-only: 0<br>Bar graph: 5<br>Pictograph: 4<br>Line graph: 4	"In many case home remedy will take care of this problem."<br>"there may be other way to solve this problem"
3	Based on personal preference	Participants who didn't give a specific reason e.g. I like this option	4	Text-only: 2<br>Pictograph: 1<br>Pictograph: 1<br>Line graph: 0	"i think its helpful"<br>"because I like it"
4	Concerns about antibiotic overuse and resistance	Participants who related their decision to the effect of overuse of antibiotics	11	Text-only: 0<br>Bar graph: 7<br>Pictograph: 4<br>Line graph: 0	"not worth becoming immune to antibiotics over a few percentage points."<br>"Because of growing immunity to anibiotics it is always best to avoid them if they are not stricty necessary"
5	Concerns about adverse events	Participants who were worried about the side effects of antibiotics	18	Text-only: 1<br>Bar graph: 8<br>Pictograph: 5<br>Line graph: 4	"Because antibiotics has side effects and most children have self antibody with which he/she can immune himself/herself."<br>"antibiotics can cause allergic reactions in kids"
6	Conditional use of antibiotics	Depends on severity of symptoms, or if prescribed by a GP	12	Text-only: 3<br>Bar graph: 4<br>Pictograph: 4<br>Line graph: 1	"Better not to take antibiotics unless it was really necessary"<br>"wait for doctor decision"
7	Antibiotics have little benefit/ not effective	Participants who thought antibiotics are not of great benefit in this case or in general	11	Text-only: 4<br>Bar graph: 3<br>Pictograph: 1<br>Line graph: 3	"Because generally middle ear infections are viral therefore do not need antibiotics"

For peer review only - http://bmjopen.bmj.com/site/about/guidelines.xhtml

Reasons of participants who were unsure					
	**Category**	**Description**	**N total**	**Count/ intervention**	**Example**
1	Based on the presented data	participants who can't understand the data presented/ need	8	Text-only: 1<br>Bar graph: 4<br>Pictograph: 2<br>Line graph: 1	"very little difference between taking and not taking drugs"<br>"did not understand"<br>"its hard to figure it"
2	Based on their preference of other alternatives	Participants who preferred to use alternative options (other medicines, home remedies), wait and see or having no treatment	6	Text-only: 1<br>Bar graph: 2<br>Pictograph: 2<br>Line graph: 1	"I think it would depend on how severe the infection is, and I would also look for a natural option opposed to anitbiotics"<br>"theres a fine line between the two,,, i prefer to wait a few days and see what eventuates,, not a big fan of antibiotics"
3	Lack of personal knowledge or experience	Participants who had no previous experience of the condition or knowledge about it	5	Text-only: 2<br>Bar graph: 2<br>Pictograph: 0<br>Line graph: 1	"None of my children have ever had a middle ear infection and they are all grown up now"<br>"I do not have children"
4	Concerns about side effects including resistance	Participants who were unsure because they were worried about side effect of using antibiotics	4	Text-only: 0<br>Bar graph: 0<br>Pictograph: 0<br>Line graph: 3	"medication causes the problem"<br>"It will depend on the child's other medical issues and the parents consent. Some children given antibiotics can have serious issues caused by them"
5	Conditional use, depends on severity, …etc	Participants who think using antibiotics depends on individual cases or on specific symptoms or causes	14	Text-only: 5<br>Bar graph: 4<br>Pictograph: 2<br>Line graph: 3	"depends on the history of the indvidual child"<br>"depends if the cause is bacterial"
6	Need more information	Participants who needed more information to make an informed decision	4	Text-only: 1<br>Bar graph: 1<br>Pictograph: 2<br>Line graph: 0	"i can not tell base on just one study"<br>"would need more details how long they have had it , if its reacurring etc"
7	Follow medical advice	Participants who would follow what the doctors say either to take antibiotics or not	9	Text-only: 3<br>Bar graph: 2<br>Pictograph: 0<br>Line graph: 4	"I prefer to consult a doctor first before answering the question."<br>"would consult a doctor"
8	Others	Participants who were unsure, don't know	11	Text-only: 3<br>Bar graph: 5<br>Pictograph: 1<br>Line graph: 2	"I don't know"<br>"Dont have any"

For peer review only

For peer review only - http://bmjopen.bmj.com/site/about/guidelines.xhtml

Trial B (Lateral epicondylitis)

**Q19- Can you please indicate the most important reason for your decision?**					
**Reasons of participants who chose corticosteroid injection**					
	**Category**	**Description**	**N Total**	**Count/ intervention**	**Examples**
1	Based on my understanding of the presented data	Participants who based their answers to their understanding of the presented data	24	Bar graph: 6<br>Pictograph: 5<br>Line graph: 3<br>Text only: 10	"The data shows that it has the best outcome of the three."<br>"Ease them immediate pain, best chance of recovery in the short term"
2	Based on previous experience	Based on the participants own experience with the treatment chosen or negative experience with the alternative treatments either personally or heard about it (e.g., someone they know)	12	Bar graph: 5<br>Pictograph: 3<br>Line graph: 3<br>Text only: 1	"because i have this condition and so far it works best for me"<br>"had previous cortizone injection"
3	Avoiding complications or side effects of alternative options/ against other options or personal preference	Participants who chose corticosteroid injection to avoid any complications that might happen if they choose the other options. either based on previous experience or expectation or as a personal preference	13	Bar graph: 2<br>Pictograph: 4<br>Line graph: 3<br>Text only: 4	"i dont believe in waiting to see if something get better or worse, i prefer this as i have had in the past"<br>"no physio here"
4	Corticosteroid offer a speedy recovery	Patients who chose steroid because it has better outcome on the short term	13	Bar graph: 4<br>Pictograph: 4<br>Line graph: 1<br>Text only: 4	"fast recovery chances"<br>"Time is not on my side, so I would take the speediect option"
5	This treatment is effective	Participants who thought this option would be effective	24	Bar graph: 7<br>Pictograph: 2<br>Line graph: 4<br>Text only: 11	"More people recover by using this method"<br>"to help cure the tennis elbow"
3 irrelevant responses were not categorized					

For peer review only - http://bmjopen.bmj.com/site/about/guidelines.xhtml

Reasons of participants who chose physiotherapy					
	**Category**	**Description**	**N Total**	**Count/intervention**	**Examples**
1	Based on my understanding of the presented data	Participants who based their answers to their understanding of the presented data	96	Bar graph: 20<br>Pictograph: 25<br>Line graph: 23<br>Text only: 28	"As the graphs show Physiotheraphy works"<br>"According to the graph patients getting physiotherapy recovers more quickly."
2	Based on previous experience and personal preference	Based on participants' own experience with the treatment chosen or negative experience with the alternative treatments either personally or heard about it (e.g., someone they know)	33	Bar graph: 10<br>Pictograph: 8<br>Line graph: 9<br>Text only: 6	"had a corticosteroid for my shoulder once willnever get one of those again it hurt all the time"<br>"I had it and it was working great"
3	Avoiding complications of the condition or side effects of alternative options/ against other options	Participants who chose corticosteroid injection to avoid any complications that might happen if they choose the other options either based on previous experience or expectation	13	Bar graph: 2<br>Pictograph: 2<br>Line graph: 3<br>Text only: 6	"drug free"<br>"I am doing something about it rather than waiting and watching and physiotherapy is more natural treatment than injecting myself"
4	A more natural option with less side effects	Participants who thought this option is safer and alleviate the side effects of other options	20	Bar graph: 3<br>Pictograph: 5<br>Line graph: 5<br>Text only: 7	"less invasive and high chance of recovery"<br>"It seems to be the best and least-invasive treatment option"
5	I would follow medical/GP advice	Participants who thought seeking medical advice is the right thing to do	6	Bar graph: 3<br>Pictograph: 0<br>Line graph: 3<br>Text only: 0	"get an expert to help with any pain or swelling"<br>"see a doctor sooner"
6	Physiotherapy is better on the long term	Participants who based their response mainly on the long-term recovery rate	12	Bar graph: 3<br>Pictograph: 2<br>Line graph: 2<br>Text only: 5	"Seems to be the best long term option"<br>"The long-term outcome"
7	Based on both short term and long-term effect	Participants who compared effectiveness both on the short term and long term	12	Bar graph: 1<br>Pictograph: 5<br>Line graph: 4<br>Text only: 2	"Best chance of short and long term recovery."<br>"Physiotherapy had a better long term result compared to the others which had a more of a short term solution"
8	Treatment is effective	Participants who thought this option is effective	94	Bar graph: 15<br>Pictograph: 28<br>Line graph: 27<br>Text only: 24	"I think it would help recover"<br>"Physio includes execises which help recovery and future protection"
9	Physiotherapy provides quicker recovery	Participants who believed physiotherapy will provide faster/ quicker recovery	33	Bar graph: 7<br>Pictograph: 9<br>Line graph: 6<br>Text only: 11	"To treat it quicker"<br>"to recover faster"

For peer review only - http://bmjopen.bmj.com/site/about/guidelines.xhtml

Reasons of participants who chose to wait and watch					
	**Category**	**Description**	**N Total**	**Count/intervention**	**Examples**
1	Based on my understanding of the presented data	Participants who based their answers to their understanding of the presented data	45	Text only: 9<br>Bar graph: 14<br>Pictograph: 15<br>Line graph:7	"Because the data shows that time will help heal the condition, and that although physiotherapy helped, doing nothing ultimately has the same outcome."
2	Based on previous experience	Based on the participants own experience with the treatment chosen or negative experience with the alternative treatments either personally or heard about it (e.g., someone they know)	15	Text only: 2<br>Bar graph: 6<br>Pictograph: 3<br>Line graph:4	"previous history and treatment"<br>"never had success with physio and other health issues make corticosteroid injection risky"
3	Avoiding complications of alternative options/ against other options	Participants who chose corticosteroid injection to avoid any complications that might happen if they choose the other options either based on previous experience or expectation	21	Text only: 3<br>Bar graph: 5<br>Pictograph: 8<br>Line graph:5	"I have tennis elbow now, I tend to let it run its course. I don't see the value in Physio vs time, and Cortisone damages the bone"<br>"i dont really like injections and physio is expensive"
4	A more natural option with less side effects	Participants who thought this option is safer and alleviate the side effects of other options	56	Text only: 16<br>Bar graph: 14<br>Pictograph: 14<br>Line graph:12	"safe option"<br>"Don't believe in using drugs if it can be avoided."
5	Long term benefit	Participants who based their response mainly on the long-term recovery rate	16	Text only: 3<br>Bar graph: 8<br>Pictograph: 3<br>Line graph:2	"The long term outcome isn't different enough to make me think that one course of action is much better than another"
6	Treatment is effective	Participants who thought this option is effective	38	Text only: 8<br>Bar graph: 11<br>Pictograph: 11<br>Line graph: 8	"effective"<br>"more chance of recovery"
7	Cost free	Participants who chose this option to avoid costs of treatment either because it is free, their insurance would not cover other treatment expenses or they wouldn't wish to spend money to achieve very minimal better chance recovery with the other options	35	Text only: 6<br>Bar graph: 11<br>Pictograph: 11<br>Line graph:7	"If I can save money not going to the doctor right away, then I'll do it."<br>"This is primarily from a cost perspective as I do not have the optimal health insurance that covers the cost of physiotherapy"

For peer review only - http://bmjopen.bmj.com/site/about/guidelines.xhtml

8	Depends on nature/ severity of the disease	Participants who chose this option as first option but are open to chose different options depending on the severity and time of the condition	17	Text only: 6<br>Bar graph: 5<br>Pictograph: 5<br>Line graph:1	"It would depend on the level of pain/discomfort and functional impairment, but I generally feel I could handle it at home with exercise and possibly analgesics if necessary"
9	Avoidance of medical advice	Either don't trust GPs/ medical advice	11	Text only: 2<br>Bar graph: 3<br>Pictograph: 4<br>Line graph:2	"I don't trust the medical profession that much, always been a wait and see person"<br>"i hate doctors"
10	compared both short term and long-term effects	Participants who compared effectiveness both on the short term and long term	4	Text only: 0<br>Bar graph: 2<br>Pictograph: 1<br>Line graph:1	"Short term you might get better results from the injection but in the long term you just need to let it work out"

Reasons of participants who were unsure					
	**Category**	**Description**	**N Total**	**Count/intervention**	**Example**
1	No reason, don't know, unsure	Participants who had no reason for their choice	11	Text only: 1<br>Bar graph: 4<br>Pictograph: 3<br>Line graph:3	"Unsure"<br>"no idea"
2	Conditional choice	Depending on severity, work commitments, cost	8	Text only: 2<br>Bar graph: 3<br>Pictograph: 1<br>Line graph:2	"depen on how much it's pain"<br>"it would depend on situation and if I was working and how much it impacted my life"
3	Previous experience/ lack of personal experience	Based on the participants own experience with the treatment chosen or negative experience with the alternative treatments either personally or heard about it (e.g., someone they know)	2	Text only: 0<br>Bar graph: 0<br>Pictograph: 1<br>Line graph:1	"because I'm unsure how I would react to find out that's what I have."<br>" Hard question... I have bilateral epicondylitis in both arms and have tried all of the above so I know nothing works lol Probably watch and wait"
4	Follow medical advice	Participants who thought seeking medical advice is the right thing to do	3	Text only: 2<br>Bar graph: 0<br>Pictograph: 0<br>Line graph:1	"I would see my doctor and be advised by his medical expertise which way to receive treatment"<br>"would probably consult with my doctor for the right treatment"

For peer review only - http://bmjopen.bmj.com/site/about/guidelines.xhtml

Reasons for first choice preference Q23

Trial A (Acute otitis media)

Q23. In a few words, please tell us why you chose the option that you ranked the highest (1)?		
	**Participants who chose text-only first**	
	**Explanation**	**Count**
0	No reason, Unsure, I don't know	6
1	It is easy to understand, clear, concise or it is just good	84
2	It is better than other presentations and easier to compare the different options in this presentation	25
3	It is my personal preference, or the presentation that I am more familiar with or appeals to me and my understanding the best	14
4	Less effort and time consuming to understand the information	3

	**Participants who chose bar graph first**	
	**Explanation**	**Count**
0	No reason, Unsure, I don't know	2
1	It is easy to understand, clear, concise or it is just good	71
2	It is better than other presentations and easier to compare the different options in this presentation	40
3	It is my personal preference, or the presentation that I am more familiar with or appeals to me and my understanding the best	9
4	Less effort and time consuming to understand the information	4
5	Visually better presented, I can understand the information at a glance. The colours are easy to distinguish	27
6	Better in showing the progress, prognosis, trend over time	0

	**Participants who chose pictograph first**	
	**Explanation**	**Count**
0	No reason, Unsure, I don't know	0
1	It is easy to understand, clear, concise or it is just good	32
2	It is better than other presentations and easier to compare the different options in this presentation	14
3	It is my personal preference, or the presentation that I am more familiar with or appeals to me and my understanding the best	1
4	Less effort and time consuming to understand the information	3
5	Visually better presented, I can understand the information at a glance. The colours are easy to distinguish	15
6	Better in showing the progress, prognosis, trend over time	0

For peer review only - http://bmjopen.bmj.com/site/about/guidelines.xhtml

	Participants who chose line graph first	
	**Explanation**	**Counts**
0	No reason, Unsure, I don't know	3
1	It is easy to understand, clear, concise or it is just good	54
2	It is better than other presentations and easier to compare the different options in this presentation	27
3	It is my personal preference, or the presentation that I am more familiar with or appeals to me and my understanding the best	1
4	Less effort and time consuming to understand the information	5
5	Visually better presented, I can understand the information at a glance. The colours are easy to distinguish	18
6	Better in showing the progress, prognosis, trend over time	11

For peer review only

Trial B (Lateral epicondylitis)

Q23. In a few words, please tell us why you chose the option that you ranked the highest (1)?		
	**Participants who chose text-only first**	
	**Explanation**	**Counts**
0	No reason, Unsure, I don't know	9
1	It is easy to understand, clear, concise or it is just good	67
2	It is better than other presentations and easier to compare the different options in this presentation	54
3	It is my personal preference, or the presentation that I am more familiar with or appeals to me and my understanding the best	16
4	Less effort and time consuming to understand the information	15

	**Participants who chose pictograph first**	
	**Explanation**	**Counts**
0	No reason, Unsure, I don't know	5
1	It is easy to understand, clear, concise or it is just good	25
2	It is better than other presentations and easier to compare the different options in this presentation	24
3	It is my personal preference, or the presentation that I am more familiar with or appeals to me and my understanding the best	3
4	Less effort and time consuming to understand the information	2
5	Visually better presented, I can understand the information at a glance. The colours are easy to distinguish	16
6	Better in showing the progress, prognosis, trend over time	0

	**Participants who chose bar graph first**	
	**Explanation**	**Counts**
0	No reason, Unsure, I don't know	3
1	It is easy to understand, clear, concise or it is just good	55
2	It is better than other presentations and easier to compare the different options in this presentation	68
3	It is my personal preference, or the presentation that I am more familiar with or appeals to me and my understanding the best	6
4	Less effort and time consuming to understand the information	8
5	Visually better presented, I can understand the information at a glance. The colours are easy to distinguish	28
6	Better in showing the progress, prognosis, trend over time	2

For peer review only - http://bmjopen.bmj.com/site/about/guidelines.xhtml

	Participants who chose line graph first	
	**Explanation**	**Counts**
0	No reason, Unsure, I don't know	1
1	It is easy to understand, clear, concise or it is just good	28
2	It is better than other presentations and easier to compare the different options in this presentation	26
3	It is my personal preference, or the presentation that I am more familiar with or appeals to me and my understanding the best	5
4	Less effort and time consuming to understand the information	4
5	Visually better presented, I can understand the information at a glance. The colours are easy to distinguish	2
6	Better in showing the progress, prognosis, trend over time	17

For peer review only

Exploratory results

Association between comprehension and MMM, time to complete comprehension questions, and graph preference

Trial A (Acute otitis media)

Variable	R2	Parameter Estimate	P-value	95% Confidence Limits	Variable
MMM scale (categorised)	2%	High vs med -0.60	0.01	-1.05	-0.14
		Low vs med -0.18	0.3	-0.52	0.16
Time to complete comprehension questions (mins)	2%	0.02	0.0066	0.01	0.04
Graph preference	3%	Bar vs text: 0.63	0.0008	0.27	1
		Picto vs text: 0.69	0.0045	0.21	1.16
		Line vs text: 0.52	0.011	0.12	0.92

Trial B (Lateral epicondylitis)

Variable	R2	Parameter Estimate	P-value	95% Confidence Limits	Variable
MMM scale (categorised)	3%	High vs med -0.81	0.0009	-1.28	-0.33
		Low vs med -0.07	0.71	-0.43	0.29
Time to complete comprehension questions (mins)	1%	0.03	0.053	0	0.05
Graph preference	4%	Bar vs text: 0.63	0.0011	0.25	1
		Picto vs text: 0.65	0.0077	0.17	1.12
		Line vs text: 0.84	0.0006	0.37	1.32

For peer review only

Overall Visual presentation preference by graph experience

Trial A (Acute otitis media)

Preference	N	Previous experience	N	Mean	Std Dev	Minimum	Maximum
Text only	124.0	Bar graph	124.0	2.9	1.2	1	5
		Pictograph	124.0	2.4	1.2	1	5
		Line graph	124.0	3.2	1.3	1	5
Bar graph	137.0	Bar graph	137.0	3.7	1.2	1	5
		Pictograph	137.0	2.5	1.2	1	5
		Line graph	137.0	3.4	1.2	1	5
Pictograph	58.0	Bar graph	58.0	3.7	1.0	1	5
		Pictograph	58.0	3.6	1.1	1	5
		Line graph	58.0	3.8	0.9	2	5
Line graph	98.0	Bar graph	98.0	3.2	1.2	1	5
		Pictograph	98.0	2.6	1.2	1	5
		Line graph	98.0	4.1	1.1	1	5

Trial B (Lateral epicondylitis)

Preference	N	Previous experience	N	Mean	Std Dev	Minimum	Maximum
Text only	144	Bar graph	144	2.92	1.18	1	5
		Pictograph	144	2.22	1.22	1	5
		Line graph	144	2.89	1.2	1	5
Bar graph	154	Bar graph	154	3.96	1.07	1	5
		Pictograph	154	2.48	1.19	1	5
		Line graph	154	3.51	1.12	1	5
Pictograph	68	Bar graph	68	3.71	1.12	1	5
		Pictograph	68	3.49	1.31	1	5
		Line graph	68	3.78	1.09	1	5
Line graph	67	Bar graph	67	3.64	1.05	1	5
		Pictograph	67	2.63	1.23	1	5
		Line graph	67	4.15	1.09	1	5

For peer review only - http://bmjopen.bmj.com/site/about/guidelines.xhtml

Items to include when reporting a randomized trial in a journal or conference abstract

Item	Description	Reported on line number
Title	Identification of the study as randomized	1-2
Authors *	Contact details for the corresponding author	4-20
Trial design	Description of the trial design (e.g. parallel, cluster, non-inferiority)	40, 42-43
Methods		
Participants	Eligibility criteria for participants and the settings where the data were collected	44-48
Interventions	Interventions intended for each group	49-53
Objective	Specific objective or hypothesis	38-41
Outcome	Clearly defined primary outcome for this report	54
Randomization	How participants were allocated to interventions	In the main article
Blinding (masking)	Whether or not participants, care givers, and those assessing the outcomes were blinded to group assignment	In the main article
Results		
Numbers randomized	Number of participants randomized to each group	45-48
Recruitment	Trial status	completed
Numbers analysed	Number of participants analysed in each group	45-48
Outcome	For the primary outcome, a result for each group and the estimated effect size and its precision	57-63
Harms	Important adverse events or side effects	No harms
Conclusions	General interpretation of the results	64-67
Trial registration	Registration number and name of trial register	68
Funding	Source of funding	92-95

**this item is specific to conference abstracts*

For peer review only - http://bmjopen.bmj.com/site/about/guidelines.xhtml

CONSORT 2010 checklist of information to include when reporting a randomised trial*

Section/Topic	Item No	Checklist item	Reported on page No
**Title and abstract**			
	1a	Identification as a randomised trial in the title	1
	1b	Structured summary of trial design, methods, results, and conclusions (for specific guidance see CONSORT for abstracts)	2-3
**Introduction**			
Background and objectives	2a	Scientific background and explanation of rationale	4-5
	2b	Specific objectives or hypotheses	5
**Methods**			
Trial design	3a	Description of trial design (such as parallel, factorial) including allocation ratio	5
	3b	Important changes to methods after trial commencement (such as eligibility criteria), with reasons	9
Participants	4a	Eligibility criteria for participants	5
	4b	Settings and locations where the data were collected	5
Interventions	5	The interventions for each group with sufficient details to allow replication, including how and when they were actually administered	6-7
Outcomes	6a	Completely defined pre-specified primary and secondary outcome measures, including how and when they were assessed	7-8
	6b	Any changes to trial outcomes after the trial commenced, with reasons	NA
Sample size	7a	How sample size was determined	8
	7b	When applicable, explanation of any interim analyses and stopping guidelines	NA
Randomisation:			
Sequence generation	8a	Method used to generate the random allocation sequence	6
	8b	Type of randomisation; details of any restriction (such as blocking and block size)	6
Allocation concealment mechanism	9	Mechanism used to implement the random allocation sequence (such as sequentially numbered containers), describing any steps taken to conceal the sequence until interventions were assigned	6

For peer review only

Implementation	10	Who generated the random allocation sequence, who enrolled participants, and who assigned participants to interventions	6
Blinding	11a	If done, who was blinded after assignment to interventions (for example, participants, care providers, those assessing outcomes) and how	10
	11b	If relevant, description of the similarity of interventions	6-7
Statistical methods	12a	Statistical methods used to compare groups for primary and secondary outcomes	9
	12b	Methods for additional analyses, such as subgroup analyses and adjusted analyses	9-10
**Results**			
Participant flow (a diagram is strongly recommended)	13a	For each group, the numbers of participants who were randomly assigned, received intended treatment, and were analysed for the primary outcome	Figure 3
	13b	For each group, losses and exclusions after randomisation, together with reasons	Figure 3
Recruitment	14a	Dates defining the periods of recruitment and follow-up	5
	14b	Why the trial ended or was stopped	NA
Baseline data	15	A table showing baseline demographic and clinical characteristics for each group	Table 1
Numbers analysed	16	For each group, number of participants (denominator) included in each analysis and whether the analysis was by original assigned groups	Tables and figures
Outcomes and estimation	17a	For each primary and secondary outcome, results for each group, and the estimated effect size and its precision (such as 95% confidence interval)	10-14, and tables
	17b	For binary outcomes, presentation of both absolute and relative effect sizes is recommended	NA
Ancillary analyses	18	Results of any other analyses performed, including subgroup analyses and adjusted analyses, distinguishing pre-specified from exploratory	Additional file 2
Harms	19	All important harms or unintended effects in each group (for specific guidance see CONSORT for harms)	Ethics application
**Discussion**			
Limitations	20	Trial limitations, addressing sources of potential bias, imprecision, and, if relevant, multiplicity of analyses	14-15
Generalisability	21	Generalisability (external validity, applicability) of the trial findings	15-16

For peer review only

Interpretation	22	Interpretation consistent with results, balancing benefits and harms, and considering other relevant evidence	14-17
**Other information**			
Registration	23	Registration number and name of trial registry	5
Protocol	24	Where the full trial protocol can be accessed, if available	In the registry
Funding	25	Sources of funding and other support (such as supply of drugs), role of funders	18

*We strongly recommend reading this statement in conjunction with the CONSORT 2010 Explanation and Elaboration for important clarifications on all the items. If relevant, we also recommend reading CONSORT extensions for cluster randomised trials, non-inferiority and equivalence trials, non-pharmacological treatments, herbal interventions, and pragmatic trials. Additional extensions are forthcoming: for those and for up to date references relevant to this checklist, see www.consort-statement.org.

For peer review only

BMJ Open

Effect of different visual presentations on the public's comprehension of prognostic information using acute and chronic condition scenarios: Two online randomised controlled trials

Journal:	*BMJ Open*
Manuscript ID	bmjopen-2022-067624.R2
Article Type:	Original research
Date Submitted by the Author:	27-Apr-2023
Complete List of Authors:	Abukmail, Eman; Bond University Institute for Evidence-Based Healthcare, Faculty of Health Sciences and Medicine<br>Bakhit, Mina; Bond University, Centre for research in Evidence-Based Practice<br>Jones, Mark; Bond University<br>Del Mar, Chris; Bond University, Faculty of Health Sciences and Medicne<br>Hoffmann, Tammy; Bond University
**Primary Subject Heading**:	General practice / Family practice
Secondary Subject Heading:	Patient-centred medicine, Communication, Evidence based practice
Keywords:	GENERAL MEDICINE (see Internal Medicine), PRIMARY CARE, INTERNAL MEDICINE, Prognosis

SCHOLARONE™
Manuscripts

I, the Submitting Author has the right to grant and does grant on behalf of all authors of the Work (as defined in the below author licence), an exclusive licence and/or a non-exclusive licence for contributions from authors who are: i) UK Crown employees; ii) where BMJ has agreed a CC-BY licence shall apply, and/or iii) in accordance with the terms applicable for US Federal Government officers or employees acting as part of their official duties; on a worldwide, perpetual, irrevocable, royalty-free basis to BMJ Publishing Group Ltd ("BMJ") its licensees and where the relevant Journal is co-owned by BMJ to the co-owners of the Journal, to publish the Work in this journal and any other BMJ products and to exploit all rights, as set out in our licence.

The Submitting Author accepts and understands that any supply made under these terms is made by BMJ to the Submitting Author unless you are acting as an employee on behalf of your employer or a postgraduate student of an affiliated institution which is paying any applicable article publishing charge ("APC") for Open Access articles. Where the Submitting Author wishes to make the Work available on an Open Access basis (and intends to pay the relevant APC), the terms of reuse of such Open Access shall be governed by a Creative Commons licence – details of these licences and which Creative Commons licence will apply to this Work are set out in our licence referred to above.

Other than as permitted in any relevant BMJ Author's Self Archiving Policies, I confirm this Work has not been accepted for publication elsewhere, is not being considered for publication elsewhere and does not duplicate material already published. I confirm all authors consent to publication of this Work and authorise the granting of this licence.

For peer review only

Effect of different visual presentations on the public's comprehension of prognostic information using acute and chronic condition scenarios: Two online randomised controlled trials

Eman Abukmail, MD, M.Sc.[1], eabukmai@bond.edu.au

Mina Bakhit, MBBCh, PhD[1], mbakhit@bond.edu.au

Mark Jones, PhD[1], majones@bond.edu.au

Chris Del Mar, MD[1], FRACGP, cdelmar@bond.edu.au

Tammy Hoffmann, PhD[1], thoffman@bond.edu.au

[1]Institute for Evidence-Based Healthcare, Faculty of Health Sciences and Medicine, Bond University, Queensland, Australia.

Corresponding author:

Eman Abukmail, MD, M.Sc.

Institute for Evidence-Based Healthcare
Faculty of Health Sciences and Medicine
Bond University
14 University Dr, Robina
QLD, Australia 4229
Email: eabukmai@bond.edu.au

For peer review only

For peer review only - http://bmjopen.bmj.com/site/about/guidelines.xhtml

Manuscript word count: 3455 (Introduction to conclusions, without tables and figure legends)

Abstract word count (without keywords): 300

Tables and Figures: 3 figures and 2 tables, 2 Additional files [Additional file 1 (Supplementary methods), Additional file 2 (Supplementary results)], copy of the registered protocol

References: 35

Keywords:

Health communication, prognosis communication, natural history communication, clinician-patient communication, communication in primary healthcare, visual presentations, formats

For peer review only

For peer review only - http://bmjopen.bmj.com/site/about/guidelines.xhtml

Abstract

Objectives

To assess the effectiveness of bar graph, pictograph, and line graph compared to text-only, and to each other for communicating prognosis to the public.

Design Two online 4-arm parallel-group randomised controlled trials. Statistical significance was set at p<0.016 to allow for 3-primary comparisons.

Participants and setting Two Australian samples were recruited from members registered at Dynata online survey company. In trial A: 470 participants were randomised to one of the four arms, 417 were included in the analysis. In trial B: 499 were randomised and 433 were analysed.

Interventions In each trial 4 visual presentations were tested: bar graph, pictograph, line graph and text-only. Trial A communicated prognostic information about an acute condition (acute otitis media-AOM) and trial B about a chronic condition (lateral epicondylitis). Both conditions are typically managed in primary care where ‘wait and see’ is a legitimate option.

Main outcome: Comprehension of information (scored 0-6).

Secondary outcomes: Decision intention, presentation satisfaction and preferences.

Results In both trials, the mean comprehension score was 3.7 for the text-only group. None of the visual presentations were superior to text-only. In trial A, the adjusted mean difference (MD) compared to text-only was: 0.19 (95% CI -0.16 to 0.55) for bar graph, 0.4 (0.04 to 0.76) for pictograph, and 0.06 (-0.32 to 0.44) for line graph. In trial B, the adjusted MD was: 0.1 (-0.27 to 0.47) for bar graph), 0.38 (0.01

For peer review only - http://bmjopen.bmj.com/site/about/guidelines.xhtml

to 0.74) for pictograph, and 0.1 (-0.27 to 0.48) for line graph. Pairwise comparisons between the three graphs showed all were clinically equivalent (95% CIs between -1.0 and 1.0). In both trials, bar graph was the most preferred presentation (chosen by 32.9% of trial A participants and 35.6% in trial B).

Conclusions

Any of the four visual presentations tested may be suitable to use when discussing quantitative prognostic information.

Trial registration (ACTRN12621001305819)

Keywords: prognosis communication, natural history communication, clinician-patient communication, visual presentations, graphs

For peer review only

For peer review only - http://bmjopen.bmj.com/site/about/guidelines.xhtml

Article Summary

Strengths and limitations

- This study is the first to focus on communicating prognostic information about conditions managed in primary care and for which "wait and see" is a legitimate option.
- The study recruited a wide range of participants to ensure inclusivity and diversity of gender, background, age and educational level.
- Baseline comprehension was not tested as it was not possible to ask specific comprehension questions without showing participants the interventions.
- Trial participants did not need prior or current experience with the condition to be eligible, which may have influenced responses to some questions.

For peer review only

For peer review only - http://bmjopen.bmj.com/site/about/guidelines.xhtml

Introduction

Providing patients with information on their health can encourage active participation in making decisions.[1] Having a conversation about prognosis can involve communicating quantitative data such as the likelihood of occurrence of an outcome, recurrence, or improvement over time, or the likely duration of the illness. Information about a condition's natural history (that is, the natural course of the condition without treatment) is also encompassed under the umbrella of prognostic information. Understanding prognostic information may help patients to have realistic expectations about the course of their condition and make well-informed decisions about subsequent steps.[2 3]

Communicating quantitative information can be challenging for both clinicians and patients[4] and most research has focused on how to communicate treatment benefits and harms. Several factors may contribute to patient's understanding of the information that is conveyed, including the format used and the framing of it.[5] We conducted a systematic review[6] to evaluate the visual presentations (e.g., a table, graph) that have been used to communicate prognostic information. The review identified only 11 studies and found no significant superior effect on comprehension for any of the visual presentations evaluated in the existing studies. It was unclear due to the lack of existing research if visual presentations would facilitate patients' understanding of the communicated information. Most studies presented prognostic information relevant to decision-making about undertaking screening or treatment for cancer.

Some decisions in primary care, for both acute and chronic conditions, may be enhanced by having prognostic information. Discussing prognostic information using

For peer review only - http://bmjopen.bmj.com/site/about/guidelines.xhtml

visual presentations may improve patients' comprehension and facilitate decision-making that accommodates patients' values and preferences. The best ways of visually presenting prognostic information for conditions that are typically managed in primary care have rarely been explored. This study aimed to investigate, using conditions typically seen in primary care, whether graphs improve the comprehension of prognostic information compared to text-only, and if the type of graph matters. Within this study, we conducted two trials (A and B). In trial A, the scenario involved an acute condition (where a decision is needed within a few days), and trial B involved a chronic condition that requires a less urgent decision about how to manage it. Results from both trials informed the generalisability of our results for conditions managed in primary care.

Methods

This study was prospectively registered at the Australian New Zealand Clinical Trials Registry (ACTRN12621001305819, 27/09/2021). CONSORT and CHERRIES reporting statements were followed when applicable (See additional file 1).

Study design

The study consisted of two online 4-arm parallel-group randomised controlled trials. Both trials had the same design and differed only in the condition used; trial A conveyed information about an acute condition (acute otitis media-AOM) and trial B conveyed information about a chronic condition (lateral epicondylitis).

Participants and recruitment

Participants were recruited between the 1st and 12th of October 2021 through an online research company, Dynata (www.dynata.com). Dynata recruited a national representative sample (for age, gender, and education level) using an algorithm-

For peer review only - http://bmjopen.bmj.com/site/about/guidelines.xhtml

based sampling tool and sent the surveys to existing registered members who had previously consented to complete online surveys. At the survey commencement, participants read study information and a statement about their right to withdraw from it at any time. To ensure validity and unique responses, Dynata used a captcha at the start of each survey as well as an IP-digital stamp for each participant. Participants were compensated with 5.90 AUD by Dynata for survey completion. An invitation to the study was emailed to the participants individually using an automated router. Participants accessed the survey link via their Dynata dashboard. More information about Dynata Australian demographics, sampling, and recruitment is provided in Additional file 1.

Participants were eligible if they lived in Australia, are 18 years old or older, and could read and understand English. Participants did not have to have the condition presented in the scenario. Participants can only participate in either trial A or B.

Randomisation

In each trial, eligible participants were randomly allocated using a computer-generated sequence, generated by Dynata, to 1 of 4 groups (text-only, bar graph, pictograph, line graph).

Trial scenarios and intervention groups

Trial A: the scenario conveyed information about the prognosis of children with AOM. AOM was chosen as it is one of the most common childhood infections[7], with about 50% to 85% of all children experiencing at least one episode.[8] Data came from a Cochrane systematic review.[8]

Trial B: the scenario conveyed information about the recovery of people with lateral epicondylitis who had completely recovered or not at two time points when they

For peer review only - http://bmjopen.bmj.com/site/about/guidelines.xhtml

decided to watch and wait, have physiotherapy, or have a corticosteroid injection. We chose lateral epicondylitis as it is a chronic condition that has a few treatment options, including the option of waiting for the condition to spontaneously improve.[9]

Graphical details of bar graphs and pictographs (e.g., symmetric distribution in pictograph, adding a scale, shape of the icon) were chosen based on best practice principles identified from relevant literature.[10-27] The interventions used are provided in Additional file 1.

Patient and public involvement

We pilot tested the AOM materials in a convenience sample of day-care centre teachers (n= 8) and the lateral epicondylitis materials with university administration staff (n=9). The feedback helped to iteratively refine minor details of the interventions and the clarity of some survey questions.

Before commencing trial recruitment, online piloting of all trial materials was conducted with approximately 10% of the sample size needed for the full trial (trial A, n= 47; trial B, n= 41). Participants were recruited by Dynata and not invited to subsequently participate in the trials. Piloting data were also used to adjust the sample size calculation and test for any technical problems. These data were not included in the trial results.

Data collection and outcome measurement

Our primary outcome was comprehension of the information presented, measured using 6 questions based on previous studies.[12 16 23 27] (see Additional file 1 for questions). The comprehension questions were developed to assess different understanding skills, for example, the ability to extract numbers from the information provided (e.g., At 10-12 days, out of 100 children who took antibiotics, how many

For peer review only - http://bmjopen.bmj.com/site/about/guidelines.xhtml

had pain?), the ability to use the numbers to compare the two options (e.g., Which group was less likely to experience pain at about 4-7 days?), and the ability to make use of the information (e.g., In the week or so after a middle ear infection starts, the majority of children will NOT be in pain, regardless of whether they do or do not take antibiotics). Therefore, we were interested to assess overall comprehension differences across groups rather than differences in comprehension of individual questions. Secondary outcomes were decision intention, satisfaction with the presented format (before revealing all formats), and format preference (after revealing all formats) and measured with a combination of ranking questions, Likert scales, and open-ended questions (Additional file 1). All outcomes were measured once (immediately post-intervention), except for decision intention which was measured pre-and post-intervention (See Additional file 2 for the results of individual questions).

Participants completed a 3-part, 28-question structured survey developed for the trial, based on surveys from relevant research. **Part 1**: five demographic questions, a baseline decision intention question, a question on previous experience with the condition, the medical maximizer-minimizer scale (MMMS assesses patients' preferences for aggressive versus more passive approaches to health care)[28], and a validated subjective numeracy scale (SNS)[29 30]. **Part 2:** each group was presented with information according to their allocated group (e.g., text-only, bar graph, pictograph, or line graph). This was followed by six comprehension questions, two questions about decision intention, and two about satisfaction with the intervention. **Part 3:** All four intervention presentations (text-only, bar graph, pictograph, and line graph) were revealed to participants and visual presentation preference was measured using three questions. Participants rated their previous graphic experience

For peer review only

on a 1-5 scale. Health literacy level was measured using the Newest Vital Sign (NVS) scale.[31]

The multiple-choice options for the comprehension and decision intention questions were randomised to minimise order bias. Participants were able to see the randomised intervention while answering the questions in Part 2, but once they moved to the next page, they were unable to go back to change their responses. This was to ensure that responses to comprehension questions were not influenced by the other interventions that were revealed in Part 3. Data is available at OSF | Effect of different visual presentations on the public's comprehension of prognostic information using acute and chronic condition scenarios: Two online randomised controlled trials.[32]

Sample size

In the online piloting, the standard deviation (SD) for the primary outcome was, at most, 1.66 units. Assuming 90% power, 0.016% level of significance (adjusted to account for 3 primary comparisons), the difference between groups of 1 unit, and up to 20% missing data, 97 participants were calculated as required in each group (a total of 388 participants) in each trial.

Data analysis

The primary analysis tested whether comprehension was different between each graphic presentation and the text-only presentation (3 comparisons). Linear regression was used and mean differences with 95% confidence intervals (CI) and p-values calculated. Adjusted analysis was performed to account for any potential important baseline imbalances using linear regression, with adjustment for age

For peer review only - http://bmjopen.bmj.com/site/about/guidelines.xhtml

group, education level, and health literacy level. Statistical significance was set at $p<0.016$ to allow for 3 primary comparisons.

To assess the equivalence of the three graphic presentations, 95% confidence intervals for the mean difference between groups were estimated for each of the three pairwise comparisons using linear regression. Equivalence was to be concluded if the 95% confidence intervals were between -1.0 and 1.0. We were guided by a previous study that used 10% as a clinically important difference for a similar comprehension outcome measure.[33] Univariable linear regression models were constructed to estimate the association between comprehension as the dependent variable and the independent variables (including health literacy, numeracy, and education level). The measure of association reported is the R^2 value. SAS® OnDemand for Academics 2014 was used to analyse the data. For the four open-ended questions (e.g., the most important reason for participant's decision to choose one of the management options), two authors (EA, MB) independently categorised the responses. Another author (TH) was consulted when there was disagreement. Each response could be categorised into one or more categories (to a maximum of three). Decision intention and visual presentation satisfaction and preferences were descriptively reported as number and percentage of participants.

Post-protocol analysis and modifications from protocol

It was not feasible to analyse the data blinded to group allocation because we needed to identify the control group (text-only) so that the appropriate comparisons could be made.

For peer review only

We estimated the association between comprehension as the dependent variable and time to finish the comprehension questions and previous experience with graphs as independent variables. (Results are provided in the Additional file 2)

As part of the review process, we conducted statistical analyses for the secondary outcomes of decision intention and satisfaction. Change in decision intention from pre- to post-intervention was compared between groups using a multinomial logistic regression model with cluster robust standard errors specified to account for the pre/post repeated measures on each participant. The dependent variable was decision intention category, and the independent variables were group, time (pre/post) and the interaction between group and time. A joint test was used to test for evidence of an interaction between group and time with statistical significance set at $p<0.05$.

ANOVA was used to test for differences in the satisfaction outcomes between intervention groups with statistical significance set to $p<0.05$.

Results

Figure 1 shows the CONSORT flow diagram of participants in both trials. Table 1 presents the baseline demographic results. There were no baseline differences among the groups in either trial, except for health literacy which was slightly higher in the pictograph group in trial A.

Please insert figure 1 here

For peer review only

For peer review only - http://bmjopen.bmj.com/site/about/guidelines.xhtml

Figure 1: CONSORT flow diagrams of participants in Trial A and Trial B.Table 1: Participants' characteristics

	Trial A				Trial B			
	**Text only (n= 103)**	**Bar graph (n= 113)**	**Pictograph (n= 110)**	**Line graph (n= 91)**	**Text only (n= 107)**	**Bar graph (n= 113)**	**Pictograph (n= 111)**	**Line graph (n= 102)**
**Age (years) n (%)**								
18-24	5 (4.9)	7 (6.2)	7 (6.4)	4 (4.4)	5 (4.7)	6 (5.3)	5 (4.5)	10 (9.8)
25-34	15 (14.6)	17 (15.0)	18 (16.4)	8 (8.8)	17 (15.9)	22 (19.5)	12 (10.8)	15 (14.7)
35-44	14 (13.6)	19 (16.8)	14 (12.7)	18 (19.8)	21 (19.6)	17 (15.0)	25 (22.5)	16 (15.7)
45-54	25 (24.3)	23 (20.4)	30 (27.3)	8 (8.8)	20 (18.7)	23 (20.4)	19 (17.1)	14 (13.7)
55-64	26 (25.2)	24 (21.2)	23 (20.9)	28 (30.8)	24 (22.4)	24 (21.2)	28 (25.2)	21 (20.6)
65+	18 (17.5)	23 (20.4)	18 (16.4)	25 (27.5)	20 (18.7)	21 (18.6)	21 (18.9)	26 (25.5)
Prefer not to say	0 (0.0)	0 (0.0)	0 (0.0)	0 (0.0)	0 (0.0)	0 (0.0)	1 (0.9)	0 (0.0)
**Gender n (%)**								
Male	48 (46.6)	53 (46.9)	49 (44.6)	47 (51.7)	52(48.6)	44 (38.9)	46 (41.4)	57 (55.9)
Female	55 (53.4)	60 (53.1)	60 (54.6)	44 (48.4)	55(51.4)	69 (61.1)	64 (57.7)	45 (44.1)
Others	0 (0.0)	0 (0.0)	1 (0.9)	0 (0.0)	0 (0.0)	0 (0.0)	1 (0.9)	0 (0.0)
**State n (%)**								
QLD	26 (25.2)	31 (27.4)	18 (16.4)	24 (26.4)	23 (21.5)	20 (17.7)	24 (21.6)	22 (21.6)
NSW	31 (30.1)	34 (30.1)	38 (34.6)	33 (36.3)	32 (29.9)	39 (34.5)	41 (36.9)	33 (32.4)
ACT	0 (0.0)	2 (1.8)	3 (2.7)	1 (1.1)	2 (1.9)	3 (2.7)	1 (0.9)	2 (2.0)
NT	1 (1.0)	0 (0.0)	0 (0.0)	0 (0.0)	1 (0.9)	0 (0.0)	1 (0.9)	1 (1.0)
WA	12 (11.7)	7 (6.2)	17 (15.5)	4 (4.4)	8 (7.5)	17 (15.0)	11 (9.9)	10 (9.8)
VIC	22 (21.4)	31 (27.4)	23 (20.9)	23 (25.3)	28 (26.2)	28 (24.8)	21 (18.9)	22 (21.6)
TAS	2 (1.9)	2 (1.8)	1 (1.0)	0 (0.0)	1 (0.9)	2 (1.8)	2 (1.8)	3 (2.9)
SA	9 (8.7)	6 (5.3)	10 (10.0)	6 (6.6)	12 (11.2)	4 (3.5)	10 (9.0)	9 (8.8)
**Educational level n (%)**								
High school Y10	13 (12.6)	19 (16.8)	12 (10.9)	10 (11.0)	12(11.2)	9(8.0)	11(9.9)	12(11.8)
High school Y12	11 (10.7)	20 (17.7)	25 (22.7)	16 (17.6)	14(13.1)	17(15.0)	19(17.1)	13(12.8)
Certificate I-IV, Diploma, or apprenticeship	31 (30.1)	32 (28.3)	32 (29.1)	25 (27.5)	39(36.5)	34(30.1)	35(31.5)	33(32.4)
Undergraduate degree[1]	32 (31.1)	29 (25.7)	28 (25.5)	28 (30.8)	29(27.1)	37(32.7)	33(29.7)	27(26.5)
Postgraduate degree[2]	16 (15.5)	13 (11.5)	13 (11.8)	12 (13.2)	13(12.2)	16(14.2)	13(11.7)	17(16.7)
**SNS Mean[3] (SD)**	4.2 (0.9)	4.2 (1.1)	4.2 (1.0)	4.2 (1.0)	4.1 (1.0)	4.2 (1.1)	4.2 (1.1)	4.2 (1.0)
SNS ability	4.3 (1.2)	4.3 (1.4)	4.3 (1.2)	4.3 (1.2)	4.1 (1.3)	4.1 (1.4)	4.1 (1.5)	4.3 (1.3)
SNS preferences	4.2 (0.8)	4.1 (1.1)	4.2 (0.9)	4.1 (1.0)	4.1 (0.9)	4.2 (1.0)	4.2 (1.1)	4.1 (1.0)
**Health literacy n (%)**								
4-6 adequate	60 (58.3)	77 (68.1)	86 (78.2)	59 (64.8)	68 (63.55)	76 (67.26)	80 (72.07)	67 (65.69)
2-3 possibly limited	38 (36.9)	25 (22.1)	19 (17.3)	24 (26.4)	24 (22.43)	23 (20.35)	16 (14.41)	26 (25.49)
0-1 highly limited	5 (4.9)	11 (9.7)	5 (4.6)	8 (8.8)	15 (14.02)	14 (12.39)	15 (13.51)	9 (8.82)

[1] Bachelor's degree, or equivalent
[2] Masters or Doctoral degree, or equivalent
[3] SNS: is a self-report measure of numeracy and scored as the average rating of 8 items (4 items assessed ability and 4 items assessed preferences) marked (1-6)

For peer review only - http://bmjopen.bmj.com/site/about/guidelines.xhtml

For peer review only

**MMM scale[4] n (%)**<br><4 Minimizer<br>=4 Neutral<br>>4 Maximizer	 54 (52.4)<br>31 (30.1)<br>18 (17.5)	 65 (57.5)<br>32 (28.3)<br>16 (14.2)	 61 (55.5)<br>31 (28.2)<br>18 (16.4)	 52 (57.1)<br>21 (23.1)<br>18 (19.8)	 63 (58.9)<br>31 (29.0)<br>13 (12.2)	 65 (57.5)<br>27(23.9)<br>21(18.6)	 62 (55.9)<br>28 (25.2)<br>21 (18.9)	 47 (46.1)<br>34 (33.3)<br>21 (20.6)
**Experienced with the condition n (%)**	47 (45.6)	48 (42.5)	50 (45.5)	46 (50.6)	32 (29.9)	46 (40.7)	39 (35.1)	26 (25.5)
**Time (mins) to finish comprehension Qs Median (IQR)**	3.5 (2.6-5.3)	3.5 (2.5-5.1)	3.5 (2.4-5.4)	3.3 (2.3-4.8)	3.4 (2.2-4.6)	3.2 (2.1-4.2)	3.2 (2.6-4.6)	3.1 (2.2-4.6)
**Speaking English at home n (%)**	98 (95.15)	105 (92.92)	101 (91.82)	86 (94.51)	100 (93.5)	101 (89.4)	103 (92.8)	94 (92.2)

Trial A

Comprehension

Figure 2A shows the mean comprehension score for each group. For the percentage of participants, in each group, who correctly answered the comprehension questions, see additional file 2. The total mean score across all questions was 3.71 (SD 1.63), 3.79 (SD 1.54), 3.81 (SD 1.53), and 4.25 (SD 1.40) for text-only, bar graph, line graph, and pictograph groups respectively. Only pictograph group participants had statistically significantly superior comprehension to the text-only group participants, with a mean difference (MD) of 0.54 (95% CI 0.13 to 0.95, *p*-value 0.011). There was no significant difference between the bar graph group and the text-only group or between the line graph group and the text-only group (Table 2). After adjustment for age group, education level, and health literacy: pictograph was not statistically different compared to text-only (MD 0.40, 95% CI 0.04 to 0.76, *p*-value 0.031). Although comprehension was higher in participants in the pictograph group than those in the other two graph groups, the 95% CIs are within the prespecified equivalence threshold, suggesting the differences among the three graph groups are not clinically meaningful. (See Additional file 2).

[4] Medical Maximizer-Minimizer Scale (MMMS): assesses patients' preferences for aggressive versus more passive approaches to health care, medical maximizers (seek health care even for minor problems), medical minimizers: (avoid medical intervention unless it is necessary)

[5]Percentages were reported to one decimal

For peer review only

For peer review only - http://bmjopen.bmj.com/site/about/guidelines.xhtml

Results reporting the analysis of the association between comprehension and health literacy, education level, and numeracy are provided in the supplementary files (Additional file 2).

	Trial A				Trial B			
	**Text only (n= 103)**	**Bar graph (n= 113)**	**Pictograph (n= 110)**	**Line graph (n= 91)**	**Text only (n= 107)**	**Bar graph (n= 113)**	**Pictograph (n= 111)**	**Line graph (n= 102)**
**Superiority comparison (not adjusted)**								
Mean (SD)	3.71 (1.63)	3.79 (1.54)	4.25 (1.40)	3.81 (1.53)	3.74 (1.60)	3.95 (1.59)	4.23 (1.59)	3.96 (1.89)
MD	Ref	0.08	0.54	0.1	Ref	0.21	0.49	0.22
95% CI	Ref	-0.33 to 0.49	0.13 to 0.95	-0.33 to 0.54	Ref	-0.23 to 0.65	0.04 to 0.93	-0.23 to 0.68
P-value	Ref	0.70	0.011	0.63	Ref	0.35	0.032	0.34
**Superiority comparison (adjusted for age group, level of education and categorised health literacy)**								
MD[1]	Ref	0.19	0.4	0.06	Ref	0.1	0.38	0.1
95% CI	Ref	-0.16 to 0.55	0.04 to 0.76	-0.32 to 0.44	Ref	-0.27 to 0.47	0.01 to 0.74	-0.27 to 0.48
P-value	Ref	0.29	0.031	0.76	Ref	0.59	0.046	0.59
**Decision intention n (%) before the intervention**								
Take antibiotics	68 (66.0)	73 (64.6)	69 (62.7)	62 (68.1)				
Not to take antibiotics	18 (17.5)	17 (15.0)	18 (16.4)	17 (18.7)				
Unsure	17 (16.5)	23 (20.4)	23 (20.9)	12 (13.2)	10 (9.4)	11 (9.7)	12 (10.8)	12 (11.8)
To wait and watch					58 (54.2)	63 (55.8)	61 (55.0)	45 (44.1)
To have physiotherapy					27 (25.2)	28 (24.8)	28 (25.2)	29 (28.4)
To have corticosteroid					12 (11.2)	11 (9.7)	10 (9.0)	16 (15.7)
**Decision intention n (%) after the intervention**								
Take antibiotics	75 (72.8)	73 (64.6)	87 (79.1)	67 (73.6)				
Not to take antibiotics	13 (12.6)	22 (19.5)	14 (12.7)	12 (13.2)				
Unsure	15 (14.6)	18 (15.9)	9 (8.2)	12 (13.2)	5 (4.7)	7 (6.2)	5 (4.5)	7 (6.9)
To wait and watch					39 (36.4)	47 (41.6)	47 (42.3)	34 (33.3)
To have physiotherapy					45 (42.1)	43 (38.1)	47 (42.3)	49 (48.0)
To have corticosteroid					18 (16.8)	16 (14.2)	12 (10.8)	12(11.8)
**Satisfaction mean (SD)**								
How easy (1-10)[2]	7.2 (2.2)	7.3 (2.5)	7.7 (2.0)	7.6 (2.2)	7.8 (1.8)	7.8 (2.2)	8.3 (1.8)	7.5 (2.6)
How satisfied (1-10)[3]	7.0 (2.2)	7.4 (2.4)	7.7 (2.0)	7.6 (2.2)	7.8 (1.9)	8.0 (2.0)	8.3 (1.9)	7.7 (2.4)
**Visual presentation preference- most preferred (1st) N (%)**								
Text only	40 (38.8)	38 (33.6)	25 (22.7)	21 (23.1)	43 (40.2)	40 (35.4)	27 (24.3)	34 (33.3)
Bar graph	31 (30.1)	43 (38.1)	34 (30.9)	29 (31.9)	40 (37.4)	56 (49.6)	28 (25.2)	30 (29.4)
Pictograph	9 (8.7)	8 (7.1)	34 (30.9)	7 (7.7)	12 (11.2)	8 (7.1)	45 (40.5)	3 (2.9)
Line graph	23 (22.3)	24 (21.2)	17 (15.5)	34 (37.4)	12 (11.2)	9 (8.0)	11 (9.9)	35 (34.3)
**Visual presentation preference- least preferred (4th) N (%)**								

Please insert figure 2 here

Figure 2: Mean comprehension score of prognostic information per group in trial A (AOM) and trial B (lateral epicondylitis). Comprehension questions are provided in additional file 1. Trial A and B comprehension questions are different.

For peer review only - http://bmjopen.bmj.com/site/about/guidelines.xhtml

Text only	29 (28.2)	25 (22.1)	47 (42.7)	29 (31.9)	20 (18.7)	30 (26.6)	41 (36.9)	32 (31.4)
Bar graph	10 (9.7)	12 (10.6)	12 (10.9)	15 (16.5)	6 (5.6)	8 (7.1)	9 (8.1)	7 (6.9)
Pictograph	43 (41.8)	56 (49.6)	25 (22.7)	34 (37.4)	41 (38.3)	34 (30.1)	11 (9.9)	45 (44.1)
Line graph	21 (20.4)	20 (17.7)	26 (23.6)	13 (14.3)	40 (37.4)	41 (36.3)	50 (45.1)	18 (17.7)
**Previous graph experience[4] mean (SD)**								
Bar graph (1-5)	3.3 (1.2)	3.3 (1.4)	3.4 (1.2)	3.3 (1.2)	3.6 (1.2)	3.6 (1.2)	3.6 (1.1)	3.3 (1.2)
Pictograph (1-5)	2.7 (1.2)	2.6 (1.3)	2.9 (1.3)	2.4 (1.1)	2.5 (1.4)	2.5 (1.3)	2.9 (1.3)	2.4 (1.2)
Line graph (1-5)	3.6 (1.1)	3.3 (1.3)	3.5 (1.1)	3.7 (1.1)	3.5 (1.2)	3.3 (1.2)	3.4 (1.3)	3.6 (1.3)

Table 2: Superiority comparisons of mean comprehension scores**,** Decision intention, Satisfaction with the presentation, Visual presentation preference in all intervention groups, in Trial A and Trial B.

[1]MD is the mean difference from text only group
[2]1=not at all easy to 10= extremely easy
[3]1= not at all satisfied to 10= I am totally satisfied
[4] 1= No experience to 5= a lot of experience

Decision intention

At baseline, about two-thirds of participants (66.0% in text-only, 64.6% in bar graph, 62.7% in pictograph, 68.1% in line graph) chose the option that a child with AOM should usually take antibiotics. This increased after viewing the intervention in three of the groups to (72.8%, 79.1%, and 73.6%) in text-only, pictograph, and line graph respectively) while in the bar graph group, the percentage remained similar (64.6%). However, when statistically tested, there was insufficient evidence of a difference between intervention groups for change in decision intention from pre- to post-intervention (p=0.087). Regardless of the allocated group, more people chose to give the child antibiotics after receiving the prognostic information (n=272,65.2% before; n=302,72.4% after). At least half of each group did not alter their choice, regardless of which intervention they received. (Figure 3A). The most common reason that participants gave for choosing antibiotics was their belief that antibiotics are effective. Other reasons are reported in the supplementary files (Additional file 2). **Satisfaction**

For peer review only

Mean scores for ease of use and satisfaction were similar for all formats, with pictograph scores slightly higher (Table 2). However, there was insufficient evidence of a difference between intervention groups for the how easy scale (p=0.32) and the how satisfied scale (p=0.081).

Visual presentation preference

Participants ranked their allocated intervention as the highest, except for participants allocated to the pictograph group who ranked pictograph and bar graph equally best (30.9% of participants). Overall and regardless of the randomised group, the bar graph was the most preferred option; chosen by 137 (32.9%), followed by text-only (n=124, 29.7%). The pictograph was the option least likely to be ranked as the first choice (chosen by 58,13.9%) (Table 2). For the least preferred options and reasons for preferences, see Additional file 2.

Trial B

Comprehension

The difference in comprehension scores between each of the three interventions (pictograph, bar graph, line graph) and text-only was not statistically significant either before or after adjustment for age group, education level, and health literacy (Table 2).

Figure 2B shows the mean comprehension score for each group. For the percentage of participants, in each group, who answered the comprehension questions correctly, see Additional file 2.

For peer review only - http://bmjopen.bmj.com/site/about/guidelines.xhtml

Results reporting the analysis of the association between comprehension and health literacy, education level, and numeracy are provided in the supplementary files (Additional file 2).

Decision intention

Overall, before receiving any prognostic information, 52.4% (n=227) of participants intended to wait and see when asked about decision intentions. After receiving the information, 30.0% of those changed their choice to physiotherapy, while 11.0% changed to corticosteroid (Figure 3B). A similar pattern was observed across all four interventions (Table 2). However, when statistically tested, there was insufficient evidence of a difference between intervention groups for change in decision intention from pre- to post-intervention (p=0.88). Of the participants who chose physiotherapy (the most common choice after receiving the information, regardless of its type), the most common reasons for doing so were their understanding of the presented information, followed by their belief that this was the most effective option. For other reasons see Additional file 2.

Please insert figure 3 here

Figure 3: Overall change in treatment decision intentions before and after viewing the interventions. The figure shows the number of participants who chose each management option in (A) acute condition-AOM and (B) chronic condition-lateral epicondylitis.

Satisfaction

For peer review only - http://bmjopen.bmj.com/site/about/guidelines.xhtml

Mean scores for ease of use and satisfaction were similar for all formats, with pictograph scores slightly higher (Table 2). However, there was insufficient evidence of a difference between intervention groups for the how easy scale (p=0.059) and the how satisfied scale (p=0.17).

Visual presentation preference

Overall, the bar graph was the most preferred option for receiving prognostic information, chosen by 35.6% of participants, followed by 33.3% who ranked text-only as their most preferred format. The bar graph was also the highest to be chosen as a second preference (35% of participants). By intervention group, 40.2% of the text-only group, 49.6% of bar graph group, 40.5% of pictograph group, and 34.31% of line graph group ranked their allocated intervention as their most preferred option to receive prognostic information in the future.

Table 2 shows the number and proportion of participants, in each group, who chose each format as their most preferred or least preferred option (other ranking choices are provided in Additional file 2).

Discussion

In our two parallel online randomised controlled trials, none of the interventions (pictograph, bar graph, and line graph) were statistically significantly superior to text-only. There were no clinically meaningful differences in comprehension between the groups that viewed the bar graph, pictograph, or line graph. After all visual presentations were revealed to all participants, the bar graph was the most preferred option for receiving prognostic information in the future; chosen by about one-third of participants in both trials. The type of visual presentation viewed did not appear to influence change in decision intention.

For peer review only - http://bmjopen.bmj.com/site/about/guidelines.xhtml

The results from our systematic review found no clear evidence to support the superiority of any particular visual presentation to communicate prognosis, with existing research mostly investigating the communication of long-term prognosis for various types of cancer.[6] In our current trials, using non-cancer conditions and shorter prognostic duration, the difference between the various graph types on comprehension or decision intention was not significant. We found some minor inconsistencies between participants' preferred graph type and their ratings of satisfaction and ease of use of the various graphs. It appears that no graph type is clearly superior at facilitating comprehension, strongly preferred by participants, and viewed as the easiest to understand. Prior to the widespread use of a graph to convey prognostic information, piloting it with the target audience (ensuring a range of health literacy levels) may help to ensure appropriateness.

Several reviews and primary studies of methods of communicating treatment or screening benefits and harms have recommended adding visual presentations to textual information when communicating quantitative data.[13 22 34 35] For example, a test of three different visual presentations (text-only, fact box, visual aid) for communicating mortality with and without screening for prostate and breast cancer found that visual aids (i.e., a pictograph) generally increased comprehension by 18%.[24] However, in our trials, we found that the pictograph was superior to text-only, but only in trial A (AOM scenario). Reasons for this are unclear and might be explained by more complicated prognostic information, and more treatment options, were presented in trial B (e.g., the prognostic course was more variable at the different time periods).

Visual presentations are often incorporated into patient decision aids, which are tools that can be used to support shared decision-making and contain quantitative

For peer review only - http://bmjopen.bmj.com/site/about/guidelines.xhtml

information about the benefits and harms of options for managing a condition. The International Patient Decision Aids Standards (IPDAS) collaboration recently reviewed the evidence about using and displaying numbers when designing patient decision aids and recommended adding a visual presentation (e.g., pictographs, bar charts) to communicate quantitative information, without specific recommendation about which type to use.[34]

Line graphs have traditionally been avoided when communicating with patients as it has been assumed they are too difficult to understand[18], however, our study did not find significantly poor comprehension in participants in the line graph group. These findings suggest line graphs are an appropriate graph that might be used to communicate prognostic information. As showing the trajectory of the condition over time is often very important, a line graph may be best suited to use when there are outcome data for more than two time points, as the equivalent information would need multiple pictographs (one at each time point) and the trajectory less clear.

Communicating quantitative information can be more challenging when multiple management options are available or multiple important outcomes. Further studies are needed to investigate how to best visually, and verbally, communicate more complex prognostic information.

There were potential limitations to these two trials; participants were members of an online survey provider and did not need to have experience with the conditions studied, and only spent about 15-20 minutes engaged in completing the survey. We were not able to assess participants' baseline knowledge as the comprehension questions asked for the exact numbers which we expected to be inapplicable for participants without showing them some information. The impact of prior experience,

For peer review only - http://bmjopen.bmj.com/site/about/guidelines.xhtml

or lack of, is unclear as previous experience may have influenced decision intention, whereas no experience or personal relevance may have reduced engagement. As the questions about decision intention were hypothetical, motivation to properly read the information presented and consider the decision options may have been low. Additionally, to make a well-informed choice, patients need information about both the benefits and harms of each available treatment option. We did not present harms data as part of the information, and this may have influenced responses to questions about decision intention. Although we chose two conditions that are typically managed in primary care settings, our findings should be interpreted with caution and might not apply to the communication of other health conditions and might only apply to a setting where participants are to interpret prognostic information by themselves, rather than when the information is presented as part of face-to-face communication.

Our trials have several strengths, including testing the visual presentations in two conditions with different trajectories and conducting the trials in parallel. Measures were used to prevent cross contamination between the trials (e.g., participants of trial A were not invited to trial B, and participants of the piloting phase were not invited to participate in any of the trials).

Conclusion

Our findings suggest that, if the information is clearly displayed, any of the tested visual presentations can be used to communicate quantitative prognostic information.

Ethics approval and consent to participate

For peer review only - http://bmjopen.bmj.com/site/about/guidelines.xhtml

This study received ethical approval from Bond University Human Research Ethics Committee (EA00088). All methods in this study were performed in accordance with the Declaration of Helsinki. Informed consent was obtained from all Participants to receive online surveys by Dynata survey company. Additionally, before starting the survey, participants were provided with a formal invitation to participate in the study which included information about the study, and their right to withdraw at any time point with no negative consequences.

Consent for publication

Not applicable

Availability of data and materials

The datasets (raw data) and statistical codes generated during the current study are available in the Open Science Framework repository (OSF).[32]

All data analysed during this study are included in this published article and its supplementary information files. (Additional files 1, 2).

A copy of the registered protocol is provided as a supplementary.

Authors' contributions

EA, TH, and CDM conceived the idea of this study. EA developed the interventions with support from TH, CDM and MB. MB helped in the refinement of pictographs intervention. EA, TH and CDM developed the survey. TH was reviewing and giving feedback on all the steps. Data collection was managed by Dynata under the direction of EA, TH. Data were analysed by EA, MJ, and MB. EA drafted the main manuscript and developed all tables and figures with support and feedback from all

For peer review only - http://bmjopen.bmj.com/site/about/guidelines.xhtml

For peer review only

authors. All authors reviewed the final version of the manuscript and approved it for submission.

Competing interests

None declared

Funding

This work was supported by the Centre for Research Excellence in Minimizing Antibiotic Resistance in the Community (CRE-MARC), funded by the Australian National Health and Medical Research Council grant number (1153299).

Acknowledgements

We thank our colleagues at the Institute of Evidence-Based Healthcare (IEBH)-Bond University, for giving their feedback on the interventions and testing the survey during the pre-piloting phase. A special thank you to the childcare teachers who pilot-tested Trial A surveys and to Bond University members who pilot-tested Trial B surveys.

This work is dedicated to the memory of Professor Chris Del Mar, one of my PhD supervisors, who contributed fully to this work (passed away in March 2022).

For peer review only

References

1. Charles C, Gafni A, Whelan T. Shared decision-making in the medical encounter: what does it mean? (or it takes at least two to tango). *Soc Sci Med* 1997;44(5):681-92. doi: 10.1016/s0277-9536(96)00221-3 [published Online First: 1997/03/01]
2. Feemster LC, Curtis JR. "We Understand the Prognosis, but We Live with Our Heads in the Clouds": Understanding Patient and Family Outcome Expectations and Their Influence on Shared Decision Making. *Am J Respir Crit Care Med* 2016;193(3):239-41. doi: 10.1164/rccm.201511-2125ED [published Online First: 2016/02/02]
3. Hoffmann TC, Del Mar C. Patients' expectations of the benefits and harms of treatments, screening, and tests: a systematic review. *JAMA Intern Med* 2015;175(2):274-86. doi: 10.1001/jamainternmed.2014.6016
4. Paladino J, Lakin JR, Sanders JJ. Communication Strategies for Sharing Prognostic Information With Patients: Beyond Survival Statistics. *JAMA* 2019 doi: 10.1001/jama.2019.11533 [published Online First: 2019/08/16]
5. Feldman-Stewart D, Brundage MD, Zotov V. Further insight into the perception of quantitative information: judgments of gist in treatment decisions. *Med Decis Making* 2007;27(1):34-43. doi: 10.1177/0272989X06297101 [published Online First: 2007/01/24]
6. Abukmail E, Bakhit M, Del Mar C, et al. Effect of different visual presentations on the comprehension of prognostic information: a systematic review. *BMC Med Inform Decis Mak* 2021;21(1):249. doi: 10.1186/s12911-021-01612-9 [published Online First: 2021/08/27]
7. Rovers MM, Zielhuis GA. Otitis media meta-analysis. *Pediatrics* 2004;114(2):508-9; author reply 08-9. doi: 10.1542/peds.114.2.508-a [published Online First: 2004/08/03]
8. Venekamp RP, Sanders SL, Glasziou PP, et al. Antibiotics for acute otitis media in children. *Cochrane Database Syst Rev* 2015(6):CD000219. doi: 10.1002/14651858.CD000219.pub4 [published Online First: 2015/06/24]
9. Bisset L, Beller E, Jull G, et al. Mobilisation with movement and exercise, corticosteroid injection, or wait and see for tennis elbow: randomised trial. *BMJ* 2006;333(7575):939. doi: 10.1136/bmj.38961.584653.AE [published Online First: 2006/10/03]
10. Lipkus IM, Hollands JG. The visual communication of risk. *J Natl Cancer Inst Monogr* 1999(25):149-63. doi: 10.1093/oxfordjournals.jncimonographs.a024191 [published Online First: 2000/06/16]
11. Fortin JM, Hirota LK, Bond BE, et al. Identifying patient preferences for communicating risk estimates: a descriptive pilot study. *BMC Med Inform Decis Mak* 2001;1:2. doi: 10.1186/1472-6947-1-2 [published Online First: 2001/09/08]

For peer review only - http://bmjopen.bmj.com/site/about/guidelines.xhtml

12. Armstrong K, Schwartz JS, Fitzgerald G, et al. Effect of framing as gain versus loss on understanding and hypothetical treatment choices: survival and mortality curves. *Med Decis Making* 2002;22(1):76-83. doi: 10.1177/0272989X0202200108 [published Online First: 2002/02/09]

13. Ancker JS, Senathirajah Y, Kukafka R, et al. Design features of graphs in health risk communication: a systematic review. *J Am Med Inform Assoc* 2006;13(6):608-18. doi: 10.1197/jamia.M2115 [published Online First: 2006/08/25]

14. Feldman-Stewart D, Brennenstuhl S, Brundage MD. A purpose-based evaluation of information for patients: an approach to measuring effectiveness. *Patient Educ Couns* 2007;65(3):311-9. doi: 10.1016/j.pec.2006.08.012 [published Online First: 2006/10/04]

15. Zikmund-Fisher BJ, Fagerlin A, Ubel PA. Mortality versus survival graphs: improving temporal consistency in perceptions of treatment effectiveness. *Patient Educ Couns* 2007;66(1):100-7. doi: 10.1016/j.pec.2006.10.013 [published Online First: 2006/12/02]

16. Zikmund-Fisher BJ, Fagerlin A, Ubel PA. Improving understanding of adjuvant therapy options by using simpler risk graphics. *Cancer* 2008;113(12):3382-90. doi: 10.1002/cncr.23959 [published Online First: 2008/11/18]

17. Zikmund-Fisher BJ, Witteman HO, Dickson M, et al. Blocks, ovals, or people? Icon type affects risk perceptions and recall of pictographs. *Med Decis Making* 2014;34(4):443-53. doi: 10.1177/0272989X13511706 [published Online First: 2013/11/20]

18. Carling CL, Kristoffersen DT, Flottorp S, et al. The effect of alternative graphical displays used to present the benefits of antibiotics for sore throat on decisions about whether to seek treatment: a randomized trial. *PLoS Med* 2009;6(8):e1000140. doi: 10.1371/journal.pmed.1000140 [published Online First: 2009/08/27]

19. Zikmund-Fisher BJ, Fagerlin A, Ubel PA. A demonstration of ''less can be more'' in risk graphics. *Med Decis Making* 2010;30(6):661-71. doi: 10.1177/0272989X10364244 [published Online First: 2010/04/09]

20. Brown SM, Culver JO, Osann KE, et al. Health literacy, numeracy, and interpretation of graphical breast cancer risk estimates. *Patient Educ Couns* 2011;83(1):92-8. doi: 10.1016/j.pec.2010.04.027 [published Online First: 2010/06/18]

21. Trevena LJ, Zikmund-Fisher BJ, Edwards A, et al. Presenting quantitative information about decision outcomes: a risk communication primer for patient decision aid developers. *BMC Med Inform Decis Mak* 2013;13 Suppl 2:S7. doi: 10.1186/1472-6947-13-S2-S7 [published Online First: 2013/01/01]

22. Zipkin DA, Umscheid CA, Keating NL, et al. Evidence-based risk communication: a systematic review. *Ann Intern Med* 2014;161(4):270-80. doi: 10.7326/M14-0295 [published Online First: 2014/08/19]

23. Hamstra DA, Johnson SB, Daignault S, et al. The impact of numeracy on verbatim knowledge of the longitudinal risk for prostate cancer recurrence following radiation therapy. *Med Decis Making* 2015;35(1):27-36. doi: 10.1177/0272989X14551639 [published Online First: 2014/10/04]

24. Petrova D, Garcia-Retamero R, Cokely ET. Understanding the Harms and Benefits of Cancer Screening: A Model of Factors That Shape Informed Decision Making. *Med Decis Making* 2015;35(7):847-58. doi: 10.1177/0272989X15587676 [published Online First: 2015/06/06]

25. Kasper J, van de Roemer A, Pottgen J, et al. A new graphical format to communicate treatment effects to patients-A web-based randomized controlled trial. *Health Expect* 2017;20(4):797-804. doi: 10.1111/hex.12522 [published Online First: 2016/12/17]

26. McDowell M, Gigerenzer G, Wegwarth O, et al. Effect of Tabular and Icon Fact Box Formats on Comprehension of Benefits and Harms of Prostate Cancer Screening: A Randomized Trial. *Med Decis Making* 2019;39(1):41-56. doi: 10.1177/0272989X18818166 [published Online First: 2019/02/26]

For peer review only - http://bmjopen.bmj.com/site/about/guidelines.xhtml

27. Brick C, McDowell M, Freeman ALJ. Risk communication in tables versus text: a registered report randomized trial on 'fact boxes'. *R Soc Open Sci* 2020;7(3):190876. doi: 10.1098/rsos.190876 [published Online First: 2020/04/10]
28. Scherer LD, Caverly TJ, Burke J, et al. Development of the Medical Maximizer-Minimizer Scale. *Health Psychol* 2016;35(11):1276-87. doi: 10.1037/hea0000417 [published Online First: 2016/10/18]
29. Fagerlin A, Zikmund-Fisher BJ, Ubel PA, et al. Measuring numeracy without a math test: development of the Subjective Numeracy Scale. *Med Decis Making* 2007;27(5):672-80. doi: 10.1177/0272989X07304449 [published Online First: 2007/07/21]
30. Zikmund-Fisher BJ, Smith DM, Ubel PA, et al. Validation of the Subjective Numeracy Scale: effects of low numeracy on comprehension of risk communications and utility elicitations. *Med Decis Making* 2007;27(5):663-71. doi: 10.1177/0272989X07303824 [published Online First: 2007/07/27]
31. Weiss BD, Mays MZ, Martz W, et al. Quick assessment of literacy in primary care: the newest vital sign. *Ann Fam Med* 2005;3(6):514-22. doi: 10.1370/afm.405 [published Online First: 2005/12/13]
[dataset] 32. Abukmail E, Hoffmann, T., Bakhit, M., Jones, M., & Del Mar, C. Data from: Effect of different visual presentations on the public's comprehension of prognostic information using acute and chronic condition scenarios: Two online randomised controlled trials. , 2021, October.
33. Woloshin S, Schwartz LM. Communicating data about the benefits and harms of treatment: a randomized trial. *Ann Intern Med* 2011;155(2):87-96. doi: 10.7326/0003-4819-155-2-201107190-00004 [published Online First: 2011/07/20]
34. Trevena LJ, Bonner C, Okan Y, et al. Current Challenges When Using Numbers in Patient Decision Aids: Advanced Concepts. *Med Decis Making* 2021:272989X21996342. doi: 10.1177/0272989X21996342 [published Online First: 2021/03/05]
35. Garcia-Retamero R, Galesic M. Who profits from visual aids: overcoming challenges in people's understanding of risks [corrected]. *Soc Sci Med* 2010;70(7):1019-25. doi: 10.1016/j.socscimed.2009.11.031 [published Online First: 20100128]

For peer review only

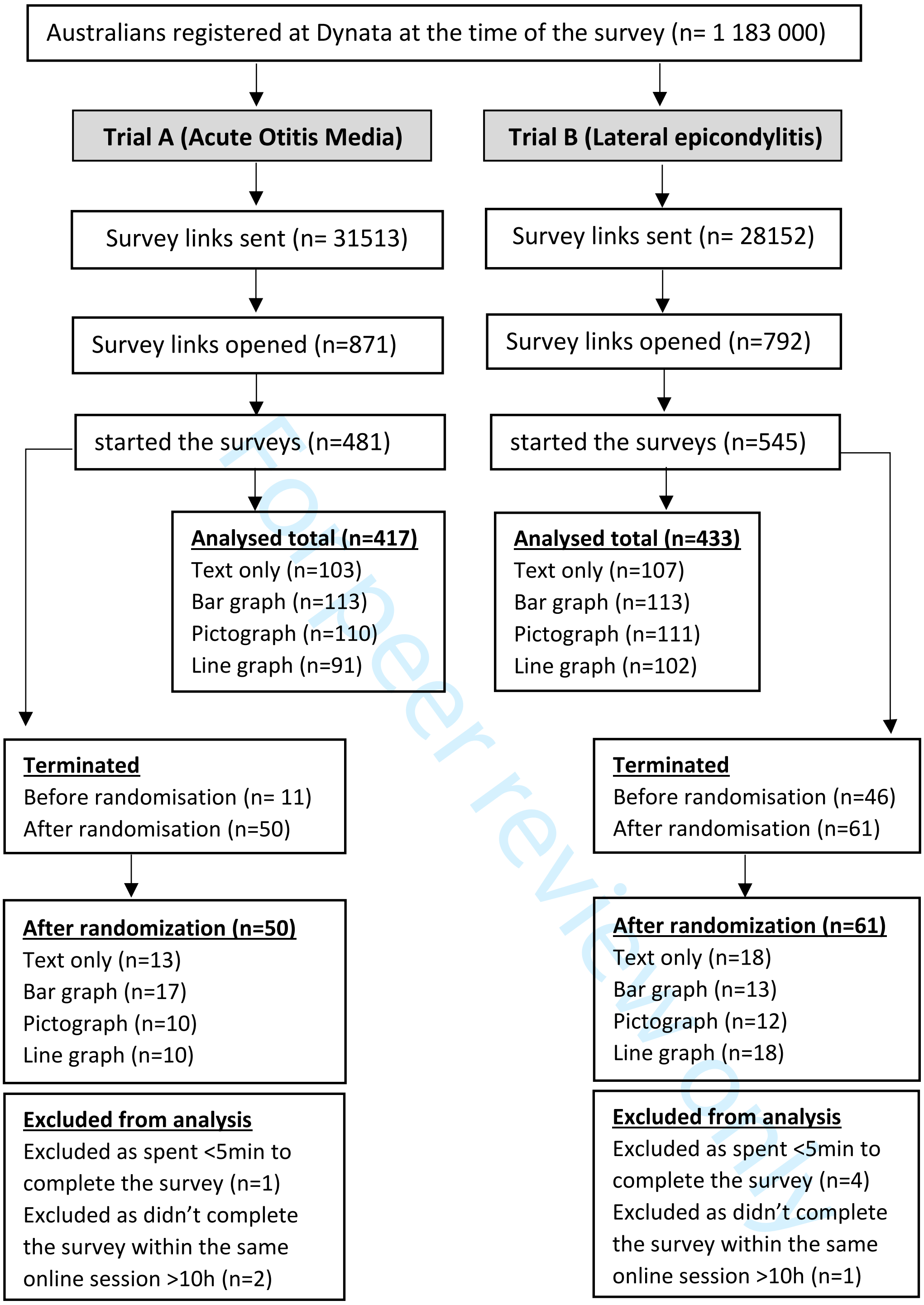

Figure 1: CONSORT flow diagrams of participants in Trial A and Trial B.

For peer review only

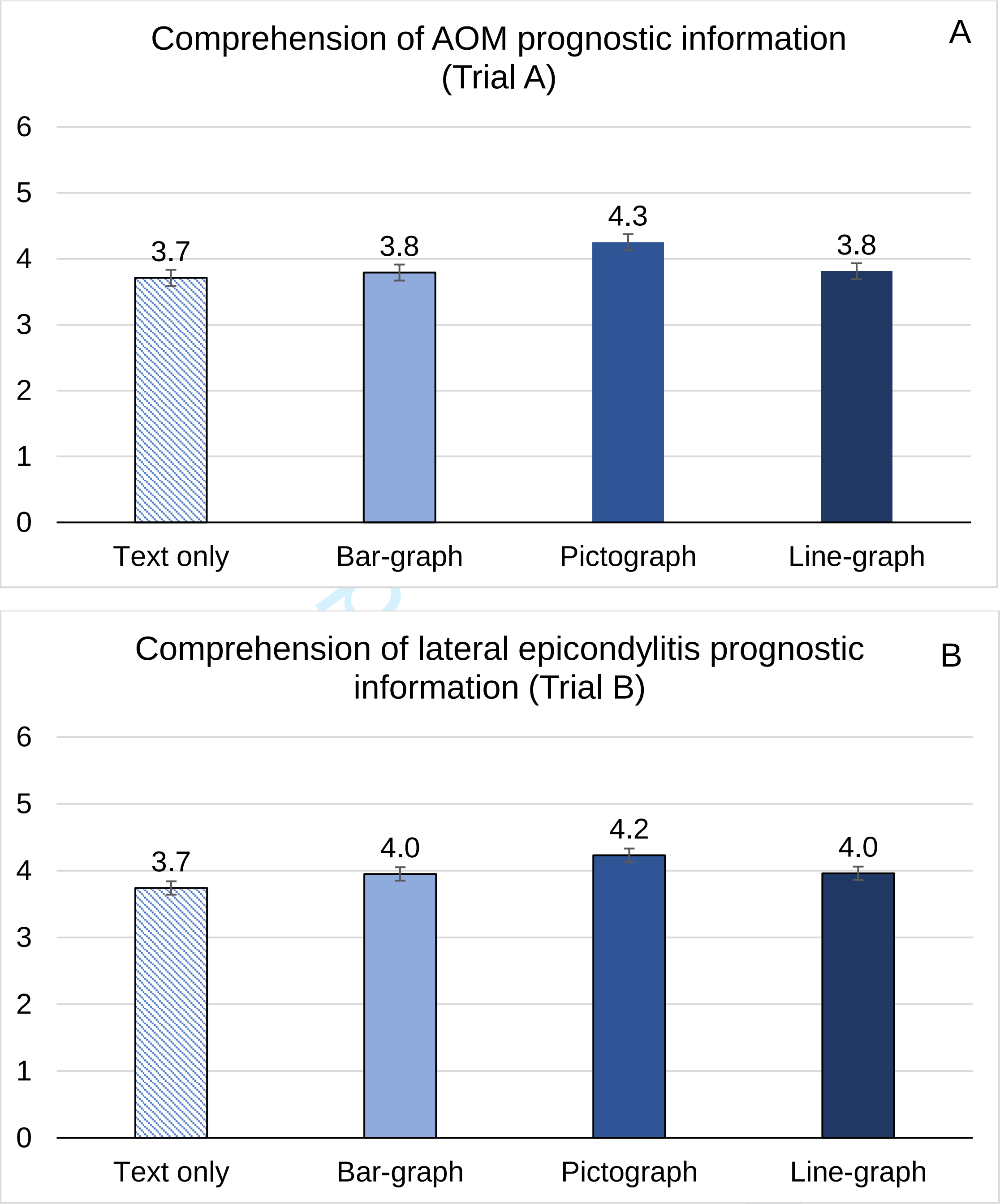

Figure 2: Mean comprehension score of prognostic information per group in trial A (AOM) and trial B (lateral epicondylitis). Comprehension questions are provided in additional file 1. Trial A and B comprehension questions are different.

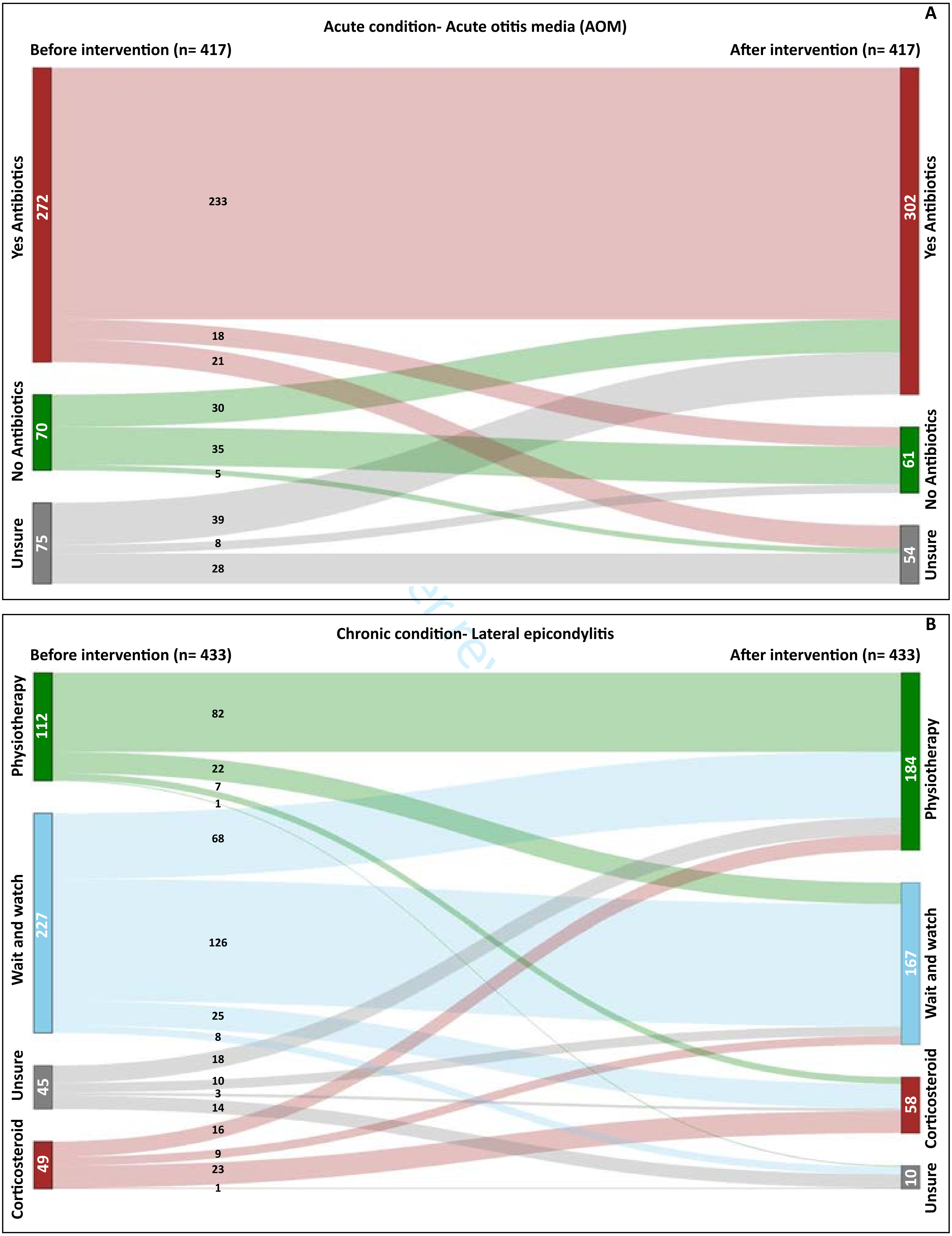

Figure 3: Overall change in treatment decision intentions before and after viewing the interventions.
The figure shows the number of participants who chose each management option in (A) acute condition-AOM and (B) chronic condition-lateral epicondylitis.

Additional file 1

(Supplementary methods)

CONSORT checklist 2

Dynata (demographics in Australia, recruitment strategies) 5

Interventions (visual presentations)

Trial A (Acute otitis media) 6

Trial B (Lateral epicondylitis)........ 9

The flow of the trials and outcome measurements 11

Survey 12

For peer review only

For peer review only - http://bmjopen.bmj.com/site/about/guidelines.xhtml

CONSORT 2010 checklist of information to include when reporting a randomised trial*

Section/Topic	Item No	Checklist item	Reported on page No
**Title and abstract**			
	1a	Identification as a randomised trial in the title	1
	1b	Structured summary of trial design, methods, results, and conclusions (for specific guidance see CONSORT for abstracts)	2-3
**Introduction**			
Background and objectives	2a	Scientific background and explanation of rationale	4-5
	2b	Specific objectives or hypotheses	5
**Methods**			
Trial design	3a	Description of trial design (such as parallel, factorial) including allocation ratio	5
	3b	Important changes to methods after trial commencement (such as eligibility criteria), with reasons	9
Participants	4a	Eligibility criteria for participants	5
	4b	Settings and locations where the data were collected	5
Interventions	5	The interventions for each group with sufficient details to allow replication, including how and when they were actually administered	6-7
Outcomes	6a	Completely defined pre-specified primary and secondary outcome measures, including how and when they were assessed	7-8
	6b	Any changes to trial outcomes after the trial commenced, with reasons	NA
Sample size	7a	How sample size was determined	8
	7b	When applicable, explanation of any interim analyses and stopping guidelines	NA
Randomisation:			
Sequence generation	8a	Method used to generate the random allocation sequence	6
	8b	Type of randomisation; details of any restriction (such as blocking and block size)	6
Allocation concealment mechanism	9	Mechanism used to implement the random allocation sequence (such as sequentially numbered containers), describing any steps taken to conceal the sequence until interventions were assigned	6
Implementation	10	Who generated the random allocation sequence, who enrolled participants, and who assigned participants to interventions	6

For peer review only

For peer review only - http://bmjopen.bmj.com/site/about/guidelines.xhtml

Blinding	11a	If done, who was blinded after assignment to interventions (for example, participants, care providers, those assessing outcomes) and how	10
	11b	If relevant, description of the similarity of interventions	6-7
Statistical methods	12a	Statistical methods used to compare groups for primary and secondary outcomes	9
	12b	Methods for additional analyses, such as subgroup analyses and adjusted analyses	9-10
**Results**			
Participant flow (a diagram is strongly recommended)	13a	For each group, the numbers of participants who were randomly assigned, received intended treatment, and were analysed for the primary outcome	Figure 3
	13b	For each group, losses and exclusions after randomisation, together with reasons	Figure 3
Recruitment	14a	Dates defining the periods of recruitment and follow-up	5
	14b	Why the trial ended or was stopped	NA
Baseline data	15	A table showing baseline demographic and clinical characteristics for each group	Table 1
Numbers analysed	16	For each group, number of participants (denominator) included in each analysis and whether the analysis was by original assigned groups	Tables and figures
Outcomes and estimation	17a	For each primary and secondary outcome, results for each group, and the estimated effect size and its precision (such as 95% confidence interval)	10-14, and tables
	17b	For binary outcomes, presentation of both absolute and relative effect sizes is recommended	NA
Ancillary analyses	18	Results of any other analyses performed, including subgroup analyses and adjusted analyses, distinguishing pre-specified from exploratory	Additional file 2
Harms	19	All important harms or unintended effects in each group (for specific guidance see CONSORT for harms)	Ethics application
**Discussion**			
Limitations	20	Trial limitations, addressing sources of potential bias, imprecision, and, if relevant, multiplicity of analyses	14-15
Generalisability	21	Generalisability (external validity, applicability) of the trial findings	15-16
Interpretation	22	Interpretation consistent with results, balancing benefits and harms, and considering other relevant evidence	14-17
**Other information**			

For peer review only - http://bmjopen.bmj.com/site/about/guidelines.xhtml

Registration	23	Registration number and name of trial registry	5
Protocol	24	Where the full trial protocol can be accessed, if available	In the registry
Funding	25	Sources of funding and other support (such as supply of drugs), role of funders	18

*We strongly recommend reading this statement in conjunction with the CONSORT 2010 Explanation and Elaboration for important clarifications on all the items. If relevant, we also recommend reading CONSORT extensions for cluster randomised trials, non-inferiority and equivalence trials, non-pharmacological treatments, herbal interventions, and pragmatic trials. Additional extensions are forthcoming: for those and for up to date references relevant to this checklist, see www.consort-statement.org.

For peer review only

Dynata (demographics in Australia, recruitment strategies)

(Screenshots were taken from the information documents Dynata shared with the researchers at the start of the study and gave permission to share)

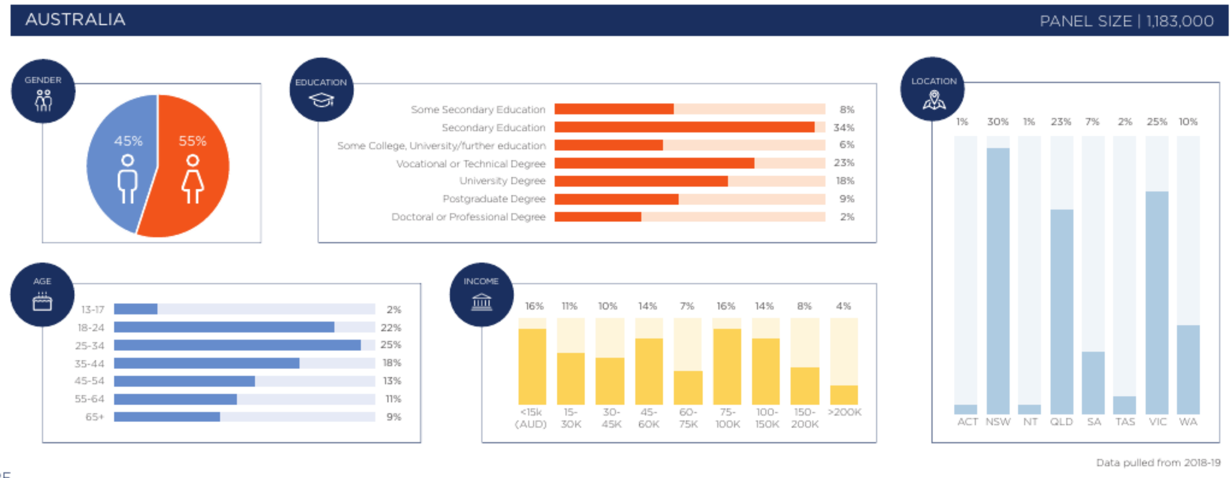

Recruitment Methodologies

SOURCE NAME	RECRUITMENT
Loyalty Panels	By invitation to 1000's of loyalty program members in travel, entertainment, media and retail.
Organic, Open Enrolment & Partnerships	1000's of websites, social media influencers and mobile apps.
Affiliate Network	Broad range of websites, incl. schools, communities. Member logging into communities with valid username and password invited to participate.

For peer review only - http://bmjopen.bmj.com/site/about/guidelines.xhtml

Interventions (visual presentations)

Trial A (Acute otitis media)

Pictograph

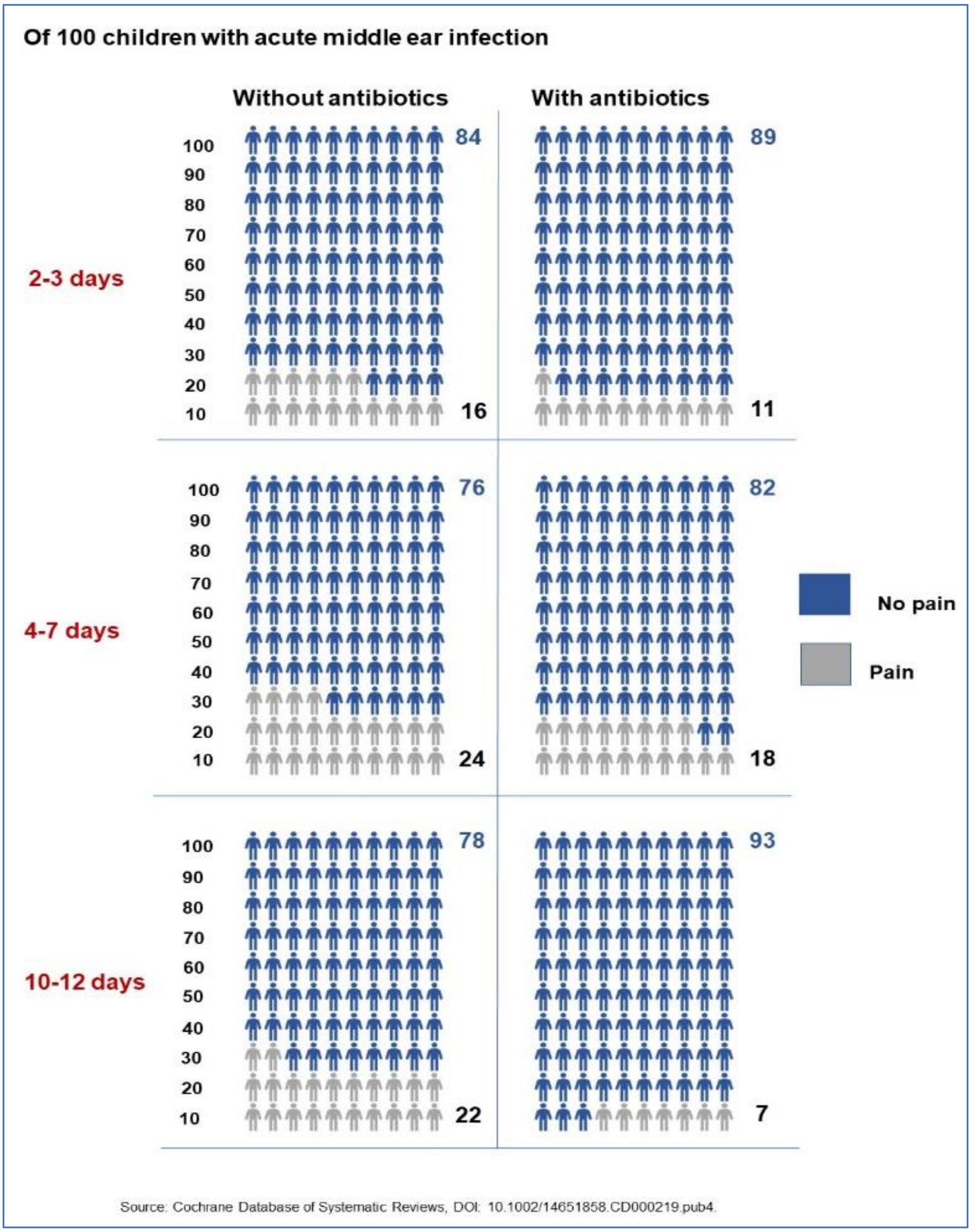

Bar graph

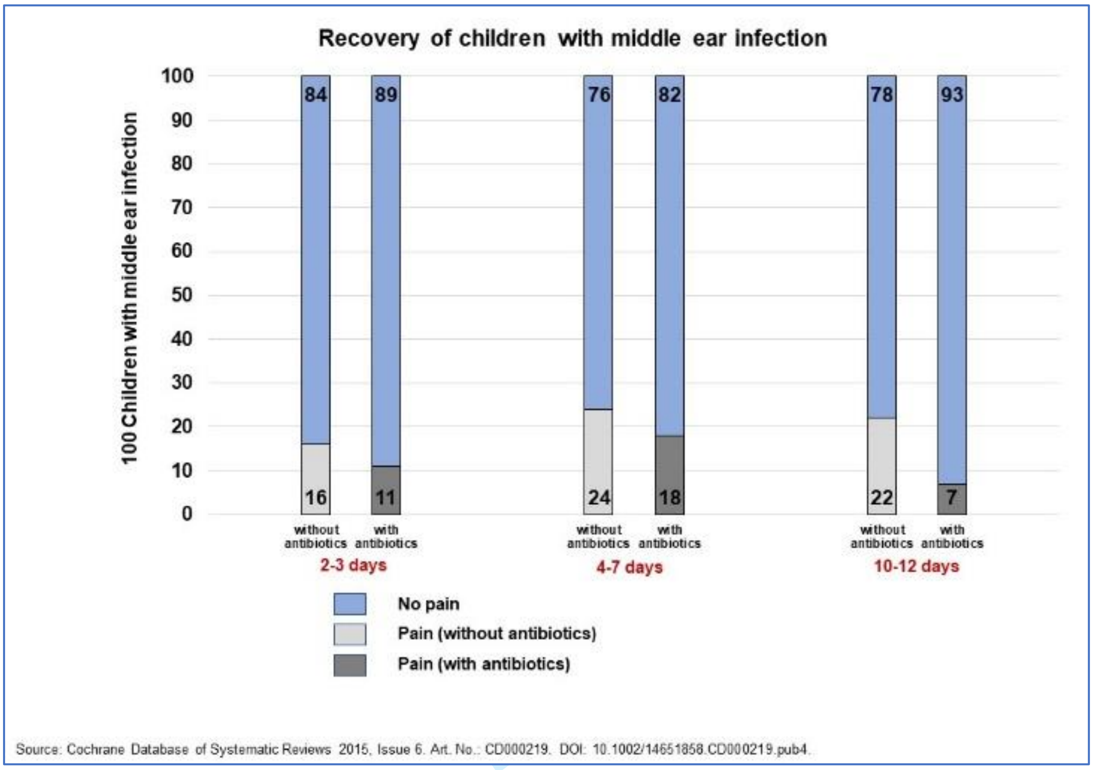

Text-only

Recovery of children with middle ear infection

Of 100 children who have middle ear infection:

Without antibiotic treatment

16 children will still experience pain at **2-3 days** after diagnosis. 24 children will have pain at **4-7 days** and 22 children will have pain at **10-12 days**. The others will have no pain.

With antibiotic treatment

11 children will still experience pain at **2-3 days** after diagnosis, 18 children will have pain at **4-7 days** and 7 children will have pain at **10-12 days**. The others will have no pain.

Source: Cochrane Database of Systematic Reviews 2015, Issue 6. Art. No.: CD000219. DOI: 10.1002/14651858.CD000219.pub4.

For peer review only - http://bmjopen.bmj.com/site/about/guidelines.xhtml

Line graph

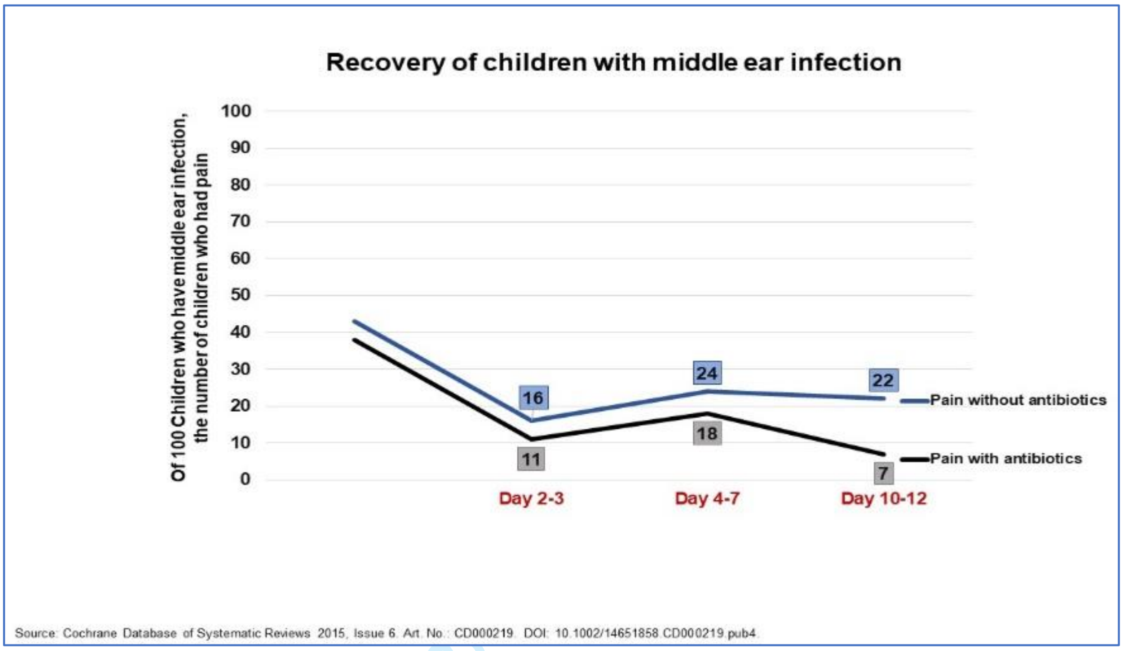

For peer review only - http://bmjopen.bmj.com/site/about/guidelines.xhtml

Trial B (Lateral epicondylitis)

Pictograph

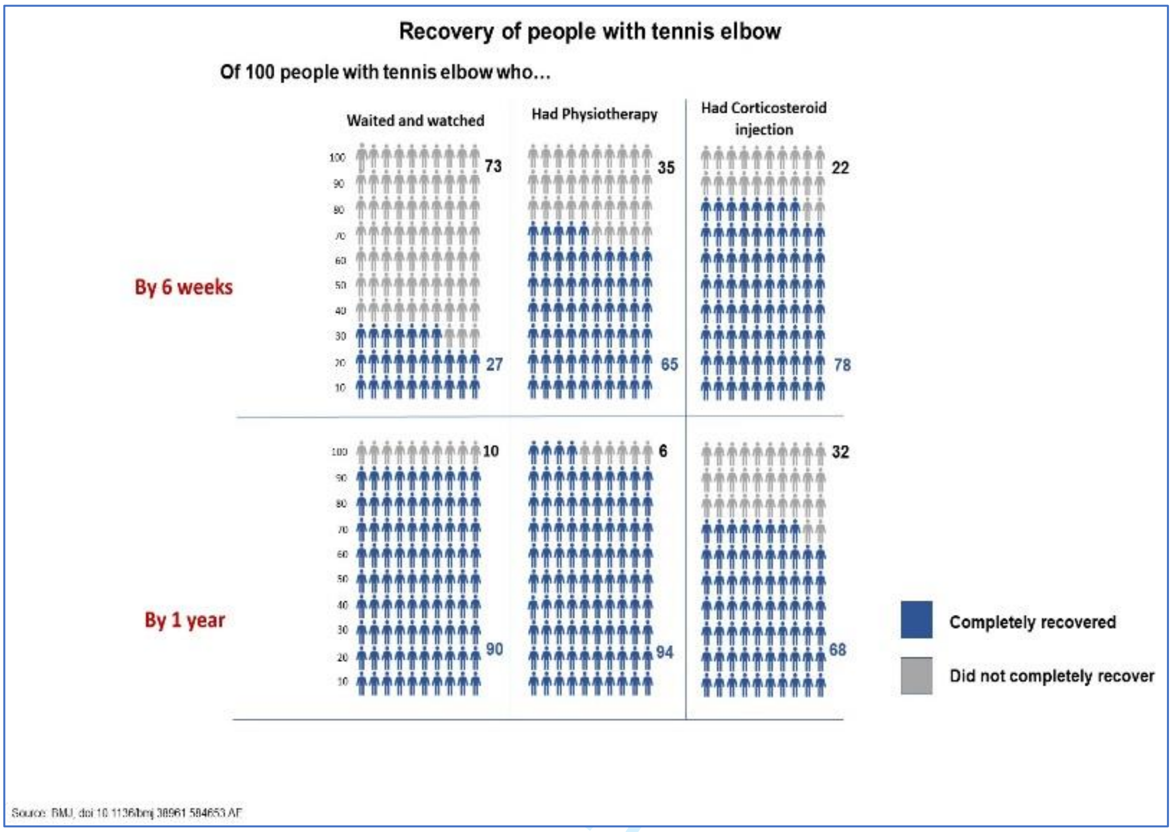

Text-only

Recovery of people with tennis elbow

Of 100 people with tennis elbow who:

Waited and watched (to see if it gets better on its own)

27 people completely recovered **by 6 weeks**. **By 1 year**, 90 people completely recovered; the other 10 did not completely recover.

Had physiotherapy

65 people completely recovered **by 6 weeks**. **By 1 year**, 94 people completely recovered; the other 6 did not completely recover.

Had corticosteroid injection

78 people completely recovered **by 6 weeks**. **By 1 year**, 68 people completely recovered; the other 32 did not completely recover.

Source: BMJ, doi:10.1136/bmj.38961.584653.AE

Bar graph

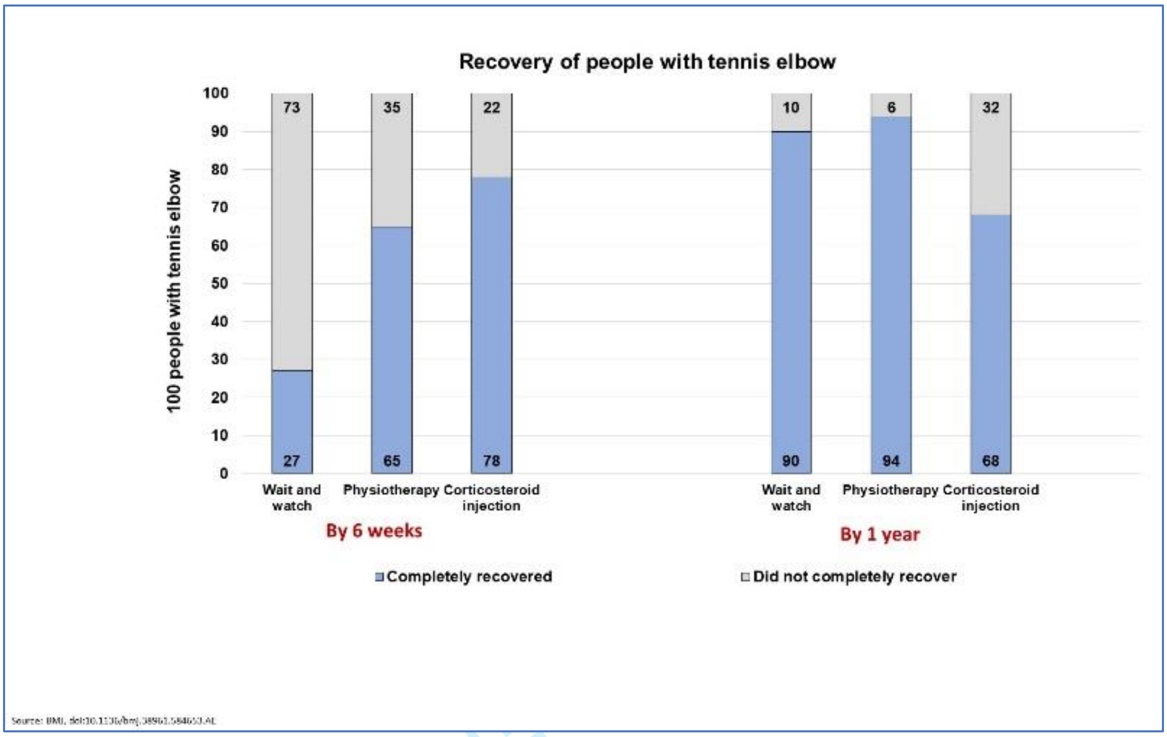

Line graph

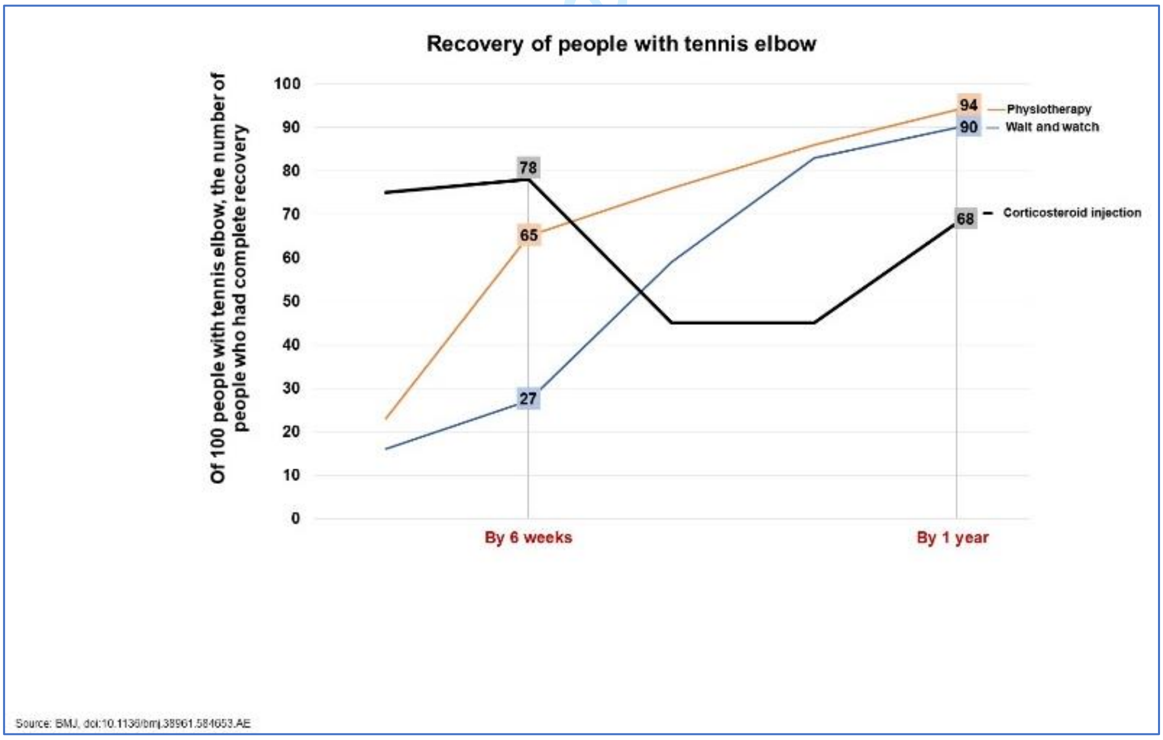

The flow of the trials and outcome measurements

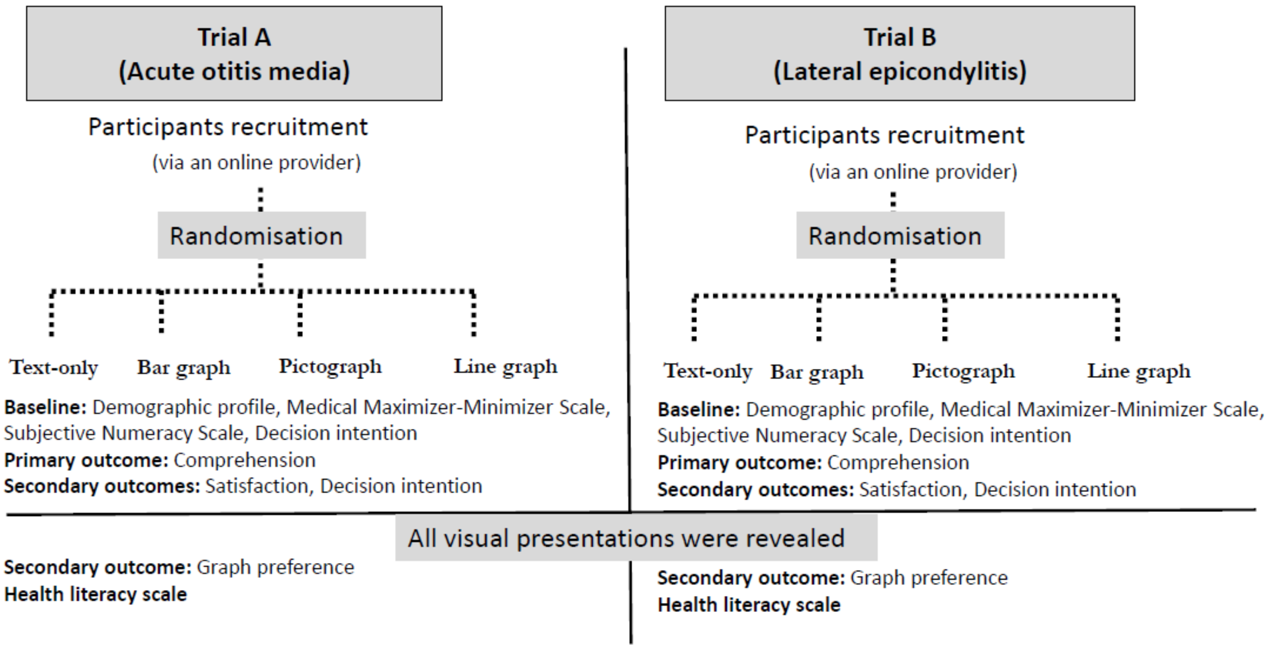

view only

For peer review only - http://bmjopen.bmj.com/site/about/guidelines.xhtml

Survey

Questions 1-6 are the same for both trials

1. Which age group applies to you?

[ ] Under 18 [ ] 18 to 24 years [ ] 25 to 34 years
[ ] 35 to 44 years [ ] 45 to 54 years [ ] 55 to 64 years
[ ] 65 years or older [ ] Prefer not to say

2. What is your gender?

[ ] Male [ ] Female
[ ] Other [ ] Prefer not to say

3. What is your level of education? Please indicate the highest level of education you have completed.

[ ] High school - Year 10
[ ] High school – Year 12
[ ] Certificate I-IV, Diploma, or apprenticeship
[ ] Undergraduate degree: Bachelor's degree, or equivalent
[ ] Postgraduate degree: Masters or Doctoral degree, or equivalent

4. Which state or territory do you live in? [PUNCH STATE FROM POSTCODE- OKAY TO MOVE THIS AFTER Q5 IN SCRIPT- DO NOT ASK RESPONDENTS]

[ ] QLD [ ] NSW [ ] ACT [ ] NT [ ] WA [ ] VIC
[ ] TAS [ ] SA

5. What is your postcode? ___________

6. Is English the main language that you speak at home?

[ ] Yes [ ] No

Questions 7 and 8 are specific for each trial

Trial A (Acute otitis media)

7. Acute middle ear infection is an infection behind the eardrum. It is very common, usually in children, and can be very painful. You can take any pain medicine with any of the options below. Imagine that a child you take care of gets a middle ear infection, what decision about using antibiotics might you make?

[ ] The child usually should take antibiotics.
[ ] The child usually should not take antibiotics.
[ ] Unsure

8. Have you, or a person that you are responsible for, previously had an acute middle ear infection?

[ ] Yes
[ ] No

Trial B (lateral epicondylitis)

7. Tennis elbow is a condition in which people have pain and tenderness over the outside of the elbow It usually gets better by itself, regardless of the type of treatment, but symptoms may last up to 2 years[1].

---

[1] Source: Olaussen M, Holmedal O, Lindbaek M, et al Treating lateral epicondylitis with corticosteroid injections or non -electrotherapeutical physiotherapy: a systematic review BMJ Open 2013;3:e003564. doi: 10.1136/bmjopen-2013-003564

For peer review only - http://bmjopen.bmj.com/site/about/guidelines.xhtml

You can take any pain medicine with any of the options below. Imagine that you have tennis elbow, what decision about other treatment options, from the below list, might you make?

[ ] I would probably have a corticosteroid injection.
[ ] I would probably have physiotherapy.
[ ] I would probably wait and watch to see if it gets better on its own.
[ ] Unsure

8. Have you, or a person that you know, previously had tennis elbow (lateral epicondylitis)?

[ ] Yes [ ] No

Questions 9 -11 are the same for both trials (Q9 is MMM scale, Q10 and 11 are Subjective Numeracy Scale)

Comprehension questions Q12-Q17 (These questions are different for trial A and trial B)

Trial A (Acute otitis media) only

Middle ear infection is one of the most frequent diseases in childhood. Children usually experience symptoms of middle ear inflammation like pain, ear discharge, and fever.[2]

The numbers below show what happens over time to children up to 15 years of age with middle ear infection when different ways of managing it are chosen.

The intervention was inserted here

Based on the information in the XXXX (please insert the type of graph e.g., pictograph), please answer the following questions. (Used for all graph interventions)

Based on the information in the text box, please answer the following questions. (Used for all text interventions):

(Q12-Q17- ON SAME PAGE ALONG WITH THE INTERVENTION).

12. At 2-3 days, out of 100 children who did NOT take antibiotics, how many had pain?

Answer: ___________

13. At 10-12 days, out of 100 children who took antibiotics, how many had pain?

Answer: ___________

14. Which group was less likely to experience pain at about 4-7 days?

[ ] Children who took antibiotics
[ ] Children who did not take antibiotics.
[ ] No difference between the groups.
[ ] I do not know.

15. At 4-7 days, how many more children who did NOT take antibiotics will have pain, compared to those who took antibiotics?

Answer: ___________

[2] Source: Cochrane Database of Systematic Reviews 2015, Issue 6. Art. No.: CD000219. DOI: 10.1002/14651858.CD000219.pub4.

For peer review only - http://bmjopen.bmj.com/site/about/guidelines.xhtml

16. At 2-3 days, how many more/fewer children who did NOT take antibiotics will be pain-free, compared to children who did take antibiotics?

[ ] 5 fewer [ ] 5 more [ ] 16 more [ ] 11 fewer

17. In the week or so after a middle ear infection starts, the majority of children will NOT be in pain, regardless of whether they do or do not take antibiotics

[ ] True [ ] False

Trial B (Lateral epicondylitis) only

Tennis elbow is a condition in which people have pain and tenderness over the outside of the elbow. This can make some movements of the arm and wrist painful.

The numbers below show what happens over time to people with tennis elbow when different ways of managing it are chosen.

The intervention was inserted here

Based on the information in the XXXX (please insert the type of graph e.g., pictograph), please answer the following questions. (Used for all graph interventions)

Based on the information in the text box, please answer the following questions. (Used for all text interventions):

(Q12-Q17- ON SAME PAGE ALONG WITH THE INTERVENTION)

12. Of 100 people with tennis elbow who chose to 'wait and watch, how many people completely recovered by 6 weeks?

Answer: ___________

13. Of 100 people with tennis elbow who chose to have a corticosteroid injection, how many people completely recovered by 6 weeks?

Answer: ___________

14. How many <u>more</u> people who chose physiotherapy completely recovered by 6 weeks, compared to those who waited and watched?

Answer: ___________

15. Which group was <u>more likely</u> to be completely recovered by 1 year?

[ ] People who waited and watched
[ ] People who had physiotherapy
[ ] People who had a corticosteroid injection
[ ] No difference between the groups
[ ] I do not know

16. **By 1 year, how many more/ fewer people who had physiotherapy completely recovered, compared to people who had a corticosteroid injection?**

[ ] 26 fewer [ ] 26 more [ ] 32 more [ ] 6 fewer

17. **By 1 year, the majority of people who chose to wait and watch will be completely recovered, without having physiotherapy or a corticosteroid injection.**

[ ] True [ ] False

For peer review only - http://bmjopen.bmj.com/site/about/guidelines.xhtml

Question 18 is trial specific

Trial A

18. Imagine again that a child you take care of gets a middle ear infection. What decision about using antibiotics might you make?

[ ] The child usually should ***take*** antibiotics.

[ ] The child usually should ***not take*** antibiotics.

[ ] Unsure

Trial B

18. Imagine again that you have tennis elbow. What decision about the management option might you make?

[ ] I would probably have a corticosteroid injection.

[ ] I would probably have physiotherapy.

[ ] I would probably wait and watch to see if it gets better on its own.

[ ] Unsure

Questions 19-23 are the same for both trials

19. **Can you please indicate the most important reason for your decision?**

Questions 20-28 are the same for both trials

Trial A statement: You have just seen A XXXX-(PIPE IN TEXT AS PER GRAPH SHOWN pictograph bar graph, line graph, text) presenting information about the recovery of children with middle ear infection

Trial B statement: You have just seen a XXXX (PIPE IN TEXT AS PER GRAPH SHOWN pictograph bar graph, line graph, text) presenting information about the recovery of people with tennis elbow over time.

The allocated intervention was shown here again

Now we would like to ask you a few questions about ***how the information*** is presented.

20. In this presentation, how ***easy*** was it to understand the information presented? (From 1= Not at all easy to 10= extremely easy)
21. How ***satisfied*** are you with **HOW** the information is presented? (From 1= not at all satisfied to 10=I am totally satisfied)
22. **In the future, you may have to make a decision about managing a health condition. To help you make this decision you may like more information about how your health may change over time because of this condition (i.e. its prognosis). You have just seen A (PIPE IN TEXT AS PER GRAPH SHOWN pictograph bar graph, line graph, text) presenting prognostic information.**

For peer review only - http://bmjopen.bmj.com/site/about/guidelines.xhtml

We will now show other ways to you and ask you to rank these ways from the MOST preferred option (1) to the LEAST preferred option (4) for receiving this type of information in future. [SHOW ALL INTERVENTIONS ALONG WITH LABELS]

[ ] Text only
[ ] A bar graph
[ ] A pictograph
[ ] A line graph

23. In a few words, please tell us why you chose the option that you ranked the highest (1)?

24. Do you prefer another way of presenting prognostic information THAT IS NOT IN THE PREVIOUS LIST (e.g., a combination of text and a graph, a table, a combination of a table and a graph)?

Please note that these are just examples and please feel free to suggest any other ways you may prefer.

25. Please rate how much experience you have had with each of the following graphs before today: (Rating from 1= no experience to 5 = a lot of experience) (graphs were shown)

Questions 26 and 27 are the NVS health literacy scale

28. If you had any difficulties with the survey (reading, understanding questions, technical problems) or you have any comments to improve the survey, please note them here

Thank you for your time and for taking part in our survey

For peer review only

Additional file 2

(Supplementary results)

Comprehension results **2**

Equivalence comparisons of graph types **3**

Association of comprehension with HL, numeracy, and education level

Trial A (Acute otitis media) 4

Trial B (Lateral epicondylitis)........ 4

Change of decision intention by intervention

Trial A (Acute otitis media) 5

Trial B (Lateral epicondylitis)........ 7

Reasons for decision intention Q19

Trial A (Acute otitis media) 9

Trial B (Lateral epicondylitis)........ 12

Reasons for first choice preference Q23

Trial A (Acute otitis media) 16

Trial B (Lateral epicondylitis)........ 18

Exploratory results

Association of comprehension with MMM, time to complete comprehension question, and graph preference

Trial A (Acute otitis media) 20

Trial B (Lateral epicondylitis)........ 20

Visual presentation preference by previous graph experience

Trial A (Acute otitis media) 21

Trial B (Lateral epicondylitis)........ 21

For peer review only

Comprehension results

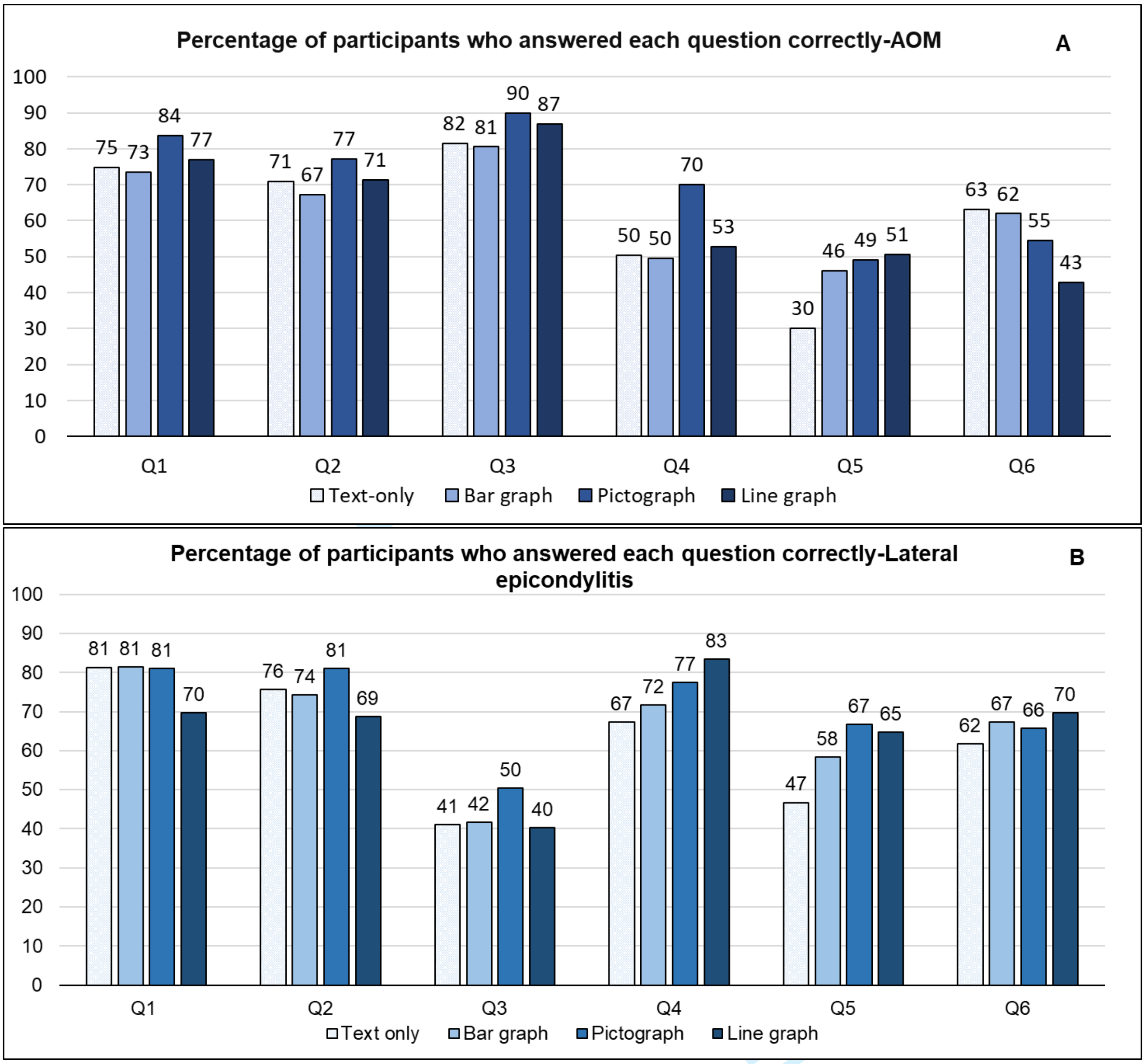

Comprehension of prognostic information. The numbers displayed represent the percentage of participants who answered each question correctly in (A) acute condition-AOM and (B) chronic condition-lateral epicondylitis. Q1-Q6 corresponds to Q12-Q17 in the survey (see additional file 1 for the questions).

Equivalence comparisons of graph types

AOM-Comprehension equivalence comparison between 3 intervention groups

Group 1	Group 2	Difference	95% CI
Pictograph	Bar graph	0.46	0.07 to 0.85
Pictograph	Line graph	0.43	0.03 to 0.84
Line graph	Bar graph	0.03	-0.40 to 0.45

Lateral epicondylitis- Comprehension equivalence comparison between 3 intervention groups

Group 1	Group 2	Difference	95% CI
Pictograph	Bar graph	0.28	-0.14 to 0.70
Pictograph	Line graph	0.26	-0.21 to 0.74
Line graph	Bar graph	0.01	-0.45 to 0.48

For peer review only

Association of comprehension with HL, numeracy, and education level

Trial A (Acute otitis media)

Variable	R2	Parameter Estimate	P-value	95% Confidence Limits	
Health literacy (categorised)	24%	High vs med 1.19	<.0001	0.86	1.51
		Low vs med -0.78	0.0007	-1.23	-0.33
Education	0%	0.05	0.47	-0.08	0.17
SNS	4%	0.33	<0.0001	0.19	0.48

Trial B (Lateral epicondylitis)

**Variable**	**R2**	**Parameter Estimate**	**P-value**	**95% Confidence Limits**	
**Health literacy (categorised)**	**29%**	**High vs med 1.28**	**<.0001**	**0.94**	**1.61**
		**Low vs med -1.29**	**<0.0001**	**-1.77**	**-0.81**
**Education**	**2%**	**0.21**	**0.0024**	**0.07**	**0.34**
**SNS**	**3%**	**0.3**	**0.0001**	**0.15**	**0.45**

For peer review only

Change of decision intention by intervention[1]

Trial A (Acute otitis media)

Acute condition - Acute otitis media (AOM) - Text only

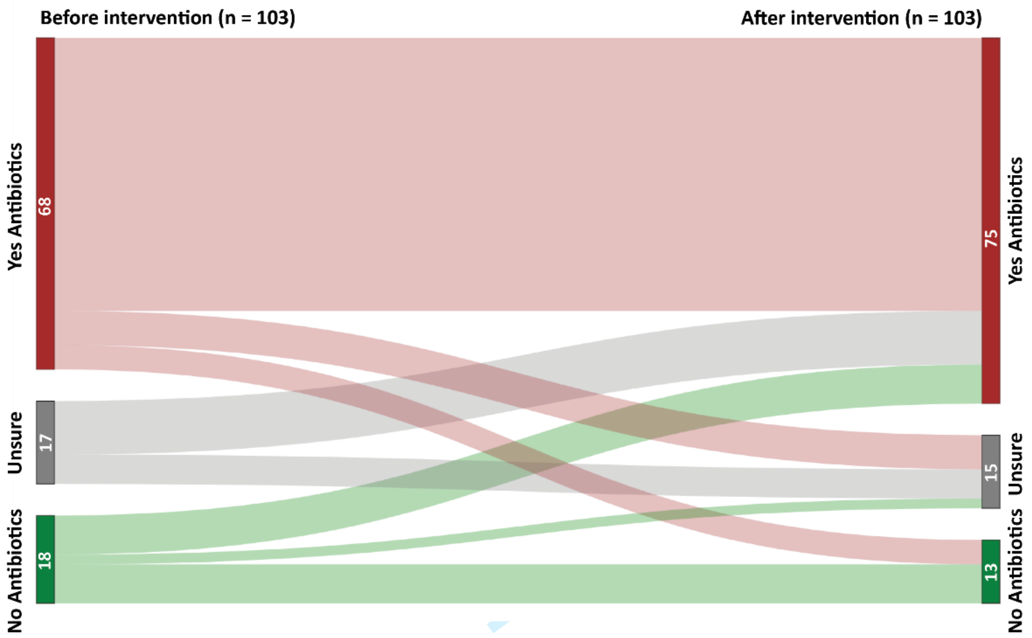

Acute condition - Acute otitis media (AOM) - Pictograph

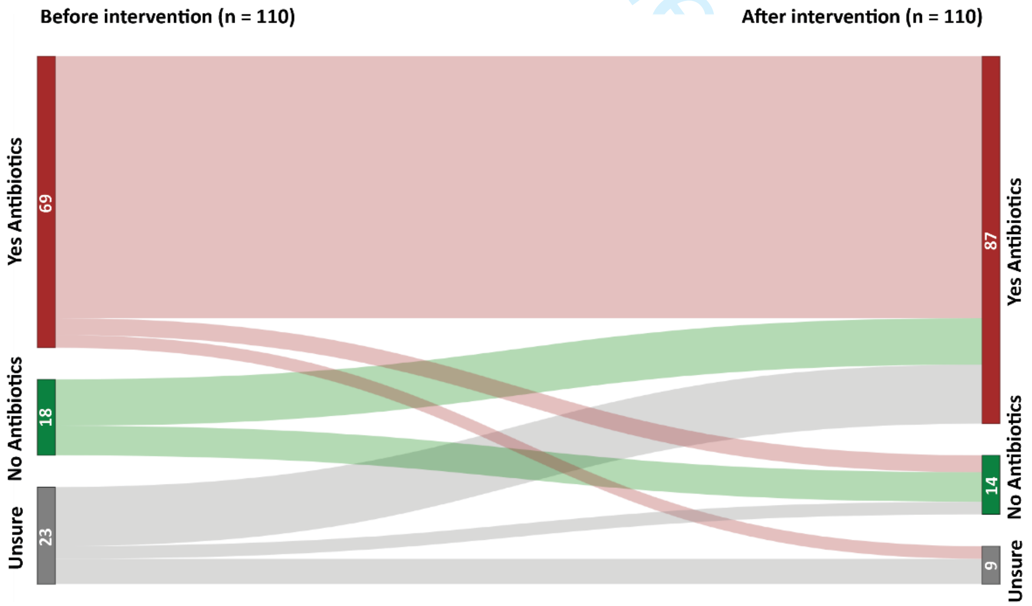

[1] Numbers and percentages of each choice before and after the interventions are presented in table 3 in the main article

For peer review only - http://bmjopen.bmj.com/site/about/guidelines.xhtml

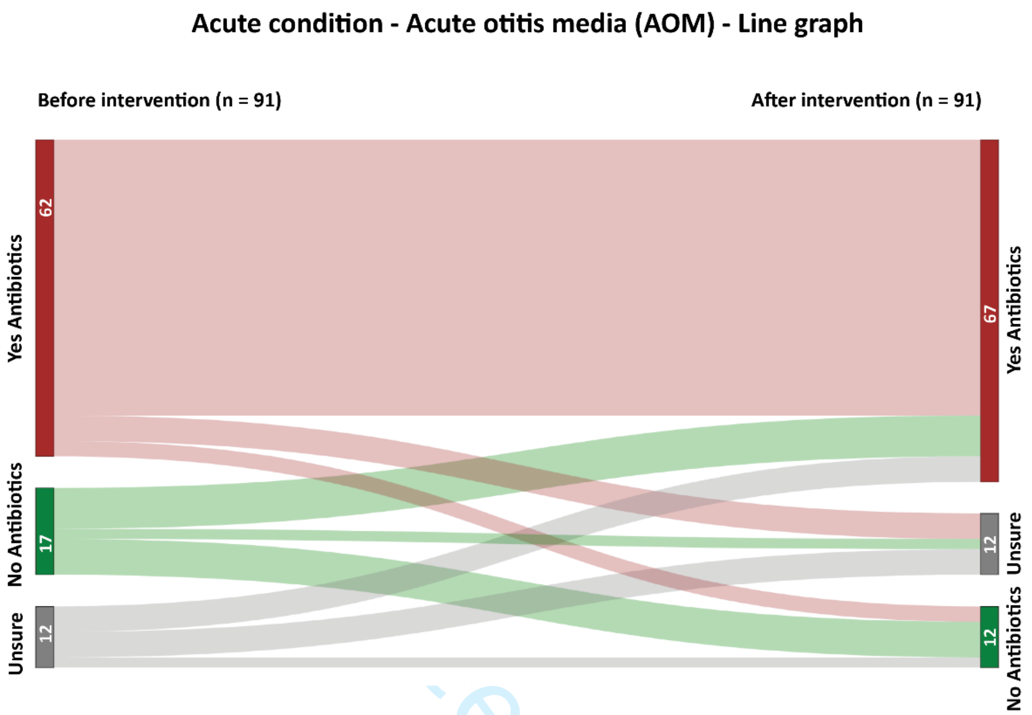
Acute condition - Acute otitis media (AOM) - Line graph
Before intervention (n = 91)
After intervention (n = 91)
Yes Antibiotics
62
No Antibiotics
17
Unsure
12
67
Yes Antibiotics
12
Unsure
12
No Antibiotics

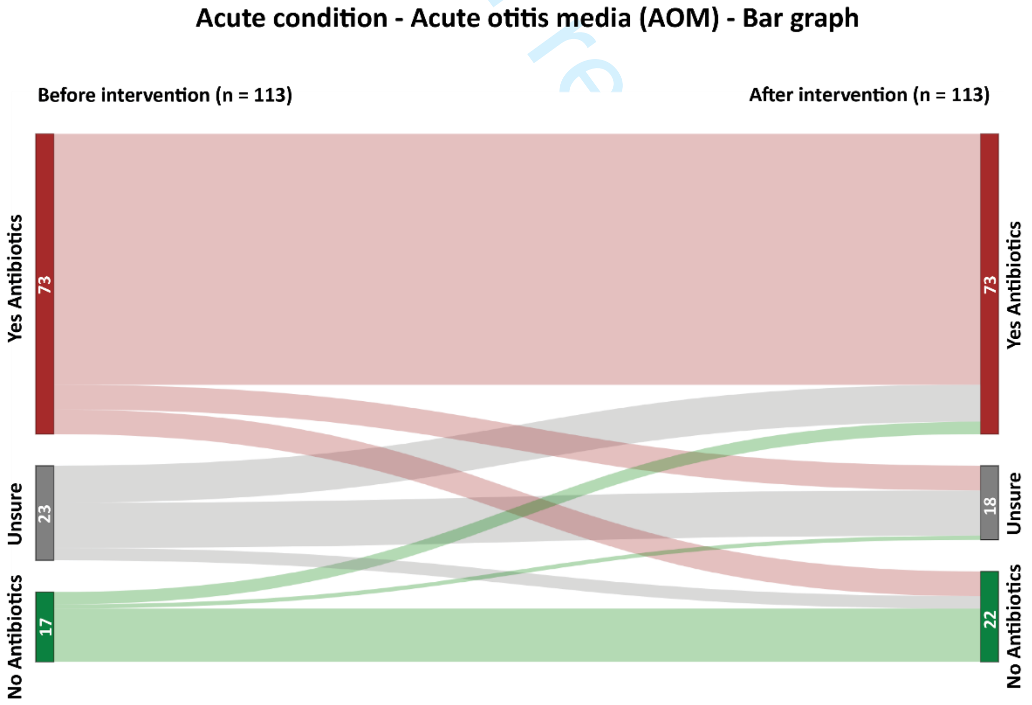
Acute condition - Acute otitis media (AOM) - Bar graph
Before intervention (n = 113)
After intervention (n = 113)
Yes Antibiotics
73
Unsure
23
No Antibiotics
17
73
Yes Antibiotics
18
Unsure
22
No Antibiotics

Trial B (Lateral epicondylitis)

Chronic condition - Lateral epicondylitis - Text only

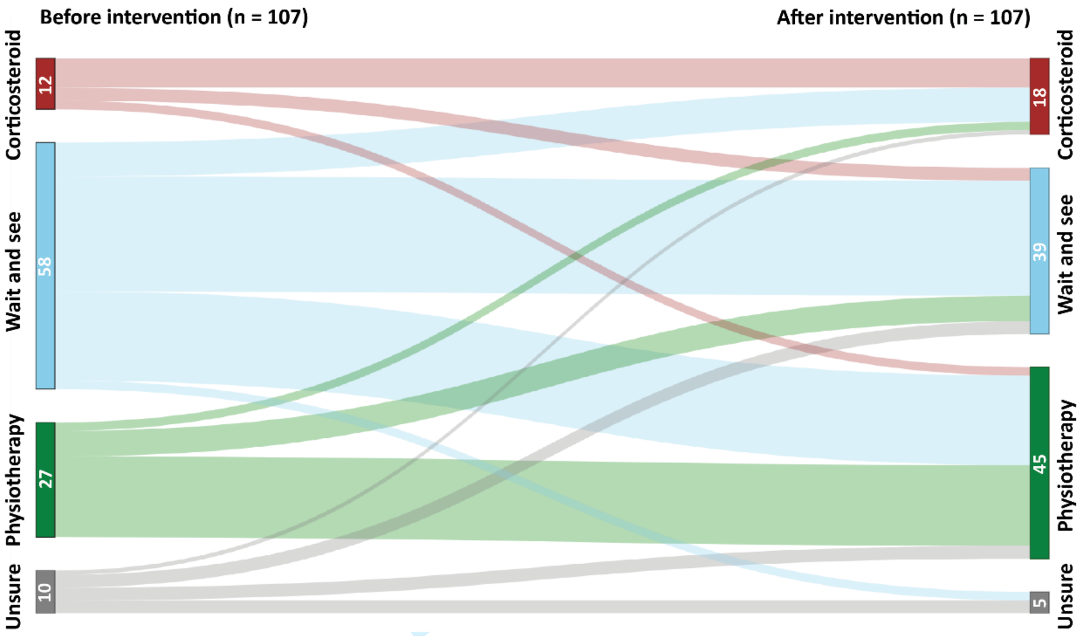

Chronic condition - Lateral epicondylitis - Pictograph

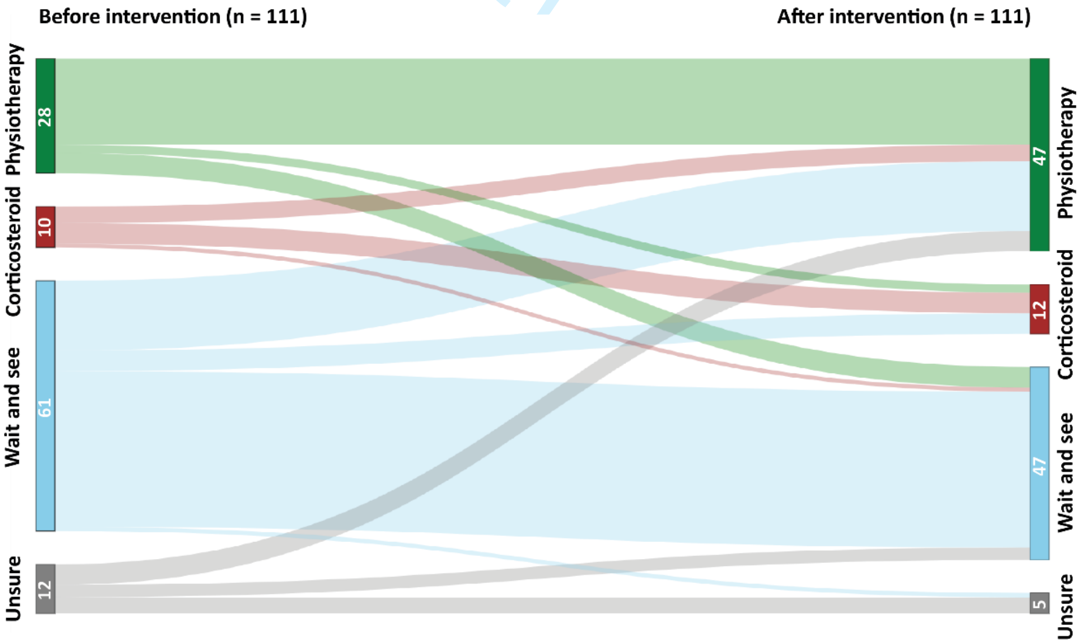

For peer review only - http://bmjopen.bmj.com/site/about/guidelines.xhtml

Chronic condition - Lateral epicondylitis - Bar graph

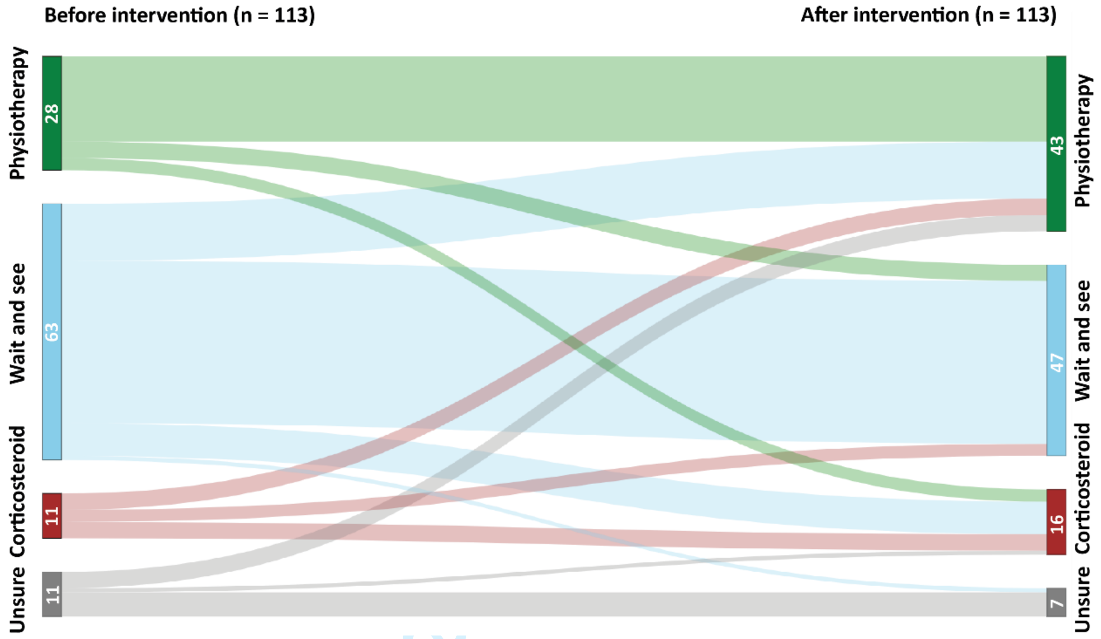

Chronic condition - Lateral epicondylitis - Line graph

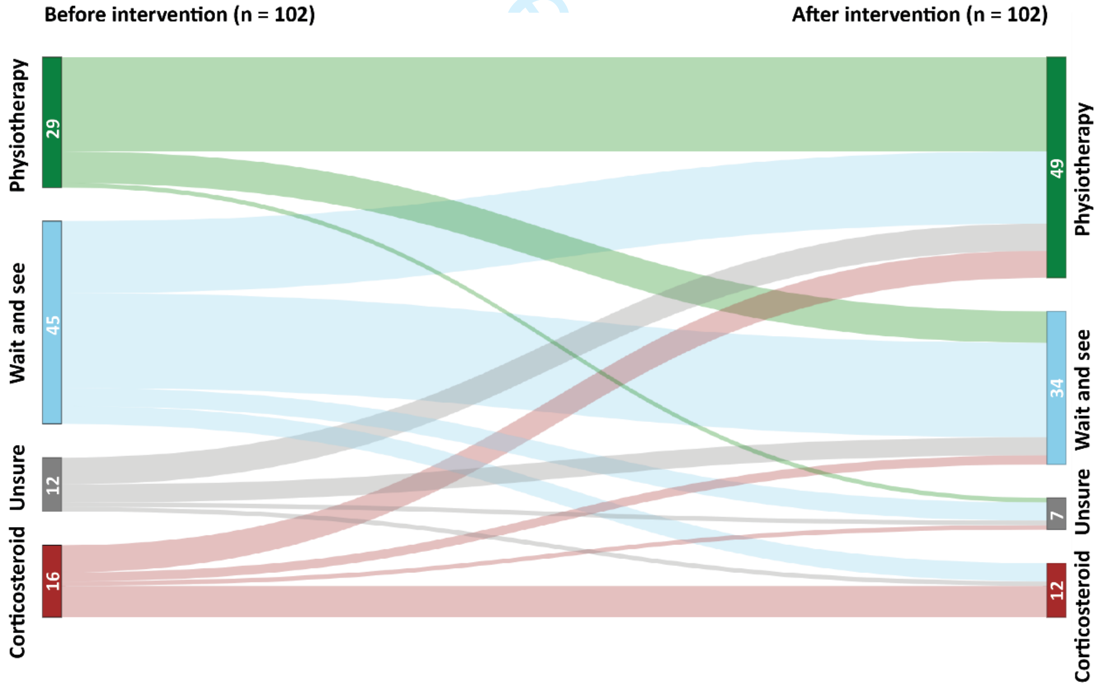

Reasons for decision intention Q19

Trial A (Acute otitis media)

**Question 19: Can you please indicate the most important reason for your decision?**					
**Reasons of participants who chose to give antibiotics**					
	**Category**	**Description**	**N total**	**Count/ intervention**	**Examples**
1	Based on the presented data	participants who thought data was favouring AB use or referred that their answers were based on the presented data	39	Text-only: 12<br>Bar graph: 9<br>Pictograph: 9<br>Line graph: 9	"the graph indicated that antibiotics has good effects"<br>"data proved fewer children in pain who took antibotics"
2	Antibiotics are effective	Decreased pain<br>Getting better<br>Reduce symptoms	145	Text-only: 33<br>Bar graph: 38<br>Pictograph: 43<br>Line graph: 31	"It helps relieve the pain for the child and fight the infection"<br>"Antibiotics will probably make slow down the infection and also pain."
3	Antibiotics save time	Decrease pain duration, quick recovery from illness, clear the infection faster	45	Text-only: 13<br>Bar graph: 11<br>Pictograph: 11<br>Line graph: 9	"quicker recovery"<br>"Because it helps it get better quickly"<br>"get rid of the pain faster"
4	Antibiotics prevent complications, concerns about complications if antibiotics were not used	Prevent superadded infections, prevent hearing loos, prevent eardrum perforation, prevent recurrence	50	Text-only: 12<br>Bar graph: 12<br>Pictograph: 17<br>Line graph: 9	"to prevent a perforated eardrum"<br>"it is important for your health and to avoid complications"<br>"The child would probably not be able to eliminate the infection themselves and this could possible lead to deafness or even death left untreated."<br>"To ensure that the infection doesnt get worse and permanently affect their hearing"
5	Following medical advice	The GPs decides if I should take antibiotics, or if necessary	14	Text-only: 3<br>Bar graph: 4<br>Pictograph: 4<br>Line graph: 3	"take it only if the doctor says"<br>"I rely on Medical Advice as to how to proceed in situations like this."
6	Based on personal experience or perspectives	Participants who had either negative/positive previous experience with the condition when they used/not antibiotics	21	Text-only: 7<br>Bar graph: 3<br>Pictograph: 4<br>Line graph: 6	"ive been a sufferer of inner ear infections so have my children antis are the only way for us"<br>"have had a child with ear infection, very painful, and always needed antibiotics"
7	Antibiotics are necessary/ needed	Participants who believed antibiotics are needed anyway, e.g., antibiotics work wonder	24	Text-only: 4<br>Bar graph: 5<br>Pictograph: 10<br>Line graph: 5	"you must take antibiotics to stop the infection"<br>"You have to treat an infection."<br>"pain is acute and the ear wont clear itself up"
8	Conditional use of antibiotics	Participants who think they would use antibiotics if pain was severe, or if there is fever, it took longer than expected, if my child is suffering	22	Text-only: 5<br>Bar graph: 5<br>Pictograph: 7<br>Line graph: 5	"Depends on the severity of the case."<br>"if he has fever"

For peer review only - http://bmjopen.bmj.com/site/about/guidelines.xhtml

Reasons of participants who chose not to give antibiotics					
	**Category**	**Description**	**N total**	**Count/ intervention**	**Example**
1	Based on the presented data	participants who thought that the presented data was not favouring antibiotic use	20	Text-only: 5<br>Bar graph: 8<br>Pictograph: 2<br>Line graph: 5	"previous chart showed only small difference"<br>"The statistics don't support antibiotic use for this condition"
2	Based on their preference of other alternatives	Participants who preferred to use alternative options (other medicines, home remedies) or to wait and see if it goes on its own	13	Text-only: 0<br>Bar graph: 5<br>Pictograph: 4<br>Line graph: 4	"In many case home remedy will take care of this problem."<br>"there may be other way to solve this problem"
3	Based on personal preference	Participants who didn't give a specific reason e.g. I like this option	4	Text-only: 2<br>Pictograph: 1<br>Pictograph: 1<br>Line graph: 0	"i think its helpful"<br>"because I like it"
4	Concerns about antibiotic overuse and resistance	Participants who related their decision to the effect of overuse of antibiotics	11	Text-only: 0<br>Bar graph: 7<br>Pictograph: 4<br>Line graph: 0	"not worth becoming immune to antibiotics over a few percentage points."<br>"Because of growing immunity to anibiotics it is always best to avoid them if they are not stricty necessary"
5	Concerns about adverse events	Participants who were worried about the side effects of antibiotics	18	Text-only: 1<br>Bar graph: 8<br>Pictograph: 5<br>Line graph: 4	"Because antibiotics has side effects and most children have self antibody with which he/she can immune himself/herself."<br>"antibiotics can cause allergic reactions in kids"
6	Conditional use of antibiotics	Depends on severity of symptoms, or if prescribed by a GP	12	Text-only: 3<br>Bar graph: 4<br>Pictograph: 4<br>Line graph: 1	"Better not to take antibiotics unless it was really necessary"<br>"wait for doctor decision"
7	Antibiotics have little benefit/ not effective	Participants who thought antibiotics are not of great benefit in this case or in general	11	Text-only: 4<br>Bar graph: 3<br>Pictograph: 1<br>Line graph: 3	"Because generally middle ear infections are viral therefore do not need antibiotics"

For peer review only - http://bmjopen.bmj.com/site/about/guidelines.xhtml

Reasons of participants who were unsure					
	**Category**	**Description**	**N total**	**Count/ intervention**	**Example**
1	Based on the presented data	participants who can't understand the data presented/ need	8	Text-only: 1<br>Bar graph: 4<br>Pictograph: 2<br>Line graph: 1	"very little difference between taking and not taking drugs"<br>"did not understand"<br>"its hard to figure it"
2	Based on their preference of other alternatives	Participants who preferred to use alternative options (other medicines, home remedies), wait and see or having no treatment	6	Text-only: 1<br>Bar graph: 2<br>Pictograph: 2<br>Line graph: 1	"I think it would depend on how severe the infection is, and I would also look for a natural option opposed to anitbiotics"<br>"theres a fine line between the two,,, i prefer to wait a few days and see what eventuates,, not a big fan of antibiotics"
3	Lack of personal knowledge or experience	Participants who had no previous experience of the condition or knowledge about it	5	Text-only: 2<br>Bar graph: 2<br>Pictograph: 0<br>Line graph: 1	"None of my children have ever had a middle ear infection and they are all grown up now"<br>"I do not have children"
4	Concerns about side effects including resistance	Participants who were unsure because they were worried about side effect of using antibiotics	4	Text-only: 0<br>Bar graph: 0<br>Pictograph: 0<br>Line graph: 3	"medication causes the problem"<br>"It will depend on the child's other medical issues and the parents consent. Some children given antibiotics can have serious issues caused by them"
5	Conditional use, depends on severity, …etc	Participants who think using antibiotics depends on individual cases or on specific symptoms or causes	14	Text-only: 5<br>Bar graph: 4<br>Pictograph: 2<br>Line graph: 3	"depends on the history of the indvidual child"<br>"depends if the cause is bacterial"
6	Need more information	Participants who needed more information to make an informed decision	4	Text-only: 1<br>Bar graph: 1<br>Pictograph: 2<br>Line graph: 0	"i can not tell base on just one study"<br>"would need more details how long they have had it , if its reacurring etc"
7	Follow medical advice	Participants who would follow what the doctors say either to take antibiotics or not	9	Text-only: 3<br>Bar graph: 2<br>Pictograph: 0<br>Line graph: 4	"I prefer to consult a doctor first before answering the question."<br>"would consult a doctor"
8	Others	Participants who were unsure, don't know	11	Text-only: 3<br>Bar graph: 5<br>Pictograph: 1<br>Line graph: 2	"I don't know"<br>"Dont have any"

For peer review only

Trial B (Lateral epicondylitis)

Q19- Can you please indicate the most important reason for your decision?					
**Reasons of participants who chose corticosteroid injection**					
	**Category**	**Description**	**N Total**	**Count/ intervention**	**Examples**
1	Based on my understanding of the presented data	Participants who based their answers to their understanding of the presented data	24	Bar graph: 6<br>Pictograph: 5<br>Line graph: 3<br>Text only: 10	"The data shows that it has the best outcome of the three."<br>"Ease them immediate pain, best chance of recovery in the short term"
2	Based on previous experience	Based on the participants own experience with the treatment chosen or negative experience with the alternative treatments either personally or heard about it (e.g., someone they know)	12	Bar graph: 5<br>Pictograph: 3<br>Line graph: 3<br>Text only: 1	"because i have this condition and so far it works best for me"<br>"had previous cortizone injection"
3	Avoiding complications or side effects of alternative options/ against other options or personal preference	Participants who chose corticosteroid injection to avoid any complications that might happen if they choose the other options. either based on previous experience or expectation or as a personal preference	13	Bar graph: 2<br>Pictograph: 4<br>Line graph: 3<br>Text only: 4	"i dont believe in waiting to see if something get better or worse, i prefer this as i have had in the past"<br>"no physio here"
4	Corticosteroid offer a speedy recovery	Patients who chose steroid because it has better outcome on the short term	13	Bar graph: 4<br>Pictograph: 4<br>Line graph: 1<br>Text only: 4	"fast recovery chances"<br>"Time is not on my side, so I would take the speediect option"
5	This treatment is effective	Participants who thought this option would be effective	24	Bar graph: 7<br>Pictograph: 2<br>Line graph: 4<br>Text only: 11	"More people recover by using this method"<br>"to help cure the tennis elbow"
3 irrelevant responses were not categorized					

For peer review only - http://bmjopen.bmj.com/site/about/guidelines.xhtml

Reasons of participants who chose physiotherapy					
	**Category**	**Description**	**N Total**	**Count/intervention**	**Examples**
1	Based on my understanding of the presented data	Participants who based their answers to their understanding of the presented data	96	Bar graph: 20<br>Pictograph: 25<br>Line graph: 23<br>Text only: 28	"As the graphs show Physiotheraphy works"<br>"According to the graph patients getting physiotherapy recovers more quickly."
2	Based on previous experience and personal preference	Based on participants' own experience with the treatment chosen or negative experience with the alternative treatments either personally or heard about it (e.g., someone they know)	33	Bar graph: 10<br>Pictograph: 8<br>Line graph: 9<br>Text only: 6	"had a corticosteroid for my shoulder once willnever get one of those again it hurt all the time"<br>"I had it and it was working great"
3	Avoiding complications of the condition or side effects of alternative options/ against other options	Participants who chose corticosteroid injection to avoid any complications that might happen if they choose the other options either based on previous experience or expectation	13	Bar graph: 2<br>Pictograph: 2<br>Line graph: 3<br>Text only: 6	"drug free"<br>"I am doing something about it rather than waiting and watching and physiotherapy is more natural treatment than injecting myself"
4	A more natural option with less side effects	Participants who thought this option is safer and alleviate the side effects of other options	20	Bar graph: 3<br>Pictograph: 5<br>Line graph: 5<br>Text only: 7	"less invasive and high chance of recovery"<br>"It seems to be the best and least-invasive treatment option"
5	I would follow medical/GP advice	Participants who thought seeking medical advice is the right thing to do	6	Bar graph: 3<br>Pictograph: 0<br>Line graph: 3<br>Text only: 0	"get an expert to help with any pain or swelling"<br>"see a doctor sooner"
6	Physiotherapy is better on the long term	Participants who based their response mainly on the long-term recovery rate	12	Bar graph: 3<br>Pictograph: 2<br>Line graph: 2<br>Text only: 5	"Seems to be the best long term option"<br>"The long-term outcome"
7	Based on both short term and long-term effect	Participants who compared effectiveness both on the short term and long term	12	Bar graph: 1<br>Pictograph: 5<br>Line graph: 4<br>Text only: 2	"Best chance of short and long term recovery."<br>"Physiotherapy had a better long term result compared to the others which had a more of a short term solution"
8	Treatment is effective	Participants who thought this option is effective	94	Bar graph: 15<br>Pictograph: 28<br>Line graph: 27<br>Text only: 24	"I think it would help recover"<br>"Physio includes execises which help recovery and future protection"
9	Physiotherapy provides quicker recovery	Participants who believed physiotherapy will provide faster/ quicker recovery	33	Bar graph: 7<br>Pictograph: 9<br>Line graph: 6<br>Text only: 11	"To treat it quicker"<br>"to recover faster"

For peer review only - http://bmjopen.bmj.com/site/about/guidelines.xhtml

Reasons of participants who chose to wait and watch					
	**Category**	**Description**	**N Total**	**Count/intervention**	**Examples**
1	Based on my understanding of the presented data	Participants who based their answers to their understanding of the presented data	45	Text only: 9<br>Bar graph: 14<br>Pictograph: 15<br>Line graph:7	"Because the data shows that time will help heal the condition, and that although physiotherapy helped, doing nothing ultimately has the same outcome."
2	Based on previous experience	Based on the participants own experience with the treatment chosen or negative experience with the alternative treatments either personally or heard about it (e.g., someone they know)	15	Text only: 2<br>Bar graph: 6<br>Pictograph: 3<br>Line graph:4	"previous history and treatment"<br>"never had success with physio and other health issues make corticosteroid injection risky"
3	Avoiding complications of alternative options/ against other options	Participants who chose corticosteroid injection to avoid any complications that might happen if they choose the other options either based on previous experience or expectation	21	Text only: 3<br>Bar graph: 5<br>Pictograph: 8<br>Line graph:5	"I have tennis elbow now, I tend to let it run its course. I don't see the value in Physio vs time, and Cortisone damages the bone"<br>"i dont really like injections and physio is expensive"
4	A more natural option with less side effects	Participants who thought this option is safer and alleviate the side effects of other options	56	Text only: 16<br>Bar graph: 14<br>Pictograph: 14<br>Line graph:12	"safe option"<br>"Don't believe in using drugs if it can be avoided."
5	Long term benefit	Participants who based their response mainly on the long-term recovery rate	16	Text only: 3<br>Bar graph: 8<br>Pictograph: 3<br>Line graph:2	"The long term outcome isn't different enough to make me think that one course of action is much better than another"
6	Treatment is effective	Participants who thought this option is effective	38	Text only: 8<br>Bar graph: 11<br>Pictograph: 11<br>Line graph: 8	"effective"<br>"more chance of recovery"
7	Cost free	Participants who chose this option to avoid costs of treatment either because it is free, their insurance would not cover other treatment expenses or they wouldn't wish to spend money to achieve very minimal better chance recovery with the other options	35	Text only: 6<br>Bar graph: 11<br>Pictograph: 11<br>Line graph:7	"If I can save money not going to the doctor right away, then I'll do it."<br>"This is primarily from a cost perspective as I do not have the optimal health insurance that covers the cost of physiotherapy"

For peer review only - http://bmjopen.bmj.com/site/about/guidelines.xhtml

8	Depends on nature/ severity of the disease	Participants who chose this option as first option but are open to chose different options depending on the severity and time of the condition	17	Text only: 6<br>Bar graph: 5<br>Pictograph: 5<br>Line graph:1	"It would depend on the level of pain/discomfort and functional impairment, but I generally feel I could handle it at home with exercise and possibly analgesics if necessary"
9	Avoidance of medical advice	Either don't trust GPs/ medical advice	11	Text only: 2<br>Bar graph: 3<br>Pictograph: 4<br>Line graph:2	"I don't trust the medical profession that much, always been a wait and see person"<br>"i hate doctors"
10	compared both short term and long-term effects	Participants who compared effectiveness both on the short term and long term	4	Text only: 0<br>Bar graph: 2<br>Pictograph: 1<br>Line graph:1	"Short term you might get better results from the injection but in the long term you just need to let it work out"

**Reasons of participants who were unsure**					
	**Category**	**Description**	**N Total**	**Count/intervention**	**Example**
1	No reason, don't know, unsure	Participants who had no reason for their choice	11	Text only: 1<br>Bar graph: 4<br>Pictograph: 3<br>Line graph:3	"Unsure"<br>"no idea"
2	Conditional choice	Depending on severity, work commitments, cost	8	Text only: 2<br>Bar graph: 3<br>Pictograph: 1<br>Line graph:2	"depen on how much it's pain"<br>"it would depend on situation and if I was working and how much it impacted my life"
3	Previous experience/ lack of personal experience	Based on the participants own experience with the treatment chosen or negative experience with the alternative treatments either personally or heard about it (e.g., someone they know)	2	Text only: 0<br>Bar graph: 0<br>Pictograph: 1<br>Line graph:1	"because I'm unsure how I would react to find out that's what I have."<br>" Hard question... I have bilateral epicondylitis in both arms and have tried all of the above so I know nothing works lol Probably watch and wait"
4	Follow medical advice	Participants who thought seeking medical advice is the right thing to do	3	Text only: 2<br>Bar graph: 0<br>Pictograph: 0<br>Line graph:1	"I would see my doctor and be advised by his medical expertise which way to receive treatment"<br>"would probably consult with my doctor for the right treatment"

For peer review only - http://bmjopen.bmj.com/site/about/guidelines.xhtml

Reasons for first choice preference Q23

Trial A (Acute otitis media)

Q23. In a few words, please tell us why you chose the option that you ranked the highest (1)?		
	**Participants who chose text-only first**	
	**Explanation**	**Count**
0	No reason, Unsure, I don't know	6
1	It is easy to understand, clear, concise or it is just good	84
2	It is better than other presentations and easier to compare the different options in this presentation	25
3	It is my personal preference, or the presentation that I am more familiar with or appeals to me and my understanding the best	14
4	Less effort and time consuming to understand the information	3

	**Participants who chose bar graph first**	
	**Explanation**	**Count**
0	No reason, Unsure, I don't know	2
1	It is easy to understand, clear, concise or it is just good	71
2	It is better than other presentations and easier to compare the different options in this presentation	40
3	It is my personal preference, or the presentation that I am more familiar with or appeals to me and my understanding the best	9
4	Less effort and time consuming to understand the information	4
5	Visually better presented, I can understand the information at a glance. The colours are easy to distinguish	27
6	Better in showing the progress, prognosis, trend over time	0

	**Participants who chose pictograph first**	
	**Explanation**	**Count**
0	No reason, Unsure, I don't know	0
1	It is easy to understand, clear, concise or it is just good	32
2	It is better than other presentations and easier to compare the different options in this presentation	14
3	It is my personal preference, or the presentation that I am more familiar with or appeals to me and my understanding the best	1
4	Less effort and time consuming to understand the information	3
5	Visually better presented, I can understand the information at a glance. The colours are easy to distinguish	15
6	Better in showing the progress, prognosis, trend over time	0

For peer review only - http://bmjopen.bmj.com/site/about/guidelines.xhtml

	Participants who chose line graph first	
	**Explanation**	**Counts**
0	No reason, Unsure, I don't know	3
1	It is easy to understand, clear, concise or it is just good	54
2	It is better than other presentations and easier to compare the different options in this presentation	27
3	It is my personal preference, or the presentation that I am more familiar with or appeals to me and my understanding the best	1
4	Less effort and time consuming to understand the information	5
5	Visually better presented, I can understand the information at a glance. The colours are easy to distinguish	18
6	Better in showing the progress, prognosis, trend over time	11

For peer review only

Trial B (Lateral epicondylitis)

Q23. In a few words, please tell us why you chose the option that you ranked the highest (1)?

	**Participants who chose text-only first**	
	**Explanation**	**Counts**
0	No reason, Unsure, I don't know	9
1	It is easy to understand, clear, concise or it is just good	67
2	It is better than other presentations and easier to compare the different options in this presentation	54
3	It is my personal preference, or the presentation that I am more familiar with or appeals to me and my understanding the best	16
4	Less effort and time consuming to understand the information	15

	**Participants who chose pictograph first**	
	**Explanation**	**Counts**
0	No reason, Unsure, I don't know	5
1	It is easy to understand, clear, concise or it is just good	25
2	It is better than other presentations and easier to compare the different options in this presentation	24
3	It is my personal preference, or the presentation that I am more familiar with or appeals to me and my understanding the best	3
4	Less effort and time consuming to understand the information	2
5	Visually better presented, I can understand the information at a glance. The colours are easy to distinguish	16
6	Better in showing the progress, prognosis, trend over time	0

	**Participants who chose bar graph first**	
	**Explanation**	**Counts**
0	No reason, Unsure, I don't know	3
1	It is easy to understand, clear, concise or it is just good	55
2	It is better than other presentations and easier to compare the different options in this presentation	68
3	It is my personal preference, or the presentation that I am more familiar with or appeals to me and my understanding the best	6
4	Less effort and time consuming to understand the information	8
5	Visually better presented, I can understand the information at a glance. The colours are easy to distinguish	28
6	Better in showing the progress, prognosis, trend over time	2

For peer review only - http://bmjopen.bmj.com/site/about/guidelines.xhtml

	Participants who chose line graph first	
	**Explanation**	**Counts**
0	No reason, Unsure, I don't know	1
1	It is easy to understand, clear, concise or it is just good	28
2	It is better than other presentations and easier to compare the different options in this presentation	26
3	It is my personal preference, or the presentation that I am more familiar with or appeals to me and my understanding the best	5
4	Less effort and time consuming to understand the information	4
5	Visually better presented, I can understand the information at a glance. The colours are easy to distinguish	2
6	Better in showing the progress, prognosis, trend over time	17

For peer review only

For peer review only - http://bmjopen.bmj.com/site/about/guidelines.xhtml

Exploratory results

Association between comprehension and MMM, time to complete comprehension questions, and graph preference

Trial A (Acute otitis media)

Variable	R2	Parameter Estimate	P-value	95% Confidence Limits	Variable
MMM scale (categorised)	2%	High vs med -0.60	0.01	-1.05	-0.14
		Low vs med -0.18	0.3	-0.52	0.16
Time to complete comprehension questions (mins)	2%	0.02	0.0066	0.01	0.04
Graph preference	3%	Bar vs text: 0.63	0.0008	0.27	1
		Picto vs text: 0.69	0.0045	0.21	1.16
		Line vs text: 0.52	0.011	0.12	0.92

Trial B (Lateral epicondylitis)

Variable	R2	Parameter Estimate	P-value	95% Confidence Limits	Variable
MMM scale (categorised)	3%	High vs med -0.81	0.0009	-1.28	-0.33
		Low vs med -0.07	0.71	-0.43	0.29
Time to complete comprehension questions (mins)	1%	0.03	0.053	0	0.05
Graph preference	4%	Bar vs text: 0.63	0.0011	0.25	1
		Picto vs text: 0.65	0.0077	0.17	1.12
		Line vs text: 0.84	0.0006	0.37	1.32

For peer review only - http://bmjopen.bmj.com/site/about/guidelines.xhtml

Overall Visual presentation preference by graph experience

Trial A (Acute otitis media)

Preference	N	Previous experience	N	Mean	Std Dev	Minimum	Maximum
Text only	124.0	Bar graph	124.0	2.9	1.2	1	5
		Pictograph	124.0	2.4	1.2	1	5
		Line graph	124.0	3.2	1.3	1	5
Bar graph	137.0	Bar graph	137.0	3.7	1.2	1	5
		Pictograph	137.0	2.5	1.2	1	5
		Line graph	137.0	3.4	1.2	1	5
Pictograph	58.0	Bar graph	58.0	3.7	1.0	1	5
		Pictograph	58.0	3.6	1.1	1	5
		Line graph	58.0	3.8	0.9	2	5
Line graph	98.0	Bar graph	98.0	3.2	1.2	1	5
		Pictograph	98.0	2.6	1.2	1	5
		Line graph	98.0	4.1	1.1	1	5

Trial B (Lateral epicondylitis)

Preference	N	Previous experience	N	Mean	Std Dev	Minimum	Maximum
Text only	144	Bar graph	144	2.92	1.18	1	5
		Pictograph	144	2.22	1.22	1	5
		Line graph	144	2.89	1.2	1	5
Bar graph	154	Bar graph	154	3.96	1.07	1	5
		Pictograph	154	2.48	1.19	1	5
		Line graph	154	3.51	1.12	1	5
Pictograph	68	Bar graph	68	3.71	1.12	1	5
		Pictograph	68	3.49	1.31	1	5
		Line graph	68	3.78	1.09	1	5
Line graph	67	Bar graph	67	3.64	1.05	1	5
		Pictograph	67	2.63	1.23	1	5
		Line graph	67	4.15	1.09	1	5

For peer review only

Items to include when reporting a randomized trial in a journal or conference abstract

Item	Description	Reported on line number
Title	Identification of the study as randomized	1-2
Authors *	Contact details for the corresponding author	4-20
Trial design	Description of the trial design (e.g. parallel, cluster, non-inferiority)	40, 42-43
Methods		
Participants	Eligibility criteria for participants and the settings where the data were collected	44-48
Interventions	Interventions intended for each group	49-53
Objective	Specific objective or hypothesis	38-41
Outcome	Clearly defined primary outcome for this report	54
Randomization	How participants were allocated to interventions	In the main article
Blinding (masking)	Whether or not participants, care givers, and those assessing the outcomes were blinded to group assignment	In the main article
Results		
Numbers randomized	Number of participants randomized to each group	45-48
Recruitment	Trial status	completed
Numbers analysed	Number of participants analysed in each group	45-48
Outcome	For the primary outcome, a result for each group and the estimated effect size and its precision	57-63
Harms	Important adverse events or side effects	No harms
Conclusions	General interpretation of the results	64-67
Trial registration	Registration number and name of trial register	68
Funding	Source of funding	92-95

**this item is specific to conference abstracts*

For peer review only - http://bmjopen.bmj.com/site/about/guidelines.xhtml

CONSORT 2010 checklist of information to include when reporting a randomised trial*

Section/Topic	Item No	Checklist item	Reported on page No
**Title and abstract**			
	1a	Identification as a randomised trial in the title	1
	1b	Structured summary of trial design, methods, results, and conclusions (for specific guidance see CONSORT for abstracts)	2-3
**Introduction**			
Background and objectives	2a	Scientific background and explanation of rationale	4-5
	2b	Specific objectives or hypotheses	5
**Methods**			
Trial design	3a	Description of trial design (such as parallel, factorial) including allocation ratio	5
	3b	Important changes to methods after trial commencement (such as eligibility criteria), with reasons	9
Participants	4a	Eligibility criteria for participants	5
	4b	Settings and locations where the data were collected	5
Interventions	5	The interventions for each group with sufficient details to allow replication, including how and when they were actually administered	6-7
Outcomes	6a	Completely defined pre-specified primary and secondary outcome measures, including how and when they were assessed	7-8
	6b	Any changes to trial outcomes after the trial commenced, with reasons	NA
Sample size	7a	How sample size was determined	8
	7b	When applicable, explanation of any interim analyses and stopping guidelines	NA
Randomisation:			
Sequence generation	8a	Method used to generate the random allocation sequence	6
	8b	Type of randomisation; details of any restriction (such as blocking and block size)	6
Allocation concealment mechanism	9	Mechanism used to implement the random allocation sequence (such as sequentially numbered containers), describing any steps taken to conceal the sequence until interventions were assigned	6

For peer review only

Implementation	10	Who generated the random allocation sequence, who enrolled participants, and who assigned participants to interventions	6
Blinding	11a	If done, who was blinded after assignment to interventions (for example, participants, care providers, those assessing outcomes) and how	10
	11b	If relevant, description of the similarity of interventions	6-7
Statistical methods	12a	Statistical methods used to compare groups for primary and secondary outcomes	9
	12b	Methods for additional analyses, such as subgroup analyses and adjusted analyses	9-10
**Results**			
Participant flow (a diagram is strongly recommended)	13a	For each group, the numbers of participants who were randomly assigned, received intended treatment, and were analysed for the primary outcome	Figure 3
	13b	For each group, losses and exclusions after randomisation, together with reasons	Figure 3
Recruitment	14a	Dates defining the periods of recruitment and follow-up	5
	14b	Why the trial ended or was stopped	NA
Baseline data	15	A table showing baseline demographic and clinical characteristics for each group	Table 1
Numbers analysed	16	For each group, number of participants (denominator) included in each analysis and whether the analysis was by original assigned groups	Tables and figures
Outcomes and estimation	17a	For each primary and secondary outcome, results for each group, and the estimated effect size and its precision (such as 95% confidence interval)	10-14, and tables
	17b	For binary outcomes, presentation of both absolute and relative effect sizes is recommended	NA
Ancillary analyses	18	Results of any other analyses performed, including subgroup analyses and adjusted analyses, distinguishing pre-specified from exploratory	Additional file 2
Harms	19	All important harms or unintended effects in each group (for specific guidance see CONSORT for harms)	Ethics application
**Discussion**			
Limitations	20	Trial limitations, addressing sources of potential bias, imprecision, and, if relevant, multiplicity of analyses	14-15
Generalisability	21	Generalisability (external validity, applicability) of the trial findings	15-16

For peer review only

Interpretation	22	Interpretation consistent with results, balancing benefits and harms, and considering other relevant evidence	14-17
**Other information**			
Registration	23	Registration number and name of trial registry	5
Protocol	24	Where the full trial protocol can be accessed, if available	In the registry
Funding	25	Sources of funding and other support (such as supply of drugs), role of funders	18

*We strongly recommend reading this statement in conjunction with the CONSORT 2010 Explanation and Elaboration for important clarifications on all the items. If relevant, we also recommend reading CONSORT extensions for cluster randomised trials, non-inferiority and equivalence trials, non-pharmacological treatments, herbal interventions, and pragmatic trials. Additional extensions are forthcoming: for those and for up to date references relevant to this checklist, see www.consort-statement.org.

For peer review only
